# Integrated analysis of microbiome and host transcriptome reveals correlations between gut microbiota and clinical outcomes in HBV-related hepatocellular carcinoma

**DOI:** 10.1186/s13073-020-00796-5

**Published:** 2020-11-23

**Authors:** Hechen Huang, Zhigang Ren, Xingxing Gao, Xiaoyi Hu, Yuan Zhou, Jianwen Jiang, Haifeng Lu, Shengyong Yin, Junfang Ji, Lin Zhou, Shusen Zheng

**Affiliations:** 1 Division of Hepatobiliary and Pancreatic Surgery, Department of Surgery, The First Affiliated Hospital, Zhejiang University School of Medicine, #79 Qingchun Road, Hangzhou, 310003 China; 2 NHFPC Key Laboratory of Combined Multi-organ Transplantation, 310003, Hangzhou, China; 3 Key Laboratory of Organ Transplantation, Hangzhou, Zhejiang Province China; 4 Department of Infectious Diseases, the First Affiliated Hospital of Zhengzhou University, Zhengzhou, China; 5 State Key Laboratory for Diagnosis and Treatment of Infectious Disease, Collaborative Innovation Center for Diagnosis and Treatment of Infectious Diseases, Zhejiang University, Hangzhou, China; 6 MOE key Laboratory of Biosystems Homeostasis & Protection, Life Sciences Institute, Zhejiang University, Hangzhou, China

**Keywords:** Gastrointestinal microbiome, Transcriptome, Prognosis, Carcinoma, hepatocellular

## Abstract

**Background:**

The gut-liver axis plays a pivotal role in the pathogenesis of hepatocellular carcinoma (HCC). However, the correlations between the gut microbiome and the liver tumor transcriptome in patients with HCC and the impact of the gut microbiota on clinical outcome are less well-understood.

**Methods:**

Fecal samples collected from HBV-related HCC patients (*n* = 113) and healthy volunteers (*n* = 100) were subjected to 16S rRNA sequencing of the microbiome. After a rigorous selection process, 32 paired tumor and adjacent non-tumor liver tissues from the HCC group were subjected to next-generation sequencing (NGS) RNA-seq. The datasets were analyzed individually and integrated with clinical characteristics for combined analysis using bioinformatics approaches. We further verified the potential of the gut microbiota to predict clinical outcome by a random forest model and a support vector machine model.

**Results:**

We found that *Bacteroides*, *Lachnospiracea incertae sedis*, and *Clostridium XIVa* were enriched in HCC patients with a high tumor burden. By integrating the microbiome and transcriptome, we identified 31 robust associations between the above three genera and well-characterized genes, indicating possible mechanistic relationships in tumor immune microenvironment. Clinical characteristics and database analysis suggested that serum bile acids may be important communication mediators between these three genera and the host transcriptome. Finally, among these three genera, six important microbial markers associated with tumor immune microenvironment or bile acid metabolism showed the potential to predict clinical outcome (AUC = 81%).

**Conclusions:**

This study revealed that changes in tumor immune microenvironment caused by the gut microbiota via serum bile acids may be important factors associated with tumor burden and adverse clinical outcome. Gut microbes can be used as biomarkers of clinical features and outcomes, and the microbe-associated transcripts of host tumors can partly explain how gut microbiota promotes HCC pathogenesis.

**Supplementary information:**

**Supplementary information** accompanies this paper at 10.1186/s13073-020-00796-5.

## Background

Primary liver cancer, including hepatocellular carcinoma (HCC) (80% of cases), was the fourth leading cause of cancer-related death worldwide in 2018 [1]. The liver closely cross-talks with the gut and performs necessary functions related to the metabolism of nutrients, immunity, and biotransformation of bacterial metabolites, and this communication is called the gut-liver axis [2]. A complete gut-liver axis relies on an intact intestinal barrier, healthy gut microbiota, and normal liver function. The gut microbiota is known as the most important microecological system in symbiosis with the human body; moreover, several clinical characteristics (age, body mass index, dietary habits, and physical exercise) were found to differentially affect the gut microbiome of healthy Chinese people [3]. Liver diseases, such as nonalcoholic fatty liver disease (NAFLD) and liver cirrhosis, are often associated with altered gut microbiota, and it has been suggested that gut bacterial products contribute to liver carcinogenesis [4–7]. Recent studies in animal models or in patients with a background of NAFLD have indicated that some specific gut microbes promote HCC tumorigenesis through the gut-liver axis [8–10]. Our previous study has already suggested gut microbes to be non-invasive biomarkers for early and advanced HCC in Northwest, Central, and East China [11]. However, whether gut microbial characteristics from a large cohort of HCC patients can be used to evaluate clinical prognosis has not yet been reported, and how gut microbiota influence the transcriptome profiles of HCC remains unclear.

To explore the connections of host-microbe in HBV-related HCC, we acquired paired data on liver transcriptome and gut microbiome from patients in East China to assess whether elevated or depleted transcripts of host liver are associated with specific gut microbes (Additional file 1: Fig. S1). While it is known that a lower tumor burden always implies a better clinical outcome, the associations between gut microbiota and tumor burden in HCC patients are less well-understood. Therefore, by referring to Stage A4 of the BCLC staging classification, we divided 113 patients into small HCC and non-small HCC groups according to tumor burden [12]. More specifically, in this study, small HCC is defined as “one single tumor nodule with a maximum diameter not more than 3 cm; or no more than three tumor nodules with the sum of the maximum diameters not being more than 3 cm”. In addition, data on 5-year survival and 2-year disease-free survival were acquired to construct a random forest model and support vector machine model to further indicate the predictive effect of gut microbiota on tumor progression and recurrence/metastasis. By the simultaneous integrated analysis of the liver tumor transcriptome, gut microbiome, and clinical characteristics, we gained better insights into the nature of the gut-metabolite-liver axis in HBV-related HCC patients.

## Methods

### Participant information

This research was designed following the prospective specimen collection and retrospective blinded evaluation principle (PRoBE) [13]. In our previous study, a total of 281 qualified stool samples from participants (131 healthy controls and 150 HCC patients) from First Affiliated Hospital, School of Medicine, Zhejiang University were prospectively collected from November 2013 to July 2014 and subjected to 16S rRNA MiSeq sequencing; meanwhile, peripheral blood samples were acquired for routine examinations of blood, liver biochemistry, and tumor markers [11]. In September 2019, we revisited these 281 participants, and after excluding missing information and liver cancer R1 &R2 resection, 100 healthy controls and 113 HCC patients were finally enrolled. Among the HCC patients, paired tumor and adjacent non-tumor liver tissues from 32 patients (Additional file 2: Table S1: zfh001-zfh034) were finally collected based on stringent criteria. The exclusion criteria were as follows: (a) more than 7 days from stool collection to liver cancer resection; (b) the use of drugs that affect liver metabolism and function before resection, such as adenosyl methionine, ursodeoxycholic acid, polyene phosphatidylcholine, glutathione, fructose diphosphate, vitamins, and Chinese materia medica; (c) for multiple HCC tumors, a variety of types of tissue differentiation in pathological diagnosis; and (d) unqualified tumor or non-tumor specimens. The detailed criteria are listed in Additional file 3: Supplementary Methods.

Overall survival (OS) was defined as the interval between surgery and death. Disease-free survival (DFS) was defined as the interval between liver resection and the first recurrence/metastasis. OS and DFS were collected from a follow-up telephone interview or electronic medical records that were reexamined by two independent follow-up staff unrelated to this study.

### 16S rRNA MiSeq sequencing and operational taxonomy unit (OTU) clustering

High-throughput V3-V5 16S rRNA sequencing was performed on an Illumina MiSeq platform. The raw data of Illumina reads from 281 fecal samples were uploaded to the ENA-EMBL-EBI database under PRJEB8708 [11]. Fecal sample collection, DNA extraction, stool moisture measurement, 16S rRNA MiSeq sequencing, and OTU annotations are shown in Additional file 2: Table S2 and Additional file 3: Supplementary Methods.

### Bacterial diversity and taxonomic analysis

For alpha diversity, the Shannon, Simpson, and Invsimpson indexes were calculated by the R program package “vegan” [14]. The data are listed in Additional file 2: Table S3. Nonmetric multidimensional scaling (NMDS) was conducted by “vegan” to display bacterial beta diversity using Manhattan’s method [14]. Linear discriminant analysis effect size (LEfSe) was applied to evaluate the differentially abundant taxa [15].

### RNA extraction, RNA-seq, and data analysis

Frozen liver tissues were used for RNA extraction, and DNA libraries were sequenced by Illumina HiSeq 2500 (pair-end 150-nucleotide read length). For the paired tumor and adjacent non-tumor liver tissues from the 32 patients, an average of 47.92 M and 45.53 M high-quality reads were generated by RNA-seq (Additional file 1: Fig. S2A). Clean data were deposited in the NCBI Gene Expression Omnibus (GSE138485/PRJNA576155). HISAT2 was used to align the sequencing reads to the human reference sequence (UCSC/hg38.p12) [16]. The featureCounts function was performed for each gene count from trimmed reads against the GENCODE (release 30) transcript models [17]. For the data from the paired liver tissue samples of the 32 patients, gene expression levels were quantitated by edgeR (Additional file 1: Fig. S2B) [18]. Differential genes were defined as its adjusted *p* value < 0.05 and |log_2_FC| > 0.8.

For single paired tumor and adjacent non-tumor liver tissue samples from each patient, a total of 32 datasets of transcriptome profiles were calculated by GFOLD [19]. Differentially expressed genes were defined as |gfold| > 0.8 and |log_2_FC| > 0.8. When calculating the correlation between differential gene expression and OTU abundance, the |log_2_FC| values of these genes that did not satisfy the above conditions were forcibly defined as zero. The details are provided in Additional file 3: Supplementary Methods.

### Correlation between OTUs and differentially expressed genes

The Pearson correlation coefficient was calculated to measure the connections between OTU abundance and differential gene expression level for each OTU-gene pair across all 32 patients. To reduce the computational load and avoid contingency, OTUs that were present in < 10% of the 32 patients were excluded, leaving 310 OTUs that constituted 95.85% of the initial abundance (Additional file 2: Table S4). A total of 1,820,940 OTU-gene pairs were calculated. The significance of each OTU-gene pair was determined based on adjusted *P* value < 0.05. Independent Student’s *t* test was applied to evaluate the difference in log_2_FC values calculated by GFOLD between the small HCC and non-small HCC groups. The detailed scripts of the correlation calculations are provided in Additional file 3: Supplementary Methods.

### Microbial biomarker identification and prediction model construction for clinical prognosis

Based on all 113 HCC patients, random forest and support vector machine with fivefold cross-validation were executed to build prediction models of 5-year survival and 2-year disease-free survival, as implemented in the python package “scikit-learn” (version 0.21.3). The detailed scripts for OTU-marker identification and model construction are described in Additional file 3: Supplementary Methods.

### Statistical analysis

Continuous variables following a normal distribution were compared by Student’s *t* test; otherwise, the Wilcoxon rank sum test was used. Fisher’s exact test was used to compare categorical variables in a 2 × 2 table. All statistical tests were two-sided. The *P* values were adjusted by the Benjamini-Hochberg correction. Variables associated with survival rate were identified by the Cox proportional hazards regression model. Statistical analyses were performed using Python and SPSS V23.0 for Mac (SPSS Inc., USA).

## Results

### Summary of clinical characteristics

**Fig. 1:**
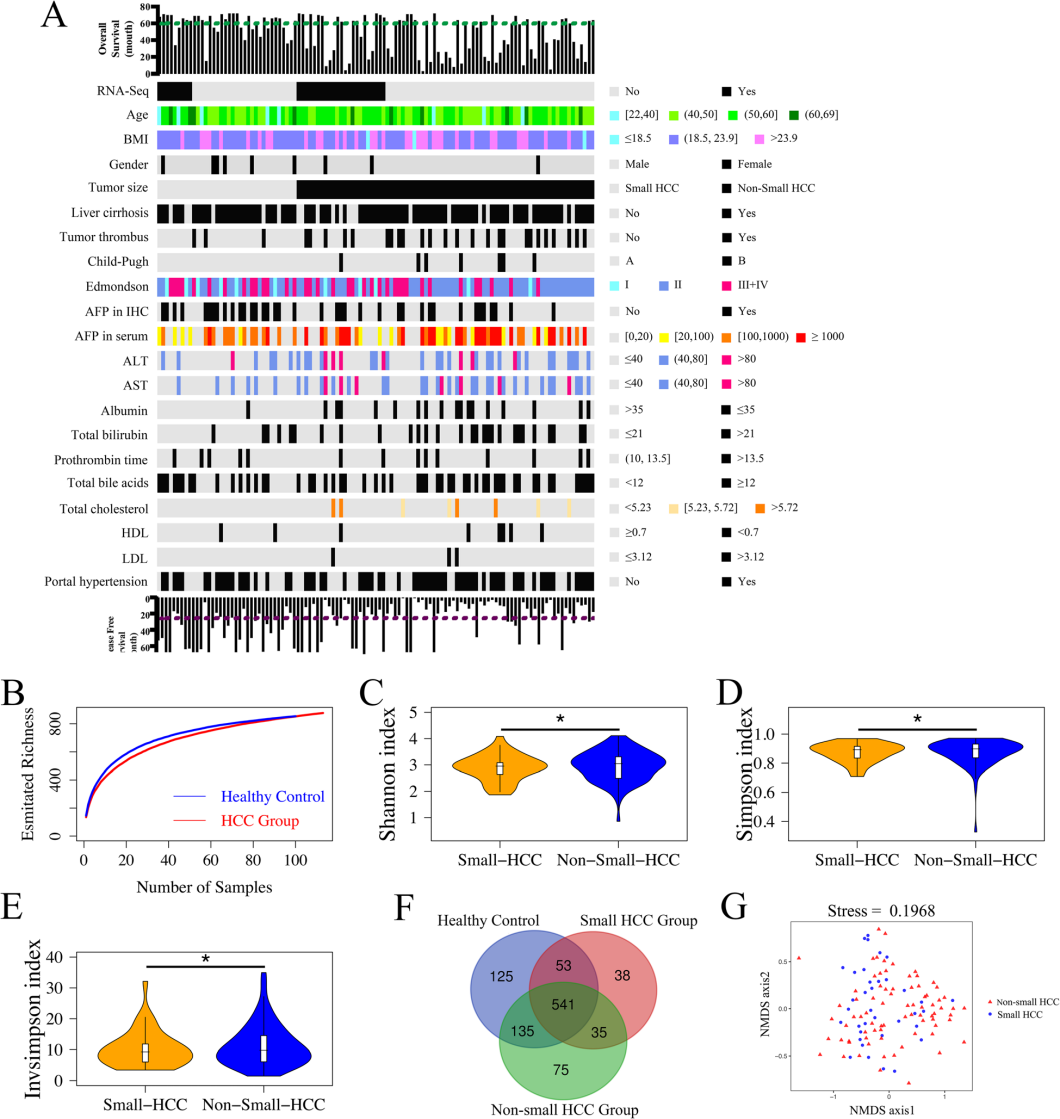
Clinicopathological features and gut microbial diversity of all patients. **a** Clinicopathological features and clinical outcomes of all 113 HCC patients. The green dotted line represents 5-year survival; the purple dotted line represents 2-year disease-free survival. **b** Shannon-Wiener curves between numbers of fecal samples and estimated richness. Compared with small HCC, fecal microbial diversities, as estimated by the Shannon index (**c**), Simpson index (**d**), and Invsimpson index (**e**), were significantly increased in patients with non-small HCC (*p* = 0.048, 0.027, and 0.027, respectively; *p < 0.05, Wilcoxon rank sum test). **f** A Venn diagram illustrates that 541 of the total richness of 1002 OTUs were shared among three groups, while 576 out of 877 OTUs were shared between the small HCC and non-small HCC subgroups. **g** Beta diversity was evaluated using NMDS by Bray-Curtis. Boxplot “boxes” indicate the first, second, and third quartiles of the data

All 113 HCC patients with HBV infection and 100 healthy volunteers were Han Chinese individuals from East China and practiced comparable dietary habits (mixed diet) to exclude dietary differences [20]. The clinicopathological characteristics (Fig. 1a, Additional file 2: Table S1, S5, S6) of these groups [healthy controls versus HCC group, small HCC subgroup (*n* = 36) versus non-small HCC (*n* = 77) subgroup, non-cirrhotic HCC subgroup (*n* = 22) versus cirrhotic HCC subgroup (*n* = 91)] were generally matched, including age, sex, BMI, tumor differentiation, and Child-Pugh score, suggesting that there were no established confounding factors affecting group discrimination before sample collection. Serum alpha-fetoprotein (AFP) levels were significantly higher in patients with HCC than in healthy controls, but the AFP levels could not distinguish tumor size or cirrhosis in HCC patients.

### Increased gut microbial diversity in non-small HCC

Following taxonomic assignment, a total of 7,934,068 qualified sequences (median = 31,679) and 1296 OTUs were obtained (Additional file 2: Table S2 and S3). Rarefaction analysis revealed that the estimated richness of OTUs almost reached saturation in healthy controls (*n* = 100) and HCC group (*n* = 113) (Fig. 1b). Compared with their own counterparts, the fecal microbial diversities of species in each sample were significantly increased in non-small HCC (Fig. 1c–e); however, there was no difference between healthy volunteers and HCC patients. A Venn diagram illustrated that 541 the total richness of 1002 OTUs (the abundances of 294 OTUs among all participants were all zero) were shared among the three groups, while 576 out of 877 OTUs were shared between the small HCC and non-small HCC subgroups (Fig. 1f). Notably, 75 out of 1002 OTUs were unique to the non-small HCC group. For beta diversity, NMDS plots evaluated by Bray-Curtis distances revealed a symmetrical distribution of gut microbiota among all pairs of samples (Fig. 1g, Additional file 1: Fig. S3).

### Phylogenetic profiles of fecal microbial communities in non-small HCC patients

**Fig. 2:**
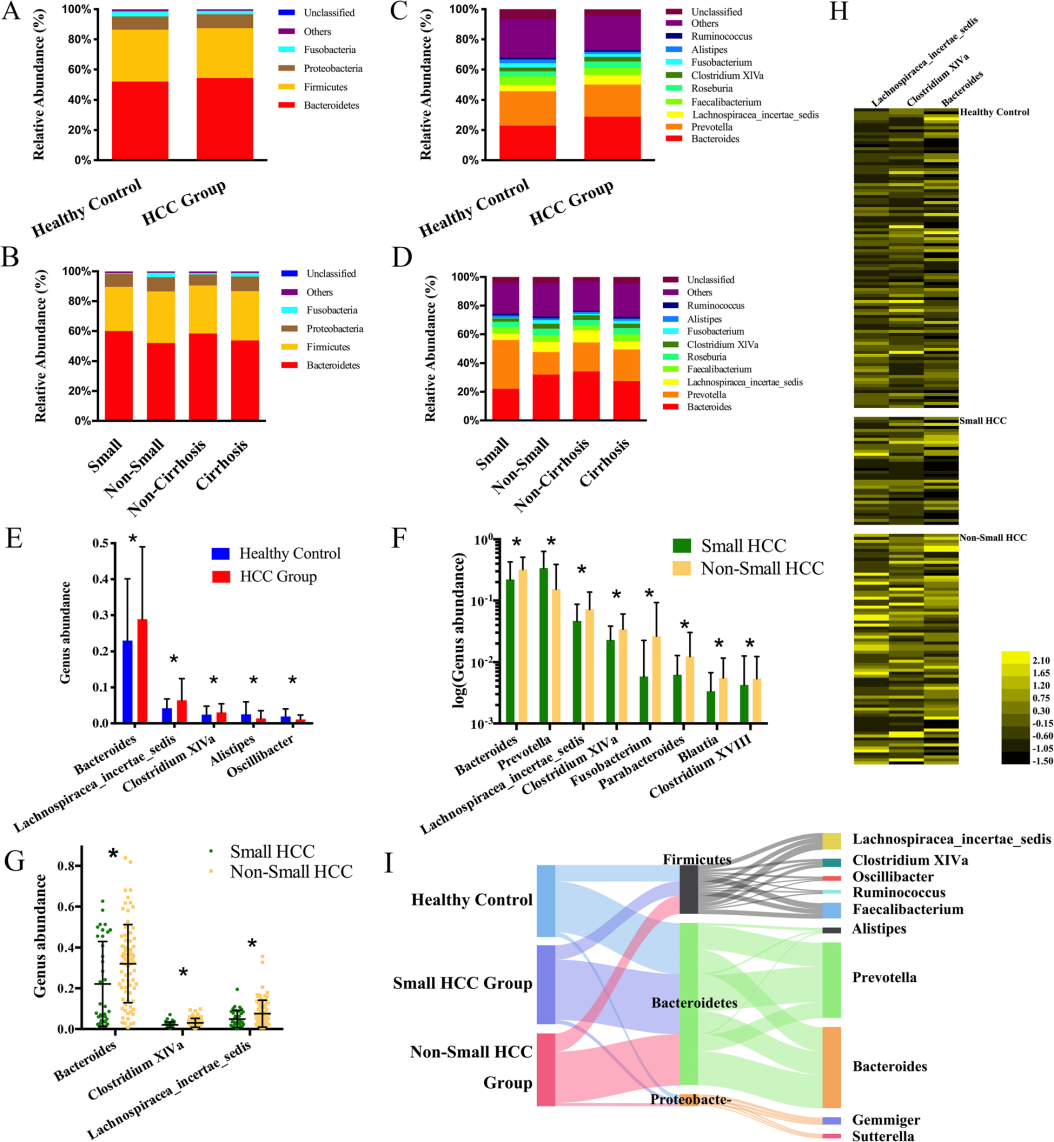
Phylogenetic profiles of gut microbes among healthy controls and all patients. Compositions of bacterial community at the phylum level between healthy controls and HCC patients (**a**), and patients from subgroups of HCC (**b**). Compositions of bacterial community (top 10) at the genus level between healthy controls and HCC patients (**c**), and patients from subgroups of HCC (**d**). **e** The differential microbial community at the genus level in HCC patients versus healthy controls. **f**, **g** The differential microbial community at the genus level in patients with small HCC versus non-small HCC. Levels of significance: *p < 0.05 (Wilcoxon rank sum test). **h** The distributions of *Bacteroides*, *Lachnospiracea incertae sedis*, and *Clostridium XIVa* normalized by a *Z*-score among healthy controls and patients with small HCC and non-small HCC. **i** Sankey analysis of healthy controls and patients with small HCC and non-small HCC. Error bars are presented as the SD

The three predominant bacterial phyla in each group were *Bacteroidetes*, *Firmicutes*, and *Proteobacteria*, altogether constituting up to 90% of the OTUs on average (Fig. 2a, b). The average compositions of the bacterial community (top 10) at the genus level are shown in Fig. 2c and Fig. 2d. To compare the differences in the fecal microbial communities between each group, Wilcoxon rank sum test was performed at the phylum and genus levels (Additional file 2: Table S7 and S8). Compared with healthy controls, *Bacteroides, Lachnospiracea incertae sedis*, and *Clostridium XIVa* were enriched in the HCC group (all *p* < 0.05, Fig. 2e). When further analyzing stratified additional clinical characteristics in the HCC group, *Bacteroides* (*p* = 0.0152), *Lachnospiracea incertae sedis* (*p* = 0.0249), *Clostridium XIVa* (*p* = 0.0168), and *Parabacteroides* (*p* = 0.006) were significantly enriched in non-small HCC subgroup compared with small HCC subgroup (Fig. 2f, g); however, these three genera showed no difference in patients with cirrhotic HCC, portal hypertension, or low-albumin levels (Additional file 1: Fig. S4).

To discover high-dimensional biomarkers, LEfSe was applied to identify predominant bacterial taxa associated with different clinical characteristics. *Bacteroides*, *Lachnospiracea incertae sedis*, and *Clostridium XIVa* were significantly overrepresented [log_10_(LDA score) > 3] in the feces of patients in non-small HCC subgroup (Additional file 1: Fig. S5). The relative abundances of these three genera were further subjected to clustering analysis, which indicated that these three genera were abundant in non-small HCC subgroup (Fig. 2h). Sankey diagrams showed the major proportion of taxa (phylum and genus) among healthy controls and small HCC and non-small HCC patients. In participants with a high tumor burden, the proportion of these three genera gradually increased and became predominant, accompanied by the changes of other bacteria (Fig. 2i).

These data suggested that *Bacteroides*, *Lachnospiracea incertae sedis*, and *Clostridium XIVa* were sufficient to differentiate the microbiota of patients with small HCC from that of patients with non-small HCC. The interrelationship between 16S OTU genus clusters (*Bacteroides*, *Lachnospiracea incertae sedis*, *Clostridium XIVa*) and taxonomic compositions (NCBI Taxonomy ID) is listed in Additional file 2: Table S9.

### Global overview of liver tumor transcriptome in HCC patients

**Fig. 3:**
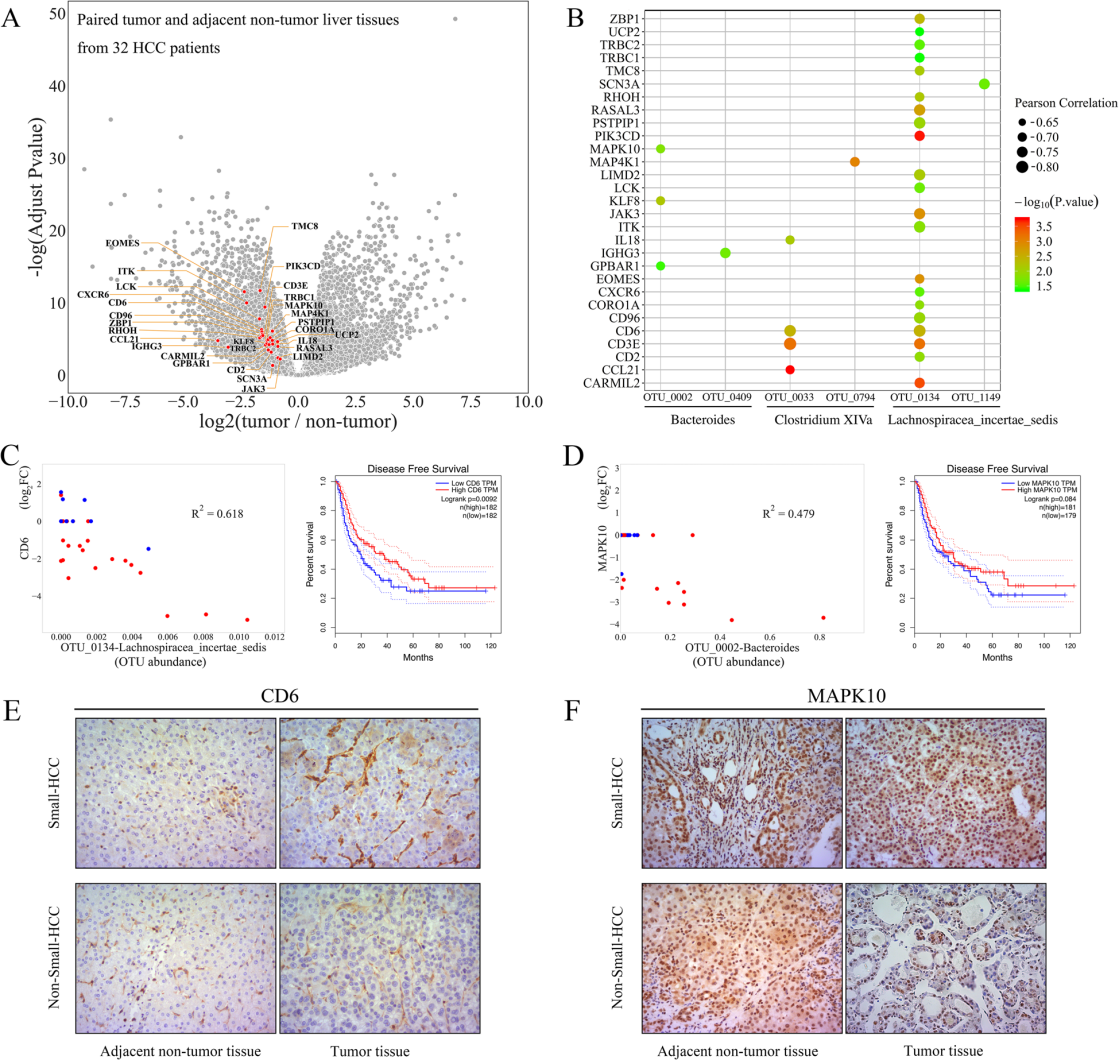
The associations between host liver gene expression and gut microbes in patients with HCC. **a** Differential expression of the microbe-associated genes from 32 paired tumor and adjacent non-tumor liver tissue samples. **b** Pearson correlation coefficients of 31 OTU-gene pairs, and *P* values evaluated by Student’s *t* test for comparing the difference in log_2_FC values calculated by GFOLD between small HCC and non-small HCC subgroups. Scatter diagrams and Cox hazards models of two typical OTU-gene pairs, OTU_0134-CD6 (**c**) and OTU_0002-MAPK10 (**d**). The *x*-axis of the scatter diagram indicates the OTU abundance. The *y*-axis indicates log_2_FC of gene expression calculated by GFOLD. Each point represents a patient (red: non-small HCC; blue: small HCC), and some points coincided at the origin. Immunohistochemical staining showed that CD6 (**e**) and MAPK10 (**f**) were highly expressed in tumor tissues of patients with small HCC versus non-small HCC (× 200)

Since HCC patients demonstrated significant associations of microbiota with clinical characteristics (tumor burden), we hypothesized that changes in the liver tumorigenesis transcriptome may be correlated with gut microbiota. Among 113 patients, paired tumor and adjacent non-tumor liver tissues from 32 HCC patients were finally collected based on stringent criteria for NGS RNA-seq. Of these 32 patients, 23 had non-small HCC. According to our definition, we identified a total of 8101 differentially expressed genes among 32 paired tumor and adjacent non-tumor liver tissues by edgeR (Fig. 3a). Furthermore, due to tumor heterogeneity and no biological duplication for a single case, we adopted GFOLD to further filter the candidate genes, in which a non-tumor specimen was used to normalize the expression of tumor specimen for each patient. Furthermore, the genes whose log_2_FC values calculated by GFOLD were zero in > 90% of patients were excluded, leaving 5874 genes for further investigation of their correlations with gut microbes (310 OTUs). To evaluate the predictive values of the candidate genes for OS or DFS, GEPIA was used as an independent diagnostic tool to compensate for the bias caused by the small sample sizes used in this study [21].

### Host liver transcriptome profiles influenced by gut microbiota via tumor immune microenvironment

Based on the above-described transcriptome and fecal microbiota data, Pearson’s correlation-based analysis was performed to discover microbe-associated genes and to test whether host transcriptional profiles in HCC patients could be partially influenced by gut microbiota (Additional file 2: Table S4). A total of 53 genes and 11 OTUs were identified, forming 56 OTU-gene pairs (Additional file 2: Table S10). The small and non-small HCC subgroups were clearly distinguished by the log_2_FC values of these 53 differential genes (all *p* < 0.05). Based on preliminary gene annotation and pathway analysis, 29 differentially expressed genes were identified as negatively correlated with 6 OTUs (members of *Bacteroides*, *Lachnospiracea incertae sedis*, and *Clostridium XIVa*), forming 31 OTU-gene pairs (median *r* = − 0.73, Fig. 3b, Additional file 1: Fig. S6). Figure 3 c and d show two typical OTU-gene pairs (OTU_0134-CD6 and OTU_0002-MAPK10). In 32 patients, the increased abundance of OTU_0134 was accompanied by a decrease in CD6 expression in HCC tissues, and it was clear that this type of downregulation was particularly pronounced in patients with non-small HCC. Small HCC usually implies a better clinical outcome, and Cox hazards models based on GEPIA indicated that CD6 and MAPK10 were tumor suppressors associated with a good clinical prognosis, which was consistent with the results of our study. Different expression levels at the protein level between the two groups also showed the same results as transcriptomics (Fig. 3e, f). To further validate the patterns of these 29 genes under different tumor burdens, edgeR was used to calculate their own values of log_2_FC and adjusted *P* values in 9 small HCC and 23 non-small HCC patients, respectively (Additional file 1: Fig. S7). The results showed that these 29 genes satisfied the definition of differential genes in the non-small HCC subgroup but not the small HCC subgroup.

**Fig. 4:**
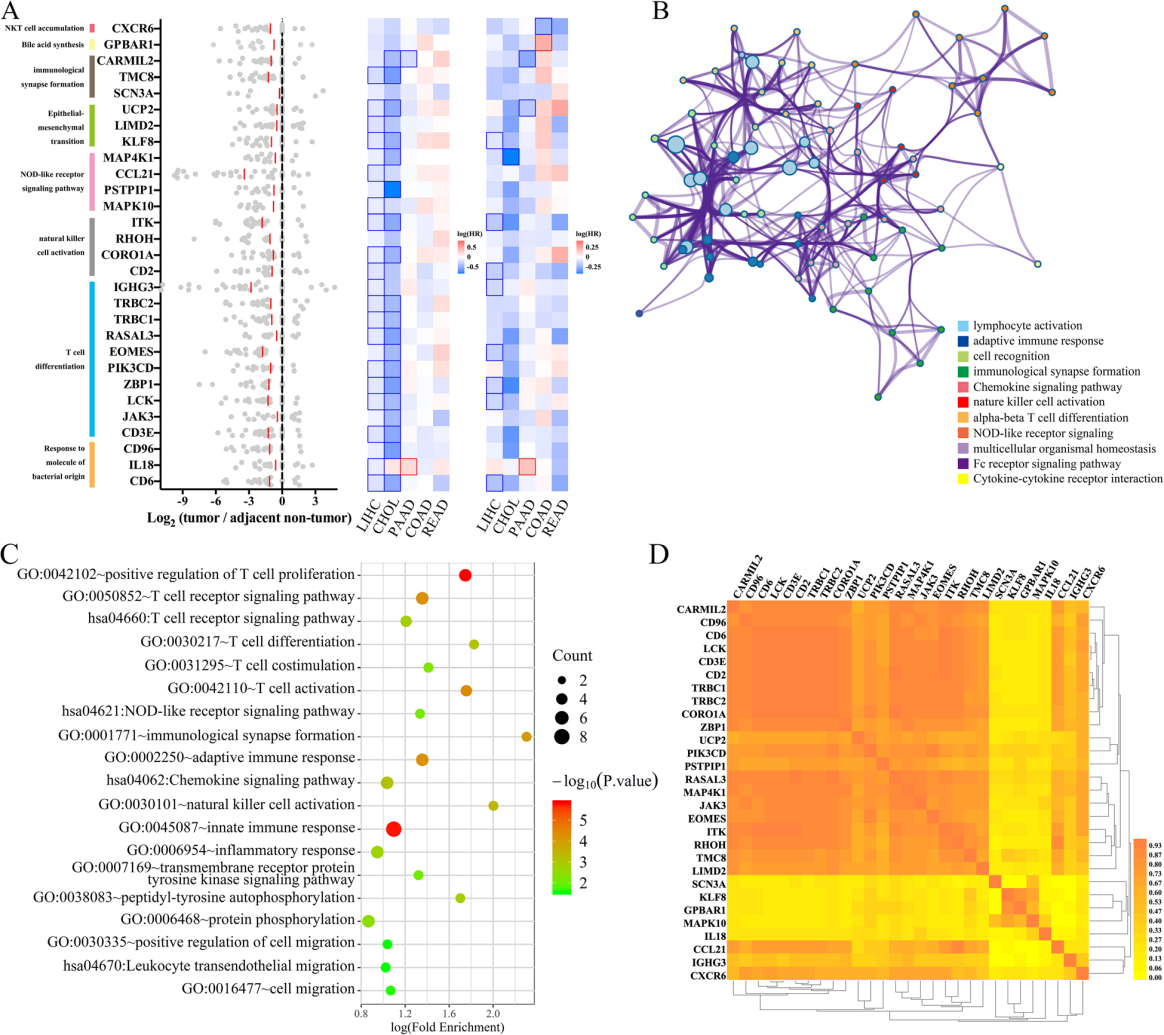
Functional annotations and pathway analysis of the microbe-associated genes. **a** Integrated analysis of key microbe-associated genes: functionalannotation and log_2_FC value of gene from each patient (*n* = 32, left panel), DFS (based on GEPIA, middle panel), and OS (based on GEPIA, right panel). The red line denotes the average values. Heatmaps represent the log_10_ (hazard ratio) value of each gene in each kind of tumor. The dark square box denotes that the clinical prognosis was statistically significant. **b** The GO enrichment analysis based on Metascape. **c** Pathway analysis based on DAVID 6.8. **d** Pairwise gene expression correlation analysis using the Pearson method shows interdependent relationships and gene co-expression according to GEPIA. LIHC, Hepatocellular carcinoma; CHOL, Cholangial carcinoma; PAAD, Pancreatic adenocarcinoma; COAD, Colon adenocarcinoma; READ, Rectum adenocarcinoma

Figure 4a describes in detail the major functional annotations of these 29 genes and the differential expression levels of each patient. According to the GEPIA dataset, 100% of the differentially expressed genes (29/29) had a low hazard ratio in HCC tissues, and 69% of the differentially expressed genes (20/29) predicted good clinical prognosis (Additional file 1: Fig. S8). Based on the GEPIA dataset, we also compared the performance of these 29 genes in cholangial carcinoma, pancreatic adenocarcinoma, colon adenocarcinoma, and rectum adenocarcinoma. Of note, their performance was consistent between HCC and cholangial carcinoma, but these genes were not associated with the prognoses of pancreatic, colon, and rectum adenocarcinoma, which indicated that the functions of these microbe-associated genes were specific in liver carcinoma. Pathway analysis revealed that these 29 genes converged on immune-related pathways (T cell receptor signaling, positive regulation of T cell proliferation, natural killer cell activation and NOD-like receptor signaling, response to molecule of bacterial origin, etc.), which are components of tumor immune microenvironment (Fig. 4b, c). GPBAR1, a member of the G protein-coupled bile acid receptor family, functions in the tumorigenesis of hepatocytes and mesenchymal immune cells [22]. CXCR6 is considered to be an important marker of NKT cells [9]. Pearson analysis was also performed to explore the correlations between these 29 genes and acute liver inflammatory factors (ALT and AST), and the results indicated that high ALT and AST levels likely do not drive an inflammatory transcriptome profile in this case (mean *R*^2^ = 0.045 and 0.023, Additional file 1: Fig. S9).

To elucidate the localization and roles of these genes in tumor immune microenvironment, we evaluated their expressions in an independent single-cell map database (based on SMART-seq2) of HCC [23]. Twenty-two genes were highly expressed in CD4^+^ T cells, 23 genes were highly expressed in CD8^+^ T cells, 23 genes were highly expressed in NK cells, 18 genes were highly expressed in macrophages, and 17 genes were highly expressed in B cells (Additional file 1: Fig. S10). Interestingly, these genes also had an interdependent relationship according to GEPIA, suggesting that gut microbiota may influence the transcriptome of HCC through a common factor (Fig. 4d).

### Serum bile acids: important communication mediators between gut microbiota and host transcriptome of HCC

**Fig. 5:**
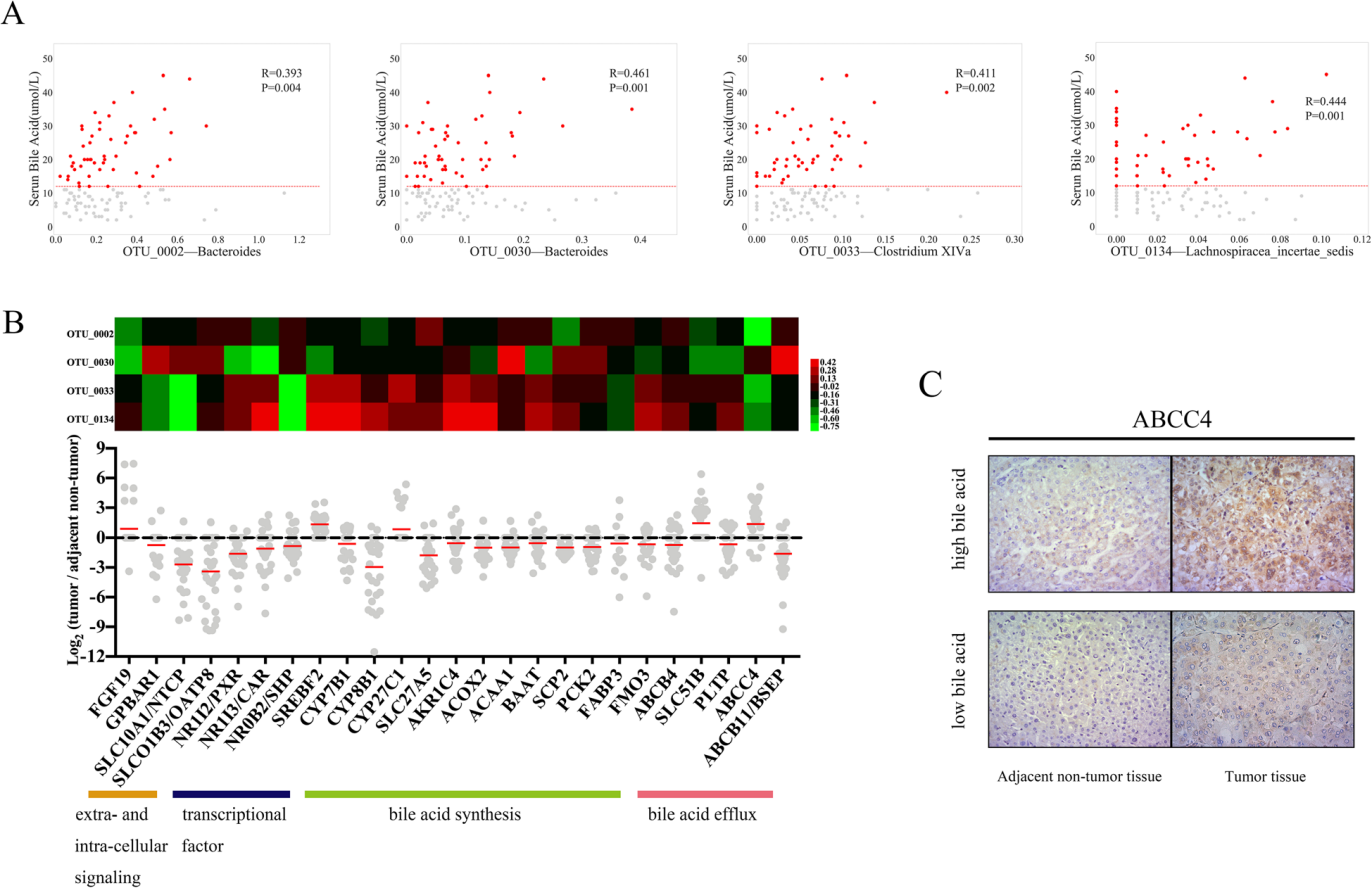
Associations among liver transcriptome, serum bile acid, and gut microbiota. **a** Scatter diagrams of OTU abundance (*x*-axis, square root, and arcsin transformation) and serum bile acids (*y*-axis). The red horizontal line denotes 12 μmol/L of serum bile acid (*n* = 52). **b** Heatmap representing the Pearson correlation coefficients of 100 OTU-gene pairs (upper panel); functional annotation and log_2_FC of genes related to bile acid metabolism for each patient (lower panel). The red line denotes the average values of log_2_FC. **c** Immunohistochemical staining showed that ABCC4 was highly expressed in tumor and adjacent non-tumor liver tissues of patient with high level of bile acid (× 200)

For intra-individual correlation analyses, we focused on associations between clinical characteristics (such as all values or abnormal values of BMI, AST, and triglyceride) and gut microbiota (75 OTUs matched to *Bacteroides*, *Lachnospiracea incertae sedis*, and *Clostridium XIVa*). Pearson’s correlation-based analysis revealed four bile acid-associated OTUs, while no positive and meaningful result was found from the other clinical characteristics (Fig. 5a, Additional file 2: Table S11 and S12). Among these four OTUs, OTU_0002, OTU_0033, and OTU_0134 were found to be gut microbes that were associated with the abovementioned transcriptome changes in tumor immune microenvironment. Using the Virtual Metabolic Human database, we identified the metabolite distributions of *Bacteroides*, *Lachnospiracea incertae sedis*, and *Clostridium XIVa*, which suggested that most of them metabolized cholic acid, chenodeoxycholic acid, 3-dehydrocholic acid, 7-dehydrochenodeoxycholic acid, taurocholic acid, etc. (Additional file 2: Table S13) [24, 25].

Further, based on 32 pairs of liver tissue samples, 25 genes related to these four OTUs were also identified (Fig. 5b), and these genes affected liver bile acid metabolism (extra- and intra-cellular signaling, transcriptional factor, and bile acid synthesis and efflux). Immunohistochemistry confirmed that ATP binding cassette subfamily C member 4 (ABCC4) proteins were highly expressed in both tumor and adjacent non-tumor liver tissues of patients with high levels of bile acid (Fig. 5c). These results suggest that bile acids may be important mediators for the communication between gut microbiota and host transcriptome of HCC.

### Identification of microbial-based markers for clinical prognosis

**Fig. 6:**
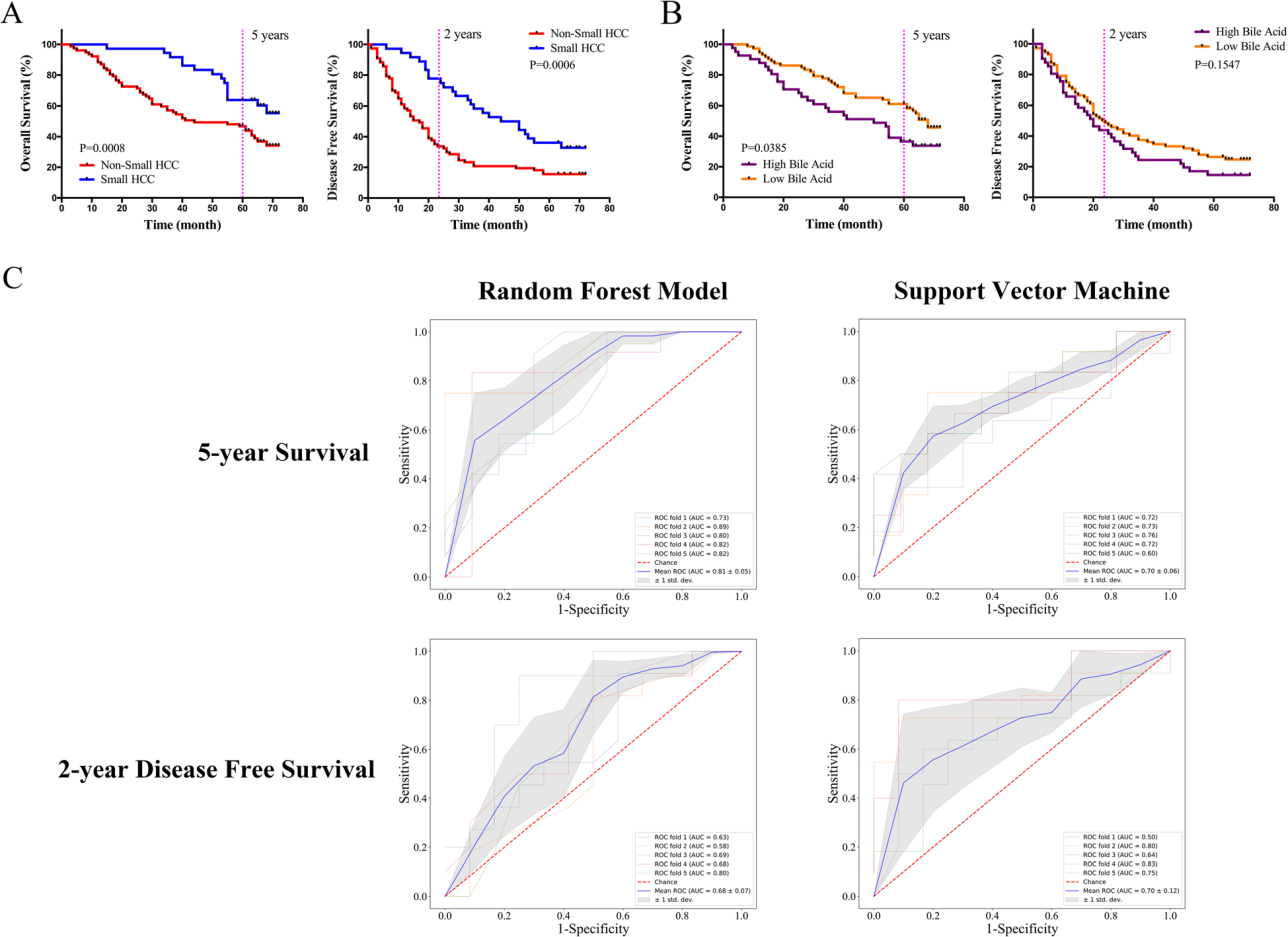
Identification of microbial-based markers for clinical prognosis by machine learning models. Kaplan-Meier curves for 5-year survival and 2-year disease-free survival based on tumor burden (**a**) and serum bile acids (**b**). **c** ROC curves for classifiers designed to predict clinical prognosis (left panel: RF; right panel: SVM). Plots showed the true positive rate (*y*-axis) versus the false positive rate (*x*-axis). AUC scores (including 95% confidence interval) of ROC curves with fivefold cross-validations are listed on the right for classification accuracy

Among 113 patients, high tumor burden (non-small HCC) and higher bile acid levels (≥ 16 μmol/L, *n* = 41) indicated worse clinical outcomes (Fig. 6a, b). We assessed the power of gut microbiota to predict clinical prognosis by assessing the following OTUs: OTU_0002, OTU_0033, and OTU_0134 (associated with both tumor immune microenvironment and bile acid metabolism), OTU_0794 (associated with tumor immune microenvironment), and OTU_0030 (associated with bile acid metabolism). Random forest and support vector machine classifier models with five repeats of 5-fold cross-validation were constructed to predict clinical prognosis of the 113 patients (Fig. 6c). These OTUs had good classification performance for discriminating 5-year survival (81% ± 5% and 70% ± 6%, respectively, in RF and SVM). In addition, the AUC values reached 68% ± 7% (RF) and 70% ± 12% (SVM) for 2-year disease-free survival. The abundances and corresponding bacterial genera of these 6 OTU markers in each patient are listed in Additional file 2: Table S14 and S15. The data indicated that these OTU markers related to tumor immune microenvironment and bile acid metabolism in HCC have the potential to predict clinical prognosis.

## Discussion

Although our study and many other studies in humans have reported that the gut community is highly differential and variable between any two individuals, the transcriptome of HCC appears to be a strong correlate of this variation in the composition of gut microbiome [26, 27]. In this study, we provided evidence that the tumor burden of HCC is associated with the presence of specific gut microbes, distinguished by the enrichment of *Bacteroides*, *Lachnospiracea incertae sedis*, and *Clostridium XIVa* in patients with non-small HCC [28]. We observed that patients with these three genera mounted a weaker host liver anti-tumor inflammatory response, which was confirmed by an independent database of single-cell sequencing and pathway enrichment analysis. A wide range of immune cells, such as T cells, NK cells, and monocytes, are well-known linchpins in the process of hepatocarcinogenesis [23]. Tregs, especially CD4^+^FOXP3^+^ regulatory T cells, in mouse models of colitis and allergic diarrhea can be induced by *Clostridium XIVa* from human microbiota [29]. In our study, inter-individual differences in gut microbiota correlated with corresponding changes in microbe-associated gene expressions in tumor immune microenvironment, which is consistent with the findings of previous reports. For example, a low level of CCL21 expressed in LAMP3^+^ DCs hinders CCR7^+^ cells, which facilitates tumor growth and indicates an unfavorable prognosis for HCC patients [30]. CD160^+^ NK cells had high expression of the transcription factor EOMES, which is expressed at low levels in patients with non-small HCC [31]. PIK3CD, MAP4K1, and MAPK10, which are key members of the LPS-stimulated MAPK pathway, are highly expressed in patients with high tumor burden. Previous reports in animals are consistent with our findings, in which *Clostridium XIVa* influences bile acid-controlled NKT cell accumulation. *Bacteroides* activate GPBAR1 and uncoupling protein 2 (UCP2) signaling to improve liver metabolism, indicating that metabolites may be important mediators for communication between gut microbiota and host transcriptome of HCC [9, 32, 33].

Cholic acid, deoxycholic acid, chenodeoxycholic acid, and lithocholic acid, as metabolites of *Bacteroides*, *Lachnospiracea incertae sedis*, and *Clostridium XIVa* in the gut, have been shown to promote liver carcinogenesis [24, 34–37]. Our data also suggested that compared to lower bile acid levels, higher bile acid levels (≥ 16 μmol/L) indicated worse clinical outcomes among patients with enrichment of *Bacteroides*, *Lachnospiracea incertae sedis*, and *Clostridium XIVa* (OTU2, OTU30, OTU33, and OTU134). As reported, sterol carrier protein 2 (SCP2) and fibroblast growth factor 19 (FGF19) regulated bile acid secretion, yet phospholipid transfer protein (PLTP) increased fecal bile acid excretion in mice [38–40]. These gut microbes and genes may be important targets for maintaining the homeostasis of bile acid metabolism in the gut-liver axis. As serum bile acids are a kind of mixture, it is essential to differentiate the chemical components for further analysis, but this is difficult to perform in a retrospective study.

In recent years, principal component analysis and k-means have been used to analyze multi-omics data. The strongest microbe-gene correlation coefficients were approximately 0.7 to 0.8 in our study, and the significant associations of many other microbe-gene pairs would not survive correction for multiple hypothesis testing (FDR) when all genes and OTUs were simultaneously analyzed. If the primary goal is to determine as many associations as possible among multiple variables, dimensionality reduction is a key workflow. Nevertheless, it must be noted that our data were evaluated against a stringent screening criterion to highlight the most significant microbes and genes, which facilitated the construction and improved the accuracy of the subsequent prognostic model. However, this did not mean that there were only a few dozen of liver tumor transcripts influenced by gut microbiota.

In our study, we identified six important OTUs that were thought to be strongly associated with bile acid metabolism and tumor burden due to changes in tumor immune microenvironment. The RF and SVM models based on these OTU markers had good prediction potential for clinical outcomes of HCC patients. Importantly, this suggests that under the premise of strict control of the treatment plan (R0 resection, but excluding acute and chronic complications), gut microbiota is a vital influencing factor for clinical prognosis of HCC patients. The predictive efficacy of the model is much better for 5-year survival than 2-year disease-free survival, suggesting that the tumorigenesis caused by gut microbiota has a long-lasting and processive association with an individual’s dietary habits, lifestyle, and even mental stress.

The strengths of our study include the large cohort of patients with clinical outcomes, whose clinical characteristics, liver tumor transcriptome, and gut microbiota data were obtained and comprehensively analyzed at the level of host gene expression, metabolites, and gut microbes. Moreover, due to the difficulty in obtaining recurrent/metastatic tumor samples and the routine applications of antibiotics after liver cancer resection, we could associate gut microbiota and host transcriptome with clinical characteristics at only one timepoint under stringent criteria.

## Conclusions

This study revealed that changes in tumor immune microenvironment correlated with gut microbiota via serum bile acids are possibly important factors associated with tumor burden and adverse clinical outcomes in HBV-related HCC. Gut microbes can be used as biomarkers of clinical features and outcomes, and microbe-associated transcripts of host tumors can partly explain how gut microbiota promotes HCC pathogenesis. Although our study has some limitations, it provides new insights into exploring the connections of gut microbiome-transcriptome for human biomarker discovery.

## Supplementary information

**Additional file 1:** **Fig. S1.** Study design and flow diagram. **Fig. S2.** RNA-seq and data analysis. (A) QC passed reads sequenced by an Illumina HiSeq 2500 (pair-end 150-nucleotide read length) for tumors and adjacent non-tumor liver tissues (*n* = 32, respectively, student’s t test). (B) Parameter of edgeR: plotBCV() and biological coefficient of variation (BCV) was 0.6114732. CPM, counts per million. **Fig. S3.** There are no differences in gut microbial diversity between healthy controls (*n* = 100) and HCC patients (*n* = 113). (A) Shannon-Wiener curves for all 213 fecal samples. Compared with healthy controls, fecal microbial diversities, as estimated by the Shannon index (B), Simpson index (C) and Invsimpson index (D), were no differences with HCC patients (all *p* > 0.05, Wilcoxon rank sum test). (E) A Venn diagram showed that 729 of the total 1002 OTUs were shared, notably, 148 of 1002 OTUs were unique for HCC. (F) Beta diversity was calculated using Bray-Curtis by NMDS. The red dot represents HCC Group, and the blue dot represents Healthy Control. HCC, hepatocellular carcinoma; OTU, Operational Taxonomy Unit; NMDS, Nonmetric multidimensional scaling. **Fig. S4.** No differences in gut microbes between patients with Cirrhotic HCC (*n* = 91) and Non-Cirrhotic HCC (*n* = 22), between patients with portal hypertension (*n* = 66) and without portal hypertension (*n* = 47), between patients with low-albumin (*n* = 16) and normal-albumin (*n* = 97). (A) The microbial community at genus level in patients with Cirrhotic HCC versus Non-Cirrhotic HCC. (B) The distributions of *Bacteroides*, *Lachnospiracea incertae sedis* and *Clostridium XIVa* normalized by Z- score among healthy controls, patients with Cirrhotic HCC and Non-Cirrhotic HCC. (C) No differences in *Bacteroides*, *Lachnospiracea incertae sedis* and *Clostridium XIVa* between patients with portal hypertension and without portal hypertension. (D) No differences in *Bacteroides*, *Lachnospiracea incertae sedis* and *Clostridium XIVa* between patients with low-albumin and normal-albumin. (Wilcoxon rank sum test) HCC, hepatocellular carcinoma. **Fig. S5.** The specific characterization of fecal microbiota to distinguish toxigenic types was analyzed by linear discriminant analysis (LDA) effect size (LEfSe) method. (A) LEfSe method identified the most differentially abundant taxons between healthy controls and HCC patients. The HCC-enriched taxa were indicated with a negative LDA score (red), and health-enriched taxa presented a positive score (green). *Bacteroides*, *Lachnospiracea incertae sedis* and *Clostridium XIVa* were significantly overrepresented in the feces of HCC patients. (B) LEfSe method identified the most differentially abundant taxons between Small HCC and Non-Small HCC. The Non-Small HCC-enriched taxa were indicated with a negative LDA score (red), and Small-HCC-enriched taxa presented a positive score (green). *Bacteroides*, *Lachnospiracea incertae sedis* and *Clostridium XIVa* were significantly overrepresented in the feces of patients in Non-Small HCC subgroup. log10(LDA score) = 3 as cut-off value. HCC, hepatocellular carcinoma. **Fig. S6.** Scatter diagrams for all 31 OTU-gene pairs. The x axis indicated the OTU abundance. The y axis indicated log2FC of gene expression calculated by GFOLD. Each point represented a patient (red: Non-Small HCC; blue: Small HCC) and some points coincided at the origin. **Fig. S7.** The expressions of 29 OTU-related genes in 23 Non-Small HCC (A) and 9 Small HCC patients (B, C). Parameter of edgeR: biological coefficient of variation (BCV) for 23 Non-Small HCC patients was 0.6090678 (D) and BCV for 9 Small HCC patients was 0.5872324 (E). **Fig. S8.** Overall survival and disease free survival for all 29 OTU-related genes based on GEPIA. Overall survival and disease free survival were identified by Log-rank test, a.k.a. the Mantel-Cox test (Cutoff-High and Cutoff-Low was both 50%). TRBC1 (official symbol) is named TRBV25–1 in GEPIA database. **Fig. S9.** Scatter diagrams for all 29 microbe-associated genes and levels of ALT/AST. The x axis indicated the levels of ALT/AST. The y axis indicated log2FC of gene expression calculated by GFOLD. Each point represented a patient (blue: ALT; red: AST). **Fig. S10.** Expressions of OTU-related genes for each cell type based on SMART-seq2 data (http://cancer-pku.cn:3838/HCC). Uniform Manifold Approximation and Projection (UMAP) plots showed 38 clusters identified by integrated analysis, colored by cell cluster (first plot). UMAP plots showed the cell distributions of tumor and adjacent liver from 6 patients (second plot). CARMIL2 was not found in this database. TRBC1 (official symbol) is named TRBV25–1 in this database. **Fig. S11.** The composition of stool color. Most of stool samples presented yellow, and showed no significant difference among the different cohorts. (A) The composition of stool color between healthy controls and HCC patients. (B) The composition of stool color in patients with Small HCC versus Non-Small HCC. HCC, hepatocellular carcinoma. **Fig. S12.** The abundance and distribution of stool moisture among the different cohorts by lyophilization assay on the frozen homogenized fecal material. Stool moisture content was determined in duplicate on the frozen homogenized fecal material (− 80 °C) as the percentage of stool mass loss after lyophilization. (A) The abundance of stool moisture between healthy controls and HCC patients. (B) The abundance of stool moisture in patients with Small HCC versus Non-Small HCC. (student’s t test) HCC, hepatocellular carcinoma.

**Additional file 2:** **Supplementary Table S1.** Clinical phenotype information of individuals with HCC. **Supplementary Table S2.** Taxon annotation of 1296 OTUs from all samples. **Supplementary Table S3.** Fecal microbial diversity index in all samples. **Supplementary Table S4.** three hundred and ten OTUs from all samples. **Supplementary Table S5.** Clinical phenotype information of healthy participants. **Supplementary Table S6.** Clinical characteristics summary of all enrolled individuals. **Supplementary Table S7.** Different degree of phylum level (*p* value) in Healthy Control and HCC Group. **Supplementary Table S8.** Different degree of genera level (p value) in Healthy Control and HCC Group. **Supplementary Table S9.** The interrelationship between 16S OTU clusters of at genus level and taxonomic compositions (NCBI Taxonomy ID). **Supplementary Table S10.** fifty-six OTU-gene pairs filtered by FDR test of Pearson correlation between OTUs and genes. **Supplementary Table S11.** Pearson correlation-based analysis of clinical characteristics (all values) and gut microbiota (75 OTUs matched to *Bacteroides* & *Lachnospiracea incertae sedis* & *Clostridium XIVa*). **Supplementary Table S12.** Pearson correlation-based analysis of clinical characteristics (abnormal values) and gut microbiota (75 OTUs matched to *Bacteroides* & *Lachnospiracea incertae sedis* & *Clostridium XIVa*). **Supplementary Table S13.** Metabolites of gut microbes according to Virtual Metabolic Human database (http://www.vmh.life). **Supplementary Table S14.** The relative abundance of six OUT-markers in each sample. **Supplementary Table S15.** The corresponding bacterial genera of six OTU-markers. **Supplementary Table S16.** Stool form scale and stool moisture in all samples*. (PDF 1380 kb)*

**Additional file 3.** Supplementary Methods (including detailed custom code and mathematical algorithm).

## Data Availability

The transcriptome data for all liver samples generated during this current study were deposited to the GEO database (Accession # GSE138485) [41]. The previous published microbiome dataset (16S rRNA sequencing) for all stool samples can be downloaded from the European Bioinformatics Institute European Nucleotide Archive database under the accession number PRJEB8708 [11]. Linear discriminant analysis effect size (LEfSe) [15] can be found at http://huttenhower.sph.harvard.edu/galaxy. NCBI Taxonomy ID can be found at https://www.ncbi.nlm.nih.gov/taxonomy. GEPIA [21] can be found at http://gepia.cancer-pku.cn. Landscape and Dynamics of Single Immune Cells in Hepatocellular Carcinoma [23] can be found at http://cancer-pku.cn:3838/HCC. Virtual Metabolic Human database [25] can be found at https://www.vmh.life. The detailed custom code and mathematical algorithm are listed in Additional file 3: Supplementary Methods.

## Abbreviations

HCC: Hepatocellular carcinoma
BMI: Body mass index
Edmondson: Edmondson’s histopathological tumor differentiation
AFP: Alpha-fetoprotein
IHC: Immunohistochemistry
ALT: Alanine aminotransferase
AST: Aspartate aminotransferase
OTU: Operational taxonomy unit
NMDS: Nonmetric multidimensional scaling
LIHC: Hepatocellular carcinoma
CHOL: Cholangial carcinoma
PAAD: Pancreatic adenocarcinoma
COAD: Colon adenocarcinoma
READ: Rectum adenocarcinoma
