# Supplementary material for: Integrated analysis of microbiome and host transcriptome reveals correlations between gut microbiota and clinical outcomes in HBV-related hepatocellular carcinoma
Source: Genome Med. 2020 Nov 23;12:102. doi: 10.1186/s13073-020-00796-5 (PMC7682083; doi:10.1186/s13073-020-00796-5)
Supplement: Supplementary file 1 — Additional file 1: Fig. S1. Study design and flow diagram. Fig. S2. RNA-seq and data analysis. (A) QC passed reads sequenced by an Illumina HiSeq 2500 (pair-end 150-nucleotide read length) for tumors and adjacent non-tumor liver tissues (n = 32, respectively, student’s t test). (B) Parameter of edgeR: plotBCV() and biological coefficient of variation (BCV) was 0.6114732. CPM, counts per million. Fig. S3. There are no differences in gut microbial diversity between healthy controls (n = 100) and HCC patients (n = 113). (A) Shannon-Wiener curves for all 213 fecal samples. Compared with healthy controls, fecal microbial diversities, as estimated by the Shannon index (B), Simpson index (C) and Invsimpson index (D), were no differences with HCC patients (all p > 0.05, Wilcoxon rank sum test). (E) A Venn diagram showed that 729 of the total 1002 OTUs were shared, notably, 148 of 1002 OTUs were unique for HCC. (F) Beta diversity was calculated using Bray-Curtis by NMDS. The red dot represents HCC Group, and the blue dot represents Healthy Control. HCC, hepatocellular carcinoma; OTU, Operational Taxonomy Unit; NMDS, Nonmetric multidimensional scaling. Fig. S4. No differences in gut microbes between patients with Cirrhotic HCC (n = 91) and Non-Cirrhotic HCC (n = 22), between patients with portal hypertension (n = 66) and without portal hypertension (n = 47), between patients with low-albumin (n = 16) and normal-albumin (n = 97). (A) The microbial community at genus level in patients with Cirrhotic HCC versus Non-Cirrhotic HCC. (B) The distributions of Bacteroides, Lachnospiracea incertae sedis and Clostridium XIVa normalized by Z- score among healthy controls, patients with Cirrhotic HCC and Non-Cirrhotic HCC. (C) No differences in Bacteroides, Lachnospiracea incertae sedis and Clostridium XIVa between patients with portal hypertension and without portal hypertension. (D) No differences in Bacteroides, Lachnospiracea incertae sedis and Clostridium XIVa between patients with l [file 13073_2020_796_MOESM1_ESM.pdf]

# Supplementary Figures

# Integrated analysis of microbiome and host transcriptome reveals correlations between gut microbiota and clinical outcomes in HBV-related hepatocellular carcinoma

Hechen Huang[1,2,3], Zhigang Ren[4,1], Xingxing Gao[1,2,3], Xiaoyi Hu[1,2,3], Yuan Zhou[1,2,3], Jianwen Jiang[1], Haifeng Lu[5], Shengyong Yin[1,2,3], Junfang Ji[6], Lin Zhou[1,2,3], Shusen Zheng[1,2,3]

[1]Division of Hepatobiliary and Pancreatic Surgery, Department of Surgery, First Affiliated Hospital, School of Medicine, Zhejiang University, Hangzhou, China
[2]NHFPC Key Laboratory of Combined Multi-organ Transplantation, Hangzhou, China
[3]Key Laboratory of Organ Transplantation, Zhejiang Province, Hangzhou, China
[4]Department of Infectious Diseases, the First Affiliated Hospital of Zhengzhou University, Zhengzhou, China
[5]State Key Laboratory for Diagnosis and Treatment of Infectious Disease; Collaborative Innovation Center for Diagnosis and Treatment of Infectious Diseases, Zhejiang University, Hangzhou, China.
[6]MOE key Laboratory of Biosystems Homeostasis & Protection, Life Sciences Institute, Zhejiang University, Hangzhou, China

Corresponding authors: Professor Shusen Zheng and Professor Lin Zhou.

Hechen Huang, Zhigang Ren and Xingxing Gao contributed equally.

**Fig. S1**

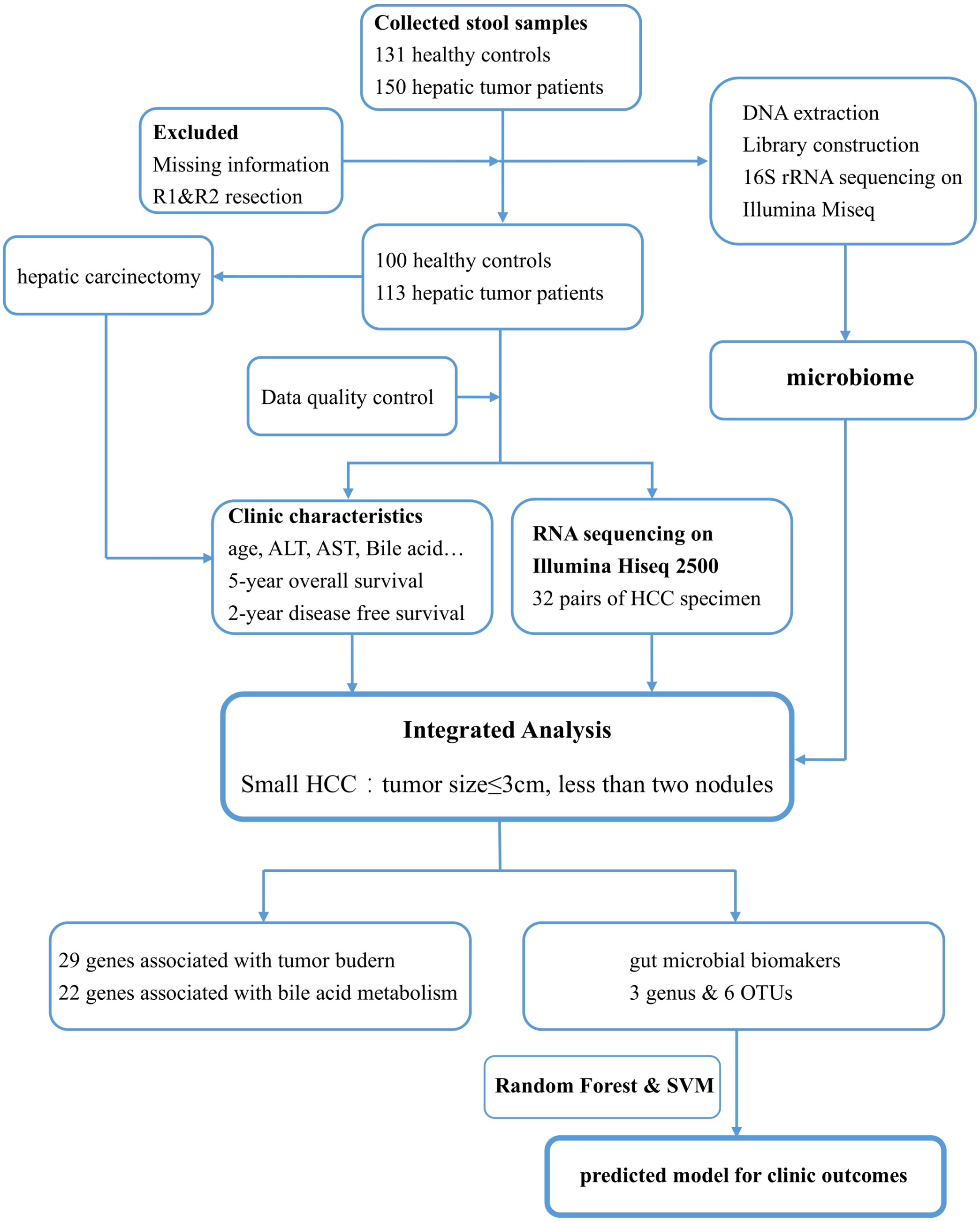


**Fig. S1** Study design and flow diagram.

Fig. S2

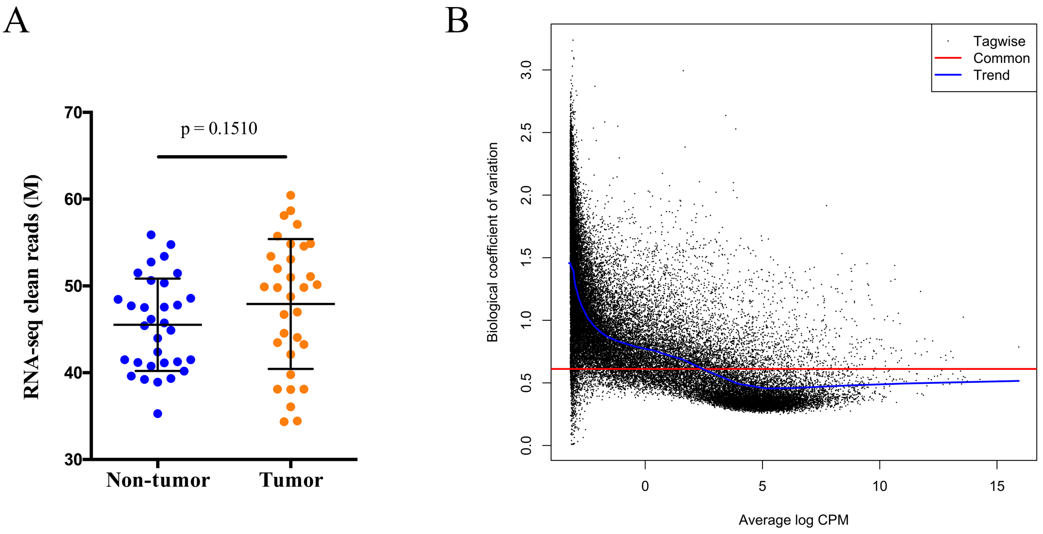


**Fig. S2** RNA-seq and data analysis. (A) QC passed reads sequenced by an Illumina HiSeq 2500 (pair-end 150-nucleotide read length) for tumors and adjacent non-tumor liver tissues (n=32, respectively, student's t test). (B) Parameter of edgeR: plotBCV() and biological coefficient of variation (BCV) was 0.6114732. CPM, counts per million.

**Fig. S3**

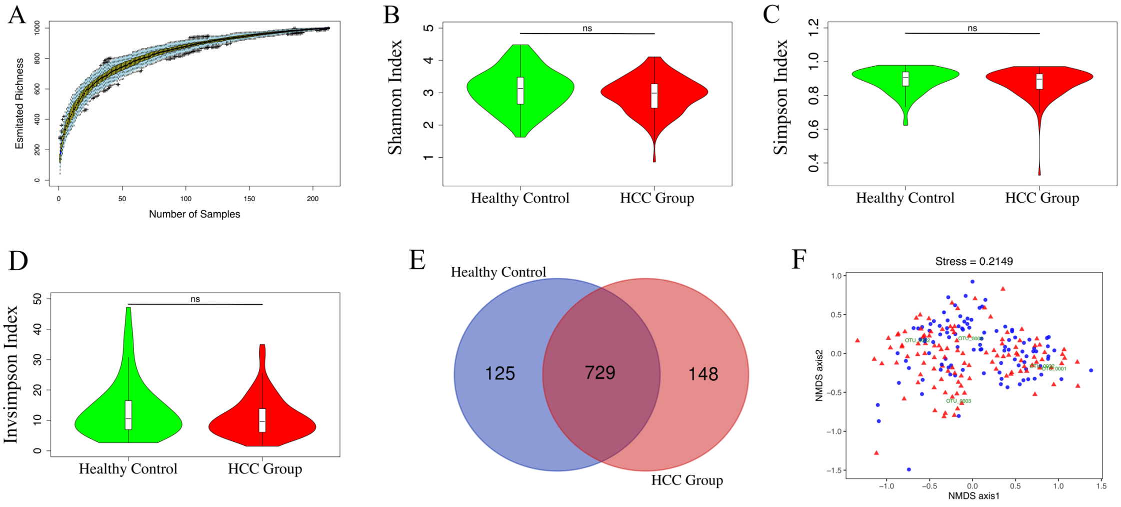


**Fig. S3** There are no differences in gut microbial diversity between healthy controls (n=100) and HCC patients (n=113). (A) Shannon-Wiener curves for all 213 faecal samples. Compared with healthy controls, faecal microbial diversities, as estimated by the Shannon index (B), Simpson index (C) and Invsimpson index (D), were no differences with HCC patients (all p>0.05, Wilcoxon rank sum test). (E) A Venn diagram showed that 729 of the total 1002 OTUs were shared, notably, 148 of 1002 OTUs were unique for HCC. (F) Beta diversity was calculated using Bray-Curtis by NMDS. The red dot represents HCC Group, and the blue dot represents Healthy Control. HCC, hepatocellular carcinoma; OTU, Operational Taxonomy Unit; NMDS, Non-metric multidimensional scaling.

**Fig. S4**

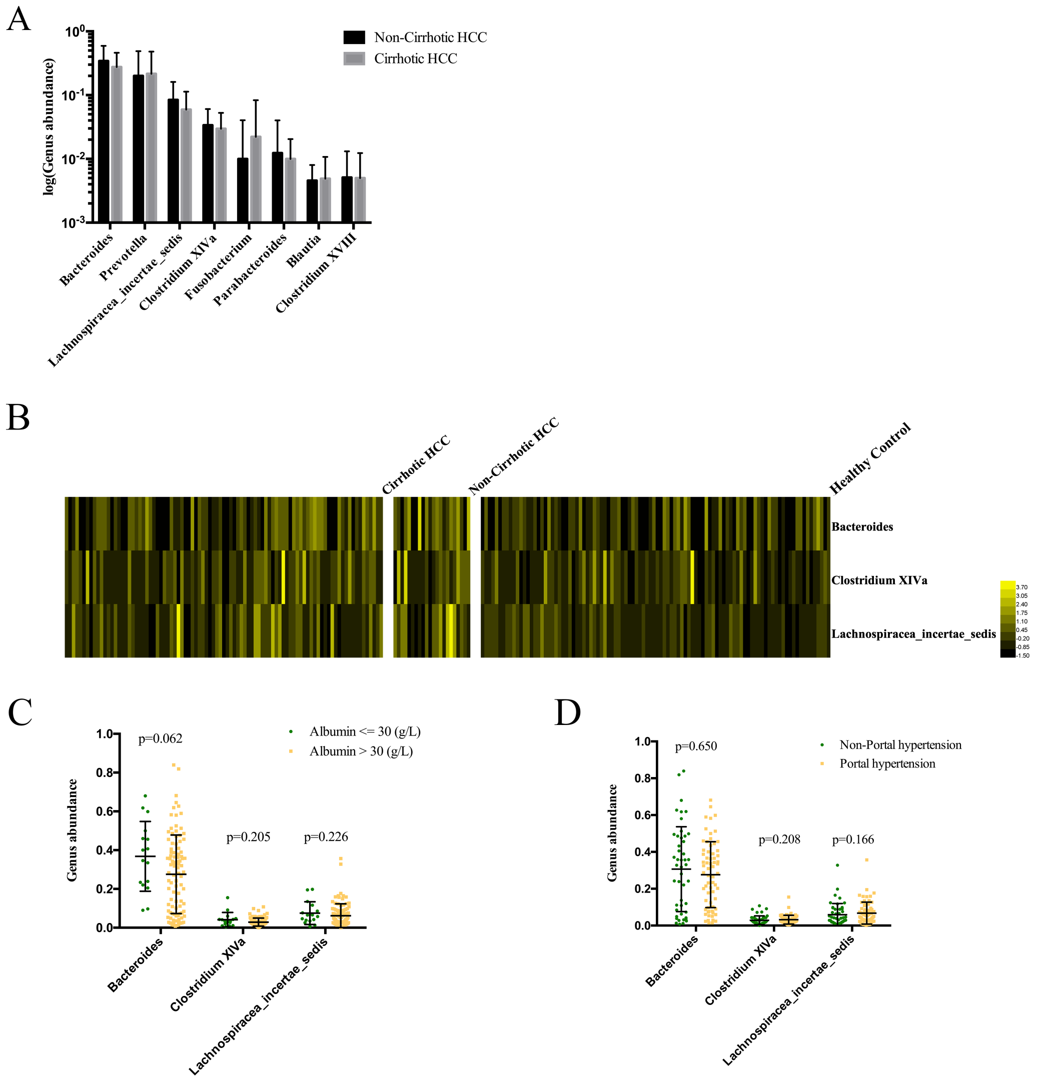


**Fig. S4** No differences in gut microbes between patients with Cirrhotic HCC (n=91) and Non-Cirrhotic HCC (n=22), between patients with portal hypertension (n=66) and without portal hypertension (n=47), between patients with low-albumin (n=16) and normal-albumin (n=97). (A) The microbial community at genus level in patients with Cirrhotic HCC versus Non-Cirrhotic HCC. (B) The distributions of *Bacteroides*, *Lachnospiracea incertae sedis* and *Clostridium XIVa* normalized by Z- score among healthy controls, patients with Cirrhotic HCC and Non-Cirrhotic HCC. (C) No differences in *Bacteroides*, *Lachnospiracea incertae sedis* and *Clostridium XIVa* between patients with portal hypertension and without portal hypertension. (D) No differences in *Bacteroides*, *Lachnospiracea incertae sedis* and *Clostridium XIVa* between patients with low-albumin and normal-albumin. (Wilcoxon rank sum test) HCC, hepatocellular carcinoma.

**Fig. S5**

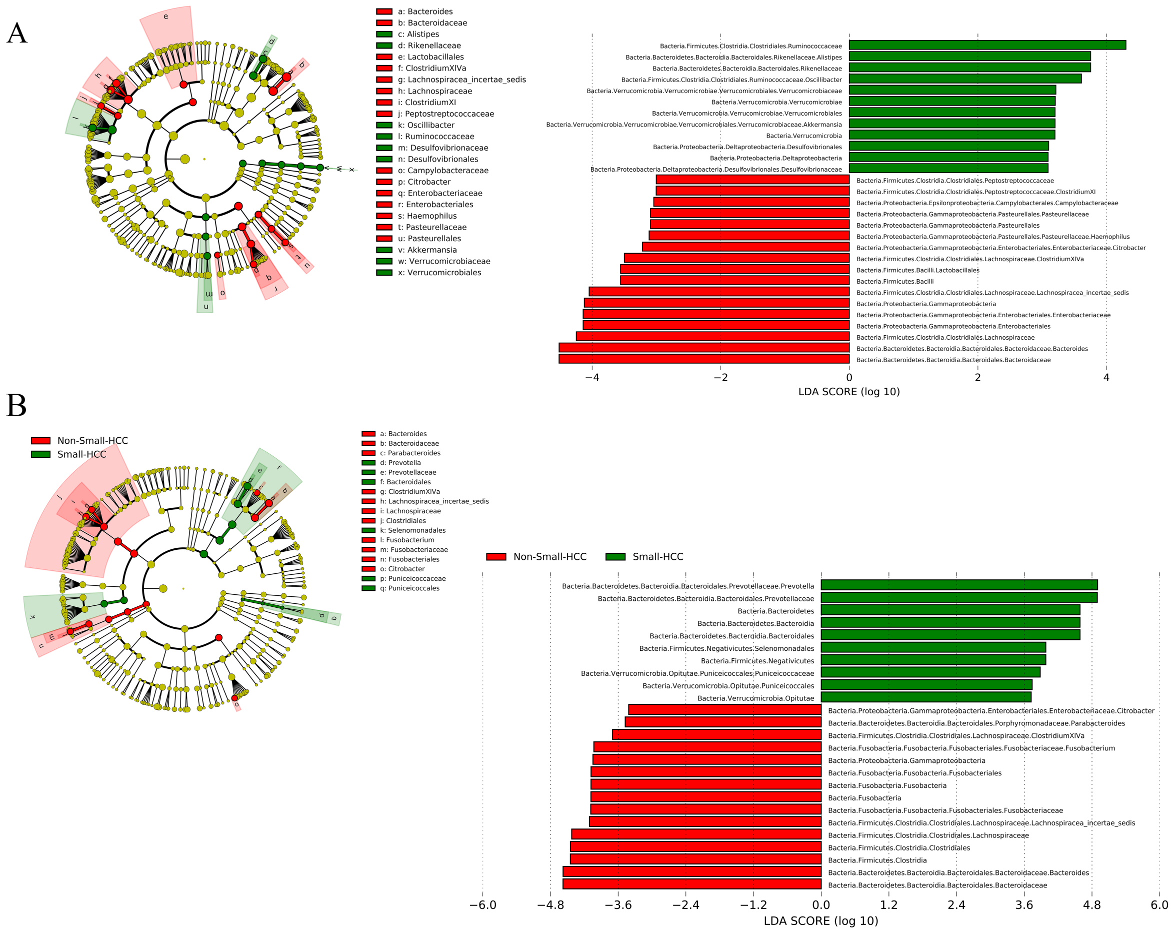


**Fig. S5** The specific characterization of fecal microbiota to distinguish toxigenic types was analyzed by linear discriminant analysis (LDA) effect size (LEfSe) method. (A) LEfSe method identified the most differentially abundant taxons between healthy controls and HCC patients. The HCC-enriched taxa were indicated with a negative LDA score (red), and health-enriched taxa presented a positive score (green). *Bacteroides*, *Lachnospiracea incertae sedis* and *Clostridium XIVa* were significantly overrepresented in the feces of HCC patients. (B) LEfSe method identified the most differentially abundant taxons between Small HCC and Non-Small HCC. The Non-Small HCC-enriched taxa were indicated with a negative LDA score (red), and Small-HCC-enriched taxa presented a positive score (green). *Bacteroides*, *Lachnospiracea incertae sedis* and *Clostridium XIVa* were significantly overrepresented in the feces of patients in Non-Small HCC subgroup. $\log_{10}$(LDA score) = 3 as cut-off value. HCC, hepatocellular carcinoma.

# Fig. S6

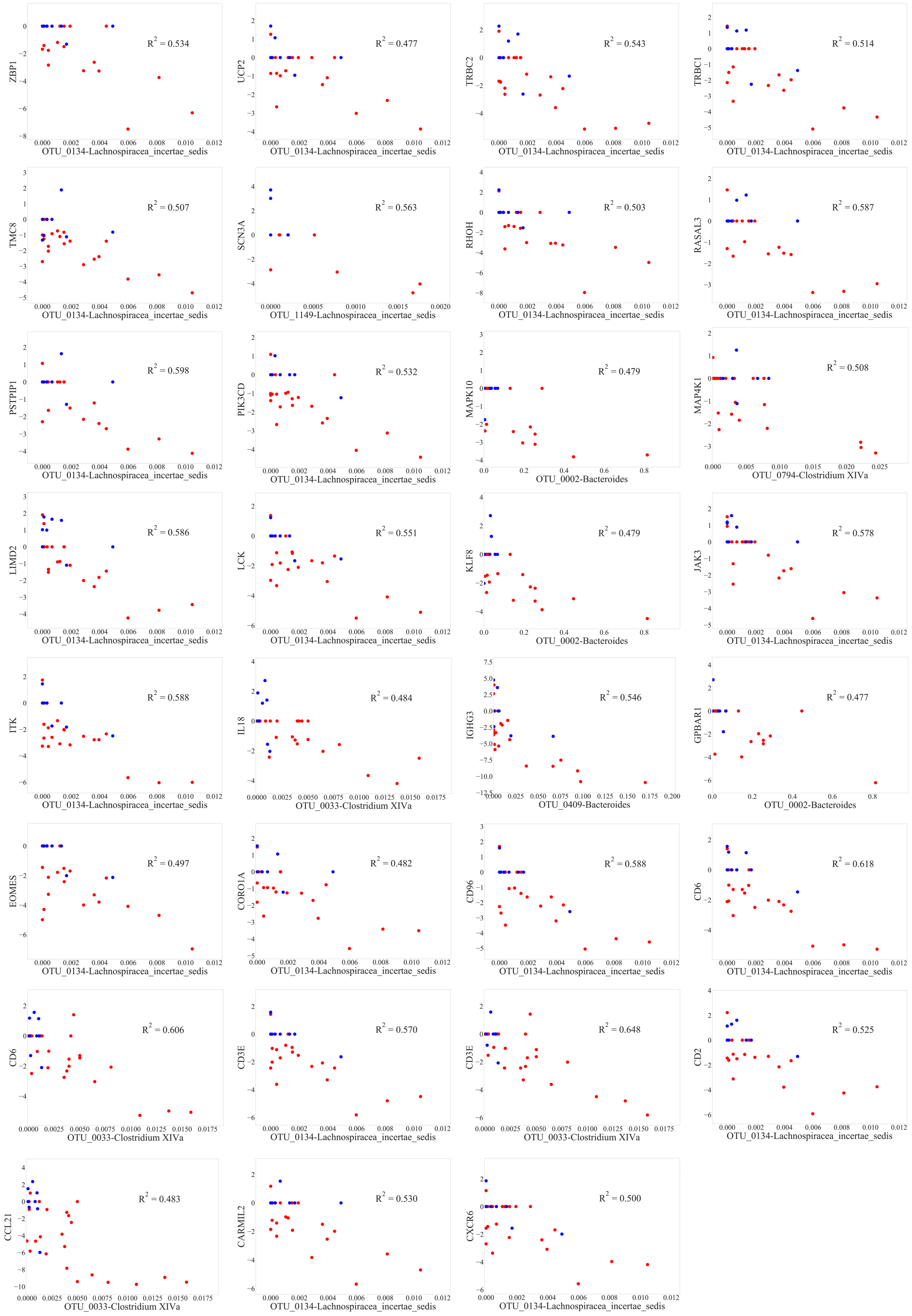


**Fig. S6** Scatter diagrams for all 31 OTU-gene pairs. The x axis indicated the OTU abundance. The y axis indicated log2FC of gene expression calculated by GFOLD. Each point represented a patient (red: Non-Small HCC; blue: Small HCC) and some points coincided at the origin.

**Fig. S7**

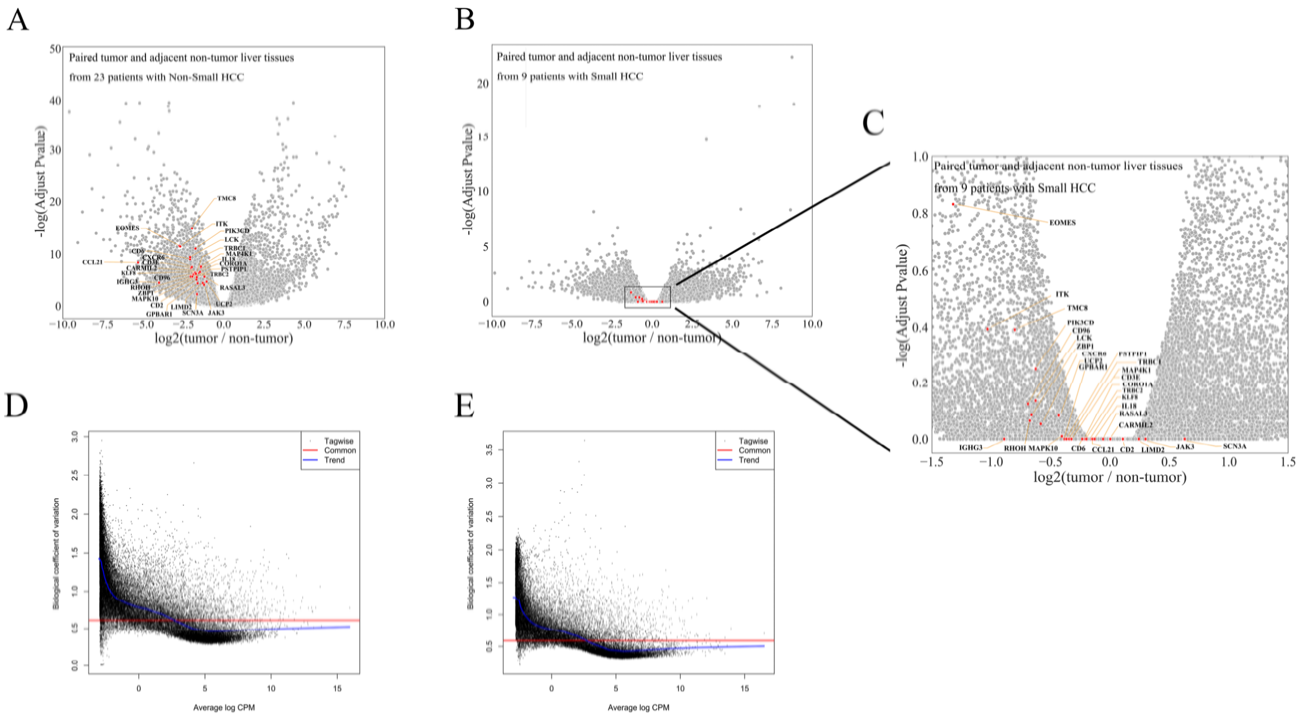


**Fig. S7** The expressions of 29 OTU-related genes in 23 Non-Small HCC (A) and 9 Small HCC patients (B, C). Parameter of edgeR: biological coefficient of variation (BCV) for 23 Non-Small HCC patients was 0.6090678 (D) and BCV for 9 Small HCC patients was 0.5872324 (E).

# Fig. S8

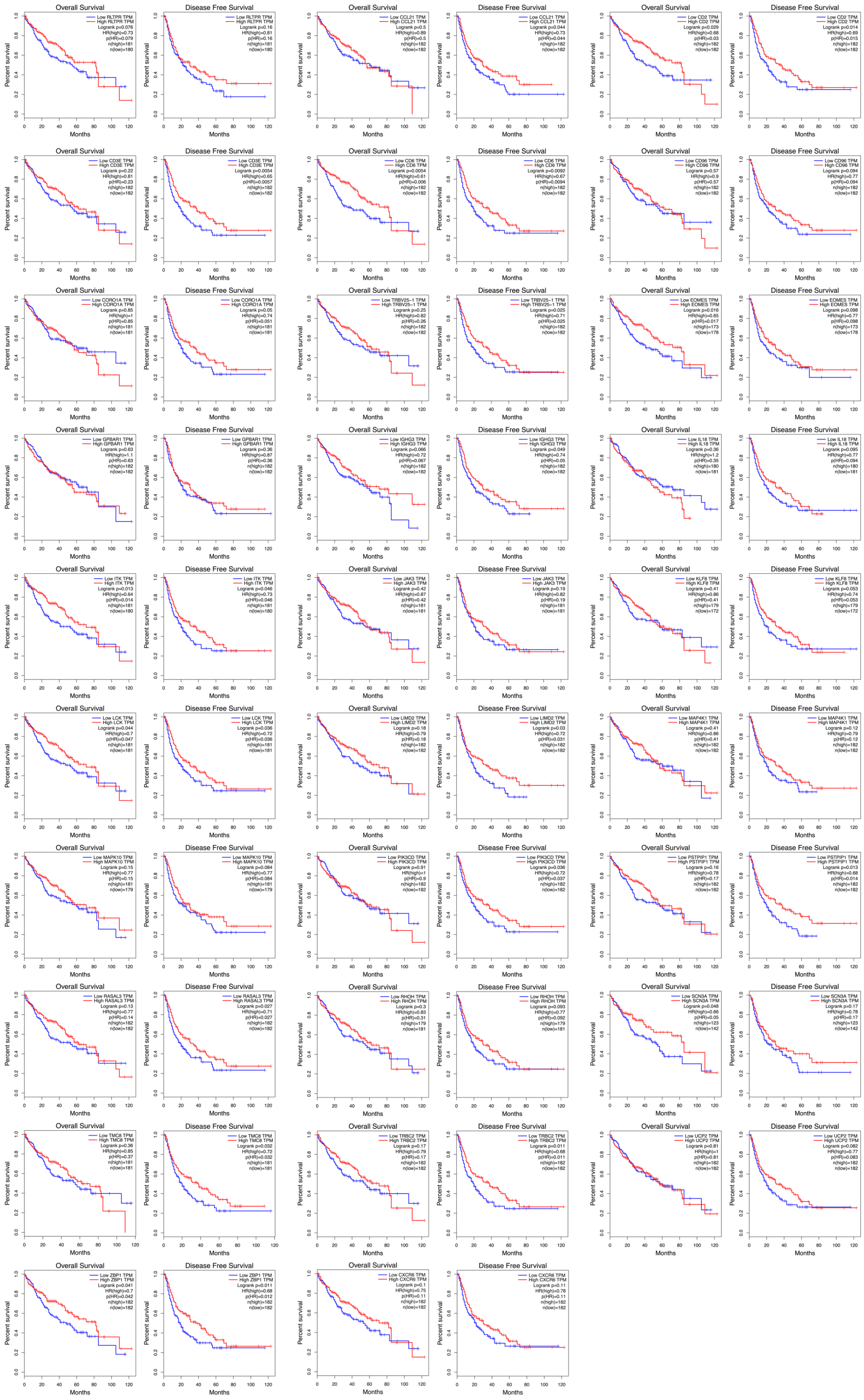

**Fig. S8** Overall survival and disease free survival for all 29 OTU-related genes based on GEPIA. Overall survival and disease free survival were identified by Log-rank test, a.k.a the Mantel-Cox test (Cutoff-High and Cutoff-Low was both 50%). TRBC1 (official symbol) is named TRBV25-1 in GEPIA database.

## Fig. S9

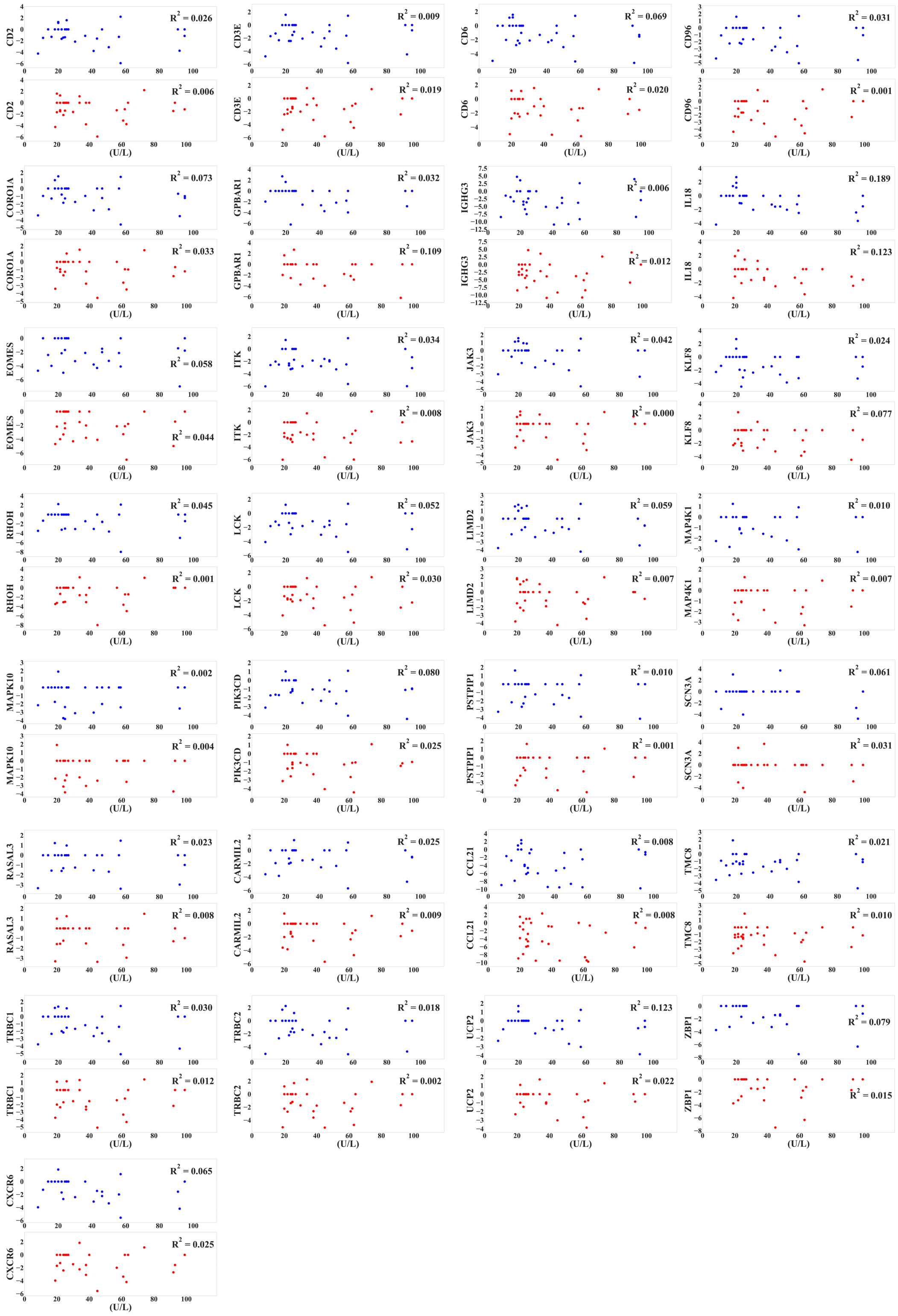


**Fig. S9** Scatter diagrams for all 29 microbe-associated genes and levels of ALT/AST. The x axis indicated the levels of ALT/AST. The y axis indicated log2FC of gene expression calculated by GFOLD. Each point represented a patient (blue: ALT; red: AST).

**Fig. S10**

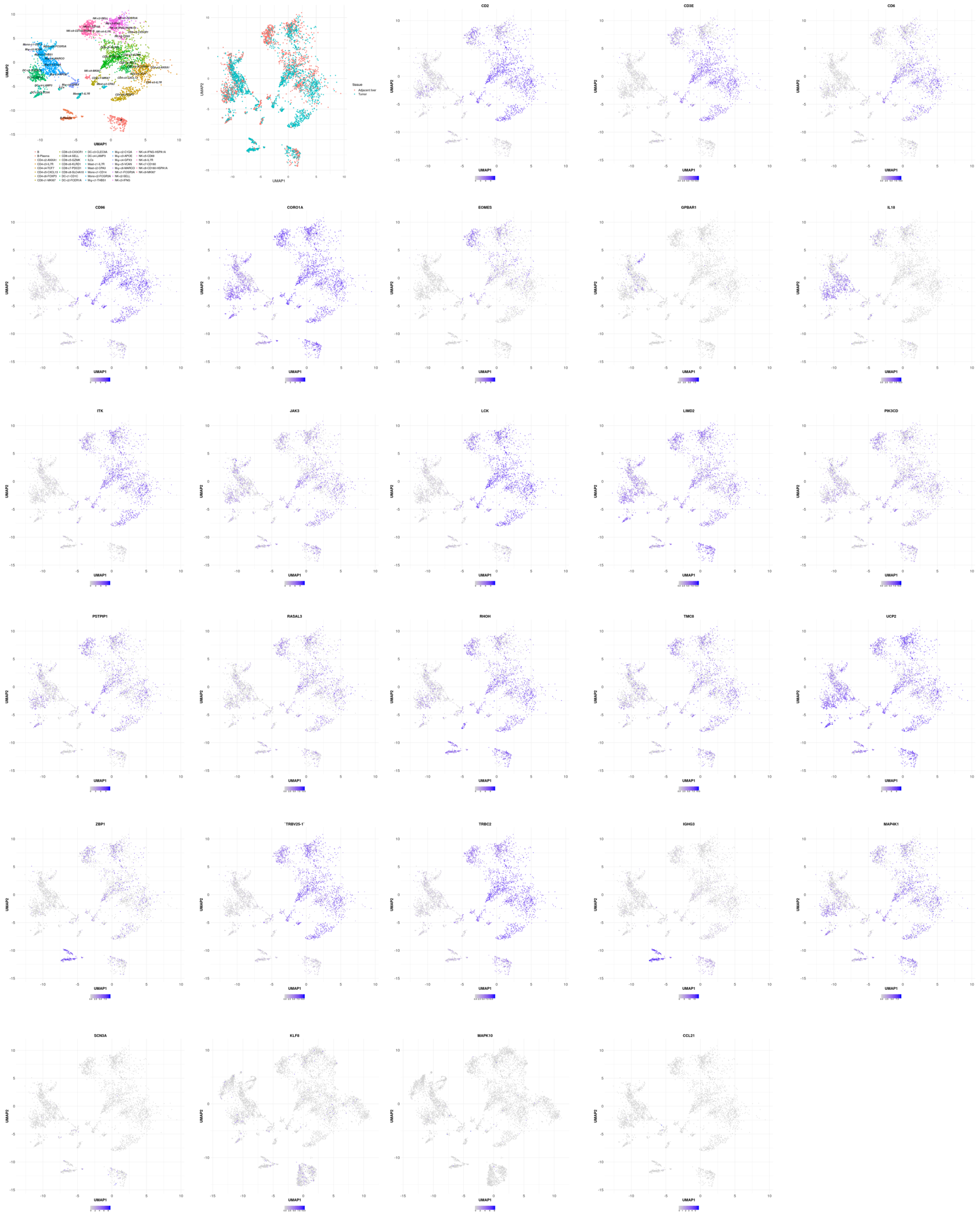

**Fig. S10** Expressions of OTU-related genes for each cell type based on SMART-seq2 data (http://cancer-pku.cn:3838/HCC). Uniform Manifold Approximation and Projection (UMAP) plots showed 38 clusters identified by integrated analysis, colored by cell cluster (first plot). UMAP plots showed the cell distributions of tumor and adjacent liver from 6 patients (second plot). CARMIL2 was not found in this database. TRBC1 (official symbol) is named TRBV25-1 in this database.

**Fig. S11**

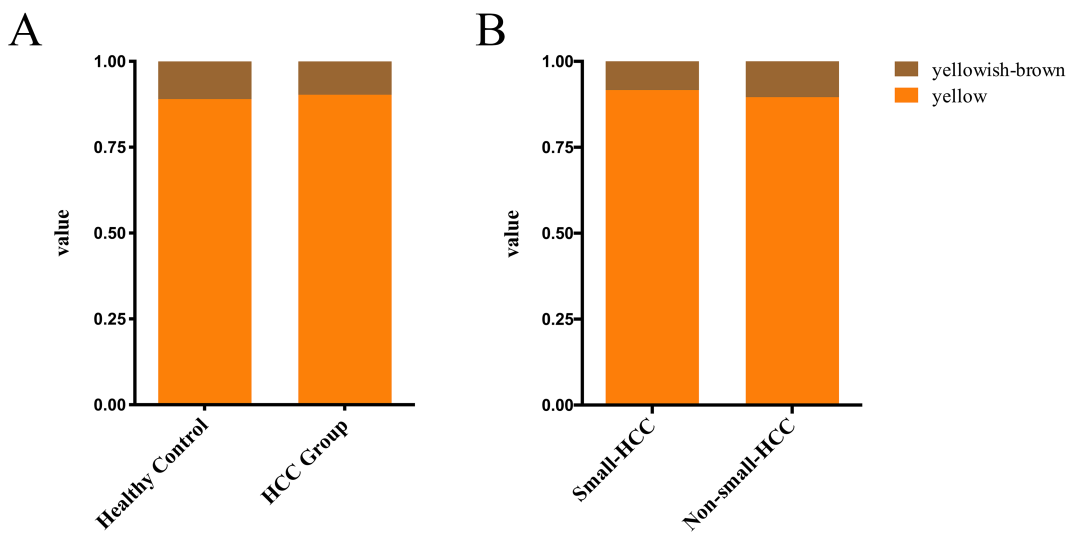


**Fig. S11** The composition of stool color. Most of stool samples presented yellow, and showed no significant difference among the different cohorts. (A) The composition of stool color between healthy controls and HCC patients. (B) The composition of stool color in patients with Small HCC versus Non-Small HCC. HCC, hepatocellular carcinoma.

**Fig. S12**

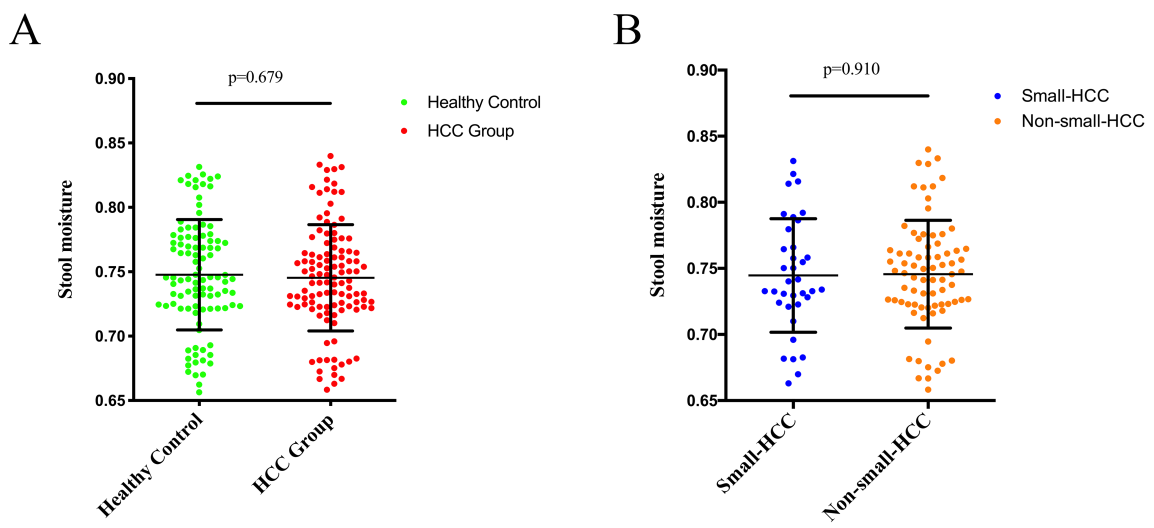


**Fig. S12** The abundance and distribution of stool moisture among the different cohorts by lyophilization assay on the frozen homogenized faecal material. Stool moisture content was determined in duplicate on the frozen homogenized faecal material (-80 °C) as the percentage of stool mass loss after lyophilization. (A) The abundance of stool moisture between healthy controls and HCC patients. (B) The abundance of stool moisture in patients with Small HCC versus Non-Small HCC. (student's t test) HCC, hepatocellular carcinoma.
