# Supplementary material for: Integrated analysis of microbiome and host transcriptome reveals correlations between gut microbiota and clinical outcomes in HBV-related hepatocellular carcinoma
Source: Genome Med. 2020 Nov 23;12:102. doi: 10.1186/s13073-020-00796-5 (PMC7682083; doi:10.1186/s13073-020-00796-5)
Supplement: Supplementary file 2 — Additional file 2: Supplementary Table S1. Clinical phenotype information of individuals with HCC. Supplementary Table S2. Taxon annotation of 1296 OTUs from all samples. Supplementary Table S3. Fecal microbial diversity index in all samples. Supplementary Table S4. three hundred and ten OTUs from all samples. Supplementary Table S5. Clinical phenotype information of healthy participants. Supplementary Table S6. Clinical characteristics summary of all enrolled individuals. Supplementary Table S7. Different degree of phylum level (p value) in Healthy Control and HCC Group. Supplementary Table S8. Different degree of genera level (p value) in Healthy Control and HCC Group. Supplementary Table S9. The interrelationship between 16S OTU clusters of at genus level and taxonomic compositions (NCBI Taxonomy ID). Supplementary Table S10. fifty-six OTU-gene pairs filtered by FDR test of Pearson correlation between OTUs and genes. Supplementary Table S11. Pearson correlation-based analysis of clinical characteristics (all values) and gut microbiota (75 OTUs matched to Bacteroides & Lachnospiracea incertae sedis & Clostridium XIVa). Supplementary Table S12. Pearson correlation-based analysis of clinical characteristics (abnormal values) and gut microbiota (75 OTUs matched to Bacteroides & Lachnospiracea incertae sedis & Clostridium XIVa). Supplementary Table S13. Metabolites of gut microbes according to Virtual Metabolic Human database (http://www.vmh.life). Supplementary Table S14. The relative abundance of six OUT-markers in each sample. Supplementary Table S15. The corresponding bacterial genera of six OTU-markers. Supplementary Table S16. Stool form scale and stool moisture in all samples. (PDF 1380 kb) [file 13073_2020_796_MOESM2_ESM.pdf]

Supplementary Tables

Integrated analysis of microbiome and host transcriptome reveals correlations between gut microbiota and clinical outcomes in HBV-related hepatocellular carcinoma

Hechen Huang[1,2,3], Zhigang Ren[4,1], Xingxing Gao[1,2,3], Xiaoyi Hu[1,2,3], Yuan Zhou[1,2,3], Jianwen Jiang[1], Haifeng Lu[5], Shengyong Yin[1,2,3], Junfang Ji[6], Lin Zhou[1,2,3], Shusen Zheng[1,2,3]

[1]Division of Hepatobiliary and Pancreatic Surgery, Department of Surgery, First Affiliated Hospital, School of Medicine, Zhejiang University, Hangzhou, China
[2]NHFPC Key Laboratory of Combined Multi-organ Transplantation, Hangzhou, China
[3]Key Laboratory of Organ Transplantation, Zhejiang Province, Hangzhou, China
[4]Department of Infectious Diseases, the First Affiliated Hospital of Zhengzhou University, Zhengzhou, China
[5]State Key Laboratory for Diagnosis and Treatment of Infectious Disease; Collaborative Innovation Center for Diagnosis and Treatment of Infectious Diseases, Zhejiang University, Hangzhou, China.
[6]MOE key Laboratory of Biosystems Homeostasis & Protection, Life Sciences Institute, Zhejiang University, Hangzhou, China

Corresponding authors: Professor Shusen Zheng and Professor Lin Zhou.

Hechen Huang, Zhigang Ren and Xingxing Gao contributed equally.

Supplementary Tables

1. supplementary Table **S1** Clinical phenotype information of individuals with HCC *(Page 1-6)*
2. supplementary Table **S2** Taxon annotation of 1296 OTUs from all samples *(Page 7-32)*
3. supplementary Table **S3** Fecal microbial diversity index in all samples *(Page 33-37)*
4. supplementary Table **S4** three hundred and ten OTUs from all samples *(Page 38-44)*
5. supplementary Table **S5** Clinical phenotype information of healthy participants *(Page 45-48)*
6. supplementary Table **S6** Clinical characteristics summary of all enrolled individuals *(Page 49)*
7. supplementary Table **S7** Different degree of phylum level (p value) in Healthy Control and HCC Group *(Page 50)*
8. supplementary Table **S8** Different degree of genera level (p value) in Healthy Control and HCC Group *(Page 51-60)*
9. supplementary Table **S9** The interrelationship between 16S OTU clusters of at genus level and taxonomic compositions (NCBI Taxonomy ID) *(Page 61)*
10. supplementary Table **S10** fifty-six OTU-gene pairs filtered by FDR test of Pearson correlation between OTUs and genes *(Page 62-63)*
11. supplementary Table **S11** Pearson correlation-based analysis of clinical characteristics (all values) and gut microbiota (75 OTUs matched to *Bacteroides* & *Lachnospiracea incertae sedis* & *Clostridium XIVa*) *(Page 64-69)*
12. supplementary Table **S12** Pearson correlation-based analysis of clinical characteristics (abnormal values) and gut microbiota (75 OTUs matched to *Bacteroides* & *Lachnospiracea incertae sedis* & *Clostridium XIVa*) *(Page 70-75)*
13. supplementary Table **S13** Metabolites of gut microbes according to Virtual Metabolic Human database (http://www.vmh.life) *(Page 76-82)*
14. supplementary Table **S14** The relative abundance of six OUT-markers in each sample *(Page 83-85)*
15. supplementary Table **S15** The corresponding bacterial genera of six OTU-markers *(Page 86)*
16. supplementary Table **S16** Stool form scale and stool moisture in all samples *(Page 87-91)*

supplementary Table S1 Clinical phenotype information of individuals with HCC

ID	Gender	Age (year)	height (cm)	weight (kg)	BMI (kg/m2)	Edmondson degree	AFP in IHC	tumor size	AFP in serum (ng/mL)	ALT (U/L)	AST (U/L)	GTT (U/L)	Total Protein (g/L)	ALB (g/L)	GLO (g/L)	PT (s)	TB (μ mol/L)	DB (μ mol/L)	IB (μ mol/L)	fasting blood glucose (mmol/L)	triglyceride (mmol/L)
zfh001	male	56	160	58	22.66	2	-	non-small	1.8	57	45	34	61.7	40.8	20.9	10.3	10	2	8	8.96	0.84
zfh003	male	57	178	73.5	23.2	2	-	non-small	4.9	14	25	20	65.2	40	25.2	11.5	16	6	10	5.06	1.18
zfh004	male	56	165.5	71.5	26.1	3	-	non-small	31417.7	50	61	92	74.7	44.8	29.9	12.9	16	7	9	4.79	1.19
zfh005	male	43	173.5	69	22.92	3	-	non-small	8.9	43	30	64	65.9	39.6	26.3	11	7	2	5	4.07	1.95
zfh006	male	50	181	62.5	19.08	2	-	non-small	14.4	24	25	29	56.4	35.8	20.6	11.8	4	2	2	4.63	0.67
zfh007	male	53	175	85	27.76	3	-	non-small	2.2	22	20	20	64.5	40.1	24.4	11.6	11	4	7	4.81	1
zfh009	male	52	158	62	24.84	2	+	non-small	＞80000.0	57	74	84	64.1	38.4	25.7	12.2	23	9	14	4.5	0.86
zfh010	female	57	163	55	20.7	1	-	non-small	339.2	95	99	257	69.4	33.8	35.6	12.2	8	3	5	4.56	0.96
zfh011	male	60	160	53	20.7	2	-	non-small	4.5	24	25	92	63.1	37.3	25.8	10.8	15	6	9	4.76	0.88
zfh012	male	53	168	64	22.68	3	-	non-small	2.7	92	63	204	74.9	36.4	38.5	12.4	8	4	4	3.26	0.96
zfh013	male	50	170	79	27.34	2	+	non-small	206	16	22	77	57.9	33.3	24.6	10.6	13	5	8	4.73	0.76
zfh014	male	57	162	50	19.05	2	+	non-small	36134.6	91	93	588	63.4	34.1	29.3	17.2	355	264	91	4.59	2.46
zfh015	male	51	168	74	26.22	2	+	non-small	3918.6	25	40	153	74.9	45.4	29.5	11.6	16	6	10	5.44	1.98
zfh016	male	50	180	61	18.83	3	-	non-small	4986.5	36	62	104	64.4	37.8	26.6	10.7	17	7	10	4.1	0.7
zfh017	male	65	165	60	22.04	3	-	non-small	12.8	18	24	31	73.3	42.4	30.9	12.1	17	9	8	4.42	0.9
zfh018	male	48	168	70	24.8	2	-	non-small	963.6	23	92	108	73.6	42.2	31.4	11.1	19	7	12	4.77	1.8
zfh019	male	47	165	60	22.04	1	+	non-small	67.5	11	22	102	72.6	40	32.6	12.2	12	5	7	4.2	1.4
zfh020	male	60	173	70	23.39	3	-	non-small	1.7	22	24	21	64.7	39.9	24.8	12.6	15	11	4	5.1	0.74
zfh021	male	60	169	45	15.76	2	-	non-small	3.7	8	19	23	68.4	39.4	29	11.9	10	4	6	5.06	0.79
zfh022	female	59	160	66	25.78	3	-	non-small	3.6	41	38	40	73.9	45.8	28.1	12	18	6	12	4.79	1.19
zfh023	male	60	172	71	24	1	-	non-small	12.9	46	34	46	61.2	35	26.2	10.9	7	2	5	4.9	0.79
zfh024	male	39	175	70	22.86	2	-	small	37.1	26	27	41	78.6	47.3	31.3	12.7	17	4	13	4.73	0.87
zfh025	female	57	152	55	23.81	2	+	small	122.7	46	38	72	69.1	41.8	27.3	13.1	20	10	10	5	0.88
zfh026	male	57	166	62	22.5	3	+	non-small	35	30	24	20	70.6	38.2	32.4	13	23	10	13	4.1	0.53
zfh027	male	51	168	67	23.74	1	+	small	15.7	25	20	17	63.4	37	26.4	10.9	20	10	10	5.07	0.85
zfh028	male	61	170	59.5	20.59	3	-	small	4	18	26	14	62.9	37.6	25.3	11.3	11	5	6	4.29	0.68
zfh029	male	52	165	57.5	21.12	3	+	small	38.3	18	22	18	61.2	39.2	22	13.9	13	6	7	4.38	0.59
zfh030	male	45	176	86	27.76	2	-	non-small	10.2	95	64	172	64.8	38	26.8	18	11	4	7	4.65	0.75
zfh031	male	34	165	58	21.3	3	-	small	4.7	56	57	52	75.5	45.5	30	10.4	8	4	4	3.54	1.27
zfh032	male	60	160	64	25	3	+	small	49.7	20	34	138	73.4	44	29.4	13.1	17	7	10	3.99	1.33
zfh033	male	56	163	53	19.95	1	-	small	4.4	20	22	16	67.2	42	25.2	10.5	18	7	11	5.13	0.73
zfh034	male	62	170	58	20.07	2	-	small	54.9	20	20	36	67.4	42	25.4	11.3	20	8	12	4.42	1.75
zfh035	male	56	164	62	23.05	3	-	non-small	4.5	48	44	74	63	37	25.9	11	15	6	9	3.94	0.73
zfh037	male	62	170	66	22.84	2	-	non-small	76.7	33	39	242	76.6	42.7	33.9	11.8	19	4	15	4.99	1.3
zfh038	male	60	182	75	22.64	3	-	non-small	3	28	27	56	66.6	34.5	32.1	11.9	16	5	11	5.72	0.92
zfh041	male	50	168	54	19.13	3	+	non-small	404	16	27	31	62.9	37.8	25.1	10.6	11	4	7	4.89	0.96
zfh042	male	55	171	66	22.57	3	+	non-small	29277	13	36	50	73.6	40.5	33.1	11.3	10	3	7	4.78	1.44
zfh044	male	69	173	70	23.39	3	-	small	6.1	24	26	88	60.7	39.7	21	13	11	7	4	4.56	0.73
zfh045	male	47	158.6	52	20.67	1	-	small	3.1	20	21	20	67.6	38.1	29.5	13.4	14	5	9	4.15	0.79
zfh046	male	37	169	71.3	24.96	2	-	small	4.4	17	17	20	62.3	37.1	25.2	16.6	9	4	5	5.02	0.65
zfh101	male	43	175	77	25.14	3	-	non-small	5.4	37	24	59	70.5	41.7	28.8	11.2	8	3	5	4.9	1.18
zfh102	male	43	175	80	26.12	2	-	non-small	3.5	22	25	25	67.5	41.7	25.8	11.5	25	8	17	4.69	1.34

zfh103	male	51	175	83.5	27.27	2	+	small	923.7	35	28	67	74.7	40.3	34.4	12.9	15	7	8	5.24	0.74
zfh104	male	46	163	79	29.73	3	+	small	1202.2	32	26	24	66.7	42.2	24.5	16.6	13	5	8	4.88	1.11
zfh105	male	54	175	53	17.31	2	-	non-small	1.9	20	24	79	66.3	43.5	22.8	12.1	10	4	6	4.08	0.69
zfh106	male	57	175	93	30.37	2	-	non-small	7.5	29	30	58	61.2	32.9	28.3	13.6	24	9	15	5.54	1.08
zfh107	female	44	159	47	18.59	2	+	small	543.9	10	16	7	77.9	49.9	28	11.2	27	10	17	5.08	0.54
zfh108	female	44	160	55	21.48	2	+	small	4.5	23	42	89	70.5	43	27.5	12.5	13	5	8	5.06	0.63
zfh109	male	53	168	68	24.09	1	-	small	1.5	8	17	21	65.6	37.6	28	12.8	7	2	5	4.29	0.87
zfh110	male	63	163	66.9	25.18	4	+	non-small	4395	54	61	59	74.8	39.1	35.7	12.8	9	1	8	4.17	2.01
zfh111	female	55	154	46	19.4	3	+	small	139.1	22	23	16	65.2	43.3	21.9	13	10	3	7	4.61	1.74
zfh112	male	42	162	77	29.34	2	-	non-small	104.8	37	55	56	63.4	27.3	36.1	16.4	44	11	33	3.92	0.79
zfh113	male	53	168	56	19.84	3	+	non-small	21984.8	21	28	47	59.4	37.2	22.2	10.5	5	2	3	4.08	0.65
zfh114	male	38	170	71	24.57	2	+	non-small	4227.7	23	20	23	71.2	47.7	23.5	10.3	8	3	5	4.37	1.28
zfh115	male	50	180	76	23.46	2	-	small	170.8	19	29	50	68.6	42.1	26.5	11.7	14	3	11	4.62	1.01
zfh116	male	47	171	78	26.67	2	-	non-small	21.9	51	69	102	64.8	35.6	29.2	19.2	27	5	22	3.77	1.24
zfh117	male	44	170	78	26.99	2	-	non-small	31	61	91	416	64	33.6	30.4	11.9	21	8	13	4.12	0.82
zfh118	male	56	168	56	19.84	2	-	non-small	165.4	33	29	27	74	45.9	28.1	12.3	16	4	12	3.83	1
zfh119	male	57	154	54	22.77	2	+	small	261	99	51	123	70.5	43.8	26.7	12.7	13	3	10	3.77	1.23
zfh120	male	43	168	58	20.55	1	-	small	14.7	22	21	28	69.7	42.3	27.4	13.1	18	5	13	4.31	0.9
zfh121	male	58	158	56	22.43	2	-	non-small	43.4	47	52	318	76	39.8	36.2	11.5	15	3	12	4.02	1.15
zfh122	male	58	168	63.5	22.5	2	-	non-small	2.9	30	21	140	68.7	41.8	26.9	11.7	17	4	13	5.14	0.92
zfh123	male	49	168	60.5	21.44	2	+	small	36.8	19	23	24	58.2	35.4	22.8	14.1	21	9	12	4.38	0.46
zfh124	male	41	178.5	77.5	24.32	2	-	non-small	31660.5	33	46	225	64.3	32.8	31.5	12.4	16	7	9	6.91	1.92
zfh125	male	56	165	53	19.47	3	+	non-small	1294.3	92	91	142	74.5	34.5	40	13.5	46	19	27	4.24	0.94
zfh126	male	46	170	54	18.69	2	-	non-small	363.2	26	32	105	72	42.4	29.6	12.5	11	4	7	4.27	0.67
zfh127	male	50	174	70	23.12	1	-	non-small	5.1	25	26	56	75.1	28.1	47	11.9	9	3	6	7.59	0.92
zfh128	male	48	170	71.5	24.74	2	-	non-small	91.2	89	69	139	65.9	35	30.9	13.5	23	11	12	5.36	0.56
zfh129	male	44	160	59	23.05	3	+	non-small	6776.7	43	43	80	59.7	35.6	24.1	12.9	24	12	12	4.28	0.63
zfh130	male	45	177	64	20.43	4	+	non-small	2705.6	16	25	26	74.1	46.1	28	11.8	18	7	11	4.97	0.62
zfh131	male	36	170	65.5	22.66	2	+	non-small	9485.1	31	25	200	66.6	35.2	31.4	12	26	10	16	6.97	1.02
zfh132	male	46	163	60.5	22.77	2	-	non-small	419.4	49	74	205	68.1	32.6	35.5	14.4	31	17	14	5.45	0.49
zfh133	male	43	165	60	22.04	3	+	small	530.4	27	26	18	65.4	36.9	28.5	12.1	9	4	5	4.26	1.21
zfh134	male	52	158	62	24.84	3	+	non-small	>80000	57	74	84	64.1	38.4	25.7	11.1	23	9	14	4.5	0.86
zfh135	male	49	170	75	25.95	2	+	non-small	3021.5	33	29	95	64	39.6	24.4	11.8	5	2	3	5.85	2.6
zfh136	male	56	165	71	26.08	2	-	small	9.8	39	73	71	57.3	28	29.4	15.2	21	7	14	4.51	0.86
zfh137	female	45	160	54	21.09	3	+	small	266.2	7	13	12	73.6	44.2	29.4	10.8	10	3	7	4.87	0.87
zfh138	male	48	165	65	23.88	2	-	non-small	6359.5	62	92	225	72.3	38.4	33.9	13.3	45	27	18	5.54	1.26
zfh139	male	51	168	54	19.13	2	-	non-small	92.5	38	58	127	66.4	34.3	32.1	13.5	20	11	9	4.79	0.7
zfh140	male	61	173	68	22.72	2	-	non-small	3.1	27	27	113	66.4	40.7	25.7	11.8	12	4	8	5.04	0.8
zfh141	male	56	173	61	20.38	3	+	non-small	1525.6	10	15	24	72.6	45.7	26.9	11.3	15	7	8	5.6	1.21
zfh142	male	57	162	60	22.86	3	+	small	123.1	46	46	213	74.8	37.9	36.9	12.5	11	5	6	4.64	0.98
zfh143	male	46	171	72.5	24.79	2	-	small	4.5	39	26	37	75.4	40.8	34.6	10.9	8	3	5	4.48	0.81
zfh144	male	44	165	54.5	20.02	2	-	non-small	7.3	83	63	240	77.7	44.2	33.5	12.7	25	11	14	5.43	0.56
zfh145	male	47	181	80.5	24.57	2	-	non-small	456.3	51	36	34	64.6	37.7	26.9	11.7	23	8	15	5.09	0.7
zfh146	male	28	175	75	24.49	2	-	non-small	269.1	30	26	61	70.6	41.9	28.7	12.3	23	10	13	4.25	0.83
zfh147	male	61	159	61	24.13	2	-	non-small	2.3	56	60	47	60.9	41.5	19.4	10.8	10	3	7	4.89	1.13
zfh148	male	43	165	61	22.41	1	-	non-small	6.6	17	27	268	78.3	35.8	42.5	13.3	9	4	5	4.84	1.02
zfh149	male	58	156	53	21.78	2	-	non-small	82.6	23	40	40	68.2	33.1	35.1	14.3	38	20	18	4.58	0.63
zfh150	male	43	170	65	22.49	3	+	small	775.7	16	16	20	73.8	44.3	29.5	11.5	24	8	16	4.49	1.24

zfh151	female	59	169	64	22.41	3	-	non-small	>80000	25	32	83	71	40.3	30.7	11.2	10	2	8	5.19	1.47
zfh152	male	22	170	70	24.22	3	+	small	7783.8	31	23	38	65.3	42.2	23.1	11.8	28	13	15	3.6	0.82
zfh153	male	57	182	85	25.66	2	-	small	15.5	46	24	284	66	37.9	28.1	11	12	4	8	5.95	0.83
zfh154	male	60	170	60	20.76	1	+	small	11.8	51	43	29	74.3	42.1	32.2	12.9	12	5	7	4.78	1.02
zfh155	male	35	170	68	23.53	2	+	small	842.3	36	23	69	67	40.9	26.1	11.4	22	9	13	5	1.23
zfh156	male	51	167	55	19.72	2	-	small	1.9	20	40	61	70.9	37.7	33.2	12.6	10	5	5	4.78	0.7
zfh157	male	59	170	58	20.07	2	-	non-small	19.2	24	44	126	65.9	36.8	29.1	13	15	8	7	4.31	0.69
zfh158	male	60	168	69	24.45	2	-	non-small	92.9	32	33	80	76	42.1	33.9	12.3	13	5	8	5.7	0.69
zfh159	male	50	156	48	19.72	3	-	small	5.6	17	28	63	67.3	42	25.3	11.5	11	6	5	5.32	0.72
zfh160	male	46	156	51	20.96	2	-	non-small	>80000	48	56	485	66.2	36	30.2	13.3	39	21	18	5.17	0.57
zfh161	male	54	172	66	22.31	2	+	non-small	9501.1	48	44	240	62.7	35.5	27.2	10.4	31	21	10	4.42	0.69
zfh162	male	50	165	65	23.88	2	-	non-small	11.7	39	33	78	72	43.4	28.6	11.8	14	6	8	5.36	1.35
zfh163	male	56	170	57.5	19.9	2	-	small	2	44	35	61	67.1	39.5	27.6	12.1	24	10	1	4.86	0.78
zfh164	male	59	167	55	19.72	2	-	non-small	273.8	23	27	22	74.2	46.9	27.3	11	14	7	7	4.77	0.74
zfh165	male	52	173	65	21.72	2	-	non-small	2.1	24	26	53	64	42.6	21.4	12	14	5	9	4.81	0.86
zfh166	male	44	170	72	24.91	2	-	non-small	>80000	47	93	238	72	46	26	12.9	17	8	9	4.27	0.75
zfh167	male	54	183	62	18.51	2	-	non-small	5.5	42	32	69	74.5	45.2	29.3	10.8	8	4	4	5.64	1.62
zfh168	male	46	168	60	21.26	2	-	non-small	259.9	59	52	92	63.8	36	27.8	12.1	19	9	10	4.21	0.64
zfh169	male	66	157	58	23.53	2	-	non-small	2.6	27	49	23	70.3	29.3	41	14.2	28	13	15	4.12	0.62
zfh170	male	47	170	52	17.99	2	-	non-small	35634.5	21	35	109	74.8	48.9	25.9	12.3	31	12	19	5.26	0.65
zfh171	male	47	163	55	20.7	2	-	non-small	2.6	42	42	150	64.4	33.3	31.1	15.5	11	7	4	4.58	0.83
zfh172	female	66	160	50	19.53	3	+	small	104	8	19	15	71.7	44.4	27.3	11.5	35	12	23	5.39	1.63
zfh173	male	50	172	66	22.31	2	-	non-small	4.6	33	30	128	65	40.6	24.4	12.7	21	10	1	4.86	0.59

total cholesterol (mmol/L)	total serum bile acid (umol/L)	HDL (mmol/L)	LDL (mmol/L)	CEA (ng/mL)	CA199 (U/mL)	CA125 (U/mL)	Child-Pugh	Cirrhotic HCC	portal hypertension	Tumor thrombus	HBV infection	overall survival (month)	disease free survival (month)
4.81	28	1.09	2.76	1.2	13.2	5	A	No	No	No	Postive	72	72
3.71	3	0.99	1.87	2.5	9.9	10.7	A	No	No	No	Postive	71	71
4.12	10	0.85	1.97	2.7	3.5	53.9	A	Yes	Yes	Yes	Postive	30	12
4.59	12	0.76	2.6	1.5	8.3	6.8	A	Yes	Yes	Yes	Postive	71	71
3.95	12	1.22	2.02	1.5	<2	10.6	A	Yes	Yes	No	Postive	33	8
4.31	4	0.88	2.54	1.7	12.2	8.1	A	No	No	No	Postive	70	70
4.4	8	1.37	2.21	1.6	8.4	17.9	A	Yes	Yes	No	Postive	69	55
4.15	30	0.94	2.18	6.2	37.7	29	A	Yes	Yes	Yes	Postive	9	3
4.22	10	1	2.3	4.7	37.6	17.8	A	Yes	No	No	Postive	16	11
5.9	45	0.77	3.73	2	14.6	17.9	A	No	No	No	Postive	28	22
3.82	7	0.84	2.06	3.3	13.7	8.2	A	Yes	No	No	Postive	68	35
8.77	20	0.36	2.22	3.5	309.4	29.8	B	No	No	No	Postive	62	26
5.03	21	1.15	2.82	1.6	8.9	11	A	Yes	Yes	Yes	Postive	4	3
3.34	3	1.26	1.69	1.1	8.9	8.8	A	No	No	No	Postive	12	8
3.45	4	1.09	1.75	5.2	14.7	9	A	No	No	Yes	Postive	63	63
4.67	10	1.33	3.05	1.9	7.7	11.3	A	No	No	No	Postive	63	63
4.05	8	1.03	1.84	2.5	3.4	6.8	A	Yes	Yes	No	Postive	44	18
3.92	15	1.06	2.15	1.6	2	8.4	A	Yes	Yes	No	Postive	70	70
3.36	9	0.78	1.95	2.4	10.8	39.1	A	Yes	Yes	No	Postive	17	12
4.05	10	1.27	2.29	0.9	8.7	10.9	A	Yes	Yes	No	Postive	27	21
0.22	13	1.79	2.82	2.4	15.1	10.2	A	Yes	No	No	Postive	72	23
4.43	12	1.1	2.5	1.8	25.5	12.4	A	Yes	No	No	Postive	68	53
2.46	33	0.87	1.17	1.3	5.9	10.8	A	Yes	Yes	No	Postive	71	50
3.47	2	1.37	1.86	3.7	小于2	9.8	A	Yes	Yes	No	Postive	70	15
2.62	15	0.75	1.31	2.1	2.3	7.9	A	Yes	Yes	No	Postive	70	70
3.65	8	1.09	1.96	4.6	11.2	4.7	A	No	No	No	Postive	70	70
2.26	24	0.94	1	3.1	19.1	10.4	A	Yes	Yes	No	Postive	34	17
4.22	29	1.32	2.27	2.4	5.8	8.6	A	Yes	Yes	No	Postive	67	67
4.55	21	0.94	2.45	1.5	4.9	7.1	A	Yes	Yes	No	Postive	55	20
4.21	34	1.08	2.28	5.2	16.2	31.4	A	Yes	Yes	No	Postive	66	34
5.1	8	1.74	2.83	1.7	6.9	7.6	A	No	No	No	Postive	64	64
3.47	15	1.07	2.01	3.1	27.9	7.7	A	No	No	No	Postive	63	63
4.18	8	1.47	2.02	5.6	9.8	12	A	Yes	No	Yes	Postive	30	20
4.8	18	1.85	2.42	2.2	11.6	21.1	A	Yes	No	Yes	Postive	20	8
3.78	5	1.24	1.71	2.4	11.7	5.2	A	Yes	No	No	Postive	61	26
3.49	6	1.09	1.74	1	3.6	14.3	A	Yes	Yes	Yes	Postive	64	22
5.58	5	1.17	2.96	1.8	16.3	7.8	A	Yes	No	Yes	Postive	63	1
2.36	19	0.81	1.2	2.1	8.2	4.7	A	Yes	No	Yes	Postive	69	69
3.95	6	1.15	2.35	1.9	2	8.8	A	Yes	No	No	Postive	69	69
4.15	6	1	2.4	3.3	8.4	11	A	Yes	No	No	Postive	64	64
4.72	9	0.76	2.68	2.5	5.3	4.5	A	Yes	No	No	Postive	71	71
3.55	7	0.83	1.83	2.1	75.6	10.3	A	No	No	No	Postive	71	71

2.63	10	1	1.29	2.2	18.2	10.1	A	Yes	Yes	Yes	Postive	15	6
3.32	9	0.75	1.75	3.7	6.1	11.2	A	Yes	Yes	No	Postive	69	69
2.91	7	0.88	1.62	1.5	3.4	10.2	A	Yes	Yes	No	Postive	64	1
3.79	20	1.04	1.94	1.8	101.6	11.2	B	Yes	Yes	No	Postive	16	6
3.45	2	1.32	1.76	1.7	8.6	58.5	A	No	No	No	Postive	55	38
3.48	17	1.04	1.8	2	2.7	25.3	A	Yes	Yes	No	Postive	50	19
2.6	7	0.6	1.42	2.1	6.6	10.5	A	Yes	Yes	No	Postive	71	24
4.19	20	0.85	2.08	4.8	15.3	8.9	A	Yes	Yes	Yes	Postive	3	3
4.42	4	0.74	2.4	1.2	5.8	11.9	A	Yes	Yes	No	Postive	65	50
3.59	30	1.58	1.34	4.2	9.1	24.4	B	Yes	Yes	No	Postive	63	15
4.42	7	0.93	2.57	2.9	6.7	5.7	A	Yes	Yes	Yes	Postive	14	8
3.31	9	1.43	1.39	4.6	15.3	9.2	A	Yes	Yes	No	Postive	72	71
4.21	11	1.03	2.33	2	15.7	17	A	Yes	Yes	No	Postive	72	35
5.14	20	1.68	2.51	2.6	37.5	18.6	A	Yes	Yes	No	Postive	26	20
3.18	19	0.75	1.14	2.2	10.6	56.9	A	Yes	Yes	No	Postive	12	4
3.82	2	1.12	2.03	3	7.5	5.8	A	Yes	Yes	Yes	Postive	26	18
4.39	17	0.89	2.53	1.6	4.9	6.2	A	Yes	Yes	No	Postive	72	25
3.85	10	1.02	2.17	1.7	4.8	8.7	A	Yes	No	No	Postive	72	72
5.5	25	0.96	3.21	1.6	8.7	11.1	A	No	No	No	Postive	19	11
3.14	12	0.75	1.66	2.3	15.6	6.7	A	Yes	No	No	Postive	8	4
2.27	31	0.96	0.79	2.2	19.1	7.9	A	Yes	Yes	No	Postive	55	52
6.37	10	1.18	3.56	1.5	27.8	86.9	A	Yes	Yes	Yes	Postive	64	17
4.93	9	1.38	2.77	1.1	9.7	476.1	B	Yes	Yes	No	Postive	12	6
3.62	18	1.36	1.96	1.3	4.5	7.5	A	Yes	Yes	No	Postive	60	8
2.74	29	0.53	1.35	2.4	20.6	20.6	A	Yes	Yes	No	Postive	30	14
2.9	5	1.07	1.38	2.4	19.7	19.4	A	Yes	Yes	Yes	Postive	61	8
3	21	1.18	1.45	2.5	23.6	30.7	A	Yes	Yes	No	Postive	55	49
3.4	3	0.93	1.88	1.2	2.1	4.3	A	No	No	No	Postive	62	5
3.54	26	1.25	1.76	2.7	11.3	11.7	A	Yes	Yes	No	Postive	20	10
2.74	27	0.71	1.44	5.3	7.8	59.5	A	No	No	Yes	Postive	15	5
3.94	11	0.82	2.19	2.7	9.7	4.4	A	Yes	Yes	No	Postive	40	14
4.4	8	1.37	2.21	1.6	8.4	17.9	A	Yes	Yes	No	Postive	70	3
7.18	14	0.9	1.19	1.6	11	14.8	A	Yes	Yes	No	Postive	13	6
4.3	30	1.56	2.14	3.8	76.4	29.6	A	Yes	Yes	No	Postive	69	69
4.07	6	0.83	2.26	0.7	4.8	19.3	A	Yes	No	No	Postive	53	50
3.39	20	0.46	1.89	1.4	117.9	605.9	B	Yes	Yes	Yes	Postive	25	10
2.46	27	0.63	1.29	2.4	10	136.5	B	Yes	Yes	Yes	Postive	40	11
4.07	6	1.54	2	4.1	9	4.3	A	Yes	No	No	Postive	35	30
2.83	3	0.62	1.46	3	<2	3.6	A	No	No	No	Postive	60	30
4.57	18	1.31	2.17	4.8	8	17.9	A	Yes	Yes	No	Postive	60	29
4.94	5	0.84	2.86	3.9	26.5	6.7	A	Yes	No	No	Postive	44	20
4.28	37	1.84	2.21	4.6	55.6	67.8	A	Yes	Yes	No	Postive	69	35
4.47	17	0.98	2.65	4.1	15.2	12.8	A	Yes	Yes	No	Postive	59	58
3.51	10	1.09	1.85	1.5	65.2	7.7	A	Yes	Yes	No	Postive	10	6
3.81	11	0.95	2.02	3.5	2.9	5.5	A	Yes	No	Yes	Postive	65	14
4.37	3	0.89	2.29	0.9	12.5	20.6	A	No	No	Yes	Postive	29	25
3.1	32	1.37	1.54	3.9	4.3	8.5	B	Yes	Yes	No	Postive	18	14
3.43	40	0.91	1.88	3.4	7.4	4.4	A	No	No	Yes	Postive	67	67

5.44	15	1.08	2.93	2	6.3	5.2	A	Yes	No	Yes	Postive	63	58
3.93	20	1.15	1.97	2.1	22.8	6.1	A	Yes	Yes	No	Postive	54	33
4.92	6	1.3	2.74	1.7	3.2	9.1	A	Yes	No	No	Postive	66	44
3.55	7	0.63	1.97	2.4	11.6	10.8	A	Yes	No	No	Postive	67	28
3.56	11	1	1.93	1.7	23.2	9.2	A	No	No	No	Postive	66	66
2.86	10	1.01	1.32	3.8	2	11.9	A	Yes	No	No	Postive	55	40
2.76	8	0.67	1.36	2.1	874.7	9.5	A	Yes	Yes	No	Postive	61	20
4.02	9	1.29	2.05	0.8	<2	13.2	A	Yes	Yes	Yes	Postive	37	7
4	11	1.21	2.09	5	9.3	18	A	Yes	Yes	No	Postive	36	11
3.99	25	1.48	2.15	6.3	124.3	19.8	A	Yes	Yes	No	Postive	5	3
4.49	19	1.38	2.42	1.3	20.2	27.9	A	Yes	Yes	Yes	Postive	41	27
3.82	7	0.9	2	2	5.3	35.1	A	Yes	No	No	Postive	40	20
4.39	7	1.59	2.48	2.7	33.4	11.9	A	Yes	Yes	No	Postive	40	19
3.95	10	1.13	2.17	9.2	53.8	8.1	A	Yes	No	No	Postive	65	65
3.07	4	1.38	1.45	58.8	6.4	7.1	A	No	No	No	Postive	66	13
5.27	5	1.32	2.89	2	28.4	103.9	A	Yes	Yes	Yes	Postive	60	20
4.43	11	1.22	2.27	5.8	5.5	4	A	No	No	No	Postive	38	32
3.56	28	1.68	1.66	2	11.7	150.9	A	Yes	Yes	Yes	Postive	18	6
3.12	44	1.43	1.33	6.7	3.2	18.4	A	Yes	Yes	No	Postive	35	10
4.29	28	1.64	2.29	1.4	<2	11.1	A	Yes	Yes	Yes	Postive	14	9
3.62	35	1.11	1.87	1.7	5.8	52.8	A	Yes	Yes	No	Postive	63	30
4.31	2	1.17	2.85	1.8	10.8	5.1	A	Yes	No	No	Postive	63	63
3.31	16	1.32	1.66	3.4	6	8	A	Yes	Yes	No	Postive	64	18

supplementary Table S2 Taxon annotation of 1296 OTUs from all samples

OTU_0001	Bacteria;Bacteroidetes;Bacteroidia;Bacteroidales;Prevotellaceae;Prevotella
OTU_0002	Bacteria;Bacteroidetes;Bacteroidia;Bacteroidales;Bacteroidaceae;Bacteroides
OTU_0003	Bacteria;Bacteroidetes;Bacteroidia;Bacteroidales;Bacteroidaceae;Bacteroides
OTU_0004	Bacteria;Proteobacteria;Gammaproteobacteria;Enterobacteriales;Enterobacteriaceae;Escherichia/Shigella
OTU_0005	Bacteria;Firmicutes;Negativicutes;Selenomonadales;Acidaminococcaceae;Phascolarctobacterium
OTU_0006	Bacteria;Firmicutes;Clostridia;Clostridiales;Ruminococcaceae;Faecalibacterium
OTU_0007	Bacteria;Firmicutes;Clostridia;Clostridiales;Lachnospiraceae;Roseburia
OTU_0008	Bacteria;Fusobacteria;Fusobacteria;Fusobacteriales;Fusobacteriaceae;Fusobacterium
OTU_0009	Bacteria;Proteobacteria;Alphaproteobacteria;Rhizobiales;Hyphomicrobiaceae;Gemmiger
OTU_0010	Bacteria;Bacteroidetes;Bacteroidia;Bacteroidales;Bacteroidaceae;Bacteroides
OTU_0011	Bacteria;Bacteroidetes;Bacteroidia;Bacteroidales;Bacteroidaceae;Bacteroides
OTU_0012	Bacteria;Firmicutes;Clostridia;Clostridiales;Ruminococcaceae;Clostridium IV
OTU_0013	Bacteria;Firmicutes;Clostridia;Clostridiales;Lachnospiraceae;Lachnospiracea_incertae_sedis
OTU_0014	Bacteria;Bacteroidetes;Bacteroidia;Bacteroidales;Rikenellaceae;Alistipes
OTU_0015	Bacteria;Firmicutes;Clostridia;Clostridiales;Lachnospiraceae;Roseburia
OTU_0016	Bacteria;Bacteroidetes;Bacteroidia;Bacteroidales;Bacteroidaceae;Bacteroides
OTU_0017	Bacteria;Bacteroidetes;Bacteroidia;Bacteroidales;Prevotellaceae;Prevotella
OTU_0018	Bacteria;Firmicutes
OTU_0019	Bacteria;Firmicutes;Bacilli;Lactobacillales;Streptococcaceae;Streptococcus
OTU_0020	Bacteria;Firmicutes;Clostridia;Clostridiales;Lachnospiraceae;Lachnospiracea_incertae_sedis
OTU_0021	Bacteria;Bacteroidetes;Bacteroidia;Bacteroidales;Bacteroidaceae;Bacteroides
OTU_0022	Bacteria;Firmicutes;Clostridia;Clostridiales;Ruminococcaceae
OTU_0023	Bacteria;Firmicutes;Clostridia;Clostridiales;Lachnospiraceae;Lachnospiracea_incertae_sedis
OTU_0024	Bacteria;Firmicutes;Clostridia;Clostridiales;Lachnospiraceae;Lachnospiracea_incertae_sedis
OTU_0025	Bacteria;Bacteroidetes;Bacteroidia;Bacteroidales;Porphyromonadaceae;Parabacteroides
OTU_0026	Bacteria;Bacteroidetes;Bacteroidia;Bacteroidales;Rikenellaceae;Alistipes
OTU_0027	Bacteria;Firmicutes;Clostridia;Clostridiales;Ruminococcaceae;Ruminococcus
OTU_0028	Bacteria;Firmicutes;Negativicutes;Selenomonadales;Veillonellaceae;Dialister
OTU_0029	Bacteria;Firmicutes;Clostridia;Clostridiales;Ruminococcaceae;Oscillibacter
OTU_0030	Bacteria;Bacteroidetes;Bacteroidia;Bacteroidales;Bacteroidaceae;Bacteroides
OTU_0031	Bacteria;Firmicutes;Clostridia;Clostridiales;Peptostreptococcaceae;Clostridium XI
OTU_0032	Bacteria;Bacteroidetes;Bacteroidia;Bacteroidales;Bacteroidaceae;Bacteroides
OTU_0033	Bacteria;Firmicutes;Clostridia;Clostridiales;Lachnospiraceae;Clostridium XlVa
OTU_0034	Bacteria;Firmicutes;Negativicutes;Selenomonadales;Veillonellaceae;Megamonas
OTU_0035	Bacteria;Actinobacteria;Actinobacteria;Bifidobacteriales;Bifidobacteriaceae;Bifidobacterium
OTU_0036	Bacteria;Firmicutes;Negativicutes;Selenomonadales;Veillonellaceae;Dialister
OTU_0037	Bacteria;Proteobacteria;Betaproteobacteria;Burkholderiales;Sutterellaceae;Parasutterella
OTU_0038	Bacteria;Firmicutes;Erysipelotrichia;Erysipelotrichales;Erysipelotrichaceae;Clostridium XVIII
OTU_0039	Bacteria;Actinobacteria;Actinobacteria;Bifidobacteriales;Bifidobacteriaceae;Bifidobacterium
OTU_0040	Bacteria;Proteobacteria;Gammaproteobacteria;Pasteurellales;Pasteurellaceae;Haemophilus
OTU_0041	Bacteria;Firmicutes;Clostridia;Clostridiales;Clostridiaceae 1;Clostridium sensu stricto
OTU_0042	Bacteria;Firmicutes;Clostridia;Clostridiales;Lachnospiraceae;Clostridium XlVa
OTU_0043	Bacteria;Firmicutes;Clostridia;Clostridiales;Lachnospiraceae;Blautia
OTU_0044	Bacteria;Firmicutes;Clostridia;Clostridiales;Ruminococcaceae
OTU_0045	Bacteria;Proteobacteria;Betaproteobacteria;Burkholderiales;Sutterellaceae;Sutterella
OTU_0046	Bacteria;Firmicutes;Negativicutes;Selenomonadales;Veillonellaceae;Megasphaera
OTU_0047	Bacteria;Firmicutes;Clostridia;Clostridiales;Lachnospiraceae
OTU_0048	Bacteria;Firmicutes
OTU_0049	Bacteria;Verrucomicrobia;Verrucomicrobiae;Verrucomicrobiales;Verrucomicrobiaceae;Akkermansia
OTU_0050	Bacteria;Firmicutes;Negativicutes;Selenomonadales;Veillonellaceae;Veillonella
OTU_0051	Bacteria;Firmicutes;Clostridia;Clostridiales;Ruminococcaceae;Oscillibacter

OTU_0052	Bacteria;Firmicutes;Clostridia;Clostridiales;Lachnospiraceae;Coprococcus
OTU_0053	Bacteria;Bacteroidetes;Bacteroidia;Bacteroidales;Porphyromonadaceae
OTU_0054	Bacteria;Firmicutes;Clostridia;Clostridiales;Ruminococcaceae;Ruminococcus
OTU_0055	Bacteria;Proteobacteria;Deltaproteobacteria;Desulfovibrionales;Desulfovibrionaceae;Bilophila
OTU_0056	Bacteria;Firmicutes;Clostridia;Clostridiales;Ruminococcaceae
OTU_0057	Bacteria;Firmicutes;Clostridia;Clostridiales;Ruminococcaceae
OTU_0058	Bacteria;Firmicutes;Clostridia;Clostridiales;Ruminococcaceae
OTU_0059	Bacteria;Firmicutes;Clostridia;Clostridiales;Ruminococcaceae;Clostridium IV
OTU_0060	Bacteria;Proteobacteria;Betaproteobacteria;Burkholderiales;Sutterellaceae
OTU_0061	Bacteria;Firmicutes;Clostridia;Clostridiales;Ruminococcaceae;Flavonifractor
OTU_0062	Bacteria;Fusobacteria;Fusobacteria;Fusobacteriales;Fusobacteriaceae;Cetobacterium
OTU_0063	Bacteria;Proteobacteria;Gammaproteobacteria;Enterobacteriales;Enterobacteriaceae
OTU_0064	Bacteria;Firmicutes;Clostridia;Clostridiales;Lachnospiraceae;Clostridium XlVa
OTU_0065	Bacteria;Firmicutes;Erysipelotrichia;Erysipelotrichales;Erysipelotrichaceae;Erysipelotrichaceae_incertae_sedis
OTU_0066	Bacteria;Bacteroidetes;Bacteroidia;Bacteroidales;Porphyromonadaceae
OTU_0067	Bacteria;Firmicutes;Negativicutes;Selenomonadales;Acidaminococcaceae;Phascolarctobacterium
OTU_0068	Bacteria;Firmicutes;Clostridia;Clostridiales;Lachnospiraceae;Dorea
OTU_0069	Bacteria;Bacteroidetes;Bacteroidia;Bacteroidales;Prevotellaceae;Prevotella
OTU_0070	Bacteria;Firmicutes;Clostridia;Clostridiales;Lachnospiraceae;Coprococcus
OTU_0071	Bacteria;Bacteroidetes;Bacteroidia;Bacteroidales;Prevotellaceae;Paraprevotella
OTU_0072	Bacteria;Proteobacteria;Gammaproteobacteria;Aeromonadales;Succinivibrionaceae;Succinivibrio
OTU_0073	Bacteria;Bacteroidetes;Bacteroidia;Bacteroidales;Bacteroidaceae;Bacteroides
OTU_0074	Bacteria;Firmicutes;Clostridia;Clostridiales;Ruminococcaceae;Sporobacter
OTU_0075	Bacteria;Bacteroidetes;Bacteroidia;Bacteroidales;Porphyromonadaceae;Parabacteroides
OTU_0076	Bacteria;Bacteroidetes;Bacteroidia;Bacteroidales;Bacteroidaceae;Bacteroides
OTU_0077	Bacteria;Bacteroidetes;Bacteroidia;Bacteroidales;Porphyromonadaceae;Odoribacter
OTU_0078	Bacteria;Proteobacteria;Betaproteobacteria;Burkholderiales;Sutterellaceae;Sutterella
OTU_0079	Bacteria;Firmicutes;Clostridia;Clostridiales;Ruminococcaceae
OTU_0080	Bacteria;Bacteroidetes;Bacteroidia;Bacteroidales;Rikenellaceae;Alistipes
OTU_0081	Bacteria;Spirochaetes;Spirochaetes;Spirochaetales;Spirochaetaceae;Treponema
OTU_0082	Bacteria;Proteobacteria;Deltaproteobacteria;Desulfovibrionales;Desulfovibrionaceae;Desulfovibrio
OTU_0083	Bacteria;Firmicutes;Clostridia;Clostridiales
OTU_0084	Bacteria;Firmicutes;Clostridia;Clostridiales;Ruminococcaceae;Acetanaerobacterium
OTU_0085	Bacteria;Firmicutes;Clostridia;Clostridiales;Lachnospiraceae;Clostridium XlVa
OTU_0086	Bacteria;Firmicutes;Clostridia;Clostridiales;Lachnospiraceae;Lachnospiracea_incertae_sedis
OTU_0087	Bacteria;Actinobacteria;Actinobacteria;Coriobacteriales;Coriobacteriaceae;Collinsella
OTU_0088	Bacteria;Firmicutes;Clostridia;Clostridiales;Lachnospiraceae;Lachnospiracea_incertae_sedis
OTU_0089	Bacteria;Firmicutes;Clostridia;Clostridiales
OTU_0090	Bacteria;Proteobacteria;Alphaproteobacteria;Rhodospirillales;Rhodospirillaceae
OTU_0091	Bacteria;Firmicutes;Clostridia;Clostridiales;Ruminococcaceae;Oscillibacter
OTU_0092	Bacteria;Bacteroidetes;Bacteroidia;Bacteroidales;Rikenellaceae;Alistipes
OTU_0093	Bacteria;Firmicutes;Clostridia;Clostridiales;Ruminococcaceae
OTU_0094	Bacteria;Bacteroidetes;Bacteroidia;Bacteroidales;Prevotellaceae;Prevotella
OTU_0095	Bacteria;Firmicutes;Clostridia;Clostridiales
OTU_0096	Bacteria;Bacteroidetes;Bacteroidia;Bacteroidales;Prevotellaceae;Prevotella
OTU_0097	Bacteria;Firmicutes;Clostridia;Clostridiales;Lachnospiraceae;Blautia
OTU_0098	Bacteria;Firmicutes;Erysipelotrichia;Erysipelotrichales;Erysipelotrichaceae;Catenibacterium
OTU_0099	Bacteria;Firmicutes;Clostridia;Clostridiales
OTU_0100	Bacteria;Firmicutes;Clostridia;Clostridiales;Lachnospiraceae;Anaerostipes
OTU_0101	Bacteria;Firmicutes;Clostridia;Clostridiales;Clostridiaceae 1;Clostridium sensu stricto
OTU_0102	Bacteria;Firmicutes;Clostridia;Clostridiales;Ruminococcaceae
OTU_0103	Bacteria;Firmicutes;Clostridia;Clostridiales;Ruminococcaceae;Ruminococcus

OTU_0104	Bacteria;Firmicutes;Clostridia;Clostridiales;Ruminococcaceae
OTU_0105	Bacteria;Proteobacteria;Betaproteobacteria;Burkholderiales;Sutterellaceae;Sutterella
OTU_0106	Bacteria;Bacteroidetes;Bacteroidia;Bacteroidales;Prevotellaceae;Prevotella
OTU_0107	Bacteria;Firmicutes;Bacilli;Lactobacillales;Streptococcaceae;Streptococcus
OTU_0108	Bacteria;Firmicutes;Bacilli;Lactobacillales;Lactobacillaceae;Lactobacillus
OTU_0109	Bacteria;Bacteroidetes;Bacteroidia;Bacteroidales;Porphyromonadaceae;Barnesiella
OTU_0110	Bacteria;Spirochaetes;Spirochaetes;Spirochaetales;Spirochaetaceae;Treponema
OTU_0111	Bacteria;Proteobacteria;Deltaproteobacteria;Bdellovibrionales;Bdellovibrionaceae;Vampirovibrio
OTU_0112	Bacteria;Firmicutes;Erysipelotrichia;Erysipelotrichales;Erysipelotrichaceae
OTU_0113	Bacteria;Firmicutes;Clostridia;Clostridiales;Ruminococcaceae;Ruminococcus
OTU_0114	Bacteria;Proteobacteria;Alphaproteobacteria;Rhodospirillales;Rhodospirillaceae
OTU_0115	Bacteria;Firmicutes;Negativicutes;Selenomonadales;Veillonellaceae;Dialister
OTU_0116	Bacteria;Firmicutes;Clostridia;Clostridiales
OTU_0117	Bacteria;Firmicutes;Clostridia;Clostridiales;Lachnospiraceae;Clostridium XlVb
OTU_0118	Bacteria;Firmicutes;Negativicutes;Selenomonadales;Acidaminococcaceae
OTU_0119	Bacteria;Firmicutes;Clostridia;Clostridiales;Peptostreptococcaceae;Clostridium XI
OTU_0120	Bacteria;Firmicutes;Erysipelotrichia;Erysipelotrichales;Erysipelotrichaceae;Clostridium XVIII
OTU_0121	Bacteria;Firmicutes;Clostridia;Clostridiales;Lachnospiraceae;Clostridium XlVb
OTU_0122	Bacteria;Proteobacteria;Betaproteobacteria;Burkholderiales;Sutterellaceae;Sutterella
OTU_0123	Bacteria;Firmicutes;Clostridia;Clostridiales;Lachnospiraceae;Clostridium XlVa
OTU_0124	Bacteria;Bacteroidetes;Bacteroidia;Bacteroidales;Rikenellaceae;Alistipes
OTU_0125	Bacteria;Firmicutes;Clostridia;Clostridiales;Lachnospiraceae
OTU_0126	Bacteria;Firmicutes;Clostridia;Clostridiales;Ruminococcaceae;Clostridium IV
OTU_0127	Bacteria;Firmicutes;Clostridia;Clostridiales;Lachnospiraceae;Lachnospiracea_incertae_sedis
OTU_0128	Bacteria;Firmicutes;Clostridia;Clostridiales;Ruminococcaceae;Butyricicoccus
OTU_0129	Bacteria;Firmicutes;Clostridia;Clostridiales;Lachnospiraceae;Lachnospiracea_incertae_sedis
OTU_0130	Bacteria;Firmicutes;Clostridia;Clostridiales;Ruminococcaceae;Clostridium IV
OTU_0131	Bacteria;Firmicutes;Clostridia
OTU_0132	Bacteria;Firmicutes;Clostridia;Clostridiales;Lachnospiraceae;Clostridium XlVb
OTU_0133	Bacteria;Actinobacteria;Actinobacteria;Coriobacteriales;Coriobacteriaceae;Slackia
OTU_0134	Bacteria;Firmicutes;Clostridia;Clostridiales;Lachnospiraceae;Lachnospiracea_incertae_sedis
OTU_0135	Bacteria;Firmicutes;Clostridia;Clostridiales
OTU_0136	Bacteria;Firmicutes;Bacilli;Lactobacillales;Lactobacillaceae;Lactobacillus
OTU_0137	Bacteria;Firmicutes;Clostridia;Clostridiales;Ruminococcaceae;Butyricicoccus
OTU_0138	Bacteria;Firmicutes;Clostridia;Clostridiales
OTU_0139	Bacteria;Bacteroidetes;Bacteroidia;Bacteroidales;Porphyromonadaceae;Butyricimonas
OTU_0140	Bacteria;Bacteroidetes;Bacteroidia;Bacteroidales;Porphyromonadaceae;Dysgonomonas
OTU_0141	Bacteria;Firmicutes;Clostridia;Clostridiales
OTU_0142	Bacteria;Firmicutes;Clostridia;Clostridiales;Lachnospiraceae
OTU_0143	Bacteria;Bacteroidetes;Bacteroidia;Bacteroidales;Prevotellaceae;Prevotella
OTU_0144	Bacteria;Bacteroidetes;Bacteroidia;Bacteroidales;Prevotellaceae;Prevotella
OTU_0145	Bacteria;Proteobacteria;Betaproteobacteria;Burkholderiales;Sutterellaceae;Sutterella
OTU_0146	Bacteria;Tenericutes;Mollicutes;Anaeroplasmatales;Anaeroplasmataceae;Asteroleplasma
OTU_0147	Bacteria;Proteobacteria;Gammaproteobacteria;Enterobacteriales;Enterobacteriaceae;Citrobacter
OTU_0148	Bacteria;Firmicutes
OTU_0149	Bacteria;Firmicutes;Clostridia;Clostridiales;Ruminococcaceae;Clostridium IV
OTU_0150	Bacteria;Proteobacteria;Alphaproteobacteria;Rhizobiales;Hyphomicrobiaceae;Gemmiger
OTU_0151	Bacteria;Firmicutes;Clostridia;Clostridiales;Ruminococcaceae;Flavonifractor
OTU_0152	Bacteria;Firmicutes;Clostridia;Clostridiales;Ruminococcaceae
OTU_0153	Bacteria;Bacteroidetes;Bacteroidia;Bacteroidales;Rikenellaceae;Alistipes
OTU_0154	Bacteria;Firmicutes;Clostridia;Clostridiales;Ruminococcaceae;Ruminococcus
OTU_0155	Bacteria;Bacteroidetes;Bacteroidia;Bacteroidales;Bacteroidaceae;Bacteroides

OTU_0156	Bacteria;Firmicutes;Bacilli;Lactobacillales;Lactobacillaceae;Lactobacillus
OTU_0157	Bacteria;Firmicutes;Erysipelotrichia;Erysipelotrichales;Erysipelotrichaceae
OTU_0158	Bacteria;Bacteroidetes;Bacteroidia;Bacteroidales;Prevotellaceae;Paraprevotella
OTU_0159	Bacteria;Firmicutes;Clostridia;Clostridiales;Ruminococcaceae;Ruminococcus
OTU_0160	Bacteria;Bacteroidetes;Bacteroidia;Bacteroidales;Porphyromonadaceae;Barnesiella
OTU_0161	Bacteria;Proteobacteria;Deltaproteobacteria;Desulfovibrionales;Desulfovibrionaceae
OTU_0162	Bacteria;Bacteroidetes;Bacteroidia;Bacteroidales;Rikenellaceae;Alistipes
OTU_0163	Bacteria;Actinobacteria;Actinobacteria;Coriobacteriales;Coriobacteriaceae
OTU_0164	Bacteria;Actinobacteria;Actinobacteria;Coriobacteriales;Coriobacteriaceae;Enterorhabdus
OTU_0165	Bacteria;Firmicutes;Negativicutes;Selenomonadales;Acidaminococcaceae;Phascolarctobacterium
OTU_0166	Bacteria;Actinobacteria;Actinobacteria;Coriobacteriales;Coriobacteriaceae;Olsenella
OTU_0167	Bacteria;Firmicutes;Clostridia;Clostridiales
OTU_0168	Bacteria;Firmicutes;Erysipelotrichia;Erysipelotrichales;Erysipelotrichaceae
OTU_0169	Bacteria;Bacteroidetes;Bacteroidia;Bacteroidales;Prevotellaceae;Hallella
OTU_0170	Bacteria;TM7;TM7_genera_incertae_sedis;TM7_genera_incertae_sedis;TM7_genera_incertae_sedis;TM7_genera_incertae_sedis
OTU_0171	Bacteria;Firmicutes;Clostridia;Clostridiales;Lachnospiraceae;Lachnospiracea_incertae_sedis
OTU_0172	Bacteria;Bacteroidetes;Bacteroidia;Bacteroidales;Porphyromonadaceae;Parabacteroides
OTU_0173	Bacteria;Proteobacteria;Deltaproteobacteria;Bdellovibrionales;Bdellovibrionaceae;Vampirovibrio
OTU_0174	Bacteria;Firmicutes;Clostridia;Clostridiales;Lachnospiraceae;Clostridium XlVa
OTU_0175	Bacteria;Proteobacteria;Betaproteobacteria;Burkholderiales;Sutterellaceae;Sutterella
OTU_0176	Bacteria;Bacteroidetes;Bacteroidia;Bacteroidales;Porphyromonadaceae;Butyricimonas
OTU_0177	Bacteria;Firmicutes;Clostridia;Clostridiales;Ruminococcaceae;Ruminococcus
OTU_0178	Bacteria;Bacteroidetes;Bacteroidia;Bacteroidales;Prevotellaceae;Prevotella
OTU_0179	Bacteria;Bacteroidetes;Bacteroidia;Bacteroidales;Bacteroidaceae;Bacteroides
OTU_0180	Bacteria;Bacteroidetes;Bacteroidia;Bacteroidales;Prevotellaceae;Prevotella
OTU_0181	Bacteria;Bacteroidetes;Bacteroidia;Bacteroidales;Prevotellaceae;Prevotella
OTU_0182	Bacteria;Firmicutes;Clostridia;Clostridiales
OTU_0183	Bacteria;Firmicutes;Clostridia;Clostridiales
OTU_0184	Bacteria;Firmicutes;Erysipelotrichia;Erysipelotrichales;Erysipelotrichaceae;Erysipelotrichaceae_incertae_sedis
OTU_0185	Bacteria;Firmicutes;Clostridia;Clostridiales
OTU_0186	Bacteria;Firmicutes;Clostridia;Clostridiales;Ruminococcaceae
OTU_0187	Bacteria;Firmicutes;Clostridia;Clostridiales;Ruminococcaceae
OTU_0188	Bacteria;Proteobacteria;Epsilonproteobacteria;Campylobacterales;Campylobacteraceae;Campylobacter
OTU_0189	Bacteria;Proteobacteria;Deltaproteobacteria;Bdellovibrionales;Bdellovibrionaceae;Vampirovibrio
OTU_0190	Bacteria;Firmicutes;Bacilli;Lactobacillales;Lactobacillaceae;Lactobacillus
OTU_0191	Bacteria;Firmicutes
OTU_0192	Bacteria;Bacteroidetes;Bacteroidia;Bacteroidales;Prevotellaceae;Prevotella
OTU_0193	Bacteria;Firmicutes;Clostridia;Clostridiales;Ruminococcaceae;Clostridium IV
OTU_0194	Bacteria;Firmicutes;Bacilli;Lactobacillales;Leuconostocaceae;Weissella
OTU_0195	Bacteria;Firmicutes;Clostridia;Clostridiales
OTU_0196	Bacteria;Bacteroidetes;Bacteroidia;Bacteroidales;Prevotellaceae;Prevotella
OTU_0197	Bacteria;Bacteroidetes;Bacteroidia;Bacteroidales;Rikenellaceae;Alistipes
OTU_0198	Bacteria;Firmicutes;Clostridia;Clostridiales;Ruminococcaceae;Oscillibacter
OTU_0199	Bacteria;Firmicutes;Clostridia;Clostridiales;Lachnospiraceae;Clostridium XlVa
OTU_0200	Bacteria;Firmicutes;Clostridia;Clostridiales;Lachnospiraceae;Lachnospiracea_incertae_sedis
OTU_0201	Bacteria;Firmicutes;Clostridia;Clostridiales;Ruminococcaceae;Flavonifractor
OTU_0202	Bacteria;Bacteroidetes;Bacteroidia;Bacteroidales;Porphyromonadaceae
OTU_0203	Bacteria;Firmicutes;Erysipelotrichia;Erysipelotrichales;Erysipelotrichaceae;Turicibacter
OTU_0204	Bacteria;Bacteroidetes
OTU_0205	Bacteria
OTU_0206	Bacteria

OTU_0207	Bacteria;Firmicutes;Clostridia;Clostridiales;Lachnospiraceae;Coprococcus
OTU_0208	Bacteria;Fusobacteria;Fusobacteria;Fusobacteriales;Fusobacteriaceae
OTU_0209	Bacteria;Actinobacteria;Actinobacteria;Bifidobacteriales;Bifidobacteriaceae;Bifidobacterium
OTU_0210	Bacteria;Firmicutes;Clostridia;Clostridiales;Ruminococcaceae
OTU_0211	Bacteria;Firmicutes;Clostridia
OTU_0212	Bacteria;Firmicutes;Clostridia;Clostridiales;Ruminococcaceae;Clostridium IV
OTU_0213	Bacteria;Proteobacteria;Alphaproteobacteria;Rhodospirillales;Rhodospirillaceae
OTU_0214	Bacteria;Firmicutes;Clostridia;Clostridiales;Lachnospiraceae
OTU_0215	Bacteria;Firmicutes;Erysipelotrichia;Erysipelotrichales;Erysipelotrichaceae;Erysipelotrichaceae_incertae_sedis
OTU_0216	Bacteria;Actinobacteria;Actinobacteria;Coriobacteriales;Coriobacteriaceae
OTU_0217	Bacteria;Bacteroidetes;Bacteroidia;Bacteroidales;Bacteroidaceae;Bacteroides
OTU_0218	Bacteria;Firmicutes;Erysipelotrichia;Erysipelotrichales;Erysipelotrichaceae;Holdemania
OTU_0219	Bacteria;Firmicutes;Negativicutes;Selenomonadales;Veillonellaceae;Megasphaera
OTU_0220	Bacteria;Firmicutes;Clostridia;Clostridiales;Lachnospiraceae
OTU_0221	Bacteria;Firmicutes;Clostridia;Clostridiales;Lachnospiraceae;Howardella
OTU_0222	Bacteria;Firmicutes;Bacilli;Lactobacillales;Enterococcaceae;Enterococcus
OTU_0223	Bacteria;Firmicutes;Clostridia;Clostridiales;Ruminococcaceae
OTU_0224	Bacteria;Firmicutes;Erysipelotrichia;Erysipelotrichales;Erysipelotrichaceae;Allobaculum
OTU_0225	Bacteria;Firmicutes;Clostridia;Clostridiales;Ruminococcaceae
OTU_0226	Bacteria;Firmicutes;Erysipelotrichia;Erysipelotrichales;Erysipelotrichaceae;Erysipelotrichaceae_incertae_sedis
OTU_0227	Bacteria;Spirochaetes;Spirochaetes;Spirochaetales;Spirochaetaceae;Treponema
OTU_0228	Bacteria;Firmicutes;Clostridia;Clostridiales;Clostridiaceae 1;Clostridium sensu stricto
OTU_0229	Bacteria;Firmicutes;Clostridia;Clostridiales;Ruminococcaceae
OTU_0230	Archaea;Euryarchaeota;Methanobacteria;Methanobacteriales;Methanobacteriaceae;Methanobrevibacter
OTU_0231	Bacteria;Firmicutes;Clostridia;Clostridiales;Ruminococcaceae
OTU_0232	Bacteria;Bacteroidetes;Bacteroidia;Bacteroidales;Bacteroidaceae;Bacteroides
OTU_0233	Bacteria;Firmicutes;Clostridia;Clostridiales;Ruminococcaceae;Clostridium IV
OTU_0234	Bacteria;Bacteroidetes;Bacteroidia;Bacteroidales;Rikenellaceae;Alistipes
OTU_0235	Bacteria;Firmicutes;Clostridia;Clostridiales;Ruminococcaceae;Clostridium IV
OTU_0236	Bacteria;Actinobacteria;Actinobacteria;Coriobacteriales;Coriobacteriaceae;Collinsella
OTU_0237	Bacteria;Firmicutes;Erysipelotrichia;Erysipelotrichales;Erysipelotrichaceae;Coprobacillus
OTU_0238	Bacteria;Tenericutes;Mollicutes;Anaeroplasmatales;Anaeroplasmataceae;Anaeroplasma
OTU_0239	Bacteria;Firmicutes;Clostridia;Clostridiales
OTU_0240	Bacteria;Proteobacteria;Deltaproteobacteria;Bdellovibrionales;Bdellovibrionaceae;Vampirovibrio
OTU_0241	Bacteria;Firmicutes;Negativicutes;Selenomonadales;Veillonellaceae;Veillonella
OTU_0242	Bacteria;Bacteroidetes;Bacteroidia;Bacteroidales;Bacteroidaceae;Bacteroides
OTU_0243	Bacteria;Bacteroidetes;Bacteroidia;Bacteroidales;Bacteroidaceae;Bacteroides
OTU_0244	Bacteria;Actinobacteria;Actinobacteria;Coriobacteriales;Coriobacteriaceae;Eggerthella
OTU_0245	Bacteria;Firmicutes;Clostridia;Clostridiales
OTU_0246	Bacteria;Firmicutes;Clostridia;Clostridiales
OTU_0247	Bacteria;Firmicutes;Bacilli;Lactobacillales;Aerococcaceae;Abiotrophia
OTU_0248	Bacteria;Bacteroidetes;Bacteroidia;Bacteroidales;Bacteroidaceae;Bacteroides
OTU_0249	Bacteria;Bacteroidetes;Bacteroidia;Bacteroidales
OTU_0250	Bacteria;Firmicutes;Clostridia;Clostridiales;Ruminococcaceae
OTU_0251	Bacteria;Firmicutes;Negativicutes;Selenomonadales;Veillonellaceae;Dialister
OTU_0252	Bacteria;Firmicutes;Bacilli;Bacillales;Bacillales_Incertae Sedis XI;Gemella
OTU_0253	Bacteria;Firmicutes;Clostridia;Clostridiales;Lachnospiraceae
OTU_0254	Bacteria;Firmicutes;Bacilli;Lactobacillales;Carnobacteriaceae;Granulicatella
OTU_0255	Bacteria;Firmicutes;Clostridia;Clostridiales;Ruminococcaceae;Faecalibacterium
OTU_0256	Bacteria;Bacteroidetes;Bacteroidia;Bacteroidales;Bacteroidaceae;Bacteroides
OTU_0257	Bacteria;Firmicutes;Clostridia;Clostridiales;Ruminococcaceae;Anaerotruncus
OTU_0258	Bacteria;Firmicutes;Negativicutes;Selenomonadales;Veillonellaceae;Mitsuokella

OTU_0259	Bacteria;Bacteroidetes;Bacteroidia;Bacteroidales;Marinilabiaceae;Anaerophaga
OTU_0260	Bacteria;Bacteroidetes;Bacteroidia;Bacteroidales;Prevotellaceae;Paraprevotella
OTU_0261	Bacteria;Lentisphaerae;Lentisphaeria;Victivallales;Victivallaceae;Victivallis
OTU_0262	Bacteria;Firmicutes;Clostridia;Clostridiales;Lachnospiraceae;Clostridium XlVa
OTU_0263	Bacteria;Firmicutes;Clostridia;Clostridiales
OTU_0264	Bacteria;Proteobacteria;Gammaproteobacteria;Enterobacteriales;Enterobacteriaceae;Proteus
OTU_0265	Bacteria;Firmicutes
OTU_0266	Bacteria;Proteobacteria;Gammaproteobacteria;Enterobacteriales;Enterobacteriaceae;Hafnia
OTU_0267	Bacteria;Firmicutes;Negativicutes;Selenomonadales;Veillonellaceae;Dialister
OTU_0268	Bacteria;Proteobacteria;Betaproteobacteria;Burkholderiales;Sutterellaceae;Parasutterella
OTU_0269	Bacteria;Firmicutes;Clostridia;Clostridiales;Ruminococcaceae
OTU_0270	Bacteria;Bacteroidetes;Bacteroidia;Bacteroidales;Bacteroidaceae;Bacteroides
OTU_0271	Bacteria;Firmicutes;Clostridia;Clostridiales;Ruminococcaceae;Acetivibrio
OTU_0272	Bacteria;Firmicutes;Clostridia;Clostridiales;Ruminococcaceae;Ruminococcus
OTU_0273	Bacteria;Firmicutes;Erysipelotrichia;Erysipelotrichales;Erysipelotrichaceae;Solobacterium
OTU_0274	Bacteria;Firmicutes;Clostridia;Clostridiales
OTU_0275	Bacteria;Firmicutes;Clostridia;Clostridiales
OTU_0276	Bacteria;Proteobacteria;Epsilonproteobacteria;Campylobacterales;Campylobacteraceae;Campylobacter
OTU_0277	Bacteria;Firmicutes;Clostridia;Clostridiales
OTU_0278	Bacteria;Firmicutes;Negativicutes;Selenomonadales;Veillonellaceae;Veillonella
OTU_0279	Bacteria;Firmicutes;Clostridia;Clostridiales;Ruminococcaceae
OTU_0280	Bacteria;Actinobacteria;Actinobacteria;Coriobacteriales;Coriobacteriaceae
OTU_0281	Bacteria;Proteobacteria;Gammaproteobacteria;Enterobacteriales;Enterobacteriaceae;Providencia
OTU_0282	Bacteria;Firmicutes;Clostridia;Clostridiales;Lachnospiraceae
OTU_0283	Bacteria;Firmicutes;Clostridia;Clostridiales;Ruminococcaceae;Pseudoflavonifractor
OTU_0284	Bacteria;Firmicutes;Erysipelotrichia;Erysipelotrichales;Erysipelotrichaceae;Coprobacillus
OTU_0285	Bacteria;TM7;TM7_genera_incertae_sedis;TM7_genera_incertae_sedis;TM7_genera_incertae_sedis;TM7_genera_incer tae_sedis
OTU_0286	Bacteria;Bacteroidetes;Bacteroidia;Bacteroidales;Porphyromonadaceae;Butyricimonas
OTU_0287	Bacteria;Firmicutes;Clostridia;Clostridiales
OTU_0288	Bacteria;Firmicutes;Erysipelotrichia;Erysipelotrichales;Erysipelotrichaceae
OTU_0289	Bacteria;Proteobacteria;Alphaproteobacteria;Rhodospirillales;Rhodospirillaceae
OTU_0290	Bacteria;Firmicutes;Clostridia;Clostridiales;Ruminococcaceae
OTU_0291	Bacteria;Firmicutes;Clostridia;Clostridiales
OTU_0292	Bacteria;Firmicutes;Clostridia;Clostridiales;Ruminococcaceae;Pseudoflavonifractor
OTU_0293	Bacteria;Firmicutes;Bacilli;Lactobacillales;Lactobacillaceae;Lactobacillus
OTU_0294	Bacteria;Firmicutes;Erysipelotrichia;Erysipelotrichales;Erysipelotrichaceae
OTU_0295	Bacteria;Firmicutes;Clostridia;Clostridiales
OTU_0296	Bacteria;Bacteroidetes;Bacteroidia;Bacteroidales;Porphyromonadaceae;Barnesiella
OTU_0297	Bacteria;Bacteroidetes;Bacteroidia;Bacteroidales;Porphyromonadaceae;Butyricimonas
OTU_0298	Bacteria;Firmicutes;Clostridia;Clostridiales;Ruminococcaceae;Ruminococcus
OTU_0299	Bacteria;Firmicutes;Clostridia;Clostridiales;Lachnospiraceae
OTU_0300	Bacteria;Bacteroidetes;Bacteroidia;Bacteroidales;Prevotellaceae;Prevotella
OTU_0301	Bacteria;Actinobacteria;Actinobacteria;Coriobacteriales;Coriobacteriaceae
OTU_0302	Bacteria;Actinobacteria;Actinobacteria;Coriobacteriales;Coriobacteriaceae;Atopobium
OTU_0303	Bacteria;Bacteroidetes;Bacteroidia;Bacteroidales;Porphyromonadaceae;Butyricimonas
OTU_0304	Bacteria;Bacteroidetes;Bacteroidia;Bacteroidales;Bacteroidaceae;Bacteroides
OTU_0305	Bacteria;Firmicutes;Negativicutes;Selenomonadales;Veillonellaceae;Megasphaera
OTU_0306	Bacteria;Firmicutes;Negativicutes;Selenomonadales;Veillonellaceae;Megasphaera
OTU_0307	Bacteria;Proteobacteria;Alphaproteobacteria;Rhizobiales;Hyphomicrobiaceae;Gemmiger
OTU_0308	Bacteria;Firmicutes;Clostridia;Clostridiales;Ruminococcaceae;Oscillibacter
OTU_0309	Bacteria;Firmicutes;Clostridia;Clostridiales;Lachnospiraceae;Clostridium XlVb

OTU_0310	Bacteria;Firmicutes;Clostridia;Clostridiales;Ruminococcaceae
OTU_0311	Bacteria;Firmicutes;Clostridia;Clostridiales;Eubacteriaceae;Eubacterium
OTU_0312	Bacteria;Lentisphaerae;Lentisphaeria;Victivallales;Victivallaceae;Victivallis
OTU_0313	Bacteria;Bacteroidetes;Bacteroidia;Bacteroidales;Porphyromonadaceae;Parabacteroides
OTU_0314	Bacteria;Bacteroidetes;Bacteroidia;Bacteroidales;Prevotellaceae;Prevotella
OTU_0315	Bacteria;Firmicutes;Clostridia;Clostridiales
OTU_0316	Bacteria;Firmicutes;Clostridia;Clostridiales;Ruminococcaceae;Anaerotruncus
OTU_0317	Bacteria;Actinobacteria;Actinobacteria;Actinomycetales;Actinomycetaceae;Actinomyces
OTU_0318	Bacteria;Firmicutes;Erysipelotrichia;Erysipelotrichales;Erysipelotrichaceae;Clostridium XVIII
OTU_0319	Bacteria;Bacteroidetes;Bacteroidia;Bacteroidales;Prevotellaceae;Prevotella
OTU_0320	Bacteria;Actinobacteria;Actinobacteria;Coriobacteriales;Coriobacteriaceae;Collinsella
OTU_0321	Bacteria;Firmicutes;Clostridia;Clostridiales;Ruminococcaceae
OTU_0322	Bacteria;Firmicutes
OTU_0323	Bacteria;Firmicutes;Clostridia;Clostridiales;Ruminococcaceae;Clostridium III
OTU_0324	Bacteria;Firmicutes;Negativicutes;Selenomonadales;Acidaminococcaceae;Acidaminococcus
OTU_0325	Bacteria;Bacteroidetes;Bacteroidia;Bacteroidales;Prevotellaceae;Prevotella
OTU_0326	Bacteria;Bacteroidetes;Bacteroidia;Bacteroidales;Bacteroidaceae;Bacteroides
OTU_0327	Bacteria;Firmicutes;Clostridia;Clostridiales;Ruminococcaceae
OTU_0328	Bacteria;Bacteroidetes;Bacteroidia;Bacteroidales;Porphyromonadaceae
OTU_0329	Bacteria;Firmicutes;Negativicutes;Selenomonadales;Veillonellaceae;Megasphaera
OTU_0330	Bacteria;Firmicutes;Bacilli;Lactobacillales;Lactobacillaceae;Lactobacillus
OTU_0331	Bacteria;Firmicutes;Negativicutes;Selenomonadales;Veillonellaceae;Dialister
OTU_0332	Bacteria;Firmicutes;Clostridia;Clostridiales
OTU_0333	Bacteria
OTU_0334	Bacteria;Firmicutes;Negativicutes;Selenomonadales;Veillonellaceae;Allisonella
OTU_0335	Bacteria;Proteobacteria;Alphaproteobacteria
OTU_0336	Bacteria;Firmicutes;Erysipelotrichia;Erysipelotrichales;Erysipelotrichaceae;Erysipelotrichaceae_incertae_sedis
OTU_0337	Bacteria;Firmicutes;Clostridia;Clostridiales
OTU_0338	Bacteria;Proteobacteria;Alphaproteobacteria;Rhodospirillales;Rhodospirillaceae
OTU_0339	Bacteria;Bacteroidetes;Bacteroidia;Bacteroidales;Bacteroidaceae;Bacteroides
OTU_0340	Bacteria;Firmicutes;Clostridia;Clostridiales;Ruminococcaceae;Papillibacter
OTU_0341	Bacteria;Firmicutes;Erysipelotrichia;Erysipelotrichales;Erysipelotrichaceae;Holdemania
OTU_0342	Bacteria;Bacteroidetes;Bacteroidia;Bacteroidales;Bacteroidaceae;Bacteroides
OTU_0343	Bacteria;Actinobacteria;Actinobacteria;Coriobacteriales;Coriobacteriaceae;Olsenella
OTU_0344	Bacteria;Firmicutes;Clostridia;Clostridiales;Clostridiales_Incertae Sedis XIII
OTU_0345	Bacteria;Bacteroidetes;Bacteroidia;Bacteroidales;Prevotellaceae
OTU_0346	Bacteria;Firmicutes;Bacilli;Lactobacillales;Streptococcaceae;Streptococcus
OTU_0347	Bacteria;Firmicutes;Clostridia;Clostridiales;Ruminococcaceae;Anaerotruncus
OTU_0348	Bacteria;Firmicutes;Clostridia;Clostridiales
OTU_0349	Bacteria;Firmicutes;Clostridia;Clostridiales;Clostridiales_Incertae Sedis XIII;Mogibacterium
OTU_0350	Bacteria;Bacteroidetes;Bacteroidia;Bacteroidales;Porphyromonadaceae
OTU_0351	Bacteria;Firmicutes;Clostridia;Clostridiales;Ruminococcaceae
OTU_0352	Bacteria;Firmicutes;Clostridia;Clostridiales;Ruminococcaceae
OTU_0353	Bacteria;Firmicutes;Clostridia;Clostridiales
OTU_0354	Bacteria;Bacteroidetes;Bacteroidia;Bacteroidales;Prevotellaceae;Paraprevotella
OTU_0355	Bacteria;Bacteroidetes;Bacteroidia;Bacteroidales;Porphyromonadaceae;Barnesiella
OTU_0356	Bacteria;Firmicutes;Clostridia;Clostridiales;Lachnospiraceae;Clostridium XlVa
OTU_0357	Bacteria;Actinobacteria;Actinobacteria;Coriobacteriales;Coriobacteriaceae;Adlercreutzia
OTU_0358	Bacteria;Proteobacteria;Deltaproteobacteria
OTU_0359	Bacteria
OTU_0360	Bacteria;Actinobacteria;Actinobacteria;Bifidobacteriales;Bifidobacteriaceae;Bifidobacterium
OTU_0361	Bacteria;Bacteroidetes;Bacteroidia;Bacteroidales;Prevotellaceae

OTU_0362	Bacteria
OTU_0363	Bacteria;Firmicutes;Clostridia;Clostridiales
OTU_0364	Bacteria;Bacteroidetes;Bacteroidia;Bacteroidales;Porphyromonadaceae;Barnesiella
OTU_0365	Bacteria;Proteobacteria;Betaproteobacteria;Burkholderiales;Comamonadaceae;Comamonas
OTU_0366	Bacteria;Proteobacteria;Deltaproteobacteria;Desulfovibrionales;Desulfomicrobiaceae;Desulfomicrobium
OTU_0367	Bacteria;Verrucomicrobia;Verrucomicrobiae;Verrucomicrobiales;Verrucomicrobiaceae;Akkermansia
OTU_0368	Bacteria;Firmicutes;Clostridia;Clostridiales
OTU_0369	Unclassified
OTU_0370	Bacteria;Firmicutes;Clostridia;Clostridiales;Ruminococcaceae
OTU_0371	Bacteria;Lentisphaerae;Lentisphaeria;Victivallales;Victivallaceae;Victivallis
OTU_0372	Bacteria;Firmicutes;Negativicutes;Selenomonadales;Acidaminococcaceae;Acidaminococcus
OTU_0373	Bacteria;Fusobacteria;Fusobacteria;Fusobacteriales;Fusobacteriaceae;Fusobacterium
OTU_0374	Bacteria;Bacteroidetes;Bacteroidia;Bacteroidales;Prevotellaceae;Prevotella
OTU_0375	Bacteria;Elusimicrobia;Elusimicrobia;Elusimicrobiales;Elusimicrobiaceae;Elusimicrobium
OTU_0376	Bacteria;Deinococcus-Thermus;Deinococci;Thermales;Thermaceae;Thermus
OTU_0377	Bacteria;Firmicutes;Clostridia;Clostridiales;Lachnospiraceae;Lachnospiracea_incertae_sedis
OTU_0378	Bacteria;Firmicutes
OTU_0379	Bacteria;Firmicutes;Clostridia;Clostridiales;Peptococcaceae 1;Peptococcus
OTU_0380	Bacteria;Bacteroidetes;Bacteroidia;Bacteroidales;Prevotellaceae;Prevotella
OTU_0381	Bacteria;Firmicutes
OTU_0382	Bacteria;Bacteroidetes;Bacteroidia;Bacteroidales;Prevotellaceae;Paraprevotella
OTU_0383	Bacteria;Firmicutes;Clostridia;Clostridiales;Ruminococcaceae;Clostridium IV
OTU_0384	Bacteria;Synergistetes;Synergistia;Synergistales;Synergistaceae;Pyramidobacter
OTU_0385	Bacteria;Firmicutes;Clostridia;Clostridiales;Ruminococcaceae
OTU_0386	Bacteria;Firmicutes
OTU_0387	Bacteria;Firmicutes;Clostridia;Clostridiales;Ruminococcaceae;Acetivibrio
OTU_0388	Bacteria;Bacteroidetes;Bacteroidia;Bacteroidales;Bacteroidaceae;Bacteroides
OTU_0389	Bacteria;Firmicutes;Bacilli;Lactobacillales;Lactobacillaceae;Lactobacillus
OTU_0390	Bacteria;Actinobacteria;Actinobacteria;Coriobacteriales;Coriobacteriaceae
OTU_0391	Bacteria;Actinobacteria;Actinobacteria;Coriobacteriales;Coriobacteriaceae
OTU_0392	Bacteria;Firmicutes;Clostridia;Clostridiales;Lachnospiraceae;Pseudobutyrivibrio
OTU_0393	Bacteria;Bacteroidetes;Bacteroidia;Bacteroidales;Porphyromonadaceae;Butyricimonas
OTU_0394	Bacteria;Lentisphaerae;Lentisphaeria;Victivallales;Victivallaceae;Victivallis
OTU_0395	Bacteria;Firmicutes;Clostridia;Clostridiales;Lachnospiraceae
OTU_0396	Bacteria;Firmicutes;Clostridia;Clostridiales
OTU_0397	Bacteria;Firmicutes;Clostridia;Clostridiales;Ruminococcaceae
OTU_0398	Bacteria;Firmicutes;Clostridia;Clostridiales;Ruminococcaceae;Butyricicoccus
OTU_0399	Bacteria;Bacteroidetes;Bacteroidia;Bacteroidales;Porphyromonadaceae
OTU_0400	Bacteria;Firmicutes;Clostridia;Clostridiales;Ruminococcaceae
OTU_0401	Bacteria;Firmicutes;Erysipelotrichia;Erysipelotrichales;Erysipelotrichaceae;Bulleidia
OTU_0402	Bacteria;Firmicutes;Clostridia;Clostridiales;Lachnospiraceae;Clostridium XlVb
OTU_0403	Bacteria;Firmicutes;Clostridia;Clostridiales;Lachnospiraceae;Lachnospiracea_incertae_sedis
OTU_0404	Bacteria;Bacteroidetes;Bacteroidia;Bacteroidales;Bacteroidaceae;Anaerorhabdus
OTU_0405	Bacteria;Firmicutes;Clostridia
OTU_0406	Bacteria;Firmicutes;Clostridia;Clostridiales;Lachnospiraceae;Clostridium XlVa
OTU_0407	Bacteria;Bacteroidetes;Bacteroidia;Bacteroidales;Porphyromonadaceae;Barnesiella
OTU_0408	Bacteria;Bacteroidetes
OTU_0409	Bacteria;Bacteroidetes;Bacteroidia;Bacteroidales;Bacteroidaceae;Bacteroides
OTU_0410	Bacteria;Firmicutes
OTU_0411	Bacteria;Proteobacteria;Gammaproteobacteria;Enterobacteriales;Enterobacteriaceae
OTU_0412	Bacteria;Proteobacteria;Betaproteobacteria;Burkholderiales;Oxalobacteraceae
OTU_0413	Bacteria;Bacteroidetes;Bacteroidia;Bacteroidales;Prevotellaceae;Prevotella

OTU_0414	Bacteria;Bacteroidetes;Bacteroidia;Bacteroidales;Bacteroidaceae;Bacteroides
OTU_0415	Bacteria;Proteobacteria;Betaproteobacteria;Burkholderiales;Sutterellaceae;Parasutterella
OTU_0416	Bacteria;Bacteroidetes;Bacteroidia;Bacteroidales;Prevotellaceae;Prevotella
OTU_0417	Bacteria;Firmicutes;Erysipelotrichia;Erysipelotrichales;Erysipelotrichaceae;Erysipelotrichaceae_incertae_sedis
OTU_0418	Bacteria;Firmicutes;Clostridia;Clostridiales;Ruminococcaceae;Flavonifractor
OTU_0419	Bacteria;Firmicutes;Clostridia;Clostridiales
OTU_0420	Bacteria;Firmicutes;Clostridia;Clostridiales;Lachnospiraceae;Cellulosilyticum
OTU_0421	Bacteria;Actinobacteria;Actinobacteria;Coriobacteriales;Coriobacteriaceae;Gordonibacter
OTU_0422	Bacteria;Firmicutes;Clostridia;Clostridiales
OTU_0423	Bacteria;Firmicutes;Clostridia;Clostridiales
OTU_0424	Bacteria;Firmicutes;Clostridia;Clostridiales;Ruminococcaceae;Sporobacter
OTU_0425	Bacteria;Proteobacteria;Gammaproteobacteria;Pasteurellales;Pasteurellaceae;Haemophilus
OTU_0426	Bacteria;Firmicutes;Clostridia;Clostridiales;Peptostreptococcaceae;Clostridium XI
OTU_0427	Bacteria;Proteobacteria;Gammaproteobacteria;Pasteurellales;Pasteurellaceae;Aggregatibacter
OTU_0428	Bacteria;Firmicutes;Bacilli;Lactobacillales;Streptococcaceae;Streptococcus
OTU_0429	Bacteria;Firmicutes;Clostridia;Clostridiales
OTU_0430	Bacteria;Bacteroidetes;Bacteroidia;Bacteroidales;Prevotellaceae;Prevotella
OTU_0431	Bacteria;Firmicutes;Bacilli;Lactobacillales;Lactobacillaceae;Lactobacillus
OTU_0432	Bacteria;TM7;TM7_genera_incertae_sedis;TM7_genera_incertae_sedis;TM7_genera_incertae_sedis;TM7_genera_incertae_sedis
OTU_0433	Bacteria
OTU_0434	Bacteria;Firmicutes
OTU_0435	Bacteria;Proteobacteria;Gammaproteobacteria;Pseudomonadales;Pseudomonadaceae;Pseudomonas
OTU_0436	Bacteria;Firmicutes;Clostridia;Clostridiales;Lachnospiraceae;Lachnospiracea_incertae_sedis
OTU_0437	Bacteria;Firmicutes;Negativicutes;Selenomonadales;Veillonellaceae;Dialister
OTU_0438	Bacteria;Actinobacteria;Actinobacteria;Coriobacteriales;Coriobacteriaceae;Gordonibacter
OTU_0439	Bacteria;TM7;TM7_genera_incertae_sedis;TM7_genera_incertae_sedis;TM7_genera_incertae_sedis;TM7_genera_incertae_sedis
OTU_0440	Bacteria;Bacteroidetes;Bacteroidia;Bacteroidales;Porphyromonadaceae;Parabacteroides
OTU_0441	Bacteria;Cyanobacteria/Chloroplast;Chloroplast;Chloroplast;Chloroplast;Streptophyta
OTU_0442	Bacteria;Firmicutes;Clostridia;Clostridiales;Ruminococcaceae
OTU_0443	Bacteria;Firmicutes;Clostridia;Clostridiales;Ruminococcaceae
OTU_0444	Bacteria;Actinobacteria;Actinobacteria;Coriobacteriales;Coriobacteriaceae
OTU_0445	Bacteria;Firmicutes;Clostridia;Clostridiales;Lachnospiraceae;Anaerostipes
OTU_0446	Bacteria;Proteobacteria;Epsilonproteobacteria;Campylobacterales;Campylobacteraceae;Campylobacter
OTU_0447	Bacteria;Firmicutes;Clostridia;Clostridiales;Clostridiales_Incertae Sedis XIII;Mogibacterium
OTU_0448	Bacteria;Firmicutes;Clostridia;Clostridiales
OTU_0449	Bacteria;Proteobacteria;Betaproteobacteria;Burkholderiales
OTU_0450	Bacteria;Proteobacteria;Alphaproteobacteria
OTU_0451	Bacteria;Actinobacteria;Actinobacteria;Actinomycetales;Micrococcaceae;Rothia
OTU_0452	Bacteria;Bacteroidetes;Bacteroidia;Bacteroidales;Porphyromonadaceae;Tannerella
OTU_0453	Bacteria;Firmicutes;Bacilli;Lactobacillales;Lactobacillaceae;Lactobacillus
OTU_0454	Bacteria;Proteobacteria;Alphaproteobacteria;Rhodospirillales;Rhodospirillaceae
OTU_0455	Bacteria;Firmicutes;Bacilli;Lactobacillales;Enterococcaceae;Vagococcus
OTU_0456	Bacteria;Bacteroidetes;Bacteroidia;Bacteroidales;Prevotellaceae;Prevotella
OTU_0457	Bacteria;Actinobacteria;Actinobacteria;Coriobacteriales;Coriobacteriaceae;Collinsella
OTU_0458	Bacteria;Bacteroidetes;Bacteroidia;Bacteroidales;Prevotellaceae;Prevotella
OTU_0459	Bacteria;Firmicutes;Clostridia;Clostridiales;Ruminococcaceae;Acetivibrio
OTU_0460	Bacteria;Actinobacteria;Actinobacteria;Coriobacteriales;Coriobacteriaceae;Olsenella
OTU_0461	Bacteria;Firmicutes;Bacilli;Lactobacillales;Leuconostocaceae;Leuconostoc
OTU_0462	Bacteria;Firmicutes;Clostridia;Clostridiales;Ruminococcaceae;Flavonifractor
OTU_0463	Bacteria;Bacteroidetes;Bacteroidia;Bacteroidales;Bacteroidaceae;Bacteroides

OTU_0464	Bacteria;Firmicutes;Clostridia;Clostridiales;Lachnospiraceae;Clostridium XlVa
OTU_0465	Bacteria;Bacteroidetes;Sphingobacteria;Sphingobacteriales;Chitinophagaceae;Sediminibacterium
OTU_0466	Bacteria;Firmicutes;Clostridia;Clostridiales
OTU_0467	Bacteria;Firmicutes
OTU_0468	Bacteria;Firmicutes;Erysipelotrichia;Erysipelotrichales;Erysipelotrichaceae
OTU_0469	Bacteria;Bacteroidetes;Bacteroidia;Bacteroidales;Porphyromonadaceae;Parabacteroides
OTU_0470	Bacteria;Firmicutes;Negativicutes;Selenomonadales;Veillonellaceae;Mitsuokella
OTU_0471	Bacteria;Firmicutes;Clostridia;Clostridiales;Ruminococcaceae;Clostridium IV
OTU_0472	Bacteria;Firmicutes;Clostridia;Clostridiales;Eubacteriaceae;Eubacterium
OTU_0473	Bacteria;Firmicutes;Bacilli;Lactobacillales;Streptococcaceae;Streptococcus
OTU_0474	Bacteria;Firmicutes;Erysipelotrichia;Erysipelotrichales;Erysipelotrichaceae
OTU_0475	Bacteria;Firmicutes;Clostridia;Clostridiales;Lachnospiraceae
OTU_0476	Bacteria;Firmicutes;Clostridia;Clostridiales;Lachnospiraceae;Blautia
OTU_0477	Bacteria;Bacteroidetes;Bacteroidia;Bacteroidales;Bacteroidaceae;Bacteroides
OTU_0478	Bacteria;Firmicutes;Negativicutes;Selenomonadales;Acidaminococcaceae;Phascolarctobacterium
OTU_0479	Bacteria;Bacteroidetes;Bacteroidia;Bacteroidales;Bacteroidaceae;Bacteroides
OTU_0480	Bacteria;Firmicutes;Clostridia;Clostridiales;Ruminococcaceae;Clostridium IV
OTU_0481	Bacteria;Firmicutes;Negativicutes;Selenomonadales;Veillonellaceae;Anaeroglobus
OTU_0482	Bacteria;Firmicutes;Clostridia;Clostridiales;Ruminococcaceae;Clostridium IV
OTU_0483	Bacteria;Proteobacteria;Gammaproteobacteria;Xanthomonadales;Xanthomonadaceae;Stenotrophomonas
OTU_0484	Bacteria;Bacteroidetes;Flavobacteria;Flavobacteriales
OTU_0485	Bacteria;Bacteroidetes;Bacteroidia;Bacteroidales;Prevotellaceae;Hallella
OTU_0486	Bacteria;Firmicutes;Clostridia;Clostridiales;Lachnospiraceae;Clostridium XlVb
OTU_0487	Bacteria;Bacteroidetes;Bacteroidia;Bacteroidales;Porphyromonadaceae;Parabacteroides
OTU_0488	Bacteria;Bacteroidetes
OTU_0489	Bacteria
OTU_0490	Bacteria;Firmicutes;Erysipelotrichia;Erysipelotrichales;Erysipelotrichaceae
OTU_0491	Bacteria;Proteobacteria;Gammaproteobacteria;Pseudomonadales;Moraxellaceae;Acinetobacter
OTU_0492	Bacteria;Firmicutes;Bacilli;Lactobacillales;Streptococcaceae;Lactococcus
OTU_0493	Bacteria;Firmicutes;Clostridia;Clostridiales
OTU_0494	Bacteria;Firmicutes;Bacilli;Lactobacillales;Streptococcaceae;Streptococcus
OTU_0495	Bacteria;Firmicutes;Clostridia;Clostridiales;Ruminococcaceae;Flavonifractor
OTU_0496	Bacteria
OTU_0497	Bacteria;Firmicutes;Clostridia;Clostridiales;Ruminococcaceae;Oscillibacter
OTU_0498	Bacteria;Firmicutes;Clostridia;Clostridiales;Ruminococcaceae
OTU_0499	Bacteria;Firmicutes;Clostridia;Clostridiales;Lachnospiraceae;Parasporobacterium
OTU_0500	Bacteria;Proteobacteria;Gammaproteobacteria;Pasteurellales;Pasteurellaceae;Aggregatibacter
OTU_0501	Bacteria;Bacteroidetes;Bacteroidia;Bacteroidales;Prevotellaceae
OTU_0502	Bacteria;Firmicutes
OTU_0503	Bacteria;Bacteroidetes;Flavobacteria;Flavobacteriales;Flavobacteriaceae;Chryseobacterium
OTU_0504	Bacteria;Firmicutes;Clostridia;Clostridiales;Ruminococcaceae;Clostridium IV
OTU_0505	Bacteria;Firmicutes;Clostridia;Clostridiales;Ruminococcaceae
OTU_0506	Bacteria;Firmicutes;Clostridia;Clostridiales;Ruminococcaceae;Flavonifractor
OTU_0507	Bacteria;Proteobacteria;Deltaproteobacteria;Desulfovibrionales;Desulfovibrionaceae
OTU_0508	Bacteria;Firmicutes;Clostridia;Clostridiales
OTU_0509	Bacteria;Firmicutes;Clostridia;Clostridiales;Peptococcaceae 1;Peptococcus
OTU_0510	Bacteria;Firmicutes;Clostridia;Clostridiales;Ruminococcaceae;Faecalibacterium
OTU_0511	Bacteria;Bacteroidetes;Bacteroidia;Bacteroidales;Bacteroidaceae;Bacteroides
OTU_0512	Bacteria;Firmicutes;Negativicutes;Selenomonadales;Veillonellaceae;Megasphaera
OTU_0513	Bacteria;Firmicutes;Clostridia;Clostridiales;Ruminococcaceae;Anaerotruncus
OTU_0514	Bacteria;Firmicutes
OTU_0515	Bacteria;Firmicutes;Clostridia;Clostridiales;Ruminococcaceae;Ruminococcus

OTU_0516	Bacteria;Proteobacteria;Gammaproteobacteria;Vibrionales;Vibrionaceae;Vibrio
OTU_0517	Bacteria;Proteobacteria;Betaproteobacteria;Burkholderiales;Sutterellaceae;Parasutterella
OTU_0518	Bacteria;Firmicutes;Clostridia;Clostridiales;Ruminococcaceae
OTU_0519	Bacteria;Firmicutes;Clostridia;Clostridiales;Clostridiales_Incertae Sedis XI;Parvimonas
OTU_0520	Bacteria;Bacteroidetes;Bacteroidia;Bacteroidales;Prevotellaceae;Prevotella
OTU_0521	Bacteria;Firmicutes;Clostridia;Clostridiales;Lachnospiraceae;Anaerostipes
OTU_0522	Bacteria;Firmicutes;Clostridia;Clostridiales;Ruminococcaceae;Anaerofilum
OTU_0523	Bacteria;Proteobacteria;Alphaproteobacteria;Rhodospirillales;Rhodospirillaceae
OTU_0524	Bacteria;Firmicutes;Clostridia;Clostridiales
OTU_0525	Bacteria;Firmicutes;Clostridia;Clostridiales
OTU_0526	Bacteria;Firmicutes;Clostridia;Clostridiales;Ruminococcaceae;Clostridium IV
OTU_0527	Bacteria
OTU_0528	Bacteria;Firmicutes;Clostridia;Clostridiales;Ruminococcaceae;Clostridium IV
OTU_0529	Bacteria;Proteobacteria;Gammaproteobacteria;Aeromonadales;Aeromonadaceae;Aeromonas
OTU_0530	Bacteria;Verrucomicrobia;Opitutae;Puniceicoccales;Puniceicoccaceae;Cerasicoccus
OTU_0531	Bacteria;Bacteroidetes;Bacteroidia;Bacteroidales;Prevotellaceae;Prevotella
OTU_0532	Bacteria;Bacteroidetes;Bacteroidia;Bacteroidales;Porphyromonadaceae
OTU_0533	Bacteria;Firmicutes;Erysipelotrichia;Erysipelotrichales;Erysipelotrichaceae
OTU_0534	Bacteria;Firmicutes;Clostridia;Clostridiales
OTU_0535	Bacteria;Bacteroidetes;Bacteroidia;Bacteroidales;Rikenellaceae;Alistipes
OTU_0536	Bacteria
OTU_0537	Bacteria;Firmicutes;Clostridia;Clostridiales
OTU_0538	Bacteria;Firmicutes;Clostridia;Clostridiales;Eubacteriaceae;Eubacterium
OTU_0539	Bacteria;Bacteroidetes;Bacteroidia;Bacteroidales;Porphyromonadaceae;Barnesiella
OTU_0540	Bacteria;Firmicutes;Clostridia;Clostridiales;Ruminococcaceae
OTU_0541	Bacteria;Firmicutes
OTU_0542	Bacteria;Bacteroidetes;Bacteroidia;Bacteroidales;Rikenellaceae;Alistipes
OTU_0543	Bacteria;Bacteroidetes;Bacteroidia;Bacteroidales;Prevotellaceae;Paraprevotella
OTU_0544	Bacteria;Firmicutes;Clostridia;Clostridiales;Ruminococcaceae;Oscillibacter
OTU_0545	Bacteria;Firmicutes;Bacilli;Lactobacillales;Lactobacillaceae;Lactobacillus
OTU_0546	Bacteria;Bacteroidetes;Bacteroidia;Bacteroidales;Prevotellaceae;Prevotella
OTU_0547	Bacteria;Proteobacteria;Deltaproteobacteria
OTU_0548	Bacteria;Firmicutes;Negativicutes;Selenomonadales;Veillonellaceae;Dialister
OTU_0549	Bacteria;Firmicutes
OTU_0550	Bacteria;Firmicutes;Clostridia;Clostridiales;Clostridiaceae 1;Clostridium sensu stricto
OTU_0551	Bacteria;Bacteroidetes;Bacteroidia;Bacteroidales;Porphyromonadaceae;Butyricimonas
OTU_0552	Bacteria;Bacteroidetes;Bacteroidia;Bacteroidales;Bacteroidaceae;Bacteroides
OTU_0553	Bacteria;Firmicutes;Clostridia;Clostridiales;Ruminococcaceae;Ruminococcus
OTU_0554	Bacteria;Bacteroidetes;Bacteroidia;Bacteroidales;Prevotellaceae;Prevotella
OTU_0555	Bacteria;Firmicutes;Clostridia;Clostridiales;Ruminococcaceae
OTU_0556	Bacteria;Firmicutes;Clostridia;Clostridiales;Ruminococcaceae;Clostridium IV
OTU_0557	Bacteria;Bacteroidetes;Bacteroidia;Bacteroidales;Porphyromonadaceae;Barnesiella
OTU_0558	Bacteria;Firmicutes;Clostridia;Clostridiales;Lachnospiraceae;Butyrivibrio
OTU_0559	Bacteria;Firmicutes;Negativicutes;Selenomonadales;Veillonellaceae;Veillonella
OTU_0560	Bacteria;Actinobacteria;Actinobacteria;Coriobacteriales;Coriobacteriaceae;Gordonibacter
OTU_0561	Bacteria;Firmicutes;Clostridia;Clostridiales;Peptococcaceae 1;Peptococcus
OTU_0562	Bacteria;Firmicutes;Clostridia;Clostridiales;Ruminococcaceae
OTU_0563	Bacteria;Firmicutes;Clostridia;Clostridiales;Clostridiales_Incertae Sedis XI
OTU_0564	Bacteria;Firmicutes;Clostridia;Clostridiales;Ruminococcaceae;Clostridium IV
OTU_0565	Bacteria;Firmicutes;Erysipelotrichia;Erysipelotrichales;Erysipelotrichaceae;Turicibacter
OTU_0566	Bacteria;Firmicutes;Clostridia;Clostridiales
OTU_0567	Bacteria;Proteobacteria;Deltaproteobacteria;Desulfovibrionales;Desulfovibrionaceae;Desulfovibrio

OTU_0568	Bacteria;Firmicutes;Clostridia;Clostridiales;Ruminococcaceae;Clostridium IV
OTU_0569	Bacteria;Firmicutes;Clostridia;Clostridiales;Ruminococcaceae
OTU_0570	Bacteria;Proteobacteria;Gammaproteobacteria;Enterobacteriales;Enterobacteriaceae;Plesiomonas
OTU_0571	Bacteria;Bacteroidetes;Bacteroidia;Bacteroidales;Prevotellaceae;Prevotella
OTU_0572	Bacteria;Firmicutes;Clostridia;Clostridiales;Ruminococcaceae
OTU_0573	Bacteria;Firmicutes;Clostridia;Clostridiales
OTU_0574	Bacteria;Firmicutes
OTU_0575	Bacteria;Bacteroidetes;Bacteroidia;Bacteroidales;Bacteroidaceae;Bacteroides
OTU_0576	Bacteria;Firmicutes;Clostridia;Clostridiales;Ruminococcaceae
OTU_0577	Bacteria;Firmicutes
OTU_0578	Bacteria;Firmicutes;Clostridia;Clostridiales;Ruminococcaceae;Anaerofilum
OTU_0579	Bacteria;Verrucomicrobia;Opitutae;Puniceicoccales;Puniceicoccaceae
OTU_0580	Bacteria;Firmicutes;Clostridia;Clostridiales
OTU_0581	Bacteria;Firmicutes;Clostridia;Clostridiales;Ruminococcaceae
OTU_0582	Bacteria;Proteobacteria;Deltaproteobacteria;Desulfovibrionales;Desulfovibrionaceae
OTU_0583	Bacteria;Lentisphaerae;Lentisphaeria;Victivallales;Victivallaceae;Victivallis
OTU_0584	Bacteria;Verrucomicrobia;Opitutae;Puniceicoccales;Puniceicoccaceae;Cerasicoccus
OTU_0585	Bacteria;Bacteroidetes;Bacteroidia;Bacteroidales;Bacteroidaceae;Bacteroides
OTU_0586	Bacteria;Firmicutes;Clostridia;Clostridiales
OTU_0587	Bacteria;Firmicutes;Clostridia;Clostridiales;Ruminococcaceae;Clostridium IV
OTU_0588	Bacteria;Firmicutes;Erysipelotrichia;Erysipelotrichales;Erysipelotrichaceae
OTU_0589	Bacteria;Proteobacteria;Betaproteobacteria;Neisseriales;Neisseriaceae;Neisseria
OTU_0590	Bacteria;Firmicutes;Erysipelotrichia;Erysipelotrichales;Erysipelotrichaceae;Erysipelotrichaceae_incertae_sedis
OTU_0591	Bacteria;Firmicutes;Clostridia;Clostridiales;Lachnospiraceae;Cellulosilyticum
OTU_0592	Bacteria;Proteobacteria;Gammaproteobacteria;Aeromonadales;Succinivibrionaceae;Succinivibrio
OTU_0593	Bacteria;Firmicutes;Clostridia
OTU_0594	Bacteria;Firmicutes;Clostridia;Clostridiales;Ruminococcaceae;Clostridium III
OTU_0595	Bacteria;Bacteroidetes;Bacteroidia;Bacteroidales;Porphyromonadaceae;Barnesiella
OTU_0596	Bacteria;Firmicutes;Clostridia;Clostridiales;Ruminococcaceae;Clostridium IV
OTU_0597	Bacteria;Firmicutes;Negativicutes;Selenomonadales;Acidaminococcaceae;Acidaminococcus
OTU_0598	Bacteria;Fusobacteria;Fusobacteria;Fusobacteriales;Leptotrichiaceae;Leptotrichia
OTU_0599	Bacteria;Bacteroidetes;Bacteroidia;Bacteroidales;Porphyromonadaceae;Barnesiella
OTU_0600	Bacteria;Firmicutes;Clostridia;Clostridiales;Lachnospiraceae;Oribacterium
OTU_0601	Bacteria;Actinobacteria;Actinobacteria;Bifidobacteriales;Bifidobacteriaceae;Scardovia
OTU_0602	Bacteria;Firmicutes;Clostridia;Clostridiales
OTU_0603	Bacteria;Fusobacteria;Fusobacteria;Fusobacteriales;Leptotrichiaceae;Leptotrichia
OTU_0604	Bacteria
OTU_0605	Bacteria;Bacteroidetes;Bacteroidia;Bacteroidales;Marinilabiaceae;Anaerophaga
OTU_0606	Bacteria;Firmicutes;Clostridia;Clostridiales;Ruminococcaceae
OTU_0607	Bacteria;Firmicutes;Clostridia;Clostridiales;Lachnospiraceae;Clostridium XlVa
OTU_0608	Bacteria;Proteobacteria;Betaproteobacteria;Burkholderiales;Sutterellaceae
OTU_0609	Bacteria;Synergistetes;Synergistia;Synergistales;Synergistaceae;Pyramidobacter
OTU_0610	Bacteria;Firmicutes;Clostridia;Clostridiales
OTU_0611	Bacteria;Bacteroidetes;Bacteroidia;Bacteroidales;Bacteroidaceae;Bacteroides
OTU_0612	Bacteria;Firmicutes;Clostridia;Clostridiales
OTU_0613	Bacteria;Firmicutes;Clostridia;Clostridiales;Ruminococcaceae;Clostridium IV
OTU_0614	Bacteria;Firmicutes;Clostridia;Clostridiales;Ruminococcaceae
OTU_0615	Bacteria;Firmicutes;Clostridia;Clostridiales;Lachnospiraceae;Lachnospiracea_incertae_sedis
OTU_0616	Bacteria;Firmicutes;Clostridia;Clostridiales;Lachnospiraceae;Lachnospiracea_incertae_sedis
OTU_0617	Bacteria;Firmicutes;Clostridia;Clostridiales;Eubacteriaceae;Eubacterium
OTU_0618	Bacteria;Bacteroidetes;Flavobacteria;Flavobacteriales
OTU_0619	Bacteria;Firmicutes;Clostridia;Clostridiales;Ruminococcaceae;Clostridium IV

OTU_0620	Bacteria;Firmicutes;Erysipelotrichia;Erysipelotrichales;Erysipelotrichaceae
OTU_0621	Bacteria;Bacteroidetes;Bacteroidia;Bacteroidales;Porphyromonadaceae;Barnesiella
OTU_0622	Bacteria;Bacteroidetes;Flavobacteria;Flavobacteriales;Flavobacteriaceae
OTU_0623	Bacteria;Synergistetes;Synergistia;Synergistales;Synergistaceae;Cloacibacillus
OTU_0624	Bacteria;Bacteroidetes;Bacteroidia;Bacteroidales;Rikenellaceae;Alistipes
OTU_0625	Bacteria;Firmicutes;Erysipelotrichia;Erysipelotrichales;Erysipelotrichaceae;Clostridium XVIII
OTU_0626	Bacteria;Proteobacteria;Betaproteobacteria;Burkholderiales;Sutterellaceae;Parasutterella
OTU_0627	Bacteria;Firmicutes;Clostridia;Clostridiales
OTU_0628	Bacteria;Bacteroidetes;Bacteroidia;Bacteroidales;Porphyromonadaceae;Odoribacter
OTU_0629	Bacteria;Firmicutes
OTU_0630	Bacteria;Proteobacteria;Betaproteobacteria;Burkholderiales;Oxalobacteraceae;Janthinobacterium
OTU_0631	Bacteria;Verrucomicrobia;Verrucomicrobiae;Verrucomicrobiales;Verrucomicrobiaceae;Akkermansia
OTU_0632	Bacteria;Firmicutes;Clostridia;Clostridiales
OTU_0633	Bacteria;Proteobacteria;Alphaproteobacteria;Rhizobiales;Rhizobiaceae;Rhizobium
OTU_0634	Bacteria;Firmicutes;Clostridia;Clostridiales;Ruminococcaceae;Butyricicoccus
OTU_0635	Bacteria;TM7;TM7_genera_incertae_sedis;TM7_genera_incertae_sedis;TM7_genera_incertae_sedis;TM7_genera_incertae_sedis
OTU_0636	Bacteria;Firmicutes;Clostridia;Clostridiales;Clostridiales_Incertae Sedis XI;Finegoldia
OTU_0637	Bacteria;Synergistetes;Synergistia;Synergistales;Synergistaceae;Cloacibacillus
OTU_0638	Bacteria;Firmicutes;Clostridia;Clostridiales;Ruminococcaceae;Hydrogenoanaerobacterium
OTU_0639	Bacteria
OTU_0640	Bacteria;Firmicutes;Bacilli;Lactobacillales;Streptococcaceae;Lactococcus
OTU_0641	Bacteria;Verrucomicrobia;Opitutae;Puniceicoccales;Puniceicoccaceae;Cerasicoccus
OTU_0642	Bacteria;Firmicutes;Clostridia;Clostridiales;Eubacteriaceae;Eubacterium
OTU_0643	Bacteria;Proteobacteria;Epsilonproteobacteria;Campylobacterales;Campylobacteraceae;Campylobacter
OTU_0644	Bacteria;Firmicutes;Clostridia;Clostridiales;Ruminococcaceae;Acetivibrio
OTU_0645	Bacteria;Bacteroidetes;Bacteroidia;Bacteroidales;Porphyromonadaceae;Barnesiella
OTU_0646	Bacteria;Firmicutes
OTU_0647	Bacteria;Firmicutes;Clostridia;Clostridiales
OTU_0648	Bacteria;Firmicutes;Clostridia;Clostridiales;Ruminococcaceae;Sporobacter
OTU_0649	Bacteria;Firmicutes;Clostridia;Clostridiales;Ruminococcaceae
OTU_0650	Bacteria
OTU_0651	Bacteria;Firmicutes;Clostridia;Clostridiales;Ruminococcaceae
OTU_0652	Bacteria;Actinobacteria;Actinobacteria;Coriobacteriales;Coriobacteriaceae;Coriobacterium
OTU_0653	Bacteria;Bacteroidetes;Bacteroidia;Bacteroidales;Prevotellaceae;Prevotella
OTU_0654	Bacteria;Firmicutes;Clostridia;Clostridiales;Clostridiales_Incertae Sedis XI;Peptoniphilus
OTU_0655	Bacteria;Firmicutes;Clostridia;Clostridiales
OTU_0656	Bacteria;Firmicutes;Clostridia;Clostridiales;Lachnospiraceae;Oribacterium
OTU_0657	Bacteria;Firmicutes;Clostridia;Clostridiales;Ruminococcaceae
OTU_0658	Bacteria;Actinobacteria;Actinobacteria;Coriobacteriales;Coriobacteriaceae;Olsenella
OTU_0659	Bacteria;Proteobacteria;Gammaproteobacteria;Xanthomonadales;Xanthomonadaceae;Lysobacter
OTU_0660	Bacteria;Firmicutes;Clostridia;Clostridiales
OTU_0661	Bacteria;Bacteroidetes;Bacteroidia;Bacteroidales;Porphyromonadaceae;Porphyromonas
OTU_0662	Bacteria;Actinobacteria;Actinobacteria;Actinomycetales;Corynebacteriaceae;Corynebacterium
OTU_0663	Bacteria;Firmicutes;Clostridia;Clostridiales;Ruminococcaceae
OTU_0664	Bacteria;Proteobacteria;Alphaproteobacteria;Caulobacterales;Caulobacteraceae;Brevundimonas
OTU_0665	Bacteria;Actinobacteria;Actinobacteria;Coriobacteriales;Coriobacteriaceae
OTU_0666	Bacteria;Bacteroidetes;Bacteroidia;Bacteroidales;Prevotellaceae;Prevotella
OTU_0667	Bacteria;Proteobacteria;Alphaproteobacteria;Rhodospirillales;Rhodospirillaceae
OTU_0668	Bacteria;Proteobacteria;Alphaproteobacteria;Rhizobiales;Bradyrhizobiaceae
OTU_0669	Bacteria;Firmicutes;Clostridia;Clostridiales;Ruminococcaceae;Clostridium IV
OTU_0670	Bacteria;Firmicutes;Clostridia;Clostridiales;Ruminococcaceae

OTU_0671	Bacteria;Firmicutes;Bacilli;Bacillales;Staphylococcaceae;Staphylococcus
OTU_0672	Bacteria;Firmicutes;Clostridia;Clostridiales;Lachnospiraceae
OTU_0673	Bacteria;Firmicutes;Clostridia;Clostridiales;Ruminococcaceae;Saccharofermentans
OTU_0674	Bacteria;Firmicutes;Clostridia;Clostridiales;Ruminococcaceae;Clostridium IV
OTU_0675	Bacteria;Firmicutes;Clostridia;Clostridiales;Ruminococcaceae;Pseudoflavonifractor
OTU_0676	Bacteria;Bacteroidetes
OTU_0677	Bacteria;Proteobacteria;Epsilonproteobacteria;Campylobacterales;Campylobacteraceae;Campylobacter
OTU_0678	Bacteria;Firmicutes;Clostridia;Clostridiales
OTU_0679	Bacteria;Firmicutes;Clostridia;Clostridiales
OTU_0680	Bacteria;Firmicutes;Clostridia;Clostridiales;Ruminococcaceae;Ruminococcus
OTU_0681	Bacteria;Fusobacteria;Fusobacteria;Fusobacteriales;Fusobacteriaceae;Fusobacterium
OTU_0682	Bacteria;Proteobacteria;Gammaproteobacteria;Pseudomonadales;Moraxellaceae;Enhydrobacter
OTU_0683	Bacteria;Firmicutes;Negativicutes;Selenomonadales;Veillonellaceae;Selenomonas
OTU_0684	Bacteria;Firmicutes;Negativicutes;Selenomonadales;Veillonellaceae;Mitsuokella
OTU_0685	Bacteria;Firmicutes;Clostridia;Clostridiales;Ruminococcaceae
OTU_0686	Bacteria;Firmicutes;Clostridia;Clostridiales
OTU_0687	Bacteria;Firmicutes;Clostridia;Clostridiales;Lachnospiraceae;Howardella
OTU_0688	Bacteria;Firmicutes;Clostridia;Clostridiales;Ruminococcaceae;Hydrogenoanaerobacterium
OTU_0689	Bacteria;Firmicutes;Clostridia;Clostridiales;Ruminococcaceae;Ruminococcus
OTU_0690	Bacteria;Bacteroidetes;Bacteroidia;Bacteroidales;Bacteroidaceae;Bacteroides
OTU_0691	Bacteria;Proteobacteria;Gammaproteobacteria;Pasteurellales;Pasteurellaceae;Haemophilus
OTU_0692	Bacteria;Actinobacteria;Actinobacteria;Bifidobacteriales;Bifidobacteriaceae;Alloscardovia
OTU_0693	Bacteria;Proteobacteria;Epsilonproteobacteria;Campylobacterales;Helicobacteraceae;Helicobacter
OTU_0694	Bacteria;Firmicutes;Clostridia;Clostridiales
OTU_0695	Bacteria;Bacteroidetes;Bacteroidia;Bacteroidales;Prevotellaceae;Prevotella
OTU_0696	Bacteria;Firmicutes;Erysipelotrichia;Erysipelotrichales;Erysipelotrichaceae;Erysipelotrichaceae_incertae_sedis
OTU_0697	Bacteria;Firmicutes;Clostridia;Clostridiales
OTU_0698	Bacteria;Actinobacteria;Actinobacteria;Coriobacteriales;Coriobacteriaceae;Slackia
OTU_0699	Bacteria;Bacteroidetes;Bacteroidia;Bacteroidales;Prevotellaceae;Prevotella
OTU_0700	Bacteria;Firmicutes;Clostridia;Clostridiales;Ruminococcaceae;Acetivibrio
OTU_0701	Bacteria;Bacteroidetes;Bacteroidia;Bacteroidales;Prevotellaceae;Prevotella
OTU_0702	Bacteria;Bacteroidetes;Bacteroidia;Bacteroidales;Prevotellaceae;Prevotella
OTU_0703	Bacteria;Verrucomicrobia;Opitutae;Puniceicoccales;Puniceicoccaceae;Coraliomargarita
OTU_0704	Bacteria;Firmicutes;Clostridia;Clostridiales
OTU_0705	Bacteria;Firmicutes;Clostridia;Clostridiales;Clostridiales_Incertae Sedis XI;Peptoniphilus
OTU_0706	Bacteria;Firmicutes;Clostridia;Clostridiales;Ruminococcaceae
OTU_0707	Bacteria
OTU_0708	Bacteria;Firmicutes;Clostridia;Clostridiales;Peptostreptococcaceae;Peptostreptococcus
OTU_0709	Bacteria;Firmicutes;Clostridia;Clostridiales
OTU_0710	Bacteria;Firmicutes;Clostridia;Clostridiales;Ruminococcaceae
OTU_0711	Unclassified
OTU_0712	Bacteria;Proteobacteria;Alphaproteobacteria;Rhizobiales;Brucellaceae;Brucella
OTU_0713	Bacteria;Firmicutes;Clostridia;Clostridiales;Eubacteriaceae;Eubacterium
OTU_0714	Bacteria;Firmicutes;Negativicutes;Selenomonadales;Veillonellaceae;Centipeda
OTU_0715	Bacteria;Proteobacteria;Deltaproteobacteria;Desulfovibrionales;Desulfovibrionaceae;Desulfovibrio
OTU_0716	Bacteria;Firmicutes;Clostridia;Clostridiales
OTU_0717	Bacteria;Bacteroidetes;Bacteroidia;Bacteroidales;Prevotellaceae
OTU_0718	Bacteria;Firmicutes;Clostridia;Clostridiales
OTU_0719	Bacteria;Firmicutes;Erysipelotrichia;Erysipelotrichales;Erysipelotrichaceae;Erysipelotrichaceae_incertae_sedis
OTU_0720	Bacteria;Firmicutes;Clostridia;Clostridiales;Lachnospiraceae;Shuttleworthia
OTU_0721	Bacteria;Firmicutes;Clostridia;Clostridiales;Lachnospiraceae;Clostridium XlVa
OTU_0722	Bacteria;Proteobacteria;Alphaproteobacteria;Rhizobiales;Bradyrhizobiaceae;Bradyrhizobium

OTU_0723	Bacteria;Firmicutes;Clostridia;Clostridiales;Peptococcaceae 1;Peptococcus
OTU_0724	Bacteria;Firmicutes;Clostridia;Clostridiales;Lachnospiraceae
OTU_0725	Bacteria;Firmicutes;Negativicutes;Selenomonadales;Veillonellaceae;Anaerovibrio
OTU_0726	Bacteria;Firmicutes;Erysipelotrichia;Erysipelotrichales;Erysipelotrichaceae;Allobaculum
OTU_0727	Bacteria;Firmicutes;Erysipelotrichia;Erysipelotrichales;Erysipelotrichaceae;Erysipelotrichaceae_incertae_sedis
OTU_0728	Bacteria
OTU_0729	Bacteria;Firmicutes;Clostridia;Clostridiales;Ruminococcaceae;Clostridium III
OTU_0730	Bacteria
OTU_0731	Bacteria;Proteobacteria;Gammaproteobacteria;Pseudomonadales;Pseudomonadaceae;Pseudomonas
OTU_0732	Bacteria
OTU_0733	Bacteria;Firmicutes;Clostridia;Clostridiales;Ruminococcaceae;Hydrogenoanaerobacterium
OTU_0734	Bacteria;Firmicutes;Clostridia;Clostridiales;Eubacteriaceae;Eubacterium
OTU_0735	Bacteria
OTU_0736	Bacteria;Bacteroidetes;Bacteroidia;Bacteroidales;Prevotellaceae;Prevotella
OTU_0737	Bacteria;Bacteroidetes;Sphingobacteria;Sphingobacteriales
OTU_0738	Bacteria;Bacteroidetes;Bacteroidia;Bacteroidales;Porphyromonadaceae;Barnesiella
OTU_0739	Bacteria;Firmicutes;Clostridia;Clostridiales;Ruminococcaceae
OTU_0740	Bacteria;TM7;TM7_genera_incertae_sedis;TM7_genera_incertae_sedis;TM7_genera_incertae_sedis;TM7_genera_incer tae_sedis
OTU_0741	Bacteria;Firmicutes;Clostridia;Clostridiales
OTU_0742	Bacteria;Firmicutes;Clostridia;Clostridiales;Lachnospiraceae;Lachnospiracea_incertae_sedis
OTU_0743	Bacteria;Firmicutes;Clostridia;Clostridiales;Ruminococcaceae;Anaerotruncus
OTU_0744	Bacteria;Firmicutes;Clostridia;Clostridiales;Ruminococcaceae;Sporobacter
OTU_0745	Bacteria;Proteobacteria;Gammaproteobacteria;Pseudomonadales;Moraxellaceae;Acinetobacter
OTU_0746	Bacteria;Bacteroidetes;Bacteroidia;Bacteroidales;Porphyromonadaceae;Barnesiella
OTU_0747	Bacteria;Firmicutes;Clostridia;Clostridiales;Lachnospiraceae;Clostridium XlVb
OTU_0748	Bacteria;Firmicutes;Negativicutes;Selenomonadales;Veillonellaceae;Megamonas
OTU_0749	Bacteria;Actinobacteria;Actinobacteria;Bifidobacteriales;Bifidobacteriaceae;Bifidobacterium
OTU_0750	Bacteria;Bacteroidetes;Bacteroidia;Bacteroidales;Porphyromonadaceae;Parabacteroides
OTU_0751	Bacteria;Firmicutes;Clostridia;Clostridiales;Ruminococcaceae;Clostridium IV
OTU_0752	Bacteria;Firmicutes;Clostridia;Clostridiales;Ruminococcaceae;Oscillibacter
OTU_0753	Bacteria;Firmicutes;Erysipelotrichia;Erysipelotrichales;Erysipelotrichaceae;Clostridium XVIII
OTU_0754	Bacteria;Actinobacteria;Actinobacteria;Actinomycetales;Actinomycetaceae;Actinomyces
OTU_0755	Bacteria;Firmicutes;Clostridia;Clostridiales;Ruminococcaceae;Sporobacter
OTU_0756	Bacteria;Bacteroidetes;Bacteroidia;Bacteroidales;Porphyromonadaceae;Odoribacter
OTU_0757	Bacteria
OTU_0758	Bacteria;Firmicutes;Clostridia;Clostridiales;Eubacteriaceae;Eubacterium
OTU_0759	Bacteria
OTU_0760	Bacteria;Bacteroidetes;Bacteroidia;Bacteroidales;Bacteroidaceae;Bacteroides
OTU_0761	Bacteria;Proteobacteria;Gammaproteobacteria;Pseudomonadales;Pseudomonadaceae;Pseudomonas
OTU_0762	Bacteria;Firmicutes;Negativicutes;Selenomonadales;Veillonellaceae;Dialister
OTU_0763	Bacteria;Firmicutes;Clostridia;Clostridiales;Ruminococcaceae;Acetanaerobacterium
OTU_0764	Bacteria;Firmicutes;Clostridia;Clostridiales;Ruminococcaceae
OTU_0765	Bacteria;Proteobacteria;Alphaproteobacteria;Sphingomonadales;Sphingomonadaceae;Sphingomonas
OTU_0766	Bacteria;Firmicutes;Clostridia;Clostridiales
OTU_0767	Bacteria;Proteobacteria;Alphaproteobacteria;Caulobacterales;Caulobacteraceae;Brevundimonas
OTU_0768	Bacteria;Firmicutes;Clostridia;Clostridiales;Lachnospiraceae
OTU_0769	Bacteria;Actinobacteria;Actinobacteria;Coriobacteriales;Coriobacteriaceae;Paraeggerthella
OTU_0770	Bacteria;Firmicutes;Clostridia;Clostridiales;Lachnospiraceae;Clostridium XlVb
OTU_0771	Bacteria;Bacteroidetes;Bacteroidia;Bacteroidales;Prevotellaceae;Prevotella
OTU_0772	Bacteria;Firmicutes;Clostridia;Clostridiales;Clostridiales_Incertae Sedis XIII;Mogibacterium
OTU_0773	Bacteria;Firmicutes;Clostridia;Clostridiales

OTU_0774	Bacteria;Firmicutes;Clostridia;Clostridiales
OTU_0775	Bacteria;Firmicutes;Clostridia;Clostridiales
OTU_0776	Bacteria;Firmicutes;Clostridia;Clostridiales;Lachnospiraceae;Lachnospiracea_incertae_sedis
OTU_0777	Bacteria;Deinococcus-Thermus;Deinococci;Thermales;Thermaceae;Thermus
OTU_0778	Bacteria;Lentisphaerae;Lentisphaeria;Victivallales;Victivallaceae;Victivallis
OTU_0779	Bacteria;Firmicutes;Clostridia;Clostridiales;Lachnospiraceae;Clostridium XlVa
OTU_0780	Bacteria;Firmicutes;Clostridia;Clostridiales;Ruminococcaceae;Clostridium IV
OTU_0781	Bacteria;Firmicutes;Clostridia;Clostridiales
OTU_0782	Bacteria;Firmicutes;Clostridia;Clostridiales;Ruminococcaceae
OTU_0783	Bacteria;Firmicutes;Clostridia;Clostridiales;Ruminococcaceae;Oscillibacter
OTU_0784	Bacteria;Firmicutes;Clostridia;Clostridiales;Lachnospiraceae
OTU_0785	Bacteria;Firmicutes;Clostridia;Clostridiales;Ruminococcaceae
OTU_0786	Bacteria;Firmicutes;Clostridia;Clostridiales;Lachnospiraceae
OTU_0787	Bacteria;Firmicutes;Clostridia;Clostridiales
OTU_0788	Bacteria;Bacteroidetes;Bacteroidia;Bacteroidales;Porphyromonadaceae;Barnesiella
OTU_0789	Bacteria;Firmicutes;Clostridia;Clostridiales;Ruminococcaceae;Anaerotruncus
OTU_0790	Bacteria;Firmicutes;Clostridia;Clostridiales;Lachnospiraceae;Lachnospiracea_incertae_sedis
OTU_0791	Bacteria;Fusobacteria;Fusobacteria;Fusobacteriales;Leptotrichiaceae;Sneathia
OTU_0792	Bacteria;Proteobacteria;Gammaproteobacteria;Enterobacteriales;Enterobacteriaceae;Serratia
OTU_0793	Bacteria;Proteobacteria;Gammaproteobacteria;Aeromonadales;Succinivibrionaceae;Succinatimonas
OTU_0794	Bacteria;Firmicutes;Clostridia;Clostridiales;Lachnospiraceae;Clostridium XlVa
OTU_0795	Bacteria;Firmicutes;Negativicutes;Selenomonadales;Veillonellaceae;Megasphaera
OTU_0796	Bacteria;Bacteroidetes;Bacteroidia;Bacteroidales;Bacteroidaceae;Bacteroides
OTU_0797	Bacteria;Bacteroidetes
OTU_0798	Bacteria;Firmicutes;Clostridia;Clostridiales;Lachnospiraceae
OTU_0799	Bacteria;Firmicutes;Erysipelotrichia;Erysipelotrichales;Erysipelotrichaceae;Erysipelotrichaceae_incertae_sedis
OTU_0800	Bacteria;Proteobacteria;Gammaproteobacteria;Enterobacteriales;Enterobacteriaceae;Morganella
OTU_0801	Bacteria;Firmicutes;Clostridia;Clostridiales;Ruminococcaceae
OTU_0802	Bacteria;Firmicutes;Clostridia;Clostridiales;Ruminococcaceae;Oscillibacter
OTU_0803	Bacteria;Firmicutes
OTU_0804	Bacteria;Bacteroidetes;Bacteroidia;Bacteroidales;Prevotellaceae;Paraprevotella
OTU_0805	Bacteria;Firmicutes;Clostridia;Clostridiales;Lachnospiraceae
OTU_0806	Bacteria;Firmicutes;Clostridia;Clostridiales
OTU_0807	Bacteria;Firmicutes;Bacilli;Lactobacillales;Lactobacillaceae;Lactobacillus
OTU_0808	Bacteria;Proteobacteria;Gammaproteobacteria;Vibrionales;Vibrionaceae;Vibrio
OTU_0809	Bacteria;Actinobacteria;Actinobacteria;Coriobacteriales;Coriobacteriaceae
OTU_0810	Bacteria
OTU_0811	Bacteria;Synergistetes;Synergistia;Synergistales;Synergistaceae;Pyramidobacter
OTU_0812	Bacteria;Bacteroidetes;Bacteroidia;Bacteroidales;Prevotellaceae;Prevotella
OTU_0813	Bacteria;Bacteroidetes;Bacteroidia;Bacteroidales;Porphyromonadaceae;Butyricimonas
OTU_0814	Bacteria
OTU_0815	Bacteria;Bacteroidetes;Bacteroidia;Bacteroidales;Porphyromonadaceae;Barnesiella
OTU_0816	Bacteria;Firmicutes
OTU_0817	Bacteria
OTU_0818	Bacteria;Actinobacteria;Actinobacteria;Actinomycetales;Micrococcaceae;Rothia
OTU_0819	Bacteria;Firmicutes;Erysipelotrichia;Erysipelotrichales;Erysipelotrichaceae;Erysipelotrichaceae_incertae_sedis
OTU_0820	Bacteria;Firmicutes;Clostridia;Clostridiales
OTU_0821	Bacteria;Proteobacteria;Deltaproteobacteria;Desulfovibrionales;Desulfovibrionaceae
OTU_0822	Bacteria;Firmicutes;Clostridia
OTU_0823	Bacteria;Cyanobacteria/Chloroplast;Chloroplast;Chloroplast;Chloroplast;Streptophyta
OTU_0824	Bacteria;Firmicutes;Clostridia;Clostridiales;Ruminococcaceae
OTU_0825	Bacteria;Firmicutes;Clostridia;Clostridiales;Lachnospiraceae;Lachnospiracea_incertae_sedis

OTU_0826	Bacteria;Firmicutes;Clostridia;Clostridiales;Lachnospiraceae;Lachnospiracea_incertae_sedis
OTU_0827	Bacteria;Firmicutes;Negativicutes;Selenomonadales;Veillonellaceae;Dialister
OTU_0828	Bacteria;Firmicutes;Erysipelotrichia;Erysipelotrichales;Erysipelotrichaceae;Erysipelotrichaceae_incertae_sedis
OTU_0829	Bacteria;Firmicutes;Clostridia;Clostridiales;Ruminococcaceae
OTU_0830	Bacteria;Firmicutes;Clostridia;Clostridiales
OTU_0831	Bacteria;Firmicutes
OTU_0832	Bacteria
OTU_0833	Bacteria;Firmicutes;Clostridia;Clostridiales;Ruminococcaceae;Clostridium IV
OTU_0834	Bacteria;Firmicutes;Clostridia;Clostridiales;Ruminococcaceae;Pseudoflavonifractor
OTU_0835	Bacteria;Bacteroidetes;Bacteroidia;Bacteroidales;Bacteroidaceae;Bacteroides
OTU_0836	Bacteria;Firmicutes;Clostridia;Clostridiales;Ruminococcaceae
OTU_0837	Bacteria;Firmicutes;Clostridia;Clostridiales;Ruminococcaceae;Clostridium IV
OTU_0838	Bacteria;Proteobacteria;Betaproteobacteria;Burkholderiales;Burkholderiales_incertae_sedis;Aquabacterium
OTU_0839	Bacteria
OTU_0840	Bacteria;Firmicutes;Clostridia;Clostridiales;Clostridiales_Incertae Sedis XI;Peptoniphilus
OTU_0841	Bacteria;Firmicutes;Clostridia
OTU_0842	Bacteria
OTU_0843	Bacteria;Bacteroidetes;Bacteroidia;Bacteroidales;Prevotellaceae;Prevotella
OTU_0844	Bacteria;Synergistetes;Synergistia;Synergistales;Synergistaceae;Aminobacterium
OTU_0845	Bacteria;Bacteroidetes;Bacteroidia;Bacteroidales;Porphyromonadaceae;Porphyromonas
OTU_0846	Bacteria;Firmicutes;Clostridia;Clostridiales;Ruminococcaceae
OTU_0847	Bacteria;Firmicutes;Clostridia;Clostridiales;Ruminococcaceae;Flavonifractor
OTU_0848	Bacteria;Firmicutes;Clostridia;Clostridiales;Ruminococcaceae
OTU_0849	Bacteria;Firmicutes;Clostridia;Clostridiales;Lachnospiraceae;Lachnospiracea_incertae_sedis
OTU_0850	Bacteria;Firmicutes;Clostridia;Clostridiales;Ruminococcaceae
OTU_0851	Bacteria;Firmicutes;Clostridia;Clostridiales;Eubacteriaceae;Eubacterium
OTU_0852	Bacteria;Bacteroidetes;Bacteroidia;Bacteroidales;Rikenellaceae;Alistipes
OTU_0853	Bacteria;Bacteroidetes;Bacteroidia;Bacteroidales;Porphyromonadaceae;Barnesiella
OTU_0854	Bacteria;Firmicutes;Clostridia;Clostridiales
OTU_0855	Bacteria;Firmicutes;Clostridia;Clostridiales;Ruminococcaceae
OTU_0856	Bacteria;Proteobacteria;Alphaproteobacteria;Rhodobacterales;Rhodobacteraceae;Paracoccus
OTU_0857	Bacteria;Firmicutes;Clostridia;Clostridiales
OTU_0858	Bacteria;Actinobacteria;Actinobacteria;Actinomycetales;Micrococcaceae;Micrococcus
OTU_0859	Bacteria;Proteobacteria;Gammaproteobacteria
OTU_0860	Bacteria;Firmicutes;Clostridia;Clostridiales;Ruminococcaceae;Flavonifractor
OTU_0861	Bacteria;Firmicutes;Bacilli;Lactobacillales;Lactobacillaceae;Lactobacillus
OTU_0862	Bacteria;Firmicutes;Bacilli;Lactobacillales;Leuconostocaceae;Weissella
OTU_0863	Bacteria;Proteobacteria;Betaproteobacteria;Burkholderiales;Comamonadaceae;Diaphorobacter
OTU_0864	Bacteria;Firmicutes;Clostridia;Clostridiales;Lachnospiraceae;Lachnospiracea_incertae_sedis
OTU_0865	Bacteria
OTU_0866	Bacteria;Firmicutes;Clostridia;Clostridiales;Clostridiales_Incertae Sedis XI;Anaerococcus
OTU_0867	Bacteria;Bacteroidetes;Bacteroidia;Bacteroidales;Prevotellaceae;Prevotella
OTU_0868	Bacteria;Proteobacteria;Betaproteobacteria;Burkholderiales
OTU_0869	Bacteria;Firmicutes;Clostridia;Clostridiales;Ruminococcaceae
OTU_0870	Bacteria;Firmicutes;Clostridia;Clostridiales;Ruminococcaceae
OTU_0871	Bacteria;Bacteroidetes;Bacteroidia;Bacteroidales;Porphyromonadaceae
OTU_0872	Bacteria;Firmicutes;Clostridia;Clostridiales;Ruminococcaceae;Flavonifractor
OTU_0873	Bacteria;Proteobacteria
OTU_0874	Bacteria;Firmicutes;Clostridia;Clostridiales;Ruminococcaceae;Hydrogenoanaerobacterium
OTU_0875	Bacteria;Firmicutes;Clostridia;Clostridiales
OTU_0876	Bacteria;Bacteroidetes;Bacteroidia;Bacteroidales;Porphyromonadaceae;Parabacteroides
OTU_0877	Bacteria;Firmicutes;Clostridia;Clostridiales

OTU_0878	Bacteria;Cyanobacteria/Chloroplast;Chloroplast;Chloroplast;Chloroplast;Streptophyta
OTU_0879	Bacteria;Firmicutes;Clostridia;Clostridiales;Lachnospiraceae
OTU_0880	Bacteria;Actinobacteria;Actinobacteria;Actinomycetales;Intrasporangiaceae;Janibacter
OTU_0881	Bacteria;Firmicutes;Clostridia;Clostridiales;Ruminococcaceae;Clostridium IV
OTU_0882	Bacteria;Firmicutes;Clostridia;Clostridiales;Ruminococcaceae;Acetivibrio
OTU_0883	Unclassified
OTU_0884	Bacteria;Firmicutes;Clostridia;Clostridiales;Lachnospiraceae;Clostridium XlVa
OTU_0885	Bacteria;Bacteroidetes;Bacteroidia;Bacteroidales;Porphyromonadaceae;Barnesiella
OTU_0886	Bacteria;Bacteroidetes;Bacteroidia;Bacteroidales;Bacteroidaceae;Bacteroides
OTU_0887	Bacteria;Bacteroidetes;Sphingobacteria;Sphingobacteriales
OTU_0888	Bacteria;Firmicutes;Clostridia;Clostridiales;Ruminococcaceae;Clostridium IV
OTU_0889	Bacteria;Firmicutes;Clostridia;Clostridiales;Ruminococcaceae;Pseudoflavonifractor
OTU_0890	Bacteria
OTU_0891	Bacteria;Bacteroidetes;Bacteroidia;Bacteroidales;Prevotellaceae;Prevotella
OTU_0892	Bacteria;Actinobacteria;Actinobacteria;Coriobacteriales;Coriobacteriaceae
OTU_0893	Bacteria;Firmicutes
OTU_0894	Bacteria;Actinobacteria;Actinobacteria;Actinomycetales;Actinomycetaceae;Actinomyces
OTU_0895	Bacteria;Firmicutes;Clostridia;Clostridiales;Peptococcaceae 1;Peptococcus
OTU_0896	Bacteria
OTU_0897	Bacteria;Firmicutes;Clostridia;Clostridiales;Ruminococcaceae
OTU_0898	Bacteria;Firmicutes;Clostridia;Clostridiales;Clostridiaceae 1;Anaerosporobacter
OTU_0899	Bacteria;Firmicutes;Clostridia;Clostridiales;Ruminococcaceae;Oscillibacter
OTU_0900	Bacteria;Firmicutes;Clostridia;Clostridiales;Ruminococcaceae;Clostridium IV
OTU_0901	Bacteria;Bacteroidetes;Bacteroidia;Bacteroidales;Prevotellaceae;Prevotella
OTU_0902	Bacteria;Bacteroidetes;Bacteroidia;Bacteroidales;Porphyromonadaceae;Barnesiella
OTU_0903	Bacteria;Actinobacteria;Actinobacteria;Coriobacteriales;Coriobacteriaceae;Olsenella
OTU_0904	Bacteria;Bacteroidetes;Bacteroidia;Bacteroidales;Porphyromonadaceae;Porphyromonas
OTU_0905	Bacteria;Firmicutes;Clostridia;Clostridiales;Ruminococcaceae;Clostridium IV
OTU_0906	Bacteria;Firmicutes;Clostridia;Clostridiales;Ruminococcaceae;Acetanaerobacterium
OTU_0907	Bacteria;Firmicutes;Clostridia;Clostridiales;Incertae Sedis XI;Murdochiella
OTU_0908	Bacteria;Proteobacteria;Deltaproteobacteria;Desulfovibrionales;Desulfovibrionaceae;Desulfovibrio
OTU_0909	Bacteria;Bacteroidetes;Bacteroidia;Bacteroidales;Bacteroidaceae;Bacteroides
OTU_0910	Bacteria;Proteobacteria;Epsilonproteobacteria;Campylobacterales;Campylobacteraceae;Campylobacter
OTU_0911	Bacteria;Actinobacteria;Actinobacteria;Actinomycetales;Actinomycetaceae;Actinomyces
OTU_0912	Bacteria;Firmicutes;Clostridia;Clostridiales;Ruminococcaceae;Clostridium IV
OTU_0913	Bacteria;Firmicutes;Clostridia;Clostridiales;Ruminococcaceae
OTU_0914	Bacteria;Actinobacteria;Actinobacteria;Coriobacteriales;Coriobacteriaceae;Slackia
OTU_0915	Bacteria;Firmicutes;Clostridia;Clostridiales
OTU_0916	Bacteria;Proteobacteria;Betaproteobacteria;Burkholderiales;Comamonadaceae;Variovorax
OTU_0917	Bacteria;Bacteroidetes;Bacteroidia;Bacteroidales;Rikenellaceae;Alistipes
OTU_0918	Bacteria;Actinobacteria;Actinobacteria;Coriobacteriales;Coriobacteriaceae;Collinsella
OTU_0919	Bacteria;Firmicutes;Erysipelotrichia;Erysipelotrichales;Erysipelotrichaceae;Clostridium XVIII
OTU_0920	Bacteria;Firmicutes;Clostridia;Clostridiales;Lachnospiraceae;Lachnospiracea_incertae_sedis
OTU_0921	Bacteria;Bacteroidetes;Bacteroidia;Bacteroidales;Prevotellaceae;Prevotella
OTU_0922	Bacteria;Proteobacteria;Alphaproteobacteria;Sphingomonadales;Sphingomonadaceae;Sphingobium
OTU_0923	Bacteria;Firmicutes;Clostridia;Clostridiales
OTU_0924	Bacteria;Verrucomicrobia;Subdivision3;Subdivision3_genera_incertae_sedis;Subdivision3_genera_incertae_sedis;Subdivision3_genera_incertae_sedis
OTU_0925	Bacteria;Firmicutes
OTU_0926	Bacteria;Proteobacteria;Deltaproteobacteria;Desulfovibrionales;Desulfovibrionaceae;Desulfovibrio
OTU_0927	Bacteria;Firmicutes;Clostridia;Clostridiales;Ruminococcaceae
OTU_0928	Bacteria;Bacteroidetes;Bacteroidia;Bacteroidales;Prevotellaceae;Prevotella

OTU_0929	Bacteria;Proteobacteria;Betaproteobacteria;Neisseriales;Neisseriaceae;Neisseria
OTU_0930	Bacteria;Firmicutes;Clostridia;Clostridiales;Lachnospiraceae;Clostridium XlVa
OTU_0931	Bacteria;Firmicutes;Clostridia;Clostridiales;Ruminococcaceae;Clostridium IV
OTU_0932	Bacteria;Bacteroidetes;Bacteroidia;Bacteroidales;Prevotellaceae;Prevotella
OTU_0933	Bacteria;Firmicutes;Clostridia;Clostridiales
OTU_0934	Bacteria
OTU_0935	Bacteria;Bacteroidetes;Bacteroidia;Bacteroidales;Porphyromonadaceae;Parabacteroides
OTU_0936	Unclassified
OTU_0937	Bacteria;Firmicutes;Clostridia;Clostridiales
OTU_0938	Bacteria;Bacteroidetes;Bacteroidia;Bacteroidales;Prevotellaceae;Prevotella
OTU_0939	Bacteria;Bacteroidetes;Bacteroidia;Bacteroidales;Rikenellaceae;Alistipes
OTU_0940	Bacteria;Firmicutes;Bacilli;Lactobacillales;Streptococcaceae;Streptococcus
OTU_0941	Bacteria;Bacteroidetes;Bacteroidia;Bacteroidales;Prevotellaceae
OTU_0942	Bacteria;Firmicutes;Clostridia;Clostridiales;Ruminococcaceae;Oscillibacter
OTU_0943	Bacteria;Bacteroidetes;Flavobacteria;Flavobacteriales;Flavobacteriaceae;Cloacibacterium
OTU_0944	Bacteria;Firmicutes;Clostridia;Clostridiales;Ruminococcaceae;Clostridium IV
OTU_0945	Bacteria;Proteobacteria;Alphaproteobacteria;Rhizobiales;Methylobacteriaceae;Methylobacterium
OTU_0946	Bacteria;Firmicutes;Clostridia;Clostridiales;Ruminococcaceae;Faecalibacterium
OTU_0947	Bacteria;Firmicutes;Clostridia;Clostridiales;Lachnospiraceae;Lachnospiracea_incertae_sedis
OTU_0948	Bacteria;Bacteroidetes;Bacteroidia;Bacteroidales;Prevotellaceae;Prevotella
OTU_0949	Bacteria;Proteobacteria;Betaproteobacteria;Burkholderiales;Comamonadaceae;Delftia
OTU_0950	Bacteria;Actinobacteria;Actinobacteria;Actinomycetales;Actinomycetaceae;Actinomyces
OTU_0951	Bacteria;TM7;TM7_genera_incertae_sedis;TM7_genera_incertae_sedis;TM7_genera_incertae_sedis;TM7_genera_incertae_sedis
OTU_0952	Bacteria;Firmicutes;Clostridia;Clostridiales;Ruminococcaceae;Clostridium IV
OTU_0953	Bacteria
OTU_0954	Bacteria
OTU_0955	Bacteria;Firmicutes;Clostridia;Clostridiales;Lachnospiraceae;Acetitomaculum
OTU_0956	Bacteria;Firmicutes;Clostridia;Clostridiales;Ruminococcaceae
OTU_0957	Bacteria;Firmicutes;Clostridia;Clostridiales;Lachnospiraceae;Lachnospiracea_incertae_sedis
OTU_0958	Bacteria;Firmicutes;Clostridia;Clostridiales;Ruminococcaceae;Oscillibacter
OTU_0959	Bacteria;Actinobacteria;Actinobacteria;Bifidobacteriales;Bifidobacteriaceae;Gardnerella
OTU_0960	Bacteria;Firmicutes;Clostridia;Clostridiales;Ruminococcaceae;Butyricicoccus
OTU_0961	Bacteria;Bacteroidetes;Bacteroidia;Bacteroidales;Prevotellaceae
OTU_0962	Bacteria;Actinobacteria;Actinobacteria;Coriobacteriales;Coriobacteriaceae
OTU_0963	Bacteria;Firmicutes;Clostridia;Clostridiales;Lachnospiraceae;Clostridium XlVb
OTU_0964	Bacteria;Firmicutes;Clostridia;Clostridiales;Gracilibacteraceae
OTU_0965	Bacteria;Firmicutes
OTU_0966	Bacteria;Bacteroidetes;Bacteroidia;Bacteroidales;Prevotellaceae;Prevotella
OTU_0967	Bacteria
OTU_0968	Bacteria;Bacteroidetes;Bacteroidia;Bacteroidales;Prevotellaceae;Prevotella
OTU_0969	Bacteria;Bacteroidetes;Bacteroidia;Bacteroidales;Bacteroidaceae;Bacteroides
OTU_0970	Bacteria;Firmicutes;Bacilli;Lactobacillales;Streptococcaceae;Streptococcus
OTU_0971	Bacteria;Bacteroidetes;Bacteroidia;Bacteroidales;Bacteroidaceae;Bacteroides
OTU_0972	Bacteria;Proteobacteria;Betaproteobacteria;Burkholderiales;Sutterellaceae;Parasutterella
OTU_0973	Bacteria;Bacteroidetes;Bacteroidia;Bacteroidales;Prevotellaceae;Prevotella
OTU_0974	Archaea;Euryarchaeota;Methanomicrobia;Methanosarcinales;Methanosarcinaceae;Methanosarcina
OTU_0975	Bacteria;Proteobacteria;Deltaproteobacteria;Desulfovibrionales;Desulfovibrionaceae;Desulfovibrio
OTU_0976	Bacteria;Bacteroidetes;Bacteroidia;Bacteroidales;Prevotellaceae;Paraprevotella
OTU_0977	Bacteria;Bacteroidetes;Bacteroidia;Bacteroidales;Prevotellaceae;Prevotella
OTU_0978	Bacteria;Proteobacteria;Betaproteobacteria;Neisseriales;Neisseriaceae;Neisseria
OTU_0979	Bacteria;Firmicutes;Clostridia;Clostridiales;Ruminococcaceae

OTU_0980	Bacteria;Bacteroidetes;Bacteroidia;Bacteroidales
OTU_0981	Bacteria;Proteobacteria;Deltaproteobacteria;Desulfovibrionales;Desulfovibrionaceae;Desulfovibrio
OTU_0982	Bacteria;Proteobacteria;Alphaproteobacteria;Rhizobiales;Methylobacteriaceae;Methylobacterium
OTU_0983	Bacteria;Firmicutes;Clostridia;Clostridiales;Ruminococcaceae;Ruminococcus
OTU_0984	Bacteria;Firmicutes
OTU_0985	Bacteria;Firmicutes;Clostridia;Clostridiales;Ruminococcaceae;Hydrogenoanaerobacterium
OTU_0986	Bacteria;Firmicutes;Clostridia;Clostridiales;Ruminococcaceae
OTU_0987	Bacteria;Bacteroidetes;Sphingobacteria;Sphingobacteriales;Sphingobacteriaceae;Sphingobacterium
OTU_0988	Bacteria;Actinobacteria;Actinobacteria;Coriobacteriales;Coriobacteriaceae
OTU_0989	Bacteria
OTU_0990	Bacteria;Bacteroidetes;Bacteroidia;Bacteroidales;Prevotellaceae;Prevotella
OTU_0991	Bacteria;Firmicutes;Erysipelotrichia;Erysipelotrichales;Erysipelotrichaceae
OTU_0992	Bacteria;Firmicutes;Clostridia;Clostridiales;Ruminococcaceae
OTU_0993	Bacteria;Firmicutes;Bacilli;Bacillales;Bacillales_Incertae Sedis XII;Exiguobacterium
OTU_0994	Bacteria;Firmicutes;Negativicutes;Selenomonadales;Veillonellaceae
OTU_0995	Bacteria;Firmicutes;Clostridia;Clostridiales;Syntrophomonadaceae;Syntrophomonas
OTU_0996	Bacteria;Firmicutes;Clostridia;Clostridiales;Ruminococcaceae
OTU_0997	Bacteria;Proteobacteria;Betaproteobacteria;Neisseriales;Neisseriaceae;Eikenella
OTU_0998	Bacteria;Bacteroidetes;Bacteroidia;Bacteroidales;Bacteroidaceae;Bacteroides
OTU_0999	Bacteria;Proteobacteria;Betaproteobacteria;Burkholderiales;Comamonadaceae;Brachymonas
OTU_1000	Bacteria;Bacteroidetes;Bacteroidia;Bacteroidales;Porphyromonadaceae;Parabacteroides
OTU_1001	Bacteria;Proteobacteria;Betaproteobacteria;Neisseriales;Neisseriaceae;Kingella
OTU_1002	Bacteria;Firmicutes;Clostridia;Clostridiales
OTU_1003	Bacteria;Firmicutes;Clostridia;Clostridiales
OTU_1004	Unclassified
OTU_1005	Bacteria;Firmicutes;Clostridia;Clostridiales;Incertae Sedis XI;Murdochiella
OTU_1006	Bacteria;Bacteroidetes;Bacteroidia;Bacteroidales;Rikenellaceae;Alistipes
OTU_1007	Bacteria;Proteobacteria;Gammaproteobacteria;Gammaproteobacteria_incertae_sedis
OTU_1008	Bacteria;Firmicutes;Clostridia;Clostridiales;Ruminococcaceae
OTU_1009	Bacteria;Firmicutes;Negativicutes;Selenomonadales;Veillonellaceae;Negativicoccus
OTU_1010	Bacteria;Bacteroidetes;Bacteroidia;Bacteroidales;Porphyromonadaceae;Parabacteroides
OTU_1011	Bacteria;Firmicutes;Clostridia;Clostridiales;Ruminococcaceae;Anaerofilum
OTU_1012	Bacteria;Firmicutes;Clostridia;Clostridiales;Ruminococcaceae;Anaerotruncus
OTU_1013	Bacteria;Firmicutes;Bacilli;Lactobacillales;Carnobacteriaceae;Atopostipes
OTU_1014	Bacteria;Firmicutes;Erysipelotrichia;Erysipelotrichales;Erysipelotrichaceae;Clostridium XVIII
OTU_1015	Bacteria;Firmicutes;Clostridia;Clostridiales;Ruminococcaceae
OTU_1016	Bacteria;Firmicutes;Clostridia;Clostridiales;Lachnospiraceae;Lachnospiracea_incertae_sedis
OTU_1017	Bacteria;Bacteroidetes;Bacteroidia;Bacteroidales;Bacteroidaceae;Bacteroides
OTU_1018	Bacteria;Proteobacteria;Alphaproteobacteria;Rhizobiales;Hyphomicrobiaceae;Gemmiger
OTU_1019	Bacteria;Bacteroidetes;Bacteroidia;Bacteroidales;Prevotellaceae;Prevotella
OTU_1020	Bacteria;Bacteroidetes;Bacteroidia;Bacteroidales;Porphyromonadaceae;Parabacteroides
OTU_1021	Bacteria;Proteobacteria;Gammaproteobacteria;Legionellales;Legionellaceae;Legionella
OTU_1022	Bacteria;Firmicutes;Clostridia;Clostridiales;Ruminococcaceae
OTU_1023	Bacteria;Actinobacteria;Actinobacteria;Coriobacteriales;Coriobacteriaceae;Paraeggerthella
OTU_1024	Bacteria;Firmicutes;Erysipelotrichia;Erysipelotrichales;Erysipelotrichaceae;Bulleidia
OTU_1025	Bacteria;Firmicutes;Clostridia;Clostridiales;Lachnospiraceae;Lachnospiracea_incertae_sedis
OTU_1026	Bacteria;Bacteroidetes;Bacteroidia;Bacteroidales;Porphyromonadaceae;Barnesiella
OTU_1027	Bacteria;Firmicutes;Clostridia;Clostridiales;Lachnospiraceae;Lachnospiracea_incertae_sedis
OTU_1028	Bacteria;Bacteroidetes;Bacteroidia;Bacteroidales;Porphyromonadaceae
OTU_1029	Bacteria;Firmicutes;Clostridia;Clostridiales
OTU_1030	Bacteria;Firmicutes;Clostridia;Clostridiales;Ruminococcaceae
OTU_1031	Bacteria;Actinobacteria;Actinobacteria;Coriobacteriales;Coriobacteriaceae;Gordonibacter

OTU_1032	Bacteria;Proteobacteria;Gammaproteobacteria;Enterobacteriales;Enterobacteriaceae;Klebsiella
OTU_1033	Bacteria;Bacteroidetes;Bacteroidia;Bacteroidales;Marinilabiaceae;Anaerophaga
OTU_1034	Bacteria;Fusobacteria;Fusobacteria;Fusobacteriales;Leptotrichiaceae;Leptotrichia
OTU_1035	Bacteria;Firmicutes;Clostridia;Clostridiales;Ruminococcaceae
OTU_1036	Bacteria;Firmicutes;Clostridia;Clostridiales;Ruminococcaceae
OTU_1037	Bacteria;Firmicutes;Negativicutes;Selenomonadales;Veillonellaceae;Selenomonas
OTU_1038	Bacteria;Proteobacteria;Alphaproteobacteria;Sphingomonadales;Sphingomonadaceae;Sphingomonas
OTU_1039	Bacteria;Firmicutes
OTU_1040	Bacteria;Firmicutes;Clostridia;Clostridiales;Ruminococcaceae
OTU_1041	Bacteria;Firmicutes;Clostridia;Clostridiales
OTU_1042	Bacteria;Proteobacteria;Betaproteobacteria;Burkholderiales;Burkholderiaceae;Cupriavidus
OTU_1043	Bacteria;Bacteroidetes;Bacteroidia;Bacteroidales;Porphyromonadaceae;Porphyromonas
OTU_1044	Bacteria;Firmicutes;Clostridia;Clostridiales;Lachnospiraceae;Lachnospiracea_incertae_sedis
OTU_1045	Bacteria;Bacteroidetes;Bacteroidia;Bacteroidales;Porphyromonadaceae;Parabacteroides
OTU_1046	Bacteria
OTU_1047	Bacteria;Firmicutes;Clostridia;Clostridiales;Ruminococcaceae;Clostridium IV
OTU_1048	Bacteria;Firmicutes;Clostridia;Clostridiales;Lachnospiraceae;Roseburia
OTU_1049	Bacteria;Firmicutes;Clostridia;Clostridiales
OTU_1050	Bacteria;Firmicutes
OTU_1051	Bacteria
OTU_1052	Bacteria;Bacteroidetes;Bacteroidia;Bacteroidales;Porphyromonadaceae;Barnesiella
OTU_1053	Bacteria;Firmicutes;Clostridia;Clostridiales;Ruminococcaceae;Anaerofilum
OTU_1054	Bacteria;Firmicutes;Clostridia;Clostridiales;Ruminococcaceae
OTU_1055	Bacteria;Actinobacteria;Actinobacteria;Coriobacteriales;Coriobacteriaceae;Olsenella
OTU_1056	Bacteria;Firmicutes;Clostridia;Clostridiales;Ruminococcaceae
OTU_1057	Bacteria;Firmicutes;Clostridia;Clostridiales;Lachnospiraceae;Lachnospiracea_incertae_sedis
OTU_1058	Bacteria;Firmicutes;Clostridia;Clostridiales;Ruminococcaceae;Clostridium IV
OTU_1059	Bacteria;Firmicutes;Clostridia
OTU_1060	Bacteria;Firmicutes;Clostridia;Clostridiales;Lachnospiraceae;Clostridium XlVb
OTU_1061	Bacteria;Bacteroidetes;Bacteroidia;Bacteroidales;Prevotellaceae;Prevotella
OTU_1062	Bacteria;Firmicutes;Clostridia;Clostridiales;Lachnospiraceae
OTU_1063	Bacteria;Proteobacteria;Gammaproteobacteria;Chromatiales;Chromatiaceae;Rheinheimera
OTU_1064	Bacteria;Proteobacteria;Betaproteobacteria;Neisseriales;Neisseriaceae
OTU_1065	Bacteria;Actinobacteria;Actinobacteria;Coriobacteriales;Coriobacteriaceae;Olsenella
OTU_1066	Bacteria;Firmicutes;Clostridia;Clostridiales;Ruminococcaceae
OTU_1067	Bacteria;Firmicutes;Clostridia;Clostridiales;Lachnospiraceae;Roseburia
OTU_1068	Bacteria;Firmicutes;Clostridia;Clostridiales;Clostridiales_Incertae Sedis XI;Tissierella
OTU_1069	Bacteria
OTU_1070	Bacteria;Bacteroidetes;Bacteroidia;Bacteroidales;Prevotellaceae
OTU_1071	Bacteria;Firmicutes;Bacilli;Lactobacillales;Lactobacillaceae;Lactobacillus
OTU_1072	Bacteria;Firmicutes
OTU_1073	Bacteria;Proteobacteria;Gammaproteobacteria;Enterobacteriales;Enterobacteriaceae;Escherichia/Shigella
OTU_1074	Bacteria;Firmicutes;Clostridia
OTU_1075	Bacteria;Bacteroidetes;Bacteroidia;Bacteroidales;Porphyromonadaceae;Barnesiella
OTU_1076	Bacteria;Bacteroidetes;Sphingobacteria;Sphingobacteriales;Sphingobacteriaceae;Pedobacter
OTU_1077	Bacteria;Firmicutes;Clostridia;Clostridiales;Lachnospiraceae;Moryella
OTU_1078	Bacteria;Firmicutes;Clostridia;Clostridiales;Ruminococcaceae
OTU_1079	Bacteria;Firmicutes;Clostridia;Clostridiales;Ruminococcaceae;Anaerotruncus
OTU_1080	Bacteria;Firmicutes;Clostridia;Clostridiales;Ruminococcaceae;Hydrogenoanaerobacterium
OTU_1081	Bacteria;Proteobacteria;Deltaproteobacteria;Desulfovibrionales
OTU_1082	Bacteria;Firmicutes;Clostridia;Clostridiales;Lachnospiraceae;Pseudobutyrivibrio
OTU_1083	Bacteria;Firmicutes;Clostridia;Clostridiales;Lachnospiraceae;Clostridium XlVa

OTU_1084	Bacteria;Firmicutes;Erysipelotrichia;Erysipelotrichales;Erysipelotrichaceae;Allobaculum
OTU_1085	Bacteria;Bacteroidetes;Bacteroidia;Bacteroidales;Porphyromonadaceae;Barnesiella
OTU_1086	Bacteria;Proteobacteria;Deltaproteobacteria;Desulfovibrionales;Desulfovibrionaceae;Bilophila
OTU_1087	Bacteria;Firmicutes;Clostridia;Clostridiales;Lachnospiraceae
OTU_1088	Bacteria;Actinobacteria;Actinobacteria;Actinomycetales;Corynebacteriaceae;Corynebacterium
OTU_1089	Bacteria;Firmicutes;Clostridia;Clostridiales;Ruminococcaceae
OTU_1090	Bacteria;Bacteroidetes;Bacteroidia;Bacteroidales;Prevotellaceae;Prevotella
OTU_1091	Bacteria;Firmicutes;Clostridia;Clostridiales;Ruminococcaceae
OTU_1092	Bacteria;Firmicutes;Clostridia;Clostridiales;Lachnospiraceae;Clostridium XlVb
OTU_1093	Bacteria;Firmicutes;Clostridia;Clostridiales
OTU_1094	Bacteria;Firmicutes;Clostridia;Clostridiales;Ruminococcaceae
OTU_1095	Bacteria;Actinobacteria;Actinobacteria;Actinomycetales;Cellulomonadaceae;Tropheryma
OTU_1096	Bacteria
OTU_1097	Bacteria;Bacteroidetes;Bacteroidia;Bacteroidales;Bacteroidaceae;Bacteroides
OTU_1098	Bacteria;Actinobacteria;Actinobacteria;Coriobacteriales;Coriobacteriaceae;Enterorhabdus
OTU_1099	Bacteria;Firmicutes;Clostridia;Clostridiales;Lachnospiraceae;Lachnospiracea_incertae_sedis
OTU_1100	Bacteria;Bacteroidetes;Bacteroidia;Bacteroidales;Porphyromonadaceae;Barnesiella
OTU_1101	Bacteria;Bacteroidetes;Bacteroidia;Bacteroidales
OTU_1102	Bacteria;Cyanobacteria/Chloroplast;Chloroplast;Chloroplast;Chloroplast;Streptophyta
OTU_1103	Bacteria;Firmicutes;Clostridia;Clostridiales;Ruminococcaceae
OTU_1104	Bacteria;Firmicutes;Clostridia;Clostridiales;Ruminococcaceae
OTU_1105	Bacteria;Firmicutes;Negativicutes;Selenomonadales;Acidaminococcaceae;Phascolarctobacterium
OTU_1106	Bacteria;Bacteroidetes
OTU_1107	Bacteria;Firmicutes;Clostridia;Clostridiales;Lachnospiraceae;Lachnospiracea_incertae_sedis
OTU_1108	Bacteria;Bacteroidetes;Bacteroidia;Bacteroidales;Prevotellaceae;Prevotella
OTU_1109	Bacteria;Bacteroidetes;Bacteroidia;Bacteroidales;Rikenellaceae;Alistipes
OTU_1110	Bacteria;Bacteroidetes;Bacteroidia;Bacteroidales;Porphyromonadaceae;Parabacteroides
OTU_1111	Bacteria;Firmicutes;Clostridia;Clostridiales
OTU_1112	Bacteria
OTU_1113	Bacteria;Firmicutes;Clostridia;Clostridiales;Lachnospiraceae;Blautia
OTU_1114	Bacteria;Bacteroidetes;Bacteroidia;Bacteroidales;Prevotellaceae;Prevotella
OTU_1115	Bacteria;Proteobacteria;Alphaproteobacteria;Rhodospirillales;Acetobacteraceae;Roseomonas
OTU_1116	Bacteria;Bacteroidetes;Bacteroidia;Bacteroidales;Bacteroidaceae;Bacteroides
OTU_1117	Bacteria;Actinobacteria;Actinobacteria;Actinomycetales;Corynebacteriaceae;Corynebacterium
OTU_1118	Bacteria;Firmicutes;Bacilli;Lactobacillales
OTU_1119	Bacteria;Firmicutes;Clostridia;Clostridiales;Lachnospiraceae;Lachnospiracea_incertae_sedis
OTU_1120	Bacteria;Proteobacteria;Betaproteobacteria;Burkholderiales;Burkholderiales_incertae_sedis;Tepidimonas
OTU_1121	Bacteria;Actinobacteria;Actinobacteria;Actinomycetales;Nocardiaceae;Rhodococcus
OTU_1122	Bacteria;Firmicutes;Clostridia;Clostridiales;Ruminococcaceae;Clostridium IV
OTU_1123	Unclassified
OTU_1124	Bacteria;Bacteroidetes;Bacteroidia;Bacteroidales;Prevotellaceae;Prevotella
OTU_1125	Bacteria;Firmicutes;Clostridia;Clostridiales;Lachnospiraceae;Roseburia
OTU_1126	Bacteria;Firmicutes;Clostridia;Clostridiales;Ruminococcaceae
OTU_1127	Bacteria;Firmicutes;Clostridia;Clostridiales;Ruminococcaceae;Faecalibacterium
OTU_1128	Bacteria;Proteobacteria;Alphaproteobacteria;Rhodospirillales;Rhodospirillaceae;Desertibacter
OTU_1129	Bacteria;Firmicutes;Clostridia;Clostridiales;Ruminococcaceae
OTU_1130	Bacteria;Firmicutes;Bacilli;Lactobacillales;Carnobacteriaceae;Dolosigranulum
OTU_1131	Bacteria
OTU_1132	Bacteria;Bacteroidetes;Bacteroidia;Bacteroidales;Prevotellaceae;Prevotella
OTU_1133	Bacteria;Bacteroidetes;Bacteroidia;Bacteroidales;Porphyromonadaceae;Barnesiella
OTU_1134	Bacteria;Proteobacteria;Deltaproteobacteria;Desulfovibrionales;Desulfovibrionaceae;Bilophila
OTU_1135	Bacteria;Firmicutes;Clostridia;Clostridiales;Lachnospiraceae;Roseburia

OTU_1136	Bacteria;Firmicutes;Erysipelotrichia;Erysipelotrichales;Erysipelotrichaceae;Turicibacter
OTU_1137	Bacteria;Bacteroidetes;Flavobacteria;Flavobacteriales;Flavobacteriaceae;Chryseobacterium
OTU_1138	Bacteria;Actinobacteria;Actinobacteria;Actinomycetales;Micrococcaceae;Kocuria
OTU_1139	Bacteria;Bacteroidetes;Bacteroidia;Bacteroidales;Porphyromonadaceae
OTU_1140	Bacteria;Firmicutes;Negativicutes;Selenomonadales;Veillonellaceae;Megasphaera
OTU_1141	Bacteria;Proteobacteria;Gammaproteobacteria;Oceanospirillales;Halomonadaceae;Halomonas
OTU_1142	Bacteria;Bacteroidetes;Bacteroidia;Bacteroidales;Prevotellaceae;Prevotella
OTU_1143	Bacteria;Bacteroidetes;Bacteroidia;Bacteroidales;Bacteroidaceae;Bacteroides
OTU_1144	Bacteria;Firmicutes;Negativicutes;Selenomonadales;Veillonellaceae;Dialister
OTU_1145	Bacteria;Bacteroidetes;Bacteroidia;Bacteroidales;Bacteroidaceae;Bacteroides
OTU_1146	Bacteria;Firmicutes;Clostridia;Clostridiales;Ruminococcaceae
OTU_1147	Bacteria;Bacteroidetes;Bacteroidia;Bacteroidales;Porphyromonadaceae;Porphyromonas
OTU_1148	Bacteria;Firmicutes;Bacilli;Bacillales;Bacillaceae 1;Bacillus
OTU_1149	Bacteria;Firmicutes;Clostridia;Clostridiales;Lachnospiraceae;Lachnospiracea_incertae_sedis
OTU_1150	Bacteria;Firmicutes;Clostridia;Clostridiales;Lachnospiraceae;Clostridium XlVa
OTU_1151	Bacteria;Actinobacteria;Actinobacteria;Coriobacteriales;Coriobacteriaceae;Cryptobacterium
OTU_1152	Bacteria;Proteobacteria;Gammaproteobacteria;Pasteurellales;Pasteurellaceae;Haemophilus
OTU_1153	Bacteria
OTU_1154	Bacteria;Firmicutes;Clostridia;Clostridiales;Clostridiaceae 1;Clostridium sensu stricto
OTU_1155	Bacteria;Firmicutes;Clostridia;Clostridiales;Ruminococcaceae
OTU_1156	Bacteria;Firmicutes;Clostridia;Clostridiales;Ruminococcaceae
OTU_1157	Bacteria;Firmicutes;Clostridia;Clostridiales;Syntrophomonadaceae;Syntrophomonas
OTU_1158	Bacteria;Firmicutes;Clostridia;Clostridiales;Lachnospiraceae;Clostridium XlVa
OTU_1159	Bacteria;Firmicutes;Clostridia;Clostridiales;Lachnospiraceae
OTU_1160	Bacteria;Proteobacteria;Alphaproteobacteria;Alphaproteobacteria_incertae_sedis;Geminicoccus;Geminicoccus
OTU_1161	Bacteria;Bacteroidetes;Bacteroidia;Bacteroidales;Prevotellaceae;Prevotella
OTU_1162	Bacteria;Firmicutes;Clostridia;Clostridiales;Lachnospiraceae;Butyrivibrio
OTU_1163	Bacteria;Firmicutes;Clostridia;Clostridiales
OTU_1164	Bacteria;Bacteroidetes;Bacteroidia;Bacteroidales;Prevotellaceae
OTU_1165	Bacteria;Bacteroidetes;Bacteroidia;Bacteroidales;Porphyromonadaceae;Barnesiella
OTU_1166	Bacteria;Firmicutes;Erysipelotrichia;Erysipelotrichales;Erysipelotrichaceae
OTU_1167	Bacteria;Actinobacteria;Actinobacteria;Actinomycetales;Actinomycetaceae;Mobiluncus
OTU_1168	Bacteria
OTU_1169	Bacteria
OTU_1170	Bacteria;Bacteroidetes;Bacteroidia;Bacteroidales;Porphyromonadaceae;Barnesiella
OTU_1171	Bacteria;Bacteroidetes;Bacteroidia;Bacteroidales;Bacteroidaceae;Bacteroides
OTU_1172	Bacteria;Firmicutes;Negativicutes;Selenomonadales;Veillonellaceae;Megamonas
OTU_1173	Bacteria;Bacteroidetes;Bacteroidia;Bacteroidales;Prevotellaceae;Prevotella
OTU_1174	Bacteria
OTU_1175	Bacteria;Firmicutes;Clostridia;Clostridiales;Ruminococcaceae
OTU_1176	Bacteria;Firmicutes;Clostridia;Clostridiales
OTU_1177	Bacteria;Firmicutes;Clostridia;Clostridiales
OTU_1178	Bacteria;Firmicutes;Clostridia;Clostridiales;Lachnospiraceae
OTU_1179	Bacteria;Firmicutes;Bacilli;Lactobacillales;Lactobacillaceae;Pediococcus
OTU_1180	Bacteria;Bacteroidetes;Bacteroidia;Bacteroidales;Porphyromonadaceae;Barnesiella
OTU_1181	Bacteria;Firmicutes;Clostridia;Clostridiales
OTU_1182	Bacteria
OTU_1183	Bacteria;Bacteroidetes;Sphingobacteria;Sphingobacteriales;Sphingobacteriaceae;Sphingobacterium
OTU_1184	Bacteria;Fusobacteria;Fusobacteria;Fusobacteriales;Fusobacteriaceae;Fusobacterium
OTU_1185	Bacteria;Aquificae;Aquificae;Aquificales;Aquificaceae;Hydrogenobacter
OTU_1186	Bacteria;Bacteroidetes;Bacteroidia;Bacteroidales;Porphyromonadaceae;Butyricimonas
OTU_1187	Bacteria;Firmicutes;Clostridia;Clostridiales;Ruminococcaceae

OTU_1188	Bacteria;Firmicutes;Clostridia;Clostridiales;Clostridiaceae 1;Clostridium sensu stricto
OTU_1189	Bacteria
OTU_1190	Bacteria
OTU_1191	Bacteria;Firmicutes;Clostridia;Clostridiales;Ruminococcaceae;Faecalibacterium
OTU_1192	Bacteria;Bacteroidetes;Flavobacteria;Flavobacteriales;Flavobacteriaceae;Planobacterium
OTU_1193	Bacteria;Bacteroidetes
OTU_1194	Bacteria;Firmicutes;Bacilli;Bacillales;Bacillales_Incertae Sedis XII;Exiguobacterium
OTU_1195	Bacteria;Bacteroidetes;Bacteroidia;Bacteroidales;Prevotellaceae;Prevotella
OTU_1196	Bacteria;Actinobacteria;Actinobacteria;Actinomycetales;Micrococcaceae;Arthrobacter
OTU_1197	Bacteria;Firmicutes;Bacilli;Lactobacillales;Lactobacillaceae;Lactobacillus
OTU_1198	Bacteria;Bacteroidetes;Bacteroidia;Bacteroidales;Porphyromonadaceae;Barnesiella
OTU_1199	Bacteria;Bacteroidetes;Bacteroidia;Bacteroidales;Porphyromonadaceae
OTU_1200	Bacteria;Bacteroidetes;Bacteroidia;Bacteroidales;Bacteroidaceae;Bacteroides
OTU_1201	Bacteria;Bacteroidetes;Bacteroidia;Bacteroidales;Bacteroidaceae;Bacteroides
OTU_1202	Bacteria;Actinobacteria;Actinobacteria;Actinomycetales;Actinomycetaceae;Actinomyces
OTU_1203	Bacteria;Firmicutes;Clostridia;Clostridiales;Ruminococcaceae;Saccharofermentans
OTU_1204	Bacteria;Firmicutes;Clostridia;Clostridiales
OTU_1205	Bacteria;Firmicutes
OTU_1206	Bacteria;Firmicutes;Clostridia;Clostridiales;Eubacteriaceae;Anaerofustis
OTU_1207	Bacteria;Firmicutes;Clostridia;Clostridiales;Ruminococcaceae;Oscillibacter
OTU_1208	Bacteria;Actinobacteria;Actinobacteria;Actinomycetales;Corynebacteriaceae;Corynebacterium
OTU_1209	Bacteria;Bacteroidetes;Bacteroidia;Bacteroidales;Prevotellaceae;Prevotella
OTU_1210	Bacteria;Bacteroidetes;Bacteroidia;Bacteroidales;Bacteroidaceae;Bacteroides
OTU_1211	Bacteria;Firmicutes;Clostridia;Clostridiales
OTU_1212	Bacteria;Proteobacteria;Alphaproteobacteria;Rhodobacterales;Rhodobacteraceae;Paracoccus
OTU_1213	Bacteria;Firmicutes;Clostridia;Clostridiales;Ruminococcaceae;Ruminococcus
OTU_1214	Bacteria
OTU_1215	Bacteria;Spirochaetes;Spirochaetes;Spirochaetales;Spirochaetaceae;Treponema
OTU_1216	Bacteria;Bacteroidetes;Bacteroidia;Bacteroidales;Bacteroidaceae;Bacteroides
OTU_1217	Bacteria;Bacteroidetes;Bacteroidia;Bacteroidales;Prevotellaceae;Prevotella
OTU_1218	Bacteria;Firmicutes;Clostridia;Clostridiales;Ruminococcaceae
OTU_1219	Bacteria;Bacteroidetes;Bacteroidia;Bacteroidales;Porphyromonadaceae;Butyricimonas
OTU_1220	Bacteria;Firmicutes;Erysipelotrichia;Erysipelotrichales;Erysipelotrichaceae;Bulleidia
OTU_1221	Bacteria;Firmicutes;Clostridia;Clostridiales
OTU_1222	Bacteria;Firmicutes;Clostridia;Clostridiales
OTU_1223	Bacteria;Firmicutes;Clostridia;Clostridiales;Ruminococcaceae;Hydrogenoanaerobacterium
OTU_1224	Bacteria
OTU_1225	Bacteria;Firmicutes;Clostridia;Clostridiales;Lachnospiraceae
OTU_1226	Bacteria;Actinobacteria;Actinobacteria;Rubrobacterales;Rubrobacteraceae;Rubrobacter
OTU_1227	Bacteria;Firmicutes;Clostridia;Clostridiales;Lachnospiraceae;Clostridium XlVa
OTU_1228	Bacteria;TM7;TM7_genera_incertae_sedis;TM7_genera_incertae_sedis;TM7_genera_incertae_sedis;TM7_genera_incertae_sedis
OTU_1229	Bacteria;Bacteroidetes;Bacteroidia;Bacteroidales;Bacteroidaceae;Bacteroides
OTU_1230	Bacteria
OTU_1231	Bacteria;Bacteroidetes;Bacteroidia;Bacteroidales;Prevotellaceae;Prevotella
OTU_1232	Bacteria;Proteobacteria;Deltaproteobacteria;Myxococcales
OTU_1233	Bacteria;Firmicutes;Clostridia;Clostridiales;Clostridiales_Incertae Sedis XI;Tepidimicrobium
OTU_1234	Bacteria;Bacteroidetes;Bacteroidia;Bacteroidales;Prevotellaceae;Prevotella
OTU_1235	Bacteria;Bacteroidetes;Flavobacteria;Flavobacteriales;Flavobacteriaceae;Capnocytophaga
OTU_1236	Bacteria
OTU_1237	Bacteria;Proteobacteria;Betaproteobacteria;Burkholderiales;Burkholderiaceae;Limnobacter
OTU_1238	Bacteria;Firmicutes;Clostridia;Clostridiales;Ruminococcaceae

OTU_1239	Unclassified
OTU_1240	Bacteria;Bacteroidetes;Bacteroidia;Bacteroidales;Prevotellaceae;Prevotella
OTU_1241	Bacteria
OTU_1242	Bacteria;Synergistetes;Synergistia;Synergistales;Synergistaceae;Cloacibacillus
OTU_1243	Bacteria;Firmicutes;Clostridia;Clostridiales;Lachnospiraceae;Dorea
OTU_1244	Bacteria;Proteobacteria
OTU_1245	Bacteria
OTU_1246	Bacteria;Firmicutes;Clostridia;Clostridiales;Peptococcaceae 1;Peptococcus
OTU_1247	Bacteria;Firmicutes;Clostridia;Clostridiales;Lachnospiraceae
OTU_1248	Bacteria
OTU_1249	Bacteria;Firmicutes;Clostridia;Clostridiales;Lachnospiraceae
OTU_1250	Bacteria;Firmicutes
OTU_1251	Bacteria;Lentisphaerae;Lentisphaeria;Victivallales;Victivallaceae;Victivallis
OTU_1252	Bacteria;Firmicutes;Clostridia;Clostridiales;Ruminococcaceae;Clostridium IV
OTU_1253	Bacteria;Firmicutes;Clostridia;Clostridiales;Ruminococcaceae
OTU_1254	Bacteria;Firmicutes;Negativicutes;Selenomonadales;Veillonellaceae;Selenomonas
OTU_1255	Bacteria;Bacteroidetes;Bacteroidia;Bacteroidales;Porphyromonadaceae;Barnesiella
OTU_1256	Bacteria;Bacteroidetes;Bacteroidia;Bacteroidales;Porphyromonadaceae;Barnesiella
OTU_1257	Bacteria;Bacteroidetes;Bacteroidia;Bacteroidales;Porphyromonadaceae
OTU_1258	Bacteria
OTU_1259	Bacteria;Firmicutes;Clostridia;Clostridiales;Lachnospiraceae;Coprococcus
OTU_1260	Bacteria;Firmicutes;Clostridia;Clostridiales;Ruminococcaceae;Oscillibacter
OTU_1261	Bacteria;Fusobacteria;Fusobacteria;Fusobacteriales;Leptotrichiaceae;Leptotrichia
OTU_1262	Bacteria;Firmicutes;Clostridia;Clostridiales;Ruminococcaceae;Hydrogenoanaerobacterium
OTU_1263	Bacteria
OTU_1264	Bacteria;Firmicutes;Clostridia;Clostridiales
OTU_1265	Bacteria;Gemmatimonadetes;Gemmatimonadetes;Gemmatimonadales;Gemmatimonadaceae;Gemmatimonas
OTU_1266	Bacteria;Bacteroidetes;Bacteroidia;Bacteroidales;Porphyromonadaceae;Barnesiella
OTU_1267	Bacteria;Firmicutes;Clostridia;Clostridiales;Clostridiales_Incertae Sedis XIII;Mogibacterium
OTU_1268	Bacteria;Proteobacteria;Gammaproteobacteria;Pseudomonadales;Moraxellaceae;Acinetobacter
OTU_1269	Bacteria;Bacteroidetes;Bacteroidia;Bacteroidales;Bacteroidaceae;Anaerorhabdus
OTU_1270	Bacteria;Firmicutes;Clostridia;Clostridiales;Ruminococcaceae;Faecalibacterium
OTU_1271	Bacteria;Actinobacteria;Actinobacteria;Actinomycetales;Geodermatophilaceae;Blastococcus
OTU_1272	Bacteria;Firmicutes;Negativicutes;Selenomonadales;Veillonellaceae;Megamonas
OTU_1273	Bacteria
OTU_1274	Bacteria;Firmicutes;Clostridia;Clostridiales;Ruminococcaceae
OTU_1275	Bacteria;Firmicutes;Clostridia;Clostridiales;Lachnospiraceae;Coprococcus
OTU_1276	Bacteria
OTU_1277	Bacteria;Firmicutes;Clostridia;Clostridiales;Ruminococcaceae
OTU_1278	Bacteria;Actinobacteria;Actinobacteria;Coriobacteriales;Coriobacteriaceae
OTU_1279	Bacteria;Firmicutes;Clostridia;Clostridiales;Ruminococcaceae
OTU_1280	Bacteria;Firmicutes;Clostridia;Clostridiales;Ruminococcaceae
OTU_1281	Bacteria;Firmicutes;Clostridia;Clostridiales;Ruminococcaceae;Oscillibacter
OTU_1282	Bacteria;Bacteroidetes;Bacteroidia;Bacteroidales;Prevotellaceae;Prevotella
OTU_1283	Bacteria;Proteobacteria;Deltaproteobacteria;Bdellovibrionales;Bdellovibrionaceae;Vampirovibrio
OTU_1284	Bacteria;Bacteroidetes;Bacteroidia;Bacteroidales;Prevotellaceae;Prevotella
OTU_1285	Bacteria;Proteobacteria;Deltaproteobacteria
OTU_1286	Bacteria;Firmicutes;Clostridia;Clostridiales;Ruminococcaceae;Sporobacter
OTU_1287	Bacteria;Firmicutes
OTU_1288	Bacteria;Bacteroidetes;Bacteroidia;Bacteroidales;Prevotellaceae;Prevotella
OTU_1289	Bacteria;Proteobacteria;Gammaproteobacteria;Pseudomonadales;Moraxellaceae;Acinetobacter
OTU_1290	Bacteria;Firmicutes;Erysipelotrichia;Erysipelotrichales;Erysipelotrichaceae;Allobaculum

OTU_1291	Bacteria;Proteobacteria;Gammaproteobacteria;Alteromonadales;Shewanellaceae;Shewanella
OTU_1292	Bacteria;Fusobacteria;Fusobacteria;Fusobacteriales;Leptotrichiaceae;Leptotrichia
OTU_1293	Bacteria;Firmicutes;Bacilli;Lactobacillales;Aerococcaceae;Aerococcus
OTU_1294	Bacteria;Firmicutes;Clostridia;Clostridiales;Ruminococcaceae;Faecalibacterium
OTU_1295	Bacteria;Bacteroidetes;Bacteroidia;Bacteroidales;Bacteroidaceae;Bacteroides
OTU_1296	Bacteria;Firmicutes;Negativicutes;Selenomonadales;Veillonellaceae;Dialister

supplementary Table S3 Fecal microbial diversity index in all samples

Sample	Group	Avaliable Reads	Shannon	Simpson	Invsimpson
zfh001	HCC Group	36428	2.986264925	0.909416613	11.039552
zfh003	HCC Group	38713	3.462691312	0.945368913	18.304597
zfh004	HCC Group	29048	3.303763397	0.93627116	15.691483
zfh005	HCC Group	31120	3.178135974	0.918809385	12.316694
zfh006	HCC Group	31679	3.180497813	0.929735349	14.231907
zfh007	HCC Group	36418	3.534248988	0.943290211	17.63364
zfh009	HCC Group	27445	3.005475682	0.891302724	9.1998626
zfh010	HCC Group	32909	3.584307123	0.948896042	19.567956
zfh011	HCC Group	35982	3.114094257	0.903043185	10.31387
zfh012	HCC Group	24238	3.09236115	0.902766867	10.28456
zfh013	HCC Group	36864	3.253750341	0.911554416	11.306387
zfh014	HCC Group	27912	1.811258419	0.637369555	2.7576284
zfh015	HCC Group	61844	3.86633124	0.96216244	26.428763
zfh016	HCC Group	25984	2.457766658	0.805933786	5.1528804
zfh017	HCC Group	30392	2.41602512	0.816071594	5.4368981
zfh018	HCC Group	25872	0.857009957	0.327225126	1.4863813
zfh019	HCC Group	23516	3.724698773	0.941545298	17.107264
zfh020	HCC Group	39454	3.308942901	0.904053516	10.422477
zfh021	HCC Group	26727	2.833131505	0.89710416	9.7185659
zfh022	HCC Group	30314	2.741602842	0.880526583	8.3700628
zfh023	HCC Group	50627	3.472303768	0.928616506	14.008841
zfh024	HCC Group	60261	2.140814139	0.784863263	4.6482066
zfh025	HCC Group	28351	3.621498911	0.956543179	23.011347
zfh026	HCC Group	31402	3.692749966	0.955126677	22.284955
zfh027	HCC Group	26654	3.075219892	0.886468964	8.8081641
zfh028	HCC Group	37003	2.399668918	0.800402126	5.0100734
zfh029	HCC Group	40343	3.18998364	0.915223314	11.795696
zfh030	HCC Group	75015	3.473065877	0.931225058	14.54018
zfh031	HCC Group	66149	2.734937969	0.869449649	7.6598799
zfh032	HCC Group	22789	3.099742936	0.886516781	8.8118755
zfh033	HCC Group	31685	2.454198083	0.812289561	5.3273542
zfh034	HCC Group	31914	2.523944864	0.834582861	6.0453228
zfh035	HCC Group	24824	3.094474897	0.911055058	11.24291
zfh037	HCC Group	27199	2.989393564	0.89575718	9.5929869
zfh038	HCC Group	36002	2.185217572	0.726568633	3.6572249
zfh041	HCC Group	55931	2.906461433	0.899296226	9.9301144
zfh042	HCC Group	62355	2.630621793	0.874879767	7.9923125
zfh044	HCC Group	13236	2.865988157	0.889253411	9.0296234
zfh045	HCC Group	16897	2.485430995	0.750457776	4.0073378
zfh046	HCC Group	16013	2.981769509	0.836726067	6.1246764
zfh101	HCC Group	25506	2.414697241	0.823100643	5.6529318
zfh102	HCC Group	29724	3.495796361	0.948746203	19.51075
zfh103	HCC Group	33973	3.046471945	0.887437203	8.8839299
zfh104	HCC Group	27448	3.460750109	0.937323607	15.95497
zfh105	HCC Group	24799	2.338083167	0.866572753	7.4947211
zfh106	HCC Group	26984	4.108642568	0.971400905	34.966141
zfh107	HCC Group	27393	2.29378721	0.774189347	4.428489
zfh108	HCC Group	27501	2.731540306	0.83324623	5.9968659
zfh109	HCC Group	29320	2.309784785	0.827188665	5.7866575
zfh110	HCC Group	42792	2.757703625	0.865397567	7.4292862

zfh111	HCC Group	62487	2.338180997	0.80346348	5.0881129
zfh112	HCC Group	29164	2.494083181	0.837523672	6.1547427
zfh113	HCC Group	41734	3.786379334	0.958670286	24.195667
zfh114	HCC Group	52534	3.14350184	0.924845035	13.305841
zfh115	HCC Group	59174	1.817582436	0.744542319	3.9145427
zfh116	HCC Group	53297	2.871294954	0.839290699	6.2224152
zfh117	HCC Group	27215	3.069849705	0.918885386	12.328235
zfh118	HCC Group	42908	3.853037966	0.965120727	28.670322
zfh119	HCC Group	54317	2.866652265	0.887409692	8.8817591
zfh120	HCC Group	61509	2.703970876	0.853406425	6.8215814
zfh121	HCC Group	49917	3.160500138	0.916918452	12.036367
zfh122	HCC Group	56950	3.527503673	0.94182415	17.189263
zfh123	HCC Group	60454	2.921311289	0.897925728	9.796788
zfh124	HCC Group	32689	2.845342839	0.902810385	10.289165
zfh125	HCC Group	55080	2.412392106	0.849067575	6.6254816
zfh126	HCC Group	41450	2.239723239	0.800199398	5.0049899
zfh127	HCC Group	28086	3.843637515	0.956147731	22.803837
zfh128	HCC Group	34008	4.010281209	0.969001373	32.259493
zfh129	HCC Group	26844	3.306106827	0.941103474	16.97893
zfh130	HCC Group	36169	3.253835251	0.925307371	13.388202
zfh131	HCC Group	37210	3.269136919	0.935823887	15.582122
zfh132	HCC Group	55348	3.693539205	0.949472939	19.791375
zfh133	HCC Group	30907	3.490762247	0.927854728	13.860922
zfh134	HCC Group	37671	3.064890407	0.900614115	10.061791
zfh135	HCC Group	26496	3.184602115	0.874479609	7.9668331
zfh136	HCC Group	31889	2.303506601	0.769275214	4.3341681
zfh137	HCC Group	38922	3.212908159	0.934424508	15.2496
zfh138	HCC Group	33557	3.338594309	0.938567158	16.277938
zfh139	HCC Group	21736	3.545086813	0.948802794	19.532316
zfh140	HCC Group	36804	2.175374118	0.764742065	4.2506536
zfh141	HCC Group	16073	2.100728329	0.7928953	4.8284756
zfh142	HCC Group	28560	4.083286617	0.968867054	32.120314
zfh143	HCC Group	33013	3.467016285	0.941132069	16.987178
zfh144	HCC Group	28673	2.790928496	0.896222579	9.6360075
zfh145	HCC Group	29475	3.016424137	0.885959768	8.7688352
zfh146	HCC Group	31370	3.439254009	0.947879085	19.186156
zfh147	HCC Group	33629	2.481325951	0.823469043	5.6647288
zfh148	HCC Group	42832	3.40367634	0.915087382	11.776813
zfh149	HCC Group	24370	2.954746272	0.899075495	9.9083964
zfh150	HCC Group	38856	3.004271379	0.915668209	11.857924
zfh151	HCC Group	62092	2.11175707	0.823668317	5.6711306
zfh152	HCC Group	62024	2.732445758	0.839814483	6.2427616
zfh153	HCC Group	19446	2.170922322	0.809995725	5.2630395
zfh154	HCC Group	27226	3.081698326	0.910937269	11.228041
zfh155	HCC Group	29725	2.969229775	0.910038223	11.115832
zfh156	HCC Group	32155	1.953670721	0.70938971	3.4410344
zfh157	HCC Group	29569	2.951087157	0.888916949	9.0022734
zfh158	HCC Group	21788	2.893872497	0.836377391	6.1116248
zfh159	HCC Group	29252	2.905352004	0.896660296	9.6768228
zfh160	HCC Group	28885	2.912195541	0.87867499	8.2423237
zfh161	HCC Group	27811	2.644509563	0.864477209	7.3788327
zfh162	HCC Group	19316	3.033847787	0.922539655	12.909831

zfh163	HCC Group	26398	2.889286156	0.859662214	7.1256646
zfh164	HCC Group	26876	3.029167022	0.914677898	11.720293
zfh165	HCC Group	30643	2.806855097	0.838763848	6.202083
zfh166	HCC Group	26146	3.273564026	0.915367657	11.815814
zfh167	HCC Group	24383	1.866395392	0.711075188	3.4611081
zfh168	HCC Group	26073	2.402006549	0.771983131	4.3856404
zfh169	HCC Group	27516	2.63263747	0.830331477	5.8938451
zfh170	HCC Group	30487	3.011706158	0.880476426	8.3665503
zfh171	HCC Group	28351	3.087192098	0.8963286	9.6458618
zfh172	HCC Group	26386	3.58124258	0.951826251	20.758194
zfh173	HCC Group	26370	2.994546458	0.923236075	13.026952
zfh201	Healthy Control	30213	2.175964861	0.79084432	4.7811276
zfh202	Healthy Control	28647	3.397634792	0.943077502	17.567746
zfh203	Healthy Control	28475	4.484016813	0.978834192	47.24601
zfh204	Healthy Control	36065	2.919299003	0.885970321	8.7696467
zfh205	Healthy Control	61844	3.866331351	0.962162438	26.428764
zfh206	Healthy Control	29789	2.566255338	0.835632757	6.0839373
zfh207	Healthy Control	67672	3.785527592	0.957740215	23.663159
zfh208	Healthy Control	32313	3.150888378	0.92374456	13.113818
zfh209	Healthy Control	31106	2.669129804	0.8576248	7.0236952
zfh210	Healthy Control	38057	3.43740984	0.949469278	19.789941
zfh211	Healthy Control	29769	3.44555668	0.925635296	13.44724
zfh212	Healthy Control	29343	3.591973306	0.937420678	15.979719
zfh213	Healthy Control	39455	3.567670049	0.946955158	18.851974
zfh214	Healthy Control	36469	4.046688589	0.966952495	30.259471
zfh215	Healthy Control	31497	3.3121965	0.921730948	12.776442
zfh216	Healthy Control	38269	3.859747795	0.958356507	24.013355
zfh217	Healthy Control	36175	3.591445237	0.93844859	16.246581
zfh218	Healthy Control	31203	3.658482563	0.93226971	14.764443
zfh219	Healthy Control	27635	3.938002759	0.934443897	15.25411
zfh220	Healthy Control	39982	3.299413513	0.924310202	13.21182
zfh221	Healthy Control	34944	4.423304856	0.977225647	43.90904
zfh222	Healthy Control	33303	2.081926839	0.743916334	3.9049738
zfh223	Healthy Control	35520	3.479112613	0.93291	14.905351
zfh224	Healthy Control	30030	2.838034578	0.88924176	9.0286736
zfh225	Healthy Control	31640	3.997895703	0.957197894	23.363336
zfh226	Healthy Control	28601	3.011889204	0.915425422	11.823884
zfh227	Healthy Control	40641	2.780178952	0.866180204	7.4727359
zfh228	Healthy Control	36406	3.461840245	0.945189834	18.24479
zfh229	Healthy Control	31771	3.023176143	0.876220826	8.0789035
zfh230	Healthy Control	38630	2.327840475	0.835975826	6.0966623
zfh231	Healthy Control	65878	3.402874845	0.941661106	17.141223
zfh232	Healthy Control	54843	2.629726655	0.828336572	5.8253526
zfh233	Healthy Control	58724	3.49629967	0.949259639	19.708177
zfh234	Healthy Control	66600	3.565057098	0.952307245	20.967545
zfh235	Healthy Control	61465	4.291641785	0.974606704	39.380473
zfh236	Healthy Control	68195	4.126305791	0.970998249	34.480677
zfh237	Healthy Control	67479	2.241782658	0.780403184	4.5538001
zfh238	Healthy Control	65698	3.485611774	0.938179099	16.175759
zfh239	Healthy Control	69888	3.889794847	0.965374668	28.880589
zfh240	Healthy Control	36721	3.220091858	0.933267198	14.985134
zfh241	Healthy Control	53444	2.827376527	0.88363332	8.5935252

zfh242	Healthy Control	74358	3.137652465	0.879663844	8.3100544
zfh243	Healthy Control	39632	3.252894871	0.926762251	13.654161
zfh244	Healthy Control	68666	2.536033652	0.831545433	5.9363187
zfh245	Healthy Control	66239	3.316705292	0.909432	11.041427
zfh246	Healthy Control	64960	2.616256311	0.830383368	5.8956483
zfh247	Healthy Control	66359	3.157848437	0.913257811	11.528416
zfh248	Healthy Control	72149	2.409630008	0.790630313	4.7762406
zfh249	Healthy Control	39675	4.157111293	0.972827941	36.80251
zfh250	Healthy Control	63797	3.215871105	0.934359682	15.234539
zfh251	Healthy Control	66966	2.291394068	0.73531349	3.7780543
zfh252	Healthy Control	71987	2.74366611	0.820717712	5.5777958
zfh253	Healthy Control	61599	3.298719773	0.933949121	15.139844
zfh254	Healthy Control	67768	2.920491255	0.901831751	10.186593
zfh255	Healthy Control	66889	2.951524782	0.901305033	10.132229
zfh256	Healthy Control	58354	3.447626228	0.945437345	18.327554
zfh257	Healthy Control	57602	3.126379368	0.921613091	12.757232
zfh258	Healthy Control	68619	3.343109704	0.942164323	17.290366
zfh259	Healthy Control	58996	2.286175312	0.798218949	4.9558667
zfh260	Healthy Control	30475	3.335953291	0.932545999	14.824917
zfh261	Healthy Control	30088	2.361999465	0.804961314	5.1271879
zfh262	Healthy Control	25128	2.303458866	0.81048849	5.2767244
zfh263	Healthy Control	34215	2.611026936	0.856243719	6.9562178
zfh264	Healthy Control	30159	2.704904405	0.869531583	7.6646902
zfh265	Healthy Control	21790	2.996306579	0.899783384	9.9783853
zfh266	Healthy Control	25953	3.086151235	0.903389237	10.350814
zfh267	Healthy Control	25088	3.43458067	0.922254074	12.86241
zfh268	Healthy Control	30667	3.052228805	0.893425136	9.3830755
zfh269	Healthy Control	24908	2.893766037	0.892091186	9.2670836
zfh270	Healthy Control	35747	2.552555195	0.80399273	5.1018516
zfh271	Healthy Control	21482	3.489580744	0.906037854	10.642584
zfh272	Healthy Control	21217	3.047112785	0.891309786	9.2004603
zfh273	Healthy Control	24976	2.647073184	0.834539366	6.0437336
zfh274	Healthy Control	26388	2.830521335	0.868845072	7.6245705
zfh275	Healthy Control	26534	2.942359946	0.915184975	11.790364
zfh276	Healthy Control	26843	2.941930349	0.909524882	11.052763
zfh277	Healthy Control	24107	1.975167406	0.622892838	2.6517661
zfh278	Healthy Control	34119	3.530483803	0.933830564	15.112718
zfh279	Healthy Control	27461	3.310059887	0.942907793	17.515525
zfh280	Healthy Control	32278	2.835964981	0.87355073	7.9083098
zfh281	Healthy Control	34330	4.269315373	0.972993895	37.028665
zfh282	Healthy Control	33285	2.489319975	0.855623734	6.9263462
zfh283	Healthy Control	20621	2.843235002	0.905206081	10.5492
zfh284	Healthy Control	24743	3.203926787	0.896658109	9.6766179
zfh285	Healthy Control	24669	3.710084494	0.955977205	22.715504
zfh286	Healthy Control	31417	1.624494732	0.645987079	2.8247557
zfh287	Healthy Control	25168	2.154148327	0.812054813	5.3207002
zfh288	Healthy Control	34269	1.934848112	0.770331179	4.3540956
zfh289	Healthy Control	28910	2.167070935	0.776032602	4.4649356
zfh290	Healthy Control	26367	4.060923694	0.958551144	24.126118
zfh291	Healthy Control	29810	2.559817303	0.875401346	8.0257689
zfh292	Healthy Control	27642	3.166723259	0.901131129	10.114407
zfh293	Healthy Control	27332	2.642108983	0.847115098	6.5408683

zfh294	Healthy Control	33053	1.869896507	0.721445819	3.5899659
zfh295	Healthy Control	25749	4.053959404	0.966959767	30.26613
zfh296	Healthy Control	24145	3.167755579	0.912089255	11.375174
zfh297	Healthy Control	21680	2.748942519	0.862368944	7.265802
zfh298	Healthy Control	22029	2.775864898	0.880691713	8.3816475
zfh299	Healthy Control	33551	2.24625016	0.837225014	6.1434501
zfh300	Healthy Control	28970	3.263987506	0.888769406	8.9903323

supplementary Table S4 three hundred and ten OTUs from all samples

		mean(HCC Group)	mean(Healthy Control)
OTU_0001	Bacteria;Bacteroidetes;Bacteroidia;Bacteroidales;Prevotellaceae;Prevotella	0.064846022	0.08115785
OTU_0002	Bacteria;Bacteroidetes;Bacteroidia;Bacteroidales;Bacteroidaceae;Bacteroides	0.091025664	0.063073737
OTU_0003	Bacteria;Bacteroidetes;Bacteroidia;Bacteroidales;Bacteroidaceae;Bacteroides	0.070753657	0.039265511
OTU_0004	Bacteria;Proteobacteria;Gammaproteobacteria;Enterobacteriales;Enterobacteriaceae;Escherichia/Shigella	0.026492666	0.010627557
OTU_0005	Bacteria;Firmicutes;Negativicutes;Selenomonadales;Acidaminococcaceae;Phascolarctobacterium	0.01948616	0.020537281
OTU_0006	Bacteria;Firmicutes;Clostridia;Clostridiales;Ruminococcaceae;Faecalibacterium	0.044300553	0.056541293
OTU_0007	Bacteria;Firmicutes;Clostridia;Clostridiales;Lachnospiraceae;Roseburia	0.021299887	0.020766096
OTU_0008	Bacteria;Fusobacteria;Fusobacteria;Fusobacteriales;Fusobacteriaceae;Fusobacterium	0.017774976	0.02703951
OTU_0009	Bacteria;Proteobacteria;Alphaproteobacteria;Rhizobiales;Hyphomicrobiaceae;Gemmiger	0.012625537	0.015511797
OTU_0010	Bacteria;Bacteroidetes;Bacteroidia;Bacteroidales;Bacteroidaceae;Bacteroides	0.013580916	0.00823053
OTU_0011	Bacteria;Bacteroidetes;Bacteroidia;Bacteroidales;Bacteroidaceae;Bacteroides	0.013978312	0.021426322
OTU_0012	Bacteria;Firmicutes;Clostridia;Clostridiales;Ruminococcaceae;Clostridium IV	0.000740442	0.001015402
OTU_0013	Bacteria;Firmicutes;Clostridia;Clostridiales;Lachnospiraceae;Lachnospiracea_incertae_sedis	0.013922405	0.006071274
OTU_0014	Bacteria;Bacteroidetes;Bacteroidia;Bacteroidales;Rikenellaceae;Alistipes	0.006449561	0.013674945
OTU_0015	Bacteria;Firmicutes;Clostridia;Clostridiales;Lachnospiraceae;Roseburia	0.014671965	0.005963483
OTU_0016	Bacteria;Bacteroidetes;Bacteroidia;Bacteroidales;Bacteroidaceae;Bacteroides	0.017370555	0.014238826
OTU_0017	Bacteria;Bacteroidetes;Bacteroidia;Bacteroidales;Prevotellaceae;Prevotella	0.001498972	0.000221444
OTU_0019	Bacteria;Firmicutes;Bacilli;Lactobacillales;Streptococcaceae;Streptococcus	0.006080786	0.000516901
OTU_0020	Bacteria;Firmicutes;Clostridia;Clostridiales;Lachnospiraceae;Lachnospiracea_incertae_sedis	0.010031969	0.004215576
OTU_0021	Bacteria;Bacteroidetes;Bacteroidia;Bacteroidales;Bacteroidaceae;Bacteroides	0.013038354	0.021234035
OTU_0022	Bacteria;Firmicutes;Clostridia;Clostridiales;Ruminococcaceae	0.000820553	0.000964836
OTU_0023	Bacteria;Firmicutes;Clostridia;Clostridiales;Lachnospiraceae;Lachnospiracea_incertae_sedis	0.010127367	0.006507928
OTU_0024	Bacteria;Firmicutes;Clostridia;Clostridiales;Lachnospiraceae;Lachnospiracea_incertae_sedis	0.00484136	0.004188995
OTU_0025	Bacteria;Bacteroidetes;Bacteroidia;Bacteroidales;Porphyromonadaceae;Parabacteroides	0.0035018	0.007388043
OTU_0026	Bacteria;Bacteroidetes;Bacteroidia;Bacteroidales;Rikenellaceae;Alistipes	0.003088197	0.003768359
OTU_0027	Bacteria;Firmicutes;Clostridia;Clostridiales;Ruminococcaceae;Ruminococcus	0.008212746	0.006398791
OTU_0028	Bacteria;Firmicutes;Negativicutes;Selenomonadales;Veillonellaceae;Dialister	0.000649995	0.010485796
OTU_0029	Bacteria;Firmicutes;Clostridia;Clostridiales;Ruminococcaceae;Oscillibacter	0.002396102	0.005123173
OTU_0030	Bacteria;Bacteroidetes;Bacteroidia;Bacteroidales;Bacteroidaceae;Bacteroides	0.013309769	0.011007818
OTU_0031	Bacteria;Firmicutes;Clostridia;Clostridiales;Peptostreptococcaceae;Clostridium XI	0.002806111	0.001225265
OTU_0032	Bacteria;Bacteroidetes;Bacteroidia;Bacteroidales;Bacteroidaceae;Bacteroides	0.005755971	0.004462147
OTU_0033	Bacteria;Firmicutes;Clostridia;Clostridiales;Lachnospiraceae;Clostridium XlVa	0.005935843	0.004379345
OTU_0034	Bacteria;Firmicutes;Negativicutes;Selenomonadales;Veillonellaceae;Megamonas	0.006719248	0.004075958
OTU_0035	Bacteria;Actinobacteria;Actinobacteria;Bifidobacteriales;Bifidobacteriaceae;Bifidobacterium	0.00190321	0.002250911
OTU_0036	Bacteria;Firmicutes;Negativicutes;Selenomonadales;Veillonellaceae;Dialister	0.001139217	0.001141022
OTU_0037	Bacteria;Proteobacteria;Betaproteobacteria;Burkholderiales;Sutterellaceae;Parasutterella	0.010714736	0.011599884
OTU_0038	Bacteria;Firmicutes;Erysipelotrichia;Erysipelotrichales;Erysipelotrichaceae;Clostridium XVIII	0.004474059	0.004203307
OTU_0039	Bacteria;Actinobacteria;Actinobacteria;Bifidobacteriales;Bifidobacteriaceae;Bifidobacterium	0.001952376	0.003778728
OTU_0040	Bacteria;Proteobacteria;Gammaproteobacteria;Pasteurellales;Pasteurellaceae;Haemophilus	0.003604775	0.00132402
OTU_0041	Bacteria;Firmicutes;Clostridia;Clostridiales;Clostridiaceae 1;Clostridium sensu stricto	0.001072333	0.00100016
OTU_0042	Bacteria;Firmicutes;Clostridia;Clostridiales;Lachnospiraceae;Clostridium XlVa	0.0053022	0.004071755
OTU_0043	Bacteria;Firmicutes;Clostridia;Clostridiales;Lachnospiraceae;Blautia	0.002548409	0.002851855
OTU_0044	Bacteria;Firmicutes;Clostridia;Clostridiales;Ruminococcaceae	0.000627744	0.003355146
OTU_0045	Bacteria;Proteobacteria;Betaproteobacteria;Burkholderiales;Sutterellaceae;Sutterella	0.006843681	0.00447108
OTU_0047	Bacteria;Firmicutes;Clostridia;Clostridiales;Lachnospiraceae	0.002805254	0.002489621
OTU_0048	Bacteria;Firmicutes	0.002263296	0.00445799
OTU_0049	Bacteria;Verrucomicrobia;Verrucomicrobiae;Verrucomicrobiales;Verrucomicrobiaceae;Akkermansia	0.000972517	0.003650033
OTU_0050	Bacteria;Firmicutes;Negativicutes;Selenomonadales;Veillonellaceae;Veillonella	0.002992899	0.002070846

OTU_0051	Bacteria;Firmicutes;Clostridia;Clostridiales;Ruminococcaceae;Oscillibacter	0.002570525	0.004017273
OTU_0052	Bacteria;Firmicutes;Clostridia;Clostridiales;Lachnospiraceae;Coprococcus	0.002246933	0.001632225
OTU_0053	Bacteria;Bacteroidetes;Bacteroidia;Bacteroidales;Porphyromonadaceae	0.004035428	0.002258448
OTU_0054	Bacteria;Firmicutes;Clostridia;Clostridiales;Ruminococcaceae;Ruminococcus	0.00184211	0.002204859
OTU_0055	Bacteria;Proteobacteria;Deltaproteobacteria;Desulfovibrionales;Desulfovibrionaceae;Bilophila	0.002255449	0.003640792
OTU_0056	Bacteria;Firmicutes;Clostridia;Clostridiales;Ruminococcaceae	0.000507646	0.000992927
OTU_0057	Bacteria;Firmicutes;Clostridia;Clostridiales;Ruminococcaceae	0.001233193	0.001590307
OTU_0058	Bacteria;Firmicutes;Clostridia;Clostridiales;Ruminococcaceae	0.000611804	0.002012422
OTU_0059	Bacteria;Firmicutes;Clostridia;Clostridiales;Ruminococcaceae;Clostridium IV	0.000934848	0.002651865
OTU_0060	Bacteria;Proteobacteria;Betaproteobacteria;Burkholderiales;Sutterellaceae	0.002612495	0.003870578
OTU_0061	Bacteria;Firmicutes;Clostridia;Clostridiales;Ruminococcaceae;Flavonifractor	0.002125527	0.002676237
OTU_0063	Bacteria;Proteobacteria;Gammaproteobacteria;Enterobacteriales;Enterobacteriaceae	0.005361142	0.000447827
OTU_0064	Bacteria;Firmicutes;Clostridia;Clostridiales;Lachnospiraceae;Clostridium XlVa	0.001885559	0.001418344
OTU_0065	Bacteria;Firmicutes;Erysipelotrichia;Erysipelotrichales;Erysipelotrichaceae;Erysipelotrichaceae_incertae_sedis	0.001834372	0.004058497
OTU_0066	Bacteria;Bacteroidetes;Bacteroidia;Bacteroidales;Porphyromonadaceae	0.00039391	0.000928238
OTU_0067	Bacteria;Firmicutes;Negativicutes;Selenomonadales;Acidaminococcaceae;Phascolarctobacterium	0.004561478	0.005138407
OTU_0068	Bacteria;Firmicutes;Clostridia;Clostridiales;Lachnospiraceae;Dorea	0.000965844	0.001681803
OTU_0069	Bacteria;Bacteroidetes;Bacteroidia;Bacteroidales;Prevotellaceae;Prevotella	0.000831634	0.00034438
OTU_0070	Bacteria;Firmicutes;Clostridia;Clostridiales;Lachnospiraceae;Coprococcus	0.002068931	0.005483803
OTU_0071	Bacteria;Bacteroidetes;Bacteroidia;Bacteroidales;Prevotellaceae;Paraprevotella	0.003751517	0.001805687
OTU_0073	Bacteria;Bacteroidetes;Bacteroidia;Bacteroidales;Bacteroidaceae;Bacteroides	0.001713961	0.000869964
OTU_0074	Bacteria;Firmicutes;Clostridia;Clostridiales;Ruminococcaceae;Sporobacter	0.000806783	0.0020408
OTU_0075	Bacteria;Bacteroidetes;Bacteroidia;Bacteroidales;Porphyromonadaceae;Parabacteroides	0.00511826	0.006146538
OTU_0076	Bacteria;Bacteroidetes;Bacteroidia;Bacteroidales;Bacteroidaceae;Bacteroides	0.002058683	0.004040034
OTU_0077	Bacteria;Bacteroidetes;Bacteroidia;Bacteroidales;Porphyromonadaceae;Odoribacter	0.002108302	0.002532242
OTU_0078	Bacteria;Proteobacteria;Betaproteobacteria;Burkholderiales;Sutterellaceae;Sutterella	0.00071131	0.005161424
OTU_0079	Bacteria;Firmicutes;Clostridia;Clostridiales;Ruminococcaceae	0.000779689	0.000592216
OTU_0080	Bacteria;Bacteroidetes;Bacteroidia;Bacteroidales;Rikenellaceae;Alistipes	0.002090151	0.0044243
OTU_0082	Bacteria;Proteobacteria;Deltaproteobacteria;Desulfovibrionales;Desulfovibrionaceae;Desulfovibrio	0.000996762	0.001863541
OTU_0083	Bacteria;Firmicutes;Clostridia;Clostridiales	0.000429055	0.001864088
OTU_0084	Bacteria;Firmicutes;Clostridia;Clostridiales;Ruminococcaceae;Acetanaerobacterium	0.000578064	0.001940588
OTU_0085	Bacteria;Firmicutes;Clostridia;Clostridiales;Lachnospiraceae;Clostridium XlVa	0.004283276	0.002555874
OTU_0086	Bacteria;Firmicutes;Clostridia;Clostridiales;Lachnospiraceae;Lachnospiracea_incertae_sedis	0.009380018	0.005733526
OTU_0087	Bacteria;Actinobacteria;Actinobacteria;Coriobacteriales;Coriobacteriaceae;Collinsella	0.001259152	0.001206086
OTU_0088	Bacteria;Firmicutes;Clostridia;Clostridiales;Lachnospiraceae;Lachnospiracea_incertae_sedis	0.001840918	0.001215458
OTU_0089	Bacteria;Firmicutes;Clostridia;Clostridiales	0.000494548	0.001956385
OTU_0091	Bacteria;Firmicutes;Clostridia;Clostridiales;Ruminococcaceae;Oscillibacter	0.001093813	0.001158043
OTU_0093	Bacteria;Firmicutes;Clostridia;Clostridiales;Ruminococcaceae	0.000687501	0.00111527
OTU_0095	Bacteria;Firmicutes;Clostridia;Clostridiales	0.000229869	0.000750277
OTU_0096	Bacteria;Bacteroidetes;Bacteroidia;Bacteroidales;Prevotellaceae;Prevotella	0.003107771	0
OTU_0097	Bacteria;Firmicutes;Clostridia;Clostridiales;Lachnospiraceae;Blautia	0.000960859	0.001505046
OTU_0098	Bacteria;Firmicutes;Erysipelotrichia;Erysipelotrichales;Erysipelotrichaceae;Catenibacterium	0.000147155	0.00020062
OTU_0099	Bacteria;Firmicutes;Clostridia;Clostridiales	0.000186495	0.000403706
OTU_0100	Bacteria;Firmicutes;Clostridia;Clostridiales;Lachnospiraceae;Anaerostipes	0.000860492	0.002830499
OTU_0101	Bacteria;Firmicutes;Clostridia;Clostridiales;Clostridiaceae 1;Clostridium sensu stricto	0.000828706	0.000627366
OTU_0102	Bacteria;Firmicutes;Clostridia;Clostridiales;Ruminococcaceae	0.000185078	0.0009979
OTU_0103	Bacteria;Firmicutes;Clostridia;Clostridiales;Ruminococcaceae;Ruminococcus	0.001084603	0.001466538
OTU_0104	Bacteria;Firmicutes;Clostridia;Clostridiales;Ruminococcaceae	0.00156817	0.001448045
OTU_0107	Bacteria;Firmicutes;Bacilli;Lactobacillales;Streptococcaceae;Streptococcus	0.000984957	0.000215133
OTU_0109	Bacteria;Bacteroidetes;Bacteroidia;Bacteroidales;Porphyromonadaceae;Barnesiella	0.00204457	0.006496573
OTU_0117	Bacteria;Firmicutes;Clostridia;Clostridiales;Lachnospiraceae;Clostridium XlVb	0.000652915	0.001050612

OTU_0119	Bacteria;Firmicutes;Clostridia;Clostridiales;Peptostreptococcaceae;Clostridium XI	0.000620068	0.000443913
OTU_0120	Bacteria;Firmicutes;Erysipelotrichia;Erysipelotrichales;Erysipelotrichaceae;Clostridium XVIII	0.000480702	0.000401672
OTU_0121	Bacteria;Firmicutes;Clostridia;Clostridiales;Lachnospiraceae;Clostridium XlVb	0.001620831	0.00163645
OTU_0123	Bacteria;Firmicutes;Clostridia;Clostridiales;Lachnospiraceae;Clostridium XlVa	0.000489232	0.000600651
OTU_0124	Bacteria;Bacteroidetes;Bacteroidia;Bacteroidales;Rikenellaceae;Alistipes	0.000342957	0.000866309
OTU_0126	Bacteria;Firmicutes;Clostridia;Clostridiales;Ruminococcaceae;Clostridium IV	0.000413838	0.000395948
OTU_0127	Bacteria;Firmicutes;Clostridia;Clostridiales;Lachnospiraceae;Lachnospiracea_incertae_sedis	0.000405857	0.0017821
OTU_0128	Bacteria;Firmicutes;Clostridia;Clostridiales;Ruminococcaceae;Butyricicoccus	0.001313176	0.001850121
OTU_0129	Bacteria;Firmicutes;Clostridia;Clostridiales;Lachnospiraceae;Lachnospiracea_incertae_sedis	0.000468472	0.000729686
OTU_0130	Bacteria;Firmicutes;Clostridia;Clostridiales;Ruminococcaceae;Clostridium IV	0.00039721	0.00065095
OTU_0131	Bacteria;Firmicutes;Clostridia	0.00014745	0.00210688
OTU_0132	Bacteria;Firmicutes;Clostridia;Clostridiales;Lachnospiraceae;Clostridium XlVb	0.000886342	0.000124186
OTU_0134	Bacteria;Firmicutes;Clostridia;Clostridiales;Lachnospiraceae;Lachnospiracea_incertae_sedis	0.001334036	0.000617181
OTU_0136	Bacteria;Firmicutes;Bacilli;Lactobacillales;Lactobacillaceae;Lactobacillus	0.000816058	2.03933E-06
OTU_0137	Bacteria;Firmicutes;Clostridia;Clostridiales;Ruminococcaceae;Butyricicoccus	0.000410771	0.000622225
OTU_0139	Bacteria;Bacteroidetes;Bacteroidia;Bacteroidales;Porphyromonadaceae;Butyricimonas	0.000743743	0.001408892
OTU_0141	Bacteria;Firmicutes;Clostridia;Clostridiales	0.000205371	9.0918E-05
OTU_0142	Bacteria;Firmicutes;Clostridia;Clostridiales;Lachnospiraceae	0.001275498	0.000410825
OTU_0144	Bacteria;Bacteroidetes;Bacteroidia;Bacteroidales;Prevotellaceae;Prevotella	0.002077603	0.001356363
OTU_0147	Bacteria;Proteobacteria;Gammaproteobacteria;Enterobacteriales;Enterobacteriaceae;Citrobacter	0.003838204	0.000473619
OTU_0148	Bacteria;Firmicutes	0.000609632	0.000993952
OTU_0150	Bacteria;Proteobacteria;Alphaproteobacteria;Rhizobiales;Hyphomicrobiaceae;Gemmiger	0.007177933	0.012104397
OTU_0151	Bacteria;Firmicutes;Clostridia;Clostridiales;Ruminococcaceae;Flavonifractor	0.000469917	0.000323884
OTU_0153	Bacteria;Bacteroidetes;Bacteroidia;Bacteroidales;Rikenellaceae;Alistipes	0.000109749	0.000279992
OTU_0161	Bacteria;Proteobacteria;Deltaproteobacteria;Desulfovibrionales;Desulfovibrionaceae	0.000353525	0.00052435
OTU_0162	Bacteria;Bacteroidetes;Bacteroidia;Bacteroidales;Rikenellaceae;Alistipes	0.000154895	0.000229383
OTU_0170	Bacteria;TM7;TM7_genera_incertae_sedis;TM7_genera_incertae_sedis;TM7_genera_incertae_sedis;TM7_genera_incertae_sedis	8.38191E-05	7.49507E-05
OTU_0171	Bacteria;Firmicutes;Clostridia;Clostridiales;Lachnospiraceae;Lachnospiracea_incertae_sedis	0.000267243	0.000824103
OTU_0172	Bacteria;Bacteroidetes;Bacteroidia;Bacteroidales;Porphyromonadaceae;Parabacteroides	0.000210812	0.000677201
OTU_0175	Bacteria;Proteobacteria;Betaproteobacteria;Burkholderiales;Sutterellaceae;Sutterella	0.000944206	0.001370065
OTU_0176	Bacteria;Bacteroidetes;Bacteroidia;Bacteroidales;Porphyromonadaceae;Butyricimonas	0.00060466	0.00030082
OTU_0177	Bacteria;Firmicutes;Clostridia;Clostridiales;Ruminococcaceae;Ruminococcus	0.000136378	0.000552013
OTU_0179	Bacteria;Bacteroidetes;Bacteroidia;Bacteroidales;Bacteroidaceae;Bacteroides	0.000419942	0.000526751
OTU_0184	Bacteria;Firmicutes;Erysipelotrichia;Erysipelotrichales;Erysipelotrichaceae;Erysipelotrichaceae_incertae_sedis	0.000362065	0.000261396
OTU_0185	Bacteria;Firmicutes;Clostridia;Clostridiales	0.000359057	0.000610076
OTU_0186	Bacteria;Firmicutes;Clostridia;Clostridiales;Ruminococcaceae	3.94102E-05	0.000350092
OTU_0193	Bacteria;Firmicutes;Clostridia;Clostridiales;Ruminococcaceae;Clostridium IV	0.000160981	0.000268558
OTU_0197	Bacteria;Bacteroidetes;Bacteroidia;Bacteroidales;Rikenellaceae;Alistipes	9.7798E-05	0.000378928
OTU_0200	Bacteria;Firmicutes;Clostridia;Clostridiales;Lachnospiraceae;Lachnospiracea_incertae_sedis	0.00044979	0.00047795
OTU_0201	Bacteria;Firmicutes;Clostridia;Clostridiales;Ruminococcaceae;Flavonifractor	0.000197331	0.00016849
OTU_0203	Bacteria;Firmicutes;Erysipelotrichia;Erysipelotrichales;Erysipelotrichaceae;Turicibacter	0.000292458	0.000104745
OTU_0207	Bacteria;Firmicutes;Clostridia;Clostridiales;Lachnospiraceae;Coprococcus	0.000143191	0.000254591
OTU_0210	Bacteria;Firmicutes;Clostridia;Clostridiales;Ruminococcaceae	0.000100186	0.000219997
OTU_0211	Bacteria;Firmicutes;Clostridia	6.22741E-05	0.000354087
OTU_0218	Bacteria;Firmicutes;Erysipelotrichia;Erysipelotrichales;Erysipelotrichaceae;Holdemania	0.000265365	0.000297169
OTU_0220	Bacteria;Firmicutes;Clostridia;Clostridiales;Lachnospiraceae	4.40539E-05	6.33401E-05
OTU_0231	Bacteria;Firmicutes;Clostridia;Clostridiales;Ruminococcaceae	2.70054E-05	3.27796E-05
OTU_0233	Bacteria;Firmicutes;Clostridia;Clostridiales;Ruminococcaceae;Clostridium IV	4.36486E-05	4.87121E-05
OTU_0234	Bacteria;Bacteroidetes;Bacteroidia;Bacteroidales;Rikenellaceae;Alistipes	7.42064E-05	0.000253425
OTU_0235	Bacteria;Firmicutes;Clostridia;Clostridiales;Ruminococcaceae;Clostridium IV	4.01227E-05	0.000343461

OTU_0237	Bacteria;Firmicutes;Erysipelotrichia;Erysipelotrichales;Erysipelotrichaceae;Coprobacillus	4.49761E-05	0.000134989
OTU_0244	Bacteria;Actinobacteria;Actinobacteria;Coriobacteriales;Coriobacteriaceae;Eggerthella	0.000147328	0.000367001
OTU_0248	Bacteria;Bacteroidetes;Bacteroidia;Bacteroidales;Bacteroidaceae;Bacteroides	0.000375075	0.000250029
OTU_0254	Bacteria;Firmicutes;Bacilli;Lactobacillales;Carnobacteriaceae;Granulicatella	4.53185E-05	2.58279E-05
OTU_0256	Bacteria;Bacteroidetes;Bacteroidia;Bacteroidales;Bacteroidaceae;Bacteroides	0.001561458	0.002704658
OTU_0260	Bacteria;Bacteroidetes;Bacteroidia;Bacteroidales;Prevotellaceae;Paraprevotella	0.002294789	0.002475462
OTU_0261	Bacteria;Lentisphaerae;Lentisphaeria;Victivallales;Victivallaceae;Victivallis	8.35309E-05	0.000187105
OTU_0269	Bacteria;Firmicutes;Clostridia;Clostridiales;Ruminococcaceae	7.74008E-05	0.000259131
OTU_0270	Bacteria;Bacteroidetes;Bacteroidia;Bacteroidales;Bacteroidaceae;Bacteroides	0.003980433	0.00310942
OTU_0273	Bacteria;Firmicutes;Erysipelotrichia;Erysipelotrichales;Erysipelotrichaceae;Solobacterium	2.63095E-05	1.12521E-05
OTU_0277	Bacteria;Firmicutes;Clostridia;Clostridiales	4.49637E-05	0.00027841
OTU_0278	Bacteria;Firmicutes;Negativicutes;Selenomonadales;Veillonellaceae;Veillonella	0.001038391	0.000700227
OTU_0282	Bacteria;Firmicutes;Clostridia;Clostridiales;Lachnospiraceae	0.000176206	0.000172341
OTU_0285	Bacteria;TM7;TM7_genera_incertae_sedis;TM7_genera_incertae_sedis;TM7_genera_incertae_sedis;TM7_genera_incertae_sedis	5.66423E-05	1.36125E-05
OTU_0286	Bacteria;Bacteroidetes;Bacteroidia;Bacteroidales;Porphyromonadaceae;Butyricimonas	0.000375986	0.000615022
OTU_0287	Bacteria;Firmicutes;Clostridia;Clostridiales	3.20324E-05	0.000266146
OTU_0290	Bacteria;Firmicutes;Clostridia;Clostridiales;Ruminococcaceae	3.62951E-05	0.000148665
OTU_0291	Bacteria;Firmicutes;Clostridia;Clostridiales	1.80692E-05	0.000100509
OTU_0292	Bacteria;Firmicutes;Clostridia;Clostridiales;Ruminococcaceae;Pseudoflavonifractor	0.00018568	0.000164415
OTU_0298	Bacteria;Firmicutes;Clostridia;Clostridiales;Ruminococcaceae;Ruminococcus	0.00012265	2.30902E-05
OTU_0299	Bacteria;Firmicutes;Clostridia;Clostridiales;Lachnospiraceae	2.24654E-05	0.000101445
OTU_0303	Bacteria;Bacteroidetes;Bacteroidia;Bacteroidales;Porphyromonadaceae;Butyricimonas	0.000401916	0.000578897
OTU_0309	Bacteria;Firmicutes;Clostridia;Clostridiales;Lachnospiraceae;Clostridium XlVb	0.000142907	0.000158521
OTU_0310	Bacteria;Firmicutes;Clostridia;Clostridiales;Ruminococcaceae	3.63084E-05	7.62066E-05
OTU_0311	Bacteria;Firmicutes;Clostridia;Clostridiales;Eubacteriaceae;Eubacterium	3.69899E-05	5.60044E-05
OTU_0312	Bacteria;Lentisphaerae;Lentisphaeria;Victivallales;Victivallaceae;Victivallis	7.12864E-05	0.000120722
OTU_0315	Bacteria;Firmicutes;Clostridia;Clostridiales	4.54842E-05	8.00916E-05
OTU_0316	Bacteria;Firmicutes;Clostridia;Clostridiales;Ruminococcaceae;Anaerotruncus	4.28976E-05	5.06176E-05
OTU_0317	Bacteria;Actinobacteria;Actinobacteria;Actinomycetales;Actinomycetaceae;Actinomyces	3.53962E-05	3.29684E-05
OTU_0318	Bacteria;Firmicutes;Erysipelotrichia;Erysipelotrichales;Erysipelotrichaceae;Clostridium XVIII	5.47787E-05	2.59203E-05
OTU_0327	Bacteria;Firmicutes;Clostridia;Clostridiales;Ruminococcaceae	4.34172E-05	5.7557E-05
OTU_0334	Bacteria;Firmicutes;Negativicutes;Selenomonadales;Veillonellaceae;Allisonella	0.000158774	0.000267398
OTU_0336	Bacteria;Firmicutes;Erysipelotrichia;Erysipelotrichales;Erysipelotrichaceae;Erysipelotrichaceae_incertae_sedis	0.000111524	3.29548E-05
OTU_0337	Bacteria;Firmicutes;Clostridia;Clostridiales	0.00074922	0.000431201
OTU_0342	Bacteria;Bacteroidetes;Bacteroidia;Bacteroidales;Bacteroidaceae;Bacteroides	0.000724608	0.000972954
OTU_0344	Bacteria;Firmicutes;Clostridia;Clostridiales;Clostridiales_Incertae Sedis XIII	6.99027E-05	0.000110198
OTU_0345	Bacteria;Bacteroidetes;Bacteroidia;Bacteroidales;Prevotellaceae	0.000108615	8.07196E-06
OTU_0347	Bacteria;Firmicutes;Clostridia;Clostridiales;Ruminococcaceae;Anaerotruncus	0.00015554	0.000197614
OTU_0351	Bacteria;Firmicutes;Clostridia;Clostridiales;Ruminococcaceae	2.01837E-05	1.6909E-05
OTU_0353	Bacteria;Firmicutes;Clostridia;Clostridiales	3.94565E-05	0.000122189
OTU_0356	Bacteria;Firmicutes;Clostridia;Clostridiales;Lachnospiraceae;Clostridium XlVa	0.000102874	0.000123501
OTU_0372	Bacteria;Firmicutes;Negativicutes;Selenomonadales;Acidaminococcaceae;Acidaminococcus	0.000191223	3.78773E-05
OTU_0373	Bacteria;Fusobacteria;Fusobacteria;Fusobacteriales;Fusobacteriaceae;Fusobacterium	7.72604E-05	0.000241862
OTU_0377	Bacteria;Firmicutes;Clostridia;Clostridiales;Lachnospiraceae;Lachnospiracea_incertae_sedis	4.74586E-05	6.73742E-05
OTU_0379	Bacteria;Firmicutes;Clostridia;Clostridiales;Peptococcaceae 1;Peptococcus	4.51273E-05	4.38402E-05
OTU_0383	Bacteria;Firmicutes;Clostridia;Clostridiales;Ruminococcaceae;Clostridium IV	1.85017E-05	6.82411E-05
OTU_0390	Bacteria;Actinobacteria;Actinobacteria;Coriobacteriales;Coriobacteriaceae	4.91995E-05	0.000167734
OTU_0392	Bacteria;Firmicutes;Clostridia;Clostridiales;Lachnospiraceae;Pseudobutyrivibrio	0.000231688	0.000321674
OTU_0393	Bacteria;Bacteroidetes;Bacteroidia;Bacteroidales;Porphyromonadaceae;Butyricimonas	7.67744E-05	0.000304964
OTU_0395	Bacteria;Firmicutes;Clostridia;Clostridiales;Lachnospiraceae	0.000919095	0.001002083

OTU_0396	Bacteria;Firmicutes;Clostridia;Clostridiales	3.47191E-05	4.85892E-05
OTU_0397	Bacteria;Firmicutes;Clostridia;Clostridiales;Ruminococcaceae	3.8694E-05	8.56839E-05
OTU_0402	Bacteria;Firmicutes;Clostridia;Clostridiales;Lachnospiraceae;Clostridium XlVb	4.8674E-05	2.48535E-05
OTU_0403	Bacteria;Firmicutes;Clostridia;Clostridiales;Lachnospiraceae;Lachnospiracea_incertae_sedis	0.000178499	0.00023962
OTU_0409	Bacteria;Bacteroidetes;Bacteroidia;Bacteroidales;Bacteroidaceae;Bacteroides	0.014660944	0.018236007
OTU_0412	Bacteria;Proteobacteria;Betaproteobacteria;Burkholderiales;Oxalobacteraceae	4.21359E-05	6.54476E-05
OTU_0413	Bacteria;Bacteroidetes;Bacteroidia;Bacteroidales;Prevotellaceae;Prevotella	0.038219873	0.034450252
OTU_0414	Bacteria;Bacteroidetes;Bacteroidia;Bacteroidales;Bacteroidaceae;Bacteroides	0.006152412	0.004175854
OTU_0425	Bacteria;Proteobacteria;Gammaproteobacteria;Pasteurellales;Pasteurellaceae;Haemophilus	6.14715E-05	1.53648E-05
OTU_0436	Bacteria;Firmicutes;Clostridia;Clostridiales;Lachnospiraceae;Lachnospiracea_incertae_sedis	4.45142E-05	5.5729E-05
OTU_0437	Bacteria;Firmicutes;Negativicutes;Selenomonadales;Veillonellaceae;Dialister	2.51973E-05	1.26941E-05
OTU_0451	Bacteria;Actinobacteria;Actinobacteria;Actinomycetales;Micrococcaceae;Rothia	4.34488E-05	1.83555E-05
OTU_0458	Bacteria;Bacteroidetes;Bacteroidia;Bacteroidales;Prevotellaceae;Prevotella	0.002522115	0.002387258
OTU_0464	Bacteria;Firmicutes;Clostridia;Clostridiales;Lachnospiraceae;Clostridium XlVa	0.003119746	0.003101103
OTU_0467	Bacteria;Firmicutes	0.000134192	0.000448166
OTU_0475	Bacteria;Firmicutes;Clostridia;Clostridiales;Lachnospiraceae	0.000772452	0.000545303
OTU_0476	Bacteria;Firmicutes;Clostridia;Clostridiales;Lachnospiraceae;Blautia	0.001179053	0.000793033
OTU_0479	Bacteria;Bacteroidetes;Bacteroidia;Bacteroidales;Bacteroidaceae;Bacteroides	2.05657E-05	9.21069E-05
OTU_0480	Bacteria;Firmicutes;Clostridia;Clostridiales;Ruminococcaceae;Clostridium IV	2.04765E-05	4.22935E-05
OTU_0486	Bacteria;Firmicutes;Clostridia;Clostridiales;Lachnospiraceae;Clostridium XlVb	0.000101515	0.000123427
OTU_0487	Bacteria;Bacteroidetes;Bacteroidia;Bacteroidales;Porphyromonadaceae;Parabacteroides	0.00018361	0.000440625
OTU_0495	Bacteria;Firmicutes;Clostridia;Clostridiales;Ruminococcaceae;Flavonifractor	3.10708E-05	0.000132715
OTU_0506	Bacteria;Firmicutes;Clostridia;Clostridiales;Ruminococcaceae;Flavonifractor	5.1065E-05	0.000126385
OTU_0511	Bacteria;Bacteroidetes;Bacteroidia;Bacteroidales;Bacteroidaceae;Bacteroides	0.000137157	9.91226E-05
OTU_0520	Bacteria;Bacteroidetes;Bacteroidia;Bacteroidales;Prevotellaceae;Prevotella	0.001584679	0.002100474
OTU_0526	Bacteria;Firmicutes;Clostridia;Clostridiales;Ruminococcaceae;Clostridium IV	1.40116E-05	1.81228E-05
OTU_0540	Bacteria;Firmicutes;Clostridia;Clostridiales;Ruminococcaceae	1.29584E-05	8.20186E-06
OTU_0552	Bacteria;Bacteroidetes;Bacteroidia;Bacteroidales;Bacteroidaceae;Bacteroides	0.001730264	0.001326457
OTU_0564	Bacteria;Firmicutes;Clostridia;Clostridiales;Ruminococcaceae;Clostridium IV	1.10269E-05	3.18581E-05
OTU_0575	Bacteria;Bacteroidetes;Bacteroidia;Bacteroidales;Bacteroidaceae;Bacteroides	0.010698634	0.005256309
OTU_0578	Bacteria;Firmicutes;Clostridia;Clostridiales;Ruminococcaceae;Anaerofilum	1.04104E-05	3.23652E-06
OTU_0585	Bacteria;Bacteroidetes;Bacteroidia;Bacteroidales;Bacteroidaceae;Bacteroides	0.000180223	8.63756E-05
OTU_0611	Bacteria;Bacteroidetes;Bacteroidia;Bacteroidales;Bacteroidaceae;Bacteroides	0.000176968	4.86822E-05
OTU_0616	Bacteria;Firmicutes;Clostridia;Clostridiales;Lachnospiraceae;Lachnospiracea_incertae_sedis	0.000147293	0.000168373
OTU_0624	Bacteria;Bacteroidetes;Bacteroidia;Bacteroidales;Rikenellaceae;Alistipes	0.001260765	0.000304993
OTU_0653	Bacteria;Bacteroidetes;Bacteroidia;Bacteroidales;Prevotellaceae;Prevotella	0.000101883	7.03649E-05
OTU_0690	Bacteria;Bacteroidetes;Bacteroidia;Bacteroidales;Bacteroidaceae;Bacteroides	4.50307E-05	2.26296E-05
OTU_0702	Bacteria;Bacteroidetes;Bacteroidia;Bacteroidales;Prevotellaceae;Prevotella	6.65492E-05	5.4547E-05
OTU_0721	Bacteria;Firmicutes;Clostridia;Clostridiales;Lachnospiraceae;Clostridium XlVa	5.48773E-06	1.14752E-05
OTU_0742	Bacteria;Firmicutes;Clostridia;Clostridiales;Lachnospiraceae;Lachnospiracea_incertae_sedis	0.000389505	0.000607942
OTU_0744	Bacteria;Firmicutes;Clostridia;Clostridiales;Ruminococcaceae;Sporobacter	0.000403781	0.00061856
OTU_0748	Bacteria;Firmicutes;Negativicutes;Selenomonadales;Veillonellaceae;Megamonas	0.00399135	0.000637904
OTU_0749	Bacteria;Actinobacteria;Actinobacteria;Bifidobacteriales;Bifidobacteriaceae;Bifidobacterium	0.000130528	0.00026995
OTU_0763	Bacteria;Firmicutes;Clostridia;Clostridiales;Ruminococcaceae;Acetanaerobacterium	3.70426E-05	4.56164E-05
OTU_0776	Bacteria;Firmicutes;Clostridia;Clostridiales;Lachnospiraceae;Lachnospiracea_incertae_sedis	0.005307424	0.003530622
OTU_0779	Bacteria;Firmicutes;Clostridia;Clostridiales;Lachnospiraceae;Clostridium XlVa	0.002491856	0.002534454
OTU_0790	Bacteria;Firmicutes;Clostridia;Clostridiales;Lachnospiraceae;Lachnospiracea_incertae_sedis	0.000389297	0.000605418
OTU_0794	Bacteria;Firmicutes;Clostridia;Clostridiales;Lachnospiraceae;Clostridium XlVa	0.004624048	0.002956972
OTU_0796	Bacteria;Bacteroidetes;Bacteroidia;Bacteroidales;Bacteroidaceae;Bacteroides	6.97024E-05	2.92639E-05
OTU_0799	Bacteria;Firmicutes;Erysipelotrichia;Erysipelotrichales;Erysipelotrichaceae;Erysipelotrichaceae_incertae_sedis	4.46111E-05	5.11041E-05
OTU_0802	Bacteria;Firmicutes;Clostridia;Clostridiales;Ruminococcaceae;Oscillibacter	0.003825647	0.007903506

OTU_0812	Bacteria;Bacteroidetes;Bacteroidia;Bacteroidales;Prevotellaceae;Prevotella	9.46056E-05	0.000167333
OTU_0825	Bacteria;Firmicutes;Clostridia;Clostridiales;Lachnospiraceae;Lachnospiracea_incertae_sedis	0.000684637	0.001348947
OTU_0826	Bacteria;Firmicutes;Clostridia;Clostridiales;Lachnospiraceae;Lachnospiracea_incertae_sedis	0.000224313	0.000271216
OTU_0830	Bacteria;Firmicutes;Clostridia;Clostridiales	0.000261957	0.000420054
OTU_0834	Bacteria;Firmicutes;Clostridia;Clostridiales;Ruminococcaceae;Pseudoflavonifractor	3.85893E-05	6.07469E-05
OTU_0835	Bacteria;Bacteroidetes;Bacteroidia;Bacteroidales;Bacteroidaceae;Bacteroides	0.000215767	7.63917E-05
OTU_0849	Bacteria;Firmicutes;Clostridia;Clostridiales;Lachnospiraceae;Lachnospiracea_incertae_sedis	0.000701332	0.000835631
OTU_0857	Bacteria;Firmicutes;Clostridia;Clostridiales	4.76921E-05	6.52689E-05
OTU_0864	Bacteria;Firmicutes;Clostridia;Clostridiales;Lachnospiraceae;Lachnospiracea_incertae_sedis	0.00012877	0.000195951
OTU_0872	Bacteria;Firmicutes;Clostridia;Clostridiales;Ruminococcaceae;Flavonifractor	0.000676265	0.000425352
OTU_0884	Bacteria;Firmicutes;Clostridia;Clostridiales;Lachnospiraceae;Clostridium XlVa	0.000897674	0.000995646
OTU_0899	Bacteria;Firmicutes;Clostridia;Clostridiales;Ruminococcaceae;Oscillibacter	0.000517715	0.000422378
OTU_0927	Bacteria;Firmicutes;Clostridia;Clostridiales;Ruminococcaceae	0.000539372	0.000922043
OTU_0930	Bacteria;Firmicutes;Clostridia;Clostridiales;Lachnospiraceae;Clostridium XlVa	0.000274891	0.000247258
OTU_0932	Bacteria;Bacteroidetes;Bacteroidia;Bacteroidales;Prevotellaceae;Prevotella	0.039347757	0.061259565
OTU_0935	Bacteria;Bacteroidetes;Bacteroidia;Bacteroidales;Porphyromonadaceae;Parabacteroides	0.001319655	0.000782423
OTU_0942	Bacteria;Firmicutes;Clostridia;Clostridiales;Ruminococcaceae;Oscillibacter	3.57185E-05	9.99109E-05
OTU_0946	Bacteria;Firmicutes;Clostridia;Clostridiales;Ruminococcaceae;Faecalibacterium	5.32548E-05	7.01849E-05
OTU_0947	Bacteria;Firmicutes;Clostridia;Clostridiales;Lachnospiraceae;Lachnospiracea_incertae_sedis	4.39095E-05	2.59485E-05
OTU_0960	Bacteria;Firmicutes;Clostridia;Clostridiales;Ruminococcaceae;Butyricicoccus	0.000135718	0.000215943
OTU_0968	Bacteria;Bacteroidetes;Bacteroidia;Bacteroidales;Prevotellaceae;Prevotella	0.022590311	0.017238388
OTU_0976	Bacteria;Bacteroidetes;Bacteroidia;Bacteroidales;Prevotellaceae;Paraprevotella	1.97871E-05	1.2416E-05
OTU_1010	Bacteria;Bacteroidetes;Bacteroidia;Bacteroidales;Porphyromonadaceae;Parabacteroides	1.01633E-05	1.06135E-06
OTU_1016	Bacteria;Firmicutes;Clostridia;Clostridiales;Lachnospiraceae;Lachnospiracea_incertae_sedis	0.000188308	0.000224018
OTU_1017	Bacteria;Bacteroidetes;Bacteroidia;Bacteroidales;Bacteroidaceae;Bacteroides	5.71817E-05	7.0587E-05
OTU_1025	Bacteria;Firmicutes;Clostridia;Clostridiales;Lachnospiraceae;Lachnospiracea_incertae_sedis	0.000411049	0.000122776
OTU_1027	Bacteria;Firmicutes;Clostridia;Clostridiales;Lachnospiraceae;Lachnospiracea_incertae_sedis	0.000147356	6.09187E-05
OTU_1032	Bacteria;Proteobacteria;Gammaproteobacteria;Enterobacteriales;Enterobacteriaceae;Klebsiella	0.000549757	2.13599E-05
OTU_1048	Bacteria;Firmicutes;Clostridia;Clostridiales;Lachnospiraceae;Roseburia	0.007145819	0.004187411
OTU_1057	Bacteria;Firmicutes;Clostridia;Clostridiales;Lachnospiraceae;Lachnospiracea_incertae_sedis	0.000440813	0.000131129
OTU_1082	Bacteria;Firmicutes;Clostridia;Clostridiales;Lachnospiraceae;Pseudobutyrivibrio	2.27986E-05	1.46491E-05
OTU_1089	Bacteria;Firmicutes;Clostridia;Clostridiales;Ruminococcaceae	1.76418E-05	4.24887E-05
OTU_1090	Bacteria;Bacteroidetes;Bacteroidia;Bacteroidales;Prevotellaceae;Prevotella	0.001080632	0.001836348
OTU_1091	Bacteria;Firmicutes;Clostridia;Clostridiales;Ruminococcaceae	2.33592E-05	0.00013378
OTU_1097	Bacteria;Bacteroidetes;Bacteroidia;Bacteroidales;Bacteroidaceae;Bacteroides	0.003150894	0.002923429
OTU_1099	Bacteria;Firmicutes;Clostridia;Clostridiales;Lachnospiraceae;Lachnospiracea_incertae_sedis	0.000723227	0.000868
OTU_1109	Bacteria;Bacteroidetes;Bacteroidia;Bacteroidales;Rikenellaceae;Alistipes	3.10433E-05	5.50726E-05
OTU_1113	Bacteria;Firmicutes;Clostridia;Clostridiales;Lachnospiraceae;Blautia	0.00014472	0.000220476
OTU_1116	Bacteria;Bacteroidetes;Bacteroidia;Bacteroidales;Bacteroidaceae;Bacteroides	2.51233E-05	1.7583E-05
OTU_1119	Bacteria;Firmicutes;Clostridia;Clostridiales;Lachnospiraceae;Lachnospiracea_incertae_sedis	7.74456E-05	3.1251E-05
OTU_1125	Bacteria;Firmicutes;Clostridia;Clostridiales;Lachnospiraceae;Roseburia	3.99123E-05	2.72711E-05
OTU_1135	Bacteria;Firmicutes;Clostridia;Clostridiales;Lachnospiraceae;Roseburia	0.000142607	3.98181E-05
OTU_1149	Bacteria;Firmicutes;Clostridia;Clostridiales;Lachnospiraceae;Lachnospiracea_incertae_sedis	0.000165345	9.60131E-05
OTU_1150	Bacteria;Firmicutes;Clostridia;Clostridiales;Lachnospiraceae;Clostridium XlVa	0.000108616	0.000207701
OTU_1154	Bacteria;Firmicutes;Clostridia;Clostridiales;Clostridiaceae 1;Clostridium sensu stricto	8.97314E-05	5.63072E-05
OTU_1158	Bacteria;Firmicutes;Clostridia;Clostridiales;Lachnospiraceae;Clostridium XlVa	0.000294841	0.000492653
OTU_1184	Bacteria;Fusobacteria;Fusobacteria;Fusobacteriales;Fusobacteriaceae;Fusobacterium	0.001884742	0.002032545
OTU_1191	Bacteria;Firmicutes;Clostridia;Clostridiales;Ruminococcaceae;Faecalibacterium	6.14608E-05	4.41283E-05
OTU_1200	Bacteria;Bacteroidetes;Bacteroidia;Bacteroidales;Bacteroidaceae;Bacteroides	0.000518704	5.75627E-05
OTU_1207	Bacteria;Firmicutes;Clostridia;Clostridiales;Ruminococcaceae;Oscillibacter	5.44493E-05	3.70578E-05
OTU_1217	Bacteria;Bacteroidetes;Bacteroidia;Bacteroidales;Prevotellaceae;Prevotella	0.025620768	0.015710267
OTU_1218	Bacteria;Firmicutes;Clostridia;Clostridiales;Ruminococcaceae	0.001694895	0.004546032

OTU_1243	Bacteria;Firmicutes;Clostridia;Clostridiales;Lachnospiraceae;Dorea	0.000317388	0.000774572
OTU_1247	Bacteria;Firmicutes;Clostridia;Clostridiales;Lachnospiraceae	0.000377971	0.000181242
OTU_1260	Bacteria;Firmicutes;Clostridia;Clostridiales;Ruminococcaceae;Oscillibacter	0.000190629	0.000219764
OTU_1275	Bacteria;Firmicutes;Clostridia;Clostridiales;Lachnospiraceae;Coprococcus	0.000129622	5.12423E-05
OTU_1281	Bacteria;Firmicutes;Clostridia;Clostridiales;Ruminococcaceae;Oscillibacter	2.39088E-05	7.22902E-05
OTU_1294	Bacteria;Firmicutes;Clostridia;Clostridiales;Ruminococcaceae;Faecalibacterium	0.000389067	0.000577654
OTU_1295	Bacteria;Bacteroidetes;Bacteroidia;Bacteroidales;Bacteroidaceae;Bacteroides	0.000207647	7.63132E-05
OTU_1296	Bacteria;Firmicutes;Negativicutes;Selenomonadales;Veillonellaceae;Dialister	0.001521759	0.000574109

supplementary Table S5 Clinical phenotype information of healthy individuals

ID	Gender	Age (year)	Height (cm)	Weight (kg)	BMI (kg/m2)	AFP in serum (ng/mL)	ALT (U/L)	AST (U/L)	GTT (U/L)	TP (g/L)	ALB (g/L)	GLO (g/L)	TB (μmol/L)	DB (μmol/L)	IB (μmol/L)	CEA (ng/mL)	CA199 (U/mL)	CA125 (U/mL)
zfh201	female	58	160	60	23.44	3	20	21	20	73.6	45.8	27.8	8	3	5	2.3	30.1	24.4
zfh202	male	47	170.8	71.6	24.54	2.2	26	28	28	71.2	48.2	23	14	4	10	3.6	17.2	10.9
zfh203	female	48	159.8	55.9	21.89	0.9	10	14	9	75	46.6	28.4	12	5	7	1.4	27.5	11.6
zfh204	male	49	171.8	71.1	23.75	2.2	18	19	13	75	51	24	11	4	7	3.5	7.9	
zfh205	male	44	176	75	24.21	2.7	21	25	27	76	48	28	11	4	7	2.2	5.1	6.1
zfh206	male	42	171	61	20.86	3.1	18	17	12	83	52.9	30.1	11	4	7			
zfh207	male	44	165.9	70.4	25.58	1.8	29	21	17	76.5	51.4	25.1	14	5	9			
zfh208	male	52	164.4	51.3	18.98	1.5	23	22	25	75.5	52.8	22.7	15	5	10			
zfh209	male	55	168.9	52.7	18.47	2.7	13	21	47	70.8	43.9	26.9	9	4	5	4.2	21	8.7
zfh210	female	52	158	59	23.63	3.5	25	29	14	75.5	49.6	25.9	12	4	8	2.9	22.3	30.5
zfh211	male	53	177.3	79.6	25.32	3.2	30	29	15	71.2	50.4	20.8	17	5	12	2.8	50.3	6.8
zfh212	male	51	171.2	60.4	20.61	3	13	15	16	79.3	53.4	25.9	14	5	9	2.6	4.2	5.3
zfh213	male	51	169.6	74.2	25.8	2.1	40	29	28	75.1	48.7	26.4	14	4	10			
zfh214	male	59	177	78	24.9	4.2	18	25	23	73.5	48.7	24.8	22	7	15	2.5	19.4	4.4
zfh215	male	55	165.7	63.2	23.02	2.7	14	14	12	70.6	47.5	23.1	14	5	9	2.4	2	7.5
zfh216	male	47	168	68	24.09	3.4	30	24	18	78.3	50.2	28.1	15	5	10	1.3	8.1	8.9
zfh217	female	49	155.8	65.7	27.07	5.6	15	24	14	74.5	48.6	25.9	11	5	6			
zfh218	male	58	167.9	62.8	22.28	4.5	19	23	16	72.7	46.7	26	16	5	11	2.3	2	5.9
zfh219	female	52	155	60	24.97	1.6	26	29	68	73.4	47.9	25.5	7	3	4	1	7.8	9.1
zfh220	male	59	175	75	24.49	2.5	15	15	37	78.6	47.9	30.7	15	4	11	3	2	8.5
zfh221	male	61	172	69.5	23.49	1.7	19	25	37	69	45	24	16	5	11	2	6.9	5.1
zfh222	male	56	172	69	23.32	3.4	28	26	37	72.9	44.9	28	12	3	9	2.2	6.2	12.4
zfh223	male	42	180	75	23.15	2	21	23	8	78.5	50.3	28.2	14	4	10	1.8	3	12.1
zfh224	male	50	165	70	25.71	1.8	21	21	37	72.8	46.8	26	12	4	8			
zfh225	male	44	182	76.6	23.13	2.2	16	19	16	72.5	46.9	25.6	8	4	4	1.8	4.2	9.6
zfh226	male	57	168.2	68.8	24.32	3.4	25	23	51	72.5	47.2	25.3	12	4	8	3.6	2.8	8.7
zfh227	male	57	175	67	21.88	1.4	22	21	24	71	48.2	22.8	14	3	11	1.3	7.3	6.9
zfh228	male	59	162.4	55.4	21.01	4.4	28	20	14	74.7	48.7	26	10	2	8	1.7	18.3	7.3
zfh229	male	56	157	48.1	19.51	6	10	12	15	69.5	48.5	21	13	4	9	1.5	14.9	15.5

zfh230	male	48	173	70	23.39	3.3	38	31	19	80.9	52	28.9	16	5	11	2.1	8.1	13.3
zfh231	male	50	169.1	66.7	23.33	3.6	12	12	15	73.6	48.2	25.4	7	4	3	1	2.7	3.2
zfh232	male	53	165.8	54.8	19.93	2.8	14	17	23	83.9	51.9	32	26	10	16	1.7	2.5	8.2
zfh233	male	44	157.1	63.4	25.69	5.3	17	15	16	77.2	49.5	27.7	9	3	6	0.6	11.8	13.6
zfh234	male	53	175	79	25.8	3.1	23	21	40	76.8	50.5	26.3	8	3	5	2.1	17.4	4.7
zfh235	male	48	159.8	55.9	21.89	1.2	10	14	9	75	46.6	28.4	12	5	7	1.4	27.5	11.6
zfh236	male	45	169.6	72.7	25.27	2.2	16	19	15	73.3	50.6	22.7	29	11	18	1.6	2	10.7
zfh237	male	42	171	61	20.86	2.5	18	17	12	83	52.9	30.1	11	4	7			
zfh238	male	52	169	75	26.26	3.3	15	19	14	71.9	51	20.9	21	5	16	1.3	9.2	10.1
zfh239	male	50	159.2	55.7	21.98	6.6	12	18	7	70.8	47.5	23.3	6	3	3	1.1	9.1	22
zfh240	female	52	158	60	24.03	1.1	28	25	44	74.7	48.7	26	12	4	8	2.9	2	11.5
zfh241	male	47	169.3	69.5	24.25	1.6	28	19	27	66.2	46.9	19.3	7	3	4			
zfh242	male	45	183.9	84.4	24.96	2.6	36	23	32	76	50.3	25.7	22	8	14	1.8	6.1	
zfh243	male	46	175	76	24.82	9	12	15	19	69.5	49.2	20.3	16	5	11	0.9	7.1	5.8
zfh244	male	57	176.9	79.6	25.44	4.1	33	27	29	76.7	50.5	26.2	15	5	10	1.9	3.9	
zfh245	male	51	175	61	19.92	3.4	15	23	17	72.5	48.7	23.8	13	5	8	2.1	2	7.7
zfh246	male	43	163.3	52.8	19.8	2.3	13	18	11	75.4	49	26.4	16	5	11			
zfh247	male	59	172	70	23.66	2.6	23	25	31	71	43.5	27.5	14	6	8			
zfh248	male	49	164.8	55.2	20.32	3.8	15	17	13	73.7	47.2	26.5	11	3	8	1.1	19.6	9
zfh249	male	44	175.5	61.6	20	1.9	41	40	27	75.2	50.4	24.8	18	4	14	2.4	3.7	10.3
zfh250	female	43	158.5	51.7	20.58	1.6	24	27	14	75.2	46.9	28.3	10	4	6	1.8	36.5	11.9
zfh251	male	48	185.2	76.2	22.22	4.4	22	30	17	70.3	45	25.3	13	5	8	0.7	9	6.2
zfh252	male	44	164	60.9	22.64	1.5	11	17	7	77.6	44.3	33.3	8	3	5	1.2	11.1	11.5
zfh253	male	51	161.7	50.8	19.43	3.1	12	16	18	75.9	44.7	31.2	14	5	9	0.6	2.6	14.2
zfh254	male	52	163	60	22.58	1.7	18	22	13	77.3	45.5	31.8	5	2	3	0.9	2.2	73.2
zfh255	male	43	176	76	24.54	1.7	19	19	23	78.9	50.2	28.7	15	4	11	2	29.1	14.7
zfh256	male	52	180.3	79.3	24.39	2.7	16	24	13	74.3	46.6	27.7	15	5	10	0.7	10.3	12.6
zfh257	male	43	175.3	62.2	20.24	5.1	16	22	22	70.5	47	23.5	18	5	13	0.9	6.7	6.1
zfh258	female	47	146.2	60.2	28.16	1.6	15	15	18	73.9	49.1	24.8	12	3	9			
zfh259	male	53	164.6	61.3	22.63	5.8	11	17	7	73.1	46.6	26.5	8	2	6			
zfh260	male	48	173.8	71.9	23.8	12.8	27	25	21	71.9	48.3	23.6	11	3	8	1	4.9	12.8
zfh261	female	54	157.3	45.3	18.31	2.3	25	24	75	75.5	49.5	26	9	4	5			

zfh262	male	36	168.5	71.3	25.11	5	15	19	15	77.3	51	26.3	9	4	5			
zfh263	female	43	163.3	52.8	19.8	2.6	13	18	11	75.4	49	26.4	16	5	11			
zfh264	female	43	163.3	52.8	19.8	2.6	13	18	11	75.4	49	26.4	16	5	11			
zfh265	male	37	175.5	60.1	19.51	2.1	20	19	16	71.7	50.5	21.2	12	5	7			
zfh266	female	49	155.8	60.7	25.01	2.5	15	24	14	74.5	48.6	25.9	11	5	6			
zfh267	male	58	167.9	62.8	22.28	4.5	19	23	16	72.7	46.7	26	16	5	11	2.3	2	5.9
zfh268	male	58	169	69	24.16	3.8	24	28	19	70.5	42.6	27.9	12	4	8	1.3	12.8	8.9
zfh269	male	43	158.5	51.7	20.58	1.6	24	23	14	75.2	46.9	28.3	10	4	6	1.8	36.5	11.9
zfh270	male	49	158.6	47	18.68	1.8	24	24	16	76	52.8	23.2	11	4	7	1.3	3.9	10.1
zfh271	male	61	172	69.5	23.49	1.7	19	23	37	69	45	24	16	5	11	2	6.9	5.1
zfh272	male	44	182.6	77	23.09	2	33	23	21	71.5	46.5	25	14	5	9	1.1	6.1	8.1
zfh273	male	51	173	74.3	24.83	2.1	22	21	20	77.8	51.6	26.2	12	5	7	2.1	4.9	11.1
zfh274	male	54	160	65	25.39	1.6	18	21	22	69.6	44.8	24.8	16	6	10			
zfh275	male	57	168.2	68.8	24.32	3.4	25	23	51	72.5	47.2	25.3	12	4	8	3.6	2.8	8.7
zfh276	male	52	161	55	21.22	1.7	18	22	13	77.3	45.5	31.8	5	2	3	0.9	2.2	73.2
zfh277	male	53	172	63.4	21.43	3.9	13	18	14	69.9	45.8	24.1	11	4	7	1.9	6.4	
zfh278	male	40	175.2	64.7	21.08	2.2	23	27	25	84.8	55.2	29.6	30	10	20	1.5	24.1	6.3
zfh279	male	53	172	63.4	21.43	2.9	13	18	14	69.9	45.8	24.1	11	4	7	1.9	6.4	
zfh280	male	39	172	70	23.66	2.8	17	27	23	75.2	50.1	25.1	16	5	11	3.9	11.4	11.5
zfh281	female	48	159.8	55.9	21.89	1.2	10	14	9	75	46.6	28.4	12	5	7	1.4	27.5	11.6
zfh282	male	42	171	61	20.86	3.1	18	17	12	83	52.9	30.1	11	4	7			
zfh283	male	59	175.5	79.1	25.68	1.8	39	19	37	69	47.9	21.1	9	4	5			
zfh284	female	52	159	61	24.13	3.3	15	19	14	71.9	51	20.9	21	5	16	1.3	9.2	10.1
zfh285	female	50	159.2	55.7	21.98	6.6	12	18	7	70.8	47.5	23.3	6	3	3	1.1	9.1	22
zfh286	male	52	164.4	51.3	18.98	2	23	22	25	75.5	52.8	22.7	15	5	10			
zfh287	male	55	168.9	52.7	18.47	2.7	13	21	47	70.8	43.9	26.9	9	4	5	4.2	21	8.7
zfh288	male	36	176	75	24.21	2	21	28	12	78.9	50.9	28	8	4	4	1.6	6.7	6.9
zfh289	male	47	169.3	69.5	24.25	1.5	28	19	27	66.2	46.9	19.3	7	3	4			
zfh290	male	45	183.9	84.4	24.96	2.6	36	23	32	76	50.3	25.7	22	8	14	1.8	6.1	
zfh291	male	52	160	59	23.05	3.5	25	24	14	75.5	49.6	25.9	12	4	8	2.9	22.3	30.5
zfh292	male	46	156.2	50.2	20.58	1.9	11	14	12	74.5	49.9	24.6	10	3	7	5.3	24.8	13.2
zfh293	male	58	176.1	67.7	21.83	2.2	24	21	12	81	50.7	30.3	20	6	14	1.4	8.6	9.8

zfh294	male	41	168.3	60.6	21.39	1.4	14	24	18	76.8	52.5	24.3	41	15	26	1.5	4	7.3
zfh295	male	41	177	82.2	26.24	2.1	18	18	28	75	51.9	23.1	16	5	11	2.1	2	5.3
zfh296	male	38	160.7	61.9	23.97	1.9	17	21	18	74.2	48.4	25.8	10	4	6	0.9	17.8	7.3
zfh297	male	57	176.9	79.6	25.44	4.1	33	27	29	76.7	50.5	26.2	15	5	10	1.9	3.9	
zfh298	male	46	169	68	23.81	9	12	15	19	69.5	49.2	20.3	16	5	11	0.9	7.1	5.8
zfh299	female	58	158	61	24.44	3	20	24	20	73.6	45.8	27.8	8	3	5	2.3	30.1	24.4
zfh300	male	51	175	79	25.8	3.4	15	23	17	72.5	48.7	23.8	13	5	8	2.1	2	7.7

supplementary Table S6 Clinical characteristics summary of all enrolled individuals

Clinical and pathological index	Healthy Control (n=100)	HCC Group (n=113)	P value	HCC Group (n=113)			HCC Group (n=113)		
				Small (n=36)	Non-Small (n=77)	P value	Non-Cirrhosis (n=22)	Cirrhosis (n=91)	P value
Age (year)	49.57±6.09	51.26± 8.07	0.051	50.11±9.66	51.79±7.22	0.407	51.68±7.52	51.15±8.24	0.919
Gender			0.069			0.028			1.000
Female	16 (16%)	9 (8%)		6 (17%)	3 (4%)		1 (5%)	8 (9%)	
Male	84 (84%)	104 (92%)		30 (83%)	74 (96%)		21 (95%)	83 (91%)	
BMI	22.9±2.2	22.7±2.7	0.509	22.5±2.5	22.8±2.8	0.550	21.9±2.4	22.9±2.8	0.113
AFP (ng/mL)			<0.001			0.571			0.484
≤20	100 (100%)	49 (43%)		17 (47%)	32 (42%)		11 (50%)	38 (42%)	
>20	0 (0%)	64 (57%)		19 (53%)	45 (58%)		11 (50%)	53 (58%)	
Tumor size (cm)			–			<0.001			0.607
≤3	–	36 (32%)		36 (100%)	0 (0%)		6 (27%)	30 (33%)	
>3	–	77 (68%)		0 (0%)	77 (100%)		16 (73%)	61 (67%)	
Tumor differentiation			–			0.273			0.613
I-II	–	77 (68%)		22 (61%)	55 (71%)		14 (64%)	63 (69%)	
III-IV	–	36 (32%)		14 (39%)	22 (29%)		8 (36%)	28 (31%)	
Liver cirrhosis	0 (0%)	91 (81%)		30 (83%)	61 (79%)		0 (0%)	91 (100%)	
Portal hypertension						0.406			<0.001
Negative	–	47 (42%)		17 (47%)	30 (39%)		22 (100%)	25 (28%)	
Positive	–	66 (58%)		19 (53%)	47 (61%)		0 (0%)	66 (72%)	
Child-Pugh			–			0.095			1.000
A	–	106 (94%)		36 (100%)	70 (91%)		21 (95%)	85 (93%)	
B	–	7 (6%)		0 (0%)	7 (9%)		1 (5%)	6 (7%)	
ALT (5-40 U/L)	20.2±7.4	36.8±25.8	<0.001	28.5±17.5	38.5±21.4	0.005	31.8±23.3	36.2±20.1	0.067
AST (8-40 U/L)	21.4±4.8	40.5±26.5	<0.001	28.9±12.8	43.3±22.0	<0.001	37.4±24.3	39.0±19.7	0.185
GGT (11-50 U/L)	21.5±12.4	94.9±97.6	<0.001	54.8±57.6	113.7±106.7	<0.001	102.7±140.1	93.1±85.2	0.210
Total protein (64.0-83.0 g/L)	74.4±3.6	68.3±5.3	<0.001	68.5±5.4	68.2±5.3	0.615	69.7±5.3	68.0±5.2	0.242
Albumin (35.0-55.0 g/L)	48.6±2.5	39.5±4.5	<0.001	40.8±3.9	38.9±4.6	0.032	40.9±4.1	39.2±4.5	0.105
Globulin (20.0-35.0 g/L)	25.8±2.9	28.8±4.8	<0.001	27.7±3.6	29.3±5.1	0.159	28.7±5.4	28.8±4.6	0.632
Total bilirubin (μmol/L)	13.4±5.4	19.9±32.9	0.001	15.7±6.5	21.9±39.6	0.446	32.3±72.4	17.0±9.0	0.378
Direct bilirubin (μmol/L)	4.6±1.8	9.2±24.6	<0.001	6.2±2.8	10.6±29.7	0.599	18.4±55.0	7.0±4.7	0.484
Prothrombin time (12-14 s)	ND	12.4±1.6	–	12.5±1.5	12.3±1.6	0.484	11.9±1.5	12.5±1.6	0.009
Fasting plasma glucose (3.89-6.1mmol/l)	ND	4.8±0.8	–	4.7±0.5	4.9±0.9	0.418	4.9±1.1	4.8±0.7	0.459
Total cholesterol (3-5.72mmol/L)	ND	3.9±1.0	–	3.7±0.8	4.0±1.1	0.355	4.2±1.3	3.9±0.9	0.856
High-density lipoprotein (0.7-2.0mmol/L)	ND	1.1±0.3	–	1.0±0.3	1.1±0.3	0.165	1.0±0.3	1.1±0.3	0.217
Low-density lipoprotein (<3.12mmol/L)	ND	2.1±0.5	–	2.0±0.5	2.1±0.5	0.534	2.2±0.6	2.0±0.5	0.286
HBV infection	0 (0%)	113 (100%)	–	36 (100%)	77 (100%)	–	22 (100%)	91 (100%)	–
Dietary habit	Mixed diet	Mixed diet	–	Mixed diet	Mixed diet	–	Mixed diet	Mixed diet	–

Continuous variables that obey normal distribution were compared using the student t's test, otherwise the Wilcoxon rank sum test was used.

Fisher's exact test compared categorical variables in a 2×2 table.

BMI, body mass index; AFP, alpha-fetoprotein; ALT, alanine aminotransferase; AST, aspartate aminotransferase; GGT, glutamyl transpeptidase; HBV, hepatitis B virus;

HCC, hepatocellular carcinoma; ND, no detection.

supplementary Table S7 Different degree of phylum level (p value) in Healthy Control and HCC Group

feature	MEAN (HCC Group)	MEAN (Healthy Control)	P	MEAN (Non-Small-HCC)	MEAN (Small-HCC)	P	MEAN (Non-Cirrhosis)	MEAN (Cirrhosis)	P
Archaea|Euryarchaeota	1.00E-05	2.00E-05	0.9574	1.00E-05	0	0.7393	1.00E-05	0	0.8109
Bacteria|Actinobacteria	0.00813	0.00877	0.0928	0.00689	0.01078	0.8727	0.00815	0.00803	0.8561
Bacteria|Aquificae	0	0	1	0	0	1	0	0	1
Bacteria|Bacteroidetes	0.54572	0.52213	0.3226	0.51996	0.60082	0.0098	0.53652	0.58375	0.3496
Bacteria|Cyanobacteria/Chloroplast	3.00E-05	2.00E-05	0.9751	4.00E-05	2.00E-05	0.4596	4.00E-05	0	0.5211
Bacteria|Deinococcus-Thermus	0	0	0.9113	0	0	0.8125	0	0	0.9364
Bacteria|Elusimicrobia	0	0	1	0	0	1	0	0	1
Bacteria|Firmicutes	0.3282	0.34183	0.6131	0.34362	0.29522	0.1161	0.32964	0.32222	0.9769
Bacteria|Fusobacteria	0.02302	0.0335	0.5723	0.03101	0.00595	0.0402	0.02604	0.01053	0.3609
Bacteria|Gemmatimonadetes	0	0	1	0	0	1	0	0	1
Bacteria|Lentisphaerae	0.00023	0.00056	0.0375	0.00015	0.00039	0.2948	0.00027	5.00E-05	0.557
Bacteria|Proteobacteria	0.09274	0.08831	0.5183	0.09596	0.08585	0.4862	0.09767	0.07235	0.149
Bacteria|Spirochaetes	2.00E-05	0	0.8237	2.00E-05	4.00E-05	0.9019	3.00E-05	0	0.8733
Bacteria|Synergistetes	2.00E-05	0.00027	0.0167	2.00E-05	2.00E-05	0.5296	2.00E-05	2.00E-05	0.8334
Bacteria|Tenericutes	0.00051	0	0.8237	0.00074	0	0.8244	0	0.0026	0.5093
Bacteria|TM7	0.00018	0.0001	0.0106	0.00023	7.00E-05	0.0017	0.00018	0.00017	0.8165
Bacteria|Verrucomicrobia	0.00098	0.00395	0.0151	0.00126	0.00037	0.4766	0.0012	4.00E-05	0.4167
Unclassified	0	0	0.9308	1.00E-05	0	0.9877	1.00E-05	0	0.8109

supplementary Table S8 Different degree of genera level (p value) in Healthy Control and HCC Group

feature	MEAN (HCC Group)	MEAN (Healthy Control)	P	MEAN (non-small-hcc)	MEAN (small-hcc)	P	MEAN (non-cirrhosis)	MEAN (cirrhosis)	P
Archaea|Euryarchaeota|Methanobacteria|Methanobacteriales|Methanobacteriaceae|Methanobrevibacter	0.00001	0.00002	0.9574	1.00E-05	0	0.7393	1.00E-05	0	0.8109
Archaea|Euryarchaeota|Methanomicrobia|Methanosarcinales|Methanosarcinaceae|Methanosarcina	0	0	1	0	0	1	0	0	1
Bacteria|Actinobacteria|Actinobacteria|Actinomycetales|Actinomycetaceae|Actinomyces	0.00005	0.00005	0.906	4.00E-05	7.00E-05	0.8461	4.00E-05	8.00E-05	0.7607
Bacteria|Actinobacteria|Actinobacteria|Actinomycetales|Actinomycetaceae|Mobiluncus	0	0	1	0	0	1	0	0	1
Bacteria|Actinobacteria|Actinobacteria|Actinomycetales|Cellulomonadaceae|Tropheryma	0	0	1	0	0	1	0	0	1
Bacteria|Actinobacteria|Actinobacteria|Actinomycetales|Corynebacteriaceae|Corynebacterium	0	0	0.8013	0	0	1	0	0	1
Bacteria|Actinobacteria|Actinobacteria|Actinomycetales|Geodermatophilaceae|Blastococcus	0	0	1	0	0	1	0	0	1
Bacteria|Actinobacteria|Actinobacteria|Actinomycetales|Intrasporangiaceae|Janibacter	0	0	1	0	0	1	0	0	1
Bacteria|Actinobacteria|Actinobacteria|Actinomycetales|Micrococcaceae|Arthrobacter	0	0	1	0	0	1	0	0	1
Bacteria|Actinobacteria|Actinobacteria|Actinomycetales|Micrococcaceae|Kocuria	0	0	1	0	0	1	0	0	1
Bacteria|Actinobacteria|Actinobacteria|Actinomycetales|Micrococcaceae|Micrococcus	0	0	0.8998	0	0	1	0	0	1
Bacteria|Actinobacteria|Actinobacteria|Actinomycetales|Micrococcaceae|Rothia	0.00005	0.00002	0.2066	3.00E-05	9.00E-05	0.751	3.00E-05	0.00013	0.7969
Bacteria|Actinobacteria|Actinobacteria|Actinomycetales|Nocardiaceae|Rhodococcus	0	0	1	0	0	1	0	0	1
Bacteria|Actinobacteria|Actinobacteria|Bifidobacteriales|Bifidobacteriaceae|Alloscardovia	0.00001	0	0.6559	1.00E-05	0	0.6573	1.00E-05	1.00E-05	0.9307
Bacteria|Actinobacteria|Actinobacteria|Bifidobacteriales|Bifidobacteriaceae|Bifidobacterium	0.00427	0.00633	0.276	0.00453	0.00372	0.8873	0.00405	0.00517	0.7997
Bacteria|Actinobacteria|Actinobacteria|Bifidobacteriales|Bifidobacteriaceae|Gardnerella	0	0	0.8998	0	0	1	0	0	1
Bacteria|Actinobacteria|Actinobacteria|Bifidobacteriales|Bifidobacteriaceae|Scardovia	0	0	0.921	0	1.00E-05	0.897	0	0	0.8733
Bacteria|Actinobacteria|Actinobacteria|Coriobacteriales|Coriobacteriaceae|Adlercreutzia	0.00001	0.00009	0.2702	1.00E-05	1.00E-05	0.9754	1.00E-05	0	0.6322
Bacteria|Actinobacteria|Actinobacteria|Coriobacteriales|Coriobacteriaceae|Atopobium	0.00001	0	0.5898	1.00E-05	0	0.664	1.00E-05	2.00E-05	0.7662

Bacteria|Actinobacteria|Actinobacteria|Coriobacteriales|Coriobacteriaceae|Collinsella	0.0015	0.00152	0.0252	0.00137	0.00178	0.644	0.00136	0.00207	0.5766
Bacteria|Actinobacteria|Actinobacteria|Coriobacteriales|Coriobacteriaceae|Coriobacterium	0.00001	0.00001	0.9645	1.00E-05	0	0.7393	0	4.00E-05	0.5619
Bacteria|Actinobacteria|Actinobacteria|Coriobacteriales|Coriobacteriaceae|Cryptobacterium	0	0	1	0	0	1	0	0	1
Bacteria|Actinobacteria|Actinobacteria|Coriobacteriales|Coriobacteriaceae|Eggerthella	0.00015	0.00037	0.4663	0.00021	2.00E-05	0.0641	0.00014	0.00017	0.8081
Bacteria|Actinobacteria|Actinobacteria|Coriobacteriales|Coriobacteriaceae|Enterorhabdus	0.00016	0	0.6559	0.0002	6.00E-05	0.8101	0.00017	0.0001	0.9249
Bacteria|Actinobacteria|Actinobacteria|Coriobacteriales|Coriobacteriaceae|Gordonibacter	0.00002	0.00006	0.3215	3.00E-05	1.00E-05	0.575	2.00E-05	3.00E-05	0.4421
Bacteria|Actinobacteria|Actinobacteria|Coriobacteriales|Coriobacteriaceae|Olsenella	0.00001	0.00002	0.8255	1.00E-05	0	0.8268	1.00E-05	1.00E-05	0.9798
Bacteria|Actinobacteria|Actinobacteria|Coriobacteriales|Coriobacteriaceae|Paraeggerthella	0.00001	0.00003	0.5822	2.00E-05	0	0.9926	1.00E-05	0	0.8109
Bacteria|Actinobacteria|Actinobacteria|Coriobacteriales|Coriobacteriaceae|Slackia	0.00178	0.00002	0.7619	0.00033	0.0049	0.6979	0.00219	0.00011	0.7333
Bacteria|Actinobacteria|Actinobacteria|Rubrobacterales|Rubrobacteraceae|Rubrobacter	0	0	1	0	0	1	0	0	1
Bacteria|Aquificae|Aquificae|Aquificales|Aquificaceae|Hydrogenobacter	0	0	1	0	0	1	0	0	1
Bacteria|Bacteroidetes|Bacteroidia|Bacteroidales|Bacteroidaceae|Anaerorhabdus	0.00009	0.00004	0.914	0.00012	1.00E-05	0.3302	0.0001	2.00E-05	0.8847
Bacteria|Bacteroidetes|Bacteroidia|Bacteroidales|Bacteroidaceae|Bacteroides	0.28887	0.22978	0.0443	0.32045	0.22132	0.0152	0.27587	0.34263	0.3135
Bacteria|Bacteroidetes|Bacteroidia|Bacteroidales|Marinilabiaceae|Anaerophaga	0.00002	0	0.9113	0	7.00E-05	0.8125	3.00E-05	0	0.9364
Bacteria|Bacteroidetes|Bacteroidia|Bacteroidales|Porphyromonadaceae|Barnesiella	0.00271	0.0076	0.2844	0.00219	0.00383	0.089	0.00311	0.00108	0.2123
Bacteria|Bacteroidetes|Bacteroidia|Bacteroidales|Porphyromonadaceae|Butyricimonas	0.00228	0.0035	0.008	0.00244	0.00193	0.9215	0.00239	0.0018	0.4728
Bacteria|Bacteroidetes|Bacteroidia|Bacteroidales|Porphyromonadaceae|Dysgonomonas	0.00014	0	0.9113	0	0.00045	0.8125	0.00018	0	0.9364
Bacteria|Bacteroidetes|Bacteroidia|Bacteroidales|Porphyromonadaceae|Odoribacter	0.00212	0.00258	0.1533	0.00225	0.00183	0.9264	0.00231	0.00132	0.0575
Bacteria|Bacteroidetes|Bacteroidia|Bacteroidales|Porphyromonadaceae|Parabacteroides	0.01041	0.01572	0.1385	0.01237	0.00621	0.006	0.00995	0.01234	0.0556
Bacteria|Bacteroidetes|Bacteroidia|Bacteroidales|Porphyromonadaceae|Porphyromonas	0.00001	0.00001	0.8621	1.00E-05	1.00E-05	0.8824	1.00E-05	0	0.69
Bacteria|Bacteroidetes|Bacteroidia|Bacteroidales|Porphyromonadaceae|Tannerella	0	0.00013	0.6147	0	0	1	0	0	1
Bacteria|Bacteroidetes|Bacteroidia|Bacteroidales|Prevotellaceae|Hallella	0.00018	0	0.5039	0.00014	0.00026	0.707	0.00022	0	0.6322

Bacteria|Bacteroidetes|Bacteroidia|Bacteroidales|Prevotellaceae|Paraprevotella	0.00702	0.00448	0.4448	0.00749	0.00603	0.1197	0.00795	0.00316	0.5047
Bacteria|Bacteroidetes|Bacteroidia|Bacteroidales|Prevotellaceae|Prevotella	0.21243	0.22791	0.7721	0.15355	0.33836	0.0113	0.21543	0.20003	0.6194
Bacteria|Bacteroidetes|Bacteroidia|Bacteroidales|Rikenellaceae|Alistipes	0.01378	0.02524	0.0003	0.0131	0.01523	0.3521	0.01378	0.0138	0.2344
Bacteria|Bacteroidetes|Flavobacteria|Flavobacteriales|Flavobacteriaceae|Capnocytophaga	0	0	1	0	0	1	0	0	1
Bacteria|Bacteroidetes|Flavobacteria|Flavobacteriales|Flavobacteriaceae|Chryseobacterium	0	0	1	0	0	1	0	0	1
Bacteria|Bacteroidetes|Flavobacteria|Flavobacteriales|Flavobacteriaceae|Cloacibacterium	0	0	0.8013	0	0	1	0	0	1
Bacteria|Bacteroidetes|Flavobacteria|Flavobacteriales|Flavobacteriaceae|Planobacterium	0	0	1	0	0	1	0	0	1
Bacteria|Bacteroidetes|Sphingobacteria|Sphingobacteriales|Chitinophagaceae|Sediminibacterium	0	0	1	0	0	1	0	0	1
Bacteria|Bacteroidetes|Sphingobacteria|Sphingobacteriales|Sphingobacteriaceae|Pedobacter	0	0	0.9113	0	0	0.9117	0	0	0.9364
Bacteria|Bacteroidetes|Sphingobacteria|Sphingobacteriales|Sphingobacteriaceae|Sphingobacterium	0	0	1	0	0	1	0	0	1
Bacteria|Cyanobacteria/Chloroplast|Chloroplast|Chloroplast|Chloroplast|Streptophyta	0.00003	0.00002	0.9751	4.00E-05	2.00E-05	0.4596	4.00E-05	0	0.5211
Bacteria|Deinococcus-Thermus|Deinococci|Thermales|Thermaceae|Thermus	0	0	0.9113	0	0	0.8125	0	0	0.9364
Bacteria|Elusimicrobia|Elusimicrobia|Elusimicrobiales|Elusimicrobiaceae|Elusimicrobium	0	0	1	0	0	1	0	0	1
Bacteria|Firmicutes|Bacilli|Bacillales|Bacillaceae 1|Bacillus	0	0	1	0	0	1	0	0	1
Bacteria|Firmicutes|Bacilli|Bacillales|Bacillales_Incertae Sedis XI|Gemella	0.00002	0.00001	0.2972	3.00E-05	2.00E-05	0.73	2.00E-05	2.00E-05	0.9191
Bacteria|Firmicutes|Bacilli|Bacillales|Bacillales_Incertae Sedis XII|Exiguobacterium	0	0	0.9113	0	0	0.9117	0	0	0.9364
Bacteria|Firmicutes|Bacilli|Bacillales|Staphylococcaceae|Staphylococcus	0	0	1	0	0	1	0	0	1
Bacteria|Firmicutes|Bacilli|Lactobacillales|Aerococcaceae|Abiotrophia	0	0	0.9574	1.00E-05	0	0.6573	0	0	0.9307
Bacteria|Firmicutes|Bacilli|Lactobacillales|Aerococcaceae|Aerococcus	0	0	1	0	0	1	0	0	1
Bacteria|Firmicutes|Bacilli|Lactobacillales|Carnobacteriaceae|Atopostipes	0	0	1	0	0	1	0	0	1
Bacteria|Firmicutes|Bacilli|Lactobacillales|Carnobacteriaceae|Dolosigranulum	0	0	0.9113	0	0	0.9117	0	0	0.9364
Bacteria|Firmicutes|Bacilli|Lactobacillales|Carnobacteriaceae|Granulicatella	0.00005	0.00003	0.1777	5.00E-05	4.00E-05	0.5855	5.00E-05	3.00E-05	0.5841
Bacteria|Firmicutes|Bacilli|Lactobacillales|Enterococcaceae|Enterococcus	0.00014	0.00001	0.5197	0.0002	2.00E-05	0.8922	5.00E-05	0.0005	0.8448

Bacteria|Firmicutes|Bacilli|Lactobacillales|Enterococcaceae|Vagococcus	0.00007	0	0.7383	0.0001	3.00E-05	0.7208	9.00E-05	0	0.8109
Bacteria|Firmicutes|Bacilli|Lactobacillales|Lactobacillaceae|Lactobacillus	0.0011	0.00009	0.0158	0.00152	0.00019	0.6797	0.00131	0.00023	0.0619
Bacteria|Firmicutes|Bacilli|Lactobacillales|Lactobacillaceae|Pediococcus	0	0	1	0	0	1	0	0	1
Bacteria|Firmicutes|Bacilli|Lactobacillales|Leuconostocaceae|Leuconostoc	0	0	0.9325	1.00E-05	0	0.9926	0	2.00E-05	0.5619
Bacteria|Firmicutes|Bacilli|Lactobacillales|Leuconostocaceae|Weissella	0.00016	0	0.4356	0.00023	1.00E-05	0.9166	0.00019	1.00E-05	0.8109
Bacteria|Firmicutes|Bacilli|Lactobacillales|Streptococcaceae|Lactococcus	0	0	0.7909	0	0	0.9117	0	0	0.7415
Bacteria|Firmicutes|Bacilli|Lactobacillales|Streptococcaceae|Streptococcus	0.00734	0.00095	0.0974	0.01021	0.00121	0.0715	0.0083	0.00341	0.2688
Bacteria|Firmicutes|Clostridia|Clostridiales|Clostridiaceae 1|Anaerosporobacter	0	0	0.9893	0	1.00E-05	0.8125	0	0	0.9364
Bacteria|Firmicutes|Clostridia|Clostridiales|Clostridiaceae 1|Clostridium sensu stricto	0.00234	0.00178	0.0044	0.00302	0.00087	0.1359	0.00245	0.00189	0.0599
Bacteria|Firmicutes|Clostridia|Clostridiales|Clostridiales_Incertae Sedis XI|Anaerococcus	0	0	1	0	0	1	0	0	1
Bacteria|Firmicutes|Clostridia|Clostridiales|Clostridiales_Incertae Sedis XI|Finegoldia	0	0	1	0	0	1	0	0	1
Bacteria|Firmicutes|Clostridia|Clostridiales|Clostridiales_Incertae Sedis XI|Parvimonas	0	0.00001	0.8866	0	0	0.9117	0	0	0.9364
Bacteria|Firmicutes|Clostridia|Clostridiales|Clostridiales_Incertae Sedis XI|Peptoniphilus	0	0	0.8013	0	0	1	0	0	1
Bacteria|Firmicutes|Clostridia|Clostridiales|Clostridiales_Incertae Sedis XI|Tepidimicrobium	0	0	1	0	0	1	0	0	1
Bacteria|Firmicutes|Clostridia|Clostridiales|Clostridiales_Incertae Sedis XI|Tissierella	0.00001	0	0.9113	1.00E-05	0	0.9117	1.00E-05	0	0.9364
Bacteria|Firmicutes|Clostridia|Clostridiales|Clostridiales_Incertae Sedis XIII|Mogibacterium	0	0.00001	0.9379	0	0	0.7393	0	0	0.8109
Bacteria|Firmicutes|Clostridia|Clostridiales|Eubacteriaceae|Anaerofustis	0	0	0.7927	0	0	0.9117	0	0	0.9364
Bacteria|Firmicutes|Clostridia|Clostridiales|Eubacteriaceae|Eubacterium	0.00008	0.00011	0.1254	9.00E-05	5.00E-05	0.0445	8.00E-05	5.00E-05	0.9076
Bacteria|Firmicutes|Clostridia|Clostridiales|Incertae Sedis XI|Murdochiella	0	0	1	0	0	1	0	0	1
Bacteria|Firmicutes|Clostridia|Clostridiales|Lachnospiraceae|Acetitomaculum	0	0	1	0	0	1	0	0	1
Bacteria|Firmicutes|Clostridia|Clostridiales|Lachnospiraceae|Anaerostipes	0.00087	0.00287	0.0124	0.00096	0.00068	0.3521	0.00074	0.00145	0.7007
Bacteria|Firmicutes|Clostridia|Clostridiales|Lachnospiraceae|Blautia	0.00483	0.00537	0.9343	0.00553	0.00335	0.0152	0.0049	0.00456	0.6635

Bacteria|Firmicutes|Clostridia|Clostridiales|Lachnospiraceae|Butyrivibrio	0	0	1	0	0	1	0	0	1
Bacteria|Firmicutes|Clostridia|Clostridiales|Lachnospiraceae|Cellulosilyticum	0.00003	0	0.6689	5.00E-05	0	0.5792	4.00E-05	1.00E-05	0.6847
Bacteria|Firmicutes|Clostridia|Clostridiales|Lachnospiraceae|Clostridium XlVa	0.0305	0.02461	0.015	0.03412	0.02277	0.0168	0.02974	0.03367	0.622
Bacteria|Firmicutes|Clostridia|Clostridiales|Lachnospiraceae|Clostridium XlVb	0.00346	0.00314	0.2583	0.00423	0.00182	0.1091	0.00361	0.00283	0.2273
Bacteria|Firmicutes|Clostridia|Clostridiales|Lachnospiraceae|Coprococcus	0.00459	0.00742	0.0777	0.00442	0.00495	0.973	0.00482	0.00362	0.769
Bacteria|Firmicutes|Clostridia|Clostridiales|Lachnospiraceae|Dorea	0.00128	0.00246	0.0029	0.00149	0.00085	0.079	0.00127	0.00133	0.8165
Bacteria|Firmicutes|Clostridia|Clostridiales|Lachnospiraceae|Howardella	0.00003	0.00001	0.3923	2.00E-05	4.00E-05	0.8437	3.00E-05	3.00E-05	0.9769
Bacteria|Firmicutes|Clostridia|Clostridiales|Lachnospiraceae|Lachnospiracea_incertae_sedis	0.06394	0.04201	0.0393	0.07214	0.04639	0.0249	0.05907	0.08406	0.2865
Bacteria|Firmicutes|Clostridia|Clostridiales|Lachnospiraceae|Moryella	0	0	1	0	0	1	0	0	1
Bacteria|Firmicutes|Clostridia|Clostridiales|Lachnospiraceae|Oribacterium	0.00001	0.00001	0.9982	2.00E-05	0	0.3732	2.00E-05	0	0.64
Bacteria|Firmicutes|Clostridia|Clostridiales|Lachnospiraceae|Parasporobacterium	0.00001	0.00003	0.5255	2.00E-05	0	0.6729	1.00E-05	2.00E-05	0.8933
Bacteria|Firmicutes|Clostridia|Clostridiales|Lachnospiraceae|Pseudobutyrivibrio	0.00025	0.00034	0.1168	0.00029	0.00018	0.3537	0.0003	8.00E-05	0.2259
Bacteria|Firmicutes|Clostridia|Clostridiales|Lachnospiraceae|Roseburia	0.0433	0.03099	0.1527	0.04643	0.03662	0.8849	0.0447	0.03752	0.4508
Bacteria|Firmicutes|Clostridia|Clostridiales|Lachnospiraceae|Shuttleworthia	0	0	0.6559	1.00E-05	0	0.6573	0	1.00E-05	0.6168
Bacteria|Firmicutes|Clostridia|Clostridiales|Peptococcaceae 1|Peptococcus	0.00008	0.00011	0.1902	8.00E-05	7.00E-05	0.9607	6.00E-05	0.00015	0.3312
Bacteria|Firmicutes|Clostridia|Clostridiales|Peptostreptococcaceae|Clostridium XI	0.00344	0.00167	0.0428	0.00368	0.00291	0.6707	0.00332	0.00394	0.5668
Bacteria|Firmicutes|Clostridia|Clostridiales|Peptostreptococcaceae|Peptostreptococcus	0	0.00001	0.5898	0	0	0.9926	0	0	0.859
Bacteria|Firmicutes|Clostridia|Clostridiales|Ruminococcaceae|Acetanaerobacterium	0.00062	0.00199	0.1592	0.00055	0.00075	0.4302	0.00068	0.00034	0.5816
Bacteria|Firmicutes|Clostridia|Clostridiales|Ruminococcaceae|Acetivibrio	0.00007	0.00004	0.8185	8.00E-05	4.00E-05	0.9411	9.00E-05	0	0.5766
Bacteria|Firmicutes|Clostridia|Clostridiales|Ruminococcaceae|Anaerofilum	0.00002	0.00003	0.9272	2.00E-05	1.00E-05	0.5156	2.00E-05	2.00E-05	0.6688
Bacteria|Firmicutes|Clostridia|Clostridiales|Ruminococcaceae|Anaerotruncus	0.00033	0.00046	0.0077	0.00023	0.00054	0.8533	0.00036	0.00024	0.653
Bacteria|Firmicutes|Clostridia|Clostridiales|Ruminococcaceae|Butyricicoccus	0.00194	0.0027	0.0076	0.00221	0.00135	0.0522	0.002	0.00169	0.2672
Bacteria|Firmicutes|Clostridia|Clostridiales|Ruminococcaceae|Clostridium III	0	0.00001	0.6843	1.00E-05	0	0.8244	1.00E-05	0	0.8733

Bacteria|Firmicutes|Clostridia|Clostridiales|Ruminococcaceae|Clostridium IV	0.00352	0.00606	0.097	0.0037	0.00313	0.0681	0.00346	0.00374	0.7997
Bacteria|Firmicutes|Clostridia|Clostridiales|Ruminococcaceae|Faecalibacterium	0.04488	0.05729	0.4111	0.04482	0.04502	0.7721	0.04723	0.03519	0.4751
Bacteria|Firmicutes|Clostridia|Clostridiales|Ruminococcaceae|Flavonifractor	0.00356	0.00395	0.6336	0.00395	0.00272	0.1528	0.00386	0.00231	0.1372
Bacteria|Firmicutes|Clostridia|Clostridiales|Ruminococcaceae|Hydrogenoanaerobacterium	0.00001	0.00005	0.0764	2.00E-05	1.00E-05	0.9068	2.00E-05	0	0.6741
Bacteria|Firmicutes|Clostridia|Clostridiales|Ruminococcaceae|Oscillibacter	0.0108	0.01923	0.0019	0.01068	0.01105	0.5876	0.01102	0.00991	0.5891
Bacteria|Firmicutes|Clostridia|Clostridiales|Ruminococcaceae|Papillibacter	0	0.00001	0.8884	0	0	0.8125	0	0	0.9364
Bacteria|Firmicutes|Clostridia|Clostridiales|Ruminococcaceae|Pseudoflavonifractor	0.00037	0.0003	0.135	0.0004	0.00031	0.751	0.00042	0.00019	0.4773
Bacteria|Firmicutes|Clostridia|Clostridiales|Ruminococcaceae|Ruminococcus	0.01234	0.01276	0.135	0.01247	0.01207	0.1327	0.0136	0.00712	0.8962
Bacteria|Firmicutes|Clostridia|Clostridiales|Ruminococcaceae|Saccharofermentans	0	0.00001	0.7585	0	0	0.9877	0	0	0.8109
Bacteria|Firmicutes|Clostridia|Clostridiales|Ruminococcaceae|Sporobacter	0.00125	0.00278	0.0084	0.00111	0.00157	0.27	0.00139	0.0007	0.9191
Bacteria|Firmicutes|Clostridia|Clostridiales|Syntrophomonadaceae|Syntrophomonas	0	0	1	0	0	1	0	0	1
Bacteria|Firmicutes|Erysipelotrichia|Erysipelotrichales|Erysipelotrichaceae|Allobaculum	0.00002	0.00013	0.6053	0	5.00E-05	0.8125	2.00E-05	0	0.9364
Bacteria|Firmicutes|Erysipelotrichia|Erysipelotrichales|Erysipelotrichaceae|Bulleidia	0	0	0.9893	0	0	0.9117	0	0	0.9364
Bacteria|Firmicutes|Erysipelotrichia|Erysipelotrichales|Erysipelotrichaceae|Catenibacterium	0.00015	0.0002	0.66	3.00E-05	0.00039	0.3211	0.00015	0.00012	0.4043
Bacteria|Firmicutes|Erysipelotrichia|Erysipelotrichales|Erysipelotrichaceae|Clostridium XVIII	0.00502	0.00465	0.9609	0.00539	0.00424	0.0315	0.005	0.00511	0.4796
Bacteria|Firmicutes|Erysipelotrichia|Erysipelotrichales|Erysipelotrichaceae|Coprobacillus	0.00022	0.00018	0.1995	0.00028	7.00E-05	0.744	0.00025	7.00E-05	0.7333
Bacteria|Firmicutes|Erysipelotrichia|Erysipelotrichales|Erysipelotrichaceae|Erysipelotrichaceae_incertae_sedis	0.00268	0.00475	0.8428	0.00268	0.00269	0.5337	0.00264	0.00285	0.7278
Bacteria|Firmicutes|Erysipelotrichia|Erysipelotrichales|Erysipelotrichaceae|Holdemania	0.00027	0.0003	0.1427	0.00028	0.00022	0.6617	0.0003	0.0001	0.0897
Bacteria|Firmicutes|Erysipelotrichia|Erysipelotrichales|Erysipelotrichaceae|Solobacterium	0.00003	0.00001	0.3605	2.00E-05	3.00E-05	0.9656	2.00E-05	5.00E-05	0.7997
Bacteria|Firmicutes|Erysipelotrichia|Erysipelotrichales|Erysipelotrichaceae|Turicibacter	0.00031	0.00011	0.2205	0.00027	0.0004	0.6573	0.00024	0.00063	0.0751
Bacteria|Firmicutes|Negativicutes|Selenomonadales|Acidaminococcaceae|Acidaminococcus	0.00022	0.00007	0.364	0.00026	0.00016	0.5666	0.00012	0.00064	0.4773
Bacteria|Firmicutes|Negativicutes|Selenomonadales|Acidaminococcaceae|Phascolarctobacterium	0.0242	0.02581	0.4054	0.02189	0.02915	0.5666	0.02027	0.04046	0.2177

Bacteria|Firmicutes|Negativicutes|Selenomonadales|Veillonellaceae|Allisonella	0.00016	0.00027	0.1075	0.0002	7.00E-05	0.7393	0.00019	4.00E-05	0.3205
Bacteria|Firmicutes|Negativicutes|Selenomonadales|Veillonellaceae|Anaeroglobus	0.00001	0	0.8516	1.00E-05	1.00E-05	0.9019	1.00E-05	0	0.69
Bacteria|Firmicutes|Negativicutes|Selenomonadales|Veillonellaceae|Anaerovibrio	0	0	1	0	0	1	0	0	1
Bacteria|Firmicutes|Negativicutes|Selenomonadales|Veillonellaceae|Centipeda	0	0	0.8237	0	0	0.8244	0	0	0.8733
Bacteria|Firmicutes|Negativicutes|Selenomonadales|Veillonellaceae|Dialister	0.00432	0.01223	0.1068	0.00158	0.01017	0.1873	0.0051	0.00106	0.2549
Bacteria|Firmicutes|Negativicutes|Selenomonadales|Veillonellaceae|Megamonas	0.01071	0.00471	0.6681	0.00888	0.01462	0.2727	0.01089	0.00997	0.8109
Bacteria|Firmicutes|Negativicutes|Selenomonadales|Veillonellaceae|Megasphaera	0.0006	0.0022	0.1963	0.00059	0.00063	0.6933	0.00071	0.00016	0.6741
Bacteria|Firmicutes|Negativicutes|Selenomonadales|Veillonellaceae|Mitsuokella	0.00001	0.00049	0.7585	2.00E-05	0	0.9877	0	5.00E-05	0.5619
Bacteria|Firmicutes|Negativicutes|Selenomonadales|Veillonellaceae|Negativicoccus	0	0	0.8998	0	0	1	0	0	1
Bacteria|Firmicutes|Negativicutes|Selenomonadales|Veillonellaceae|Selenomonas	0.00001	0	0.5776	1.00E-05	0	0.5792	1.00E-05	0	0.69
Bacteria|Firmicutes|Negativicutes|Selenomonadales|Veillonellaceae|Veillonella	0.00412	0.00277	0.0009	0.00512	0.00197	0.3716	0.00483	0.00115	0.6168
Bacteria|Fusobacteria|Fusobacteria|Fusobacteriales|Fusobacteriaceae|Cetobacterium	0.00311	0.00384	0.7602	0.00451	0.00011	0.8824	0.00386	0	0.69
Bacteria|Fusobacteria|Fusobacteria|Fusobacteriales|Fusobacteriaceae|Fusobacterium	0.01978	0.02932	0.6178	0.0263	0.00584	0.0393	0.02215	0.00998	0.5473
Bacteria|Fusobacteria|Fusobacteria|Fusobacteriales|Leptotrichiaceae|Leptotrichia	0.00002	0	0.7594	4.00E-05	0	0.5057	3.00E-05	0	0.6322
Bacteria|Fusobacteria|Fusobacteria|Fusobacteriales|Leptotrichiaceae|Sneathia	0	0	1	0	0	1	0	0	1
Bacteria|Gemmatimonadetes|Gemmatimonadetes|Gemmatimonadales|Gemmatimonadaceae|Gemmatimonas	0	0	1	0	0	1	0	0	1
Bacteria|Lentisphaerae|Lentisphaeria|Victivallales|Victivallaceae|Victivallis	0.00023	0.00056	0.0375	0.00015	0.00039	0.2948	0.00027	5.00E-05	0.557
Bacteria|Proteobacteria|Alphaproteobacteria|Alphaproteobacteria_incertae_sedis|Geminicoccus|Geminicoccus	0	0	1	0	0	1	0	0	1
Bacteria|Proteobacteria|Alphaproteobacteria|Caulobacterales|Caulobacteraceae|Brevundimonas	0.00001	0.00001	0.8726	1.00E-05	1.00E-05	0.9705	0	1.00E-05	0.7333
Bacteria|Proteobacteria|Alphaproteobacteria|Rhizobiales|Bradyrhizobiaceae|Bradyrhizobium	0	0	1	0	0	1	0	0	1
Bacteria|Proteobacteria|Alphaproteobacteria|Rhizobiales|Brucellaceae|Brucella	0	0.00001	0.9751	0	1.00E-05	0.897	0	0	0.8733
Bacteria|Proteobacteria|Alphaproteobacteria|Rhizobiales|Hyphomicrobiaceae|Gemmiger	0.01981	0.02762	0.0619	0.01798	0.0237	0.0955	0.0201	0.01858	0.9884

Bacteria|Proteobacteria|Alphaproteobacteria|Rhizobiales|Methylobacteriaceae|Methylobacterium	0	0	0.8013	0	0	1	0	0	1
Bacteria|Proteobacteria|Alphaproteobacteria|Rhizobiales|Rhizobiaceae|Rhizobium	0	0	0.9361	0	0	0.9926	0	0	0.8109
Bacteria|Proteobacteria|Alphaproteobacteria|Rhodobacterales|Rhodobacteraceae|Paracoccus	0	0	1	0	0	1	0	0	1
Bacteria|Proteobacteria|Alphaproteobacteria|Rhodospirillales|Acetobacteraceae|Roseomonas	0	0	1	0	0	1	0	0	1
Bacteria|Proteobacteria|Alphaproteobacteria|Rhodospirillales|Rhodospirillaceae|Desertibacter	0	0	1	0	0	1	0	0	1
Bacteria|Proteobacteria|Alphaproteobacteria|Sphingomonadales|Sphingomonadaceae|Sphingobium	0	0	0.9113	0	0	0.9117	0	0	0.9364
Bacteria|Proteobacteria|Alphaproteobacteria|Sphingomonadales|Sphingomonadaceae|Sphingomonas	0	0	0.8998	0	0	1	0	0	1
Bacteria|Proteobacteria|Betaproteobacteria|Burkholderiales|Burkholderiaceae|Cupriavidus	0	0	0.8998	0	0	1	0	0	1
Bacteria|Proteobacteria|Betaproteobacteria|Burkholderiales|Burkholderiaceae|Limnobacter	0	0	1	0	0	1	0	0	1
Bacteria|Proteobacteria|Betaproteobacteria|Burkholderiales|Burkholderiales_incertae_sedis|Aquabacterium	0	0	1	0	0	1	0	0	1
Bacteria|Proteobacteria|Betaproteobacteria|Burkholderiales|Burkholderiales_incertae_sedis|Tepidimonas	0	0	1	0	0	1	0	0	1
Bacteria|Proteobacteria|Betaproteobacteria|Burkholderiales|Comamonadaceae|Brachymonas	0	0	1	0	0	1	0	0	1
Bacteria|Proteobacteria|Betaproteobacteria|Burkholderiales|Comamonadaceae|Comamonas	0.00001	0.00006	0.3432	1.00E-05	1.00E-05	0.9264	1.00E-05	1.00E-05	0.9307
Bacteria|Proteobacteria|Betaproteobacteria|Burkholderiales|Comamonadaceae|Delftia	0	0	1	0	0	1	0	0	1
Bacteria|Proteobacteria|Betaproteobacteria|Burkholderiales|Comamonadaceae|Diaphorobacter	0	0	1	0	0	1	0	0	1
Bacteria|Proteobacteria|Betaproteobacteria|Burkholderiales|Comamonadaceae|Variovorax	0	0	1	0	0	1	0	0	1
Bacteria|Proteobacteria|Betaproteobacteria|Burkholderiales|Oxalobacteraceae|Janthinobacterium	0	0	1	0	0	1	0	0	1
Bacteria|Proteobacteria|Betaproteobacteria|Burkholderiales|Sutterellaceae|Parasutterella	0.01089	0.01217	0.4048	0.01005	0.01268	0.2121	0.01168	0.00763	0.557
Bacteria|Proteobacteria|Betaproteobacteria|Burkholderiales|Sutterellaceae|Sutterella	0.01199	0.01582	0.561	0.01083	0.01446	0.7934	0.01371	0.00488	0.1584
Bacteria|Proteobacteria|Betaproteobacteria|Neisseriales|Neisseriaceae|Eikenella	0.00001	0	0.5776	1.00E-05	0	0.5792	0	2.00E-05	0.6583
Bacteria|Proteobacteria|Betaproteobacteria|Neisseriales|Neisseriaceae|Kingella	0	0	0.9113	0	0	0.9117	0	0	0.9364
Bacteria|Proteobacteria|Betaproteobacteria|Neisseriales|Neisseriaceae|Neisseria	0.00001	0.00001	0.5372	1.00E-05	1.00E-05	0.7024	1.00E-05	1.00E-05	0.9191

Bacteria|Proteobacteria|Deltaproteobacteria|Bdellovibrionales|Bdellovibrionaceae|Vampirovibrio	0.00039	0.00028	0.8708	0.0004	0.00037	0.9754	0.00045	0.00012	0.9422
Bacteria|Proteobacteria|Deltaproteobacteria|Desulfovibrionales|Desulfomicrobiaceae|Desulfomicrobium	0.00002	0.00008	0.9698	2.00E-05	0	0.8316	1.00E-05	5.00E-05	0.9798
Bacteria|Proteobacteria|Deltaproteobacteria|Desulfovibrionales|Desulfovibrionaceae|Bilophila	0.00226	0.00367	0.0015	0.00234	0.00208	0.5855	0.00244	0.00149	0.4595
Bacteria|Proteobacteria|Deltaproteobacteria|Desulfovibrionales|Desulfovibrionaceae|Desulfovibrio	0.00106	0.00202	0.0325	0.00068	0.00187	0.1265	0.00106	0.00105	0.1751
Bacteria|Proteobacteria|Epsilonproteobacteria|Campylobacterales|Campylobacteraceae|Campylobacter	0.00074	0.00002	0.1004	0.00108	3.00E-05	0.5337	0.00092	2.00E-05	0.4684
Bacteria|Proteobacteria|Epsilonproteobacteria|Campylobacterales|Helicobacteraceae|Helicobacter	0	0	1	0	0	1	0	0	1
Bacteria|Proteobacteria|Gammaproteobacteria|Aeromonadales|Aeromonadaceae|Aeromonas	0.00006	0	0.8237	9.00E-05	0	0.8244	8.00E-05	1.00E-05	0.8053
Bacteria|Proteobacteria|Gammaproteobacteria|Aeromonadales|Succinivibrionaceae|Succinatimonas	0	0	0.921	0	1.00E-05	0.9019	0	1.00E-05	0.7997
Bacteria|Proteobacteria|Gammaproteobacteria|Aeromonadales|Succinivibrionaceae|Succinivibrio	0.00005	0.00427	0.9698	8.00E-05	0	0.5792	2.00E-05	0.0002	0.6741
Bacteria|Proteobacteria|Gammaproteobacteria|Alteromonadales|Shewanellaceae|Shewanella	0	0	1	0	0	1	0	0	1
Bacteria|Proteobacteria|Gammaproteobacteria|Chromatiales|Chromatiaceae|Rheinheimera	0	0	1	0	0	1	0	0	1
Bacteria|Proteobacteria|Gammaproteobacteria|Enterobacteriales|Enterobacteriaceae|Citrobacter	0.00384	0.00047	0.0116	0.00522	0.00088	0.0433	0.00431	0.00191	0.7007
Bacteria|Proteobacteria|Gammaproteobacteria|Enterobacteriales|Enterobacteriaceae|Escherichia/Shigella	0.0265	0.01063	0.4515	0.02948	0.02011	0.1359	0.02702	0.02432	0.5234
Bacteria|Proteobacteria|Gammaproteobacteria|Enterobacteriales|Enterobacteriaceae|Hafnia	0.00001	0	0.9308	1.00E-05	0	0.7393	1.00E-05	0	0.8109
Bacteria|Proteobacteria|Gammaproteobacteria|Enterobacteriales|Enterobacteriaceae|Klebsiella	0.00055	0.00002	0.0008	0.0005	0.00065	0.0636	0.00047	0.00088	0.8675
Bacteria|Proteobacteria|Gammaproteobacteria|Enterobacteriales|Enterobacteriaceae|Morganella	0.00002	0	0.5082	2.00E-05	0	0.655	1.00E-05	3.00E-05	0.7773
Bacteria|Proteobacteria|Gammaproteobacteria|Enterobacteriales|Enterobacteriaceae|Plesiomonas	0.00002	0	0.7383	2.00E-05	0	0.7393	2.00E-05	0	0.8109
Bacteria|Proteobacteria|Gammaproteobacteria|Enterobacteriales|Enterobacteriaceae|Proteus	0.00058	0.00004	0.8691	0.0008	9.00E-05	0.9705	0.00072	0	0.9307
Bacteria|Proteobacteria|Gammaproteobacteria|Enterobacteriales|Enterobacteriaceae|Providencia	0.00026	0	0.7383	0.00038	0	0.7393	0	0.00133	0.859
Bacteria|Proteobacteria|Gammaproteobacteria|Enterobacteriales|Enterobacteriaceae|Serratia	0	0	0.9113	0	0	0.9117	0	0	0.9364
Bacteria|Proteobacteria|Gammaproteobacteria|Legionellales|Legionellaceae|Legionella	0	0	1	0	0	1	0	0	1
Bacteria|Proteobacteria|Gammaproteobacteria|Oceanospirillales|Halomonadaceae|Halomonas	0	0	1	0	0	1	0	0	1

Bacteria|Proteobacteria|Gammaproteobacteria|Pasteurellales|Pasteurellaceae|Aggregatibacter	0.00008	0.00001	0.1476	9.00E-05	3.00E-05	0.7815	9.00E-05	0	0.1931
Bacteria|Proteobacteria|Gammaproteobacteria|Pasteurellales|Pasteurellaceae|Haemophilus	0.0037	0.00134	0	0.00437	0.00227	0.2594	0.00366	0.00387	0.8733
Bacteria|Proteobacteria|Gammaproteobacteria|Pseudomonadales|Moraxellaceae|Acinetobacter	0	0	0.9893	0	0	0.8125	0	0	0.9364
Bacteria|Proteobacteria|Gammaproteobacteria|Pseudomonadales|Moraxellaceae|Enhydrobacter	0	0	1	0	0	1	0	0	1
Bacteria|Proteobacteria|Gammaproteobacteria|Pseudomonadales|Pseudomonadaceae|Pseudomonas	0.00001	0.00002	0.4048	1.00E-05	0	0.744	0	2.00E-05	0.4508
Bacteria|Proteobacteria|Gammaproteobacteria|Vibrionales|Vibrionaceae|Vibrio	0.00007	0	0.9113	0.00011	0	0.9117	9.00E-05	0	0.9364
Bacteria|Proteobacteria|Gammaproteobacteria|Xanthomonadales|Xanthomonadaceae|Lysobacter	0	0	1	0	0	1	0	0	1
Bacteria|Proteobacteria|Gammaproteobacteria|Xanthomonadales|Xanthomonadaceae|Stenotrophomonas	0	0.00001	0.7738	1.00E-05	0	0.9926	0	1.00E-05	0.8647
Bacteria|Spirochaetes|Spirochaetes|Spirochaetales|Spirochaetaceae|Treponema	0.00002	0	0.8237	2.00E-05	4.00E-05	0.9019	3.00E-05	0	0.8733
Bacteria|Synergistetes|Synergistia|Synergistales|Synergistaceae|Aminobacterium	0	0	1	0	0	1	0	0	1
Bacteria|Synergistetes|Synergistia|Synergistales|Synergistaceae|Cloacibacillus	0.00001	0.00008	0.1011	1.00E-05	1.00E-05	0.5541	1.00E-05	0	0.5766
Bacteria|Synergistetes|Synergistia|Synergistales|Synergistaceae|Pyramidobacter	0.00001	0.00019	0.2972	1.00E-05	1.00E-05	0.9362	1.00E-05	2.00E-05	0.7885
Bacteria|Tenericutes|Mollicutes|Anaeroplasmatales|Anaeroplasmataceae|Anaeroplasma	0	0	1	0	0	1	0	0	1
Bacteria|Tenericutes|Mollicutes|Anaeroplasmatales|Anaeroplasmataceae|Asteroleplasma	0.00051	0	0.8237	0.00074	0	0.8244	0	0.0026	0.5093
Bacteria|TM7|TM7_genera_incertae_sedis|TM7_genera_incertae_sedis|TM7_genera_incertae_sedis|TM7_genera_incertae_sedis	0.00018	0.0001	0.0106	0.00023	7.00E-05	0.0017	0.00018	0.00017	0.8165
Bacteria|Verrucomicrobia|Opitutae|Puniceicoccales|Puniceicoccaceae|Cerasicoccus	0	0.00002	0.6917	0	0	0.8125	0	0	0.9364
Bacteria|Verrucomicrobia|Opitutae|Puniceicoccales|Puniceicoccaceae|Coraliomargarita	0	0	0.8998	0	0	1	0	0	1
Bacteria|Verrucomicrobia|Subdivision3|Subdivision3_genera_incertae_sedis|Subdivision3_genera_incertae_sedis|Subdivision3_genera_incertae_sedis	0	0	1	0	0	1	0	0	1
Bacteria|Verrucomicrobia|Verrucomicrobiae|Verrucomicrobiales|Verrucomicrobiaceae|Akkermansia	0.00097	0.0039	0.0178	0.00126	0.00036	0.6351	0.0012	4.00E-05	0.4639
Unclassified	0	0	0.9308	1.00E-05	0	0.9877	1.00E-05	0	0.8109

supplementary Table S9 The interrelationship between 16S OTU clusters of at genus level and taxonomic compositions (NCBI Taxonomy ID)

***Clostridium* (cluster) XIVa [1,2]**		**NCBI Taxonomy ID**
Lachnospiraceae	Rainey 2010	txid186803
Acetitomaculum	Greening and Leedle 1995	txid31980
Acetitomaculum ruminis	Greening and Leedle 1995	txid2382
Anaerocolumna	Ueki et al. 2016	txid1843210
Anaerocolumna aminovalerica	Ueki et al. 2016	txid1527
Blautia	Durand et al. 2017	txid572511
Ruminococcus torques	Holdeman and Moore 1974	txid33039
Blautia coccoides	Liu et al. 2008	txid1532
Blautia hansenii	Liu et al. 2008	txid1322
Blautia producta	Liu et al. 2008	txid33035
Coprococcus	Holdeman and Moore 1974	txid33042
Coprococcus eutactus	Holdeman and Moore 1974	txid33043
Faecalicatena	Sakamoto et al. 2017	txid2005359
Faecalicatena orotica	Sakamoto et al. 2017	txid1544
Lachnoclostridium	Yutin and Galperin 2013	txid1506553
Clostridium aerotolerans	Chamkha et al. 2001	txid36832
Clostridium aldenense	Warren et al. 2007	txid358742
Clostridium aminophilum	Paster et al. 1993	txid1526
Clostridium asparagiforme	Mohan et al. 2007	txid333367
Clostridium bolteae	Song et al. 2003	txid208479
Clostridium celerecrescens	Chamkha et al. 2001	txid29354
Clostridium citroniae	Warren et al. 2007	txid358743
Clostridium clostridioforme	Kaneuchi et al. 1976	txid1531
Clostridium herbivorans	Varel et al. 1995	txid39479
Clostridium lavalense	Domingo et al. 2009	txid460384
Clostridium polysaccharolyticum	Gylswyk et al. 1983	txid29364
Clostridium populeti	Sleat and Mah 1985	txid37658
Clostridium sphenoides	Bergey et al. 1923	txid29370
Clostridium symbiosum	Kaneuchi et al. 1976	txid1512
Clostridium xylanolyticum	Chamkha et al. 2001	txid29375
Roseburia	Stanton and Savage 1983	txid841
Roseburia cecicola	Stanton and Savage 1983	txid842
Syntrophococcus	Krumholz and Bryant 1986	txid84036
Tyzzerella	Yutin and Galperin 2013	txid1506577
Tyzzerella nexilis	Yutin and Galperin 2013	txid29361

Bacteroides		**NCBI Taxonomy ID**
Bacteroidaceae	Pribram 1933	txid815
Bacteroides	Castellani and Chalmers 1919	txid816

Lachnospiracea_incertae_sedis		**NCBI Taxonomy ID**
Lachnospiraceae	Rainey 2010	txid186803
unclassified Lachnospiraceae	/	txid186928

[1] Collins MD, Lawson PA, Willems A, et al. The phylogeny of the genus Clostridium: proposal of five new genera and eleven new species combinations. Int J Syst Bacteriol. 1994; 44: 812-26.

[2] Taxonomy of Clostridium clusters XIVa and IV. (https://help.ezbiocloud.net/taxonomy-of-clostridium-cluster-xiva-iv)

supplementary Table S10 fifty-six OTU-gene pairs filtered by FDR test of Pearson correlation between OTUs and genes

ENSEMBL GENE ID	OFFICIAL SYMBOL	OTU ANNOTATION	PEARSON CORRELATION	Q VALUE	P VALUE
ENSG00000013725.14	CD6	OTU_0033	-0.778698695	0.00371433	0.003431859
ENSG00000013725.14	CD6	OTU_0134	-0.785889637	0.003461228	0.003431859
ENSG00000015133.19	CCDC88C	OTU_0134	-0.722686957	0.027071714	0.004619884
ENSG00000072818.12	ACAP1	OTU_0134	-0.702825707	0.039856216	0.001775613
ENSG00000078589.13	P2RY10	OTU_0134	-0.82115452	0.000777381	0.022303592
ENSG00000100979.15	PLTP	OTU_0134	-0.695825993	0.046605455	0.033251176
ENSG00000102349.18	KLF8	OTU_0002	-0.692171077	0.048100171	0.00631544
ENSG00000102879.15	CORO1A	OTU_0134	-0.694079385	0.047867613	0.013365886
ENSG00000104814.13	MAP4K1	OTU_0794	-0.712881255	0.032671106	0.000934728
ENSG00000104938.16	CLEC4M	OTU_0067	0.81253542	0.001317141	0.045845692
ENSG00000105122.13	RASAL3	OTU_0134	-0.766118949	0.007129631	0.022207023
ENSG00000105639.18	JAK3	OTU_0134	-0.760275272	0.008527813	0.014517514
ENSG00000109339.22	MAPK10	OTU_0002	-0.692079645	0.048286496	0.04154831
ENSG00000113263.12	ITK	OTU_0134	-0.766874213	0.007033937	0.014734987
ENSG00000116824.5	CD2	OTU_0134	-0.724450086	0.025961809	0.012618457
ENSG00000120057.5	SFRP5	OTU_0067	0.711618773	0.033594638	0.025944768
ENSG00000124256.15	ZBP1	OTU_0134	-0.730978128	0.021921734	0.003947478
ENSG00000134242.16	PTPN22	OTU_0134	-0.7389057	0.017566106	0.002158303
ENSG00000136286.16	MYO1G	OTU_0134	-0.762875454	0.007799183	0.001723739
ENSG00000136490.9	LIMD2	OTU_0134	-0.765377047	0.00725448	0.007057414
ENSG00000137077.8	CCL21	OTU_0033	-0.694674829	0.047732763	3.07859E-07
ENSG00000138964.17	PARVG	OTU_0134	-0.731427401	0.021708977	0.003090708
ENSG00000140368.12	PSTPIP1	OTU_0134	-0.773489264	0.005696178	0.045070103
ENSG00000142748.12	FCN3	OTU_0067	0.755974662	0.009589407	0.018744759
ENSG00000142765.18	SYTL1	OTU_0134	-0.760106359	0.008527813	0.036608722
ENSG00000143369.15	ECM1	OTU_0067	0.726560694	0.024593671	0.005057125
ENSG00000147138.2	GPR174	OTU_0134	-0.718904813	0.028837769	0.010636931
ENSG00000150782.12	IL18	OTU_0033	-0.695751964	0.047642763	0.007017704
ENSG00000153253.17	SCN3A	OTU_1149	-0.750322902	0.011190361	0.022997997
ENSG00000153283.12	CD96	OTU_0134	-0.766790234	0.007033937	0.010791134
ENSG00000154914.17	USP43	OTU_0175	-0.722069833	0.027071714	0.043240802
ENSG00000154914.17	USP43	OTU_0244	-0.719250837	0.028569082	0.043240802
ENSG00000159753.14	CARMIL2	OTU_0134	-0.728297192	0.023533888	0.000321687
ENSG00000160766.14	GBAP1	OTU_1191	0.70823704	0.036747545	0.021967335
ENSG00000160801.14	PTH1R	OTU_0067	0.738212675	0.017806036	0.004019001
ENSG00000163508.12	EOMES	OTU_0134	-0.705032237	0.038188105	0.001388436
ENSG00000164530.15	PI16	OTU_0035	-0.779186712	0.00445025	0.016613838
ENSG00000167208.15	SNX20	OTU_0134	-0.695071222	0.047086392	0.013054382
ENSG00000167895.14	TMC8	OTU_0134	-0.71201958	0.033418648	0.007240155
ENSG00000168421.13	RHOH	OTU_0134	-0.709339993	0.035449477	0.006519095
ENSG00000168874.13	ATOH8	OTU_0067	0.751808867	0.010514149	0.037619941
ENSG00000170571.12	EMB	OTU_0134	-0.72160914	0.027071714	0.018883567
ENSG00000171608.15	PIK3CD	OTU_0134	-0.729244233	0.02289151	0.000190665
ENSG00000172215.6	CXCR6	OTU_0134	-0.707291027	0.036875824	0.027086466
ENSG00000172575.12	RASGRP1	OTU_0134	-0.765178902	0.00725448	0.013636111
ENSG00000175567.9	UCP2	OTU_0134	-0.69035565	0.049922721	0.043231364
ENSG00000179921.15	GPBAR1	OTU_0002	-0.690960985	0.049479734	0.036240914
ENSG00000182866.17	LCK	OTU_0134	-0.742415366	0.015528538	0.003793415
ENSG00000198851.9	CD3E	OTU_0033	-0.804708668	0.001676015	0.000621244
ENSG00000198851.9	CD3E	OTU_0134	-0.755125621	0.009735614	0.000621244
ENSG00000211751.9	TRBC1	OTU_0134	-0.716748842	0.030405836	0.04257789
ENSG00000211772.11	TRBC2	OTU_0134	-0.737055162	0.018494952	0.027421717
ENSG00000211897.9	IGHG3	OTU_0409	-0.738918231	0.017788256	0.021486886
ENSG00000211899.10	IGHM	OTU_0134	-0.73857888	0.017604863	0.011922994
ENSG00000267121.5	AC008105.3	OTU_0134	-0.756781301	0.00944381	0.040651036

ENSG00000285094.1	LINC01488	OTU_0067	0.698976246	0.043077746	0.016465788

"Q VALUE" means adjusted P value for Pearson correlation by FDR.
"P VALUE" is evaluated by student's t test for comparing the difference of log2FC values calculated by GFOLD

supplementary Table S11 Pearson correlation-based analysis of clinical characteristics (all values) and gut microbiota (75 OTUs matched to Bacteroides & Lachnospiracea incertae sedis & Clostridium XIVa) ------- Pearson Correlation Coefficient

Clinical phenotype	BMI	ALT	AST	Total Protein	ALB	TB	DB	IB	triglyceride	total cholesterol	HDL	LDL	total serum bile acid
OTU_0002-Bacteroides	-0.02933015	0.037552338	0.28186701	0.138964945	-0.03618186	-0.005542393	-0.030305585	0.064234776	0.038632456	0.087504551	0.240099941	0.124446688	0.170334811
OTU_0003-Bacteroides	-0.05640701	0.12173089	0.00053785	0.002078178	-0.01333668	0.385136174	0.402599316	0.314766735	0.156521699	0.206787579	0.058783534	0.059076694	-0.006851706
OTU_0010-Bacteroides	-0.03881459	0.106322082	0.03425135	-0.09830779	0.026860964	-0.057442585	-0.053364079	-0.05405428	-0.03886767	0.037211988	0.008900082	0.002756309	0.089776611
OTU_0011-Bacteroides	0.07895911	0.071418083	0.04880966	0.046468908	0.046026436	0.011784523	-0.014243396	0.087375743	-0.01789363	-0.027685372	-0.02453449	0.023041496	0.178977613
OTU_0016-Bacteroides	0.085634756	0.113440992	0.06786171	0.116181343	0.125375035	-0.030685768	-0.028473052	-0.03791905	0.136408224	0.048447507	-0.11616505	0.048024476	0.145347715
OTU_0021-Bacteroides	-0.02602464	-0.05573264	-0.0732597	0.141763538	0.189203649	-0.033400752	-0.032262675	-0.02270505	0.088121866	-0.013660067	-0.07883259	0.077687335	-0.143365206
OTU_0030-Bacteroides	-0.01127128	0.051409036	0.11457263	-0.01307159	-0.11620764	-0.012827469	-0.026554511	0.030657571	-0.08196967	0.003359515	0.142835808	-0.032501384	0.147194062
OTU_0032-Bacteroides	-0.08337807	-0.00889872	0.05114891	-0.03751792	-0.02312097	0.00677104	-0.015241496	0.059031701	-0.09852008	-0.077217791	0.056264137	-0.097188339	-0.003841226
OTU_0073-Bacteroides	-0.11768655	-0.04159197	-0.00484749	-0.08530214	-0.06385296	0.165293799	0.176721445	0.122586945	0.035935031	0.006315745	-0.07564568	-0.096995323	-0.068036022
OTU_0076-Bacteroides	0.177949668	0.040654061	0.10928218	-0.04263693	-0.03319436	0.041910738	0.004268487	0.147958345	0.006573069	-0.029697531	0.129109778	-0.057461763	0.058960578
OTU_0179-Bacteroides	-0.00556259	0.034801419	0.0755574	-0.11285765	-0.1674981	-0.007191793	0.000482724	-0.01836145	-0.03189527	-0.041381295	-0.08832559	-0.025544405	0.135900063
OTU_0248-Bacteroides	0.032731782	0.188129019	0.24349276	0.072051241	0.032031094	0.035860652	0.004871265	0.121547036	-0.0134918	0.05441068	0.107057083	0.049071865	-0.084173806
OTU_0256-Bacteroides	0.072865064	0.01861638	0.02487889	-0.1011866	-0.13198504	-0.015292381	-0.014503056	-0.01155693	0.174310888	0.138445633	0.011276399	0.15974475	-0.109927964
OTU_0270-Bacteroides	-0.02376728	-0.03541381	0.01005883	0.113158916	-0.05818296	-0.004417583	0.000698841	-0.01032203	0.027184136	-0.052964844	-0.1065495	-0.075798937	0.117274353
OTU_0342-Bacteroides	-0.08607722	0.013792326	0.00169604	-0.08299867	-0.12442036	-0.028789551	-0.006330254	-0.08233617	-0.02279082	-0.003000768	-0.03004537	-0.003294112	0.160312101
OTU_0409-Bacteroides	-0.09336698	0.07325505	0.02935799	0.18810055	0.168387423	-0.004380286	-0.015928163	-0.00115352	-0.03637599	-0.019535988	0.08531464	0.039201978	0.089465919
OTU_0414-Bacteroides	0.05741649	0.102140679	0.2137498	0.022503676	-0.11780539	0.009825362	-0.020852007	0.09981558	0.031239256	0.142547505	0.134778942	0.151972784	0.106080566
OTU_0479-Bacteroides	-0.02645489	0.069400618	0.06663158	0.011305335	0.056868334	0.10481169	0.112296315	0.066766281	0.026674535	0.052801386	-0.00497914	-0.000791977	0.203706247
OTU_0511-Bacteroides	0.073899082	0.009854863	-0.09224892	-0.03599467	-0.01982861	-0.030471141	-0.029378865	-0.0268196	-0.02845278	0.088296204	0.05700164	0.109866988	-0.09673036
OTU_0552-Bacteroides	-0.11109185	0.100640382	0.11606814	-0.0103801	-0.11992152	0.000845387	-0.007466187	0.029209222	0.009770712	0.103742967	0.076502761	0.136965383	0.129778966
OTU_0575-Bacteroides	0.0384075	0.03686177	-0.00832622	0.201090962	0.156502707	-0.055026708	-0.041815141	-0.0816003	0.128477826	0.062540185	-0.0863392	0.040734855	-0.118811848
OTU_0585-Bacteroides	0.086979217	0.05589785	-0.07652276	-0.05583457	-0.02595022	0.002860694	0.006538931	-0.00285688	-0.02687618	0.106659011	0.094927688	0.096789662	-0.049129783
OTU_0611-Bacteroides	-0.06944498	0.241521339	0.18250167	-0.06449169	-0.09829838	0.856664513	0.87394901	0.73443818	0.285759393	0.378773156	-0.12330854	0.010646044	0.050630699
OTU_0690-Bacteroides	-0.09819522	0.015669211	-0.01805075	0.057621447	0.001578156	0.044355868	0.050063382	0.031859295	0.04222069	0.06797019	0.127200119	0.029152239	0.103269676
OTU_0796-Bacteroides	-0.17502916	0.144700293	0.13346869	0.135584069	0.01608137	-0.016007995	-0.024951479	0.017216592	-0.0242844	0.08647753	0.059560253	0.122854681	0.07355784
OTU_0835-Bacteroides	0.015864818	0.103170992	0.05570904	-0.11512517	-0.08233113	0.204156092	0.208096655	0.178118785	0.002060675	0.093812607	0.089180051	-0.013393034	-0.034939948
OTU_1017-Bacteroides	-0.12100804	-0.02560093	0.02314325	0.022552458	-0.00082463	-0.010480061	-0.010070509	-0.03522351	-0.19095274	-0.061394554	0.069594076	-0.050155687	-0.085206142
OTU_1097-Bacteroides	0.259438567	-0.01792744	-0.12319006	-0.08297933	-0.0652997	-0.024575152	-0.029115991	-0.00529414	-0.01461125	0.060775069	-0.04529641	0.088600055	-0.132722904
OTU_1116-Bacteroides	-0.02427068	-0.05721295	-0.03899975	0.048692034	0.111099787	-0.059671189	-0.05365119	-0.11843378	-0.01301598	0.067266938	-0.0358493	0.110075063	-0.234551474
OTU_1200-Bacteroides	-0.08220447	0.062390652	0.07049916	-0.0498944	-0.12876384	0.124959596	0.144753761	0.062685907	0.031656414	0.053319477	-0.02555794	-0.005910715	0.212484182
OTU_1295-Bacteroides	-0.10113511	0.057512438	0.08043971	0.053139297	0.066456875	0.018094798	0.01162753	0.035122479	-0.02018679	-0.032958165	-0.02270721	-0.05046253	-0.07371106

supplementary Table S11 Pearson correlation-based analysis of clinical characteristics (all values) and gut microbiota (75 OTUs matched to Bacteroides & Lachnospiracea incertae sedis & Clostridium XIVa) ------- q value

Clinical phenotype	BMI	ALT	AST	Total Protein	ALB	TB	DB	IB	triglyceride	total cholesterol	HDL	LDL	total serum bile acid
OTU_0002-Bacteroides	0.888685159	0.92277769	0.077191646	0.805941928	0.949777325	0.99290956	0.995951252	0.990325271	0.97640987	0.896827324	0.323081859	0.954185885	0.440915424
OTU_0003-Bacteroides	0.856994518	0.92277769	0.995488921	0.982571015	0.949777325	0.000391975	0.000152512	0.010615984	0.75801976	0.433706987	0.780505905	0.993357507	0.967791029
OTU_0010-Bacteroides	0.888685159	0.92277769	0.995488921	0.846131154	0.949777325	0.99290956	0.995951252	0.990325271	0.97640987	0.919254613	0.956308055	0.993357507	0.528971375
OTU_0011-Bacteroides	0.800573539	0.92277769	0.941900662	0.874276203	0.949777325	0.99290956	0.995951252	0.990325271	0.97640987	0.919254613	0.898243399	0.993357507	0.440915424
OTU_0016-Bacteroides	0.800573539	0.92277769	0.881080754	0.805941928	0.620849738	0.99290956	0.995951252	0.990325271	0.90438217	0.900989355	0.776536799	0.993357507	0.440915424
OTU_0021-Bacteroides	0.888685159	0.92277769	0.881080754	0.805941928	0.620849738	0.99290956	0.995951252	0.990325271	0.97640987	0.974835703	0.776536799	0.993357507	0.440915424
OTU_0030-Bacteroides	0.935871262	0.92277769	0.858455882	0.943545784	0.620849738	0.99290956	0.995951252	0.990325271	0.97640987	0.974835703	0.776536799	0.993357507	0.440915424
OTU_0032-Bacteroides	0.800573539	0.9254708	0.941900662	0.874276203	0.949777325	0.99290956	0.995951252	0.990325271	0.97640987	0.896827324	0.780505905	0.954185885	0.967791029
OTU_0073-Bacteroides	0.800573539	0.92277769	0.995488921	0.846380669	0.89680058	0.621429346	0.47386539	0.936770348	0.97640987	0.974835703	0.776536799	0.954185885	0.587732635
OTU_0076-Bacteroides	0.658284524	0.92277769	0.858455882	0.874276203	0.949777325	0.99290956	0.995951252	0.913173221	0.97640987	0.919254613	0.776536799	0.993357507	0.63793231
OTU_0179-Bacteroides	0.953370966	0.92277769	0.881080754	0.805941928	0.620849738	0.99290956	0.995951252	0.990325271	0.97640987	0.919254613	0.776536799	0.993357507	0.440915424
OTU_0248-Bacteroides	0.888685159	0.71288989	0.144993628	0.874276203	0.949777325	0.99290956	0.995951252	0.936770348	0.97640987	0.896827324	0.776536799	0.993357507	0.528971375
OTU_0256-Bacteroides	0.800573539	0.9254708	0.995488921	0.846131154	0.620849738	0.99290956	0.995951252	0.990325271	0.66978659	0.896827324	0.956308055	0.954185885	0.503993658
OTU_0270-Bacteroides	0.888685159	0.92277769	0.995488921	0.805941928	0.89680058	0.99290956	0.995951252	0.990325271	0.97640987	0.896827324	0.776536799	0.993357507	0.478432381
OTU_0342-Bacteroides	0.800573539	0.9254708	0.995488921	0.846380669	0.620849738	0.99290956	0.995951252	0.990325271	0.97640987	0.974835703	0.898243399	0.993357507	0.440915424
OTU_0409-Bacteroides	0.800573539	0.92277769	0.995488921	0.713409376	0.620849738	0.99290956	0.995951252	0.990325271	0.97640987	0.961315145	0.776536799	0.993357507	0.528971375
OTU_0414-Bacteroides	0.856994518	0.92277769	0.237770399	0.933417793	0.620849738	0.99290956	0.995951252	0.990325271	0.97640987	0.896827324	0.776536799	0.954185885	0.503993658
OTU_0479-Bacteroides	0.888685159	0.92277769	0.881080754	0.943545784	0.89680058	0.99290956	0.995951252	0.990325271	0.97640987	0.896827324	0.958257126	0.993357507	0.314722353
OTU_0511-Bacteroides	0.800573539	0.9254708	0.881080754	0.874276203	0.949777325	0.99290956	0.995951252	0.990325271	0.97640987	0.896827324	0.780505905	0.954185885	0.528971375
OTU_0552-Bacteroides	0.800573539	0.92277769	0.858455882	0.943545784	0.620849738	0.99290956	0.995951252	0.990325271	0.97640987	0.896827324	0.776536799	0.954185885	0.440915424
OTU_0575-Bacteroides	0.888685159	0.92277769	0.995488921	0.713409376	0.620849738	0.99290956	0.995951252	0.990325271	0.90438217	0.896827324	0.776536799	0.993357507	0.478432381
OTU_0585-Bacteroides	0.800573539	0.92277769	0.881080754	0.874276203	0.949777325	0.99290956	0.995951252	0.990325271	0.97640987	0.896827324	0.776536799	0.954185885	0.670178115
OTU_0611-Bacteroides	0.800573539	0.3088338	0.4109313	0.874276203	0.716068046	3.37137E-32	4.41946E-35	6.53117E-19	0.0667743	0.001091405	0.776536799	0.993357507	0.670178115
OTU_0690-Bacteroides	0.800573539	0.9254708	0.995488921	0.874276203	0.993083643	0.99290956	0.995951252	0.990325271	0.97640987	0.896827324	0.776536799	0.993357507	0.503993658
OTU_0796-Bacteroides	0.658284524	0.92277769	0.858455882	0.805941928	0.949777325	0.99290956	0.995951252	0.990325271	0.97640987	0.896827324	0.780505905	0.954185885	0.566733622
OTU_0835-Bacteroides	0.927373126	0.92277769	0.941900662	0.805941928	0.854665941	0.310871758	0.278816432	0.610629807	0.98271778	0.896827324	0.776536799	0.993357507	0.762515758
OTU_1017-Bacteroides	0.800573539	0.9254708	0.995488921	0.933417793	0.993083643	0.99290956	0.995951252	0.990325271	0.66290408	0.896827324	0.780505905	0.993357507	0.528971375
OTU_1097-Bacteroides	0.171185256	0.9254708	0.858455882	0.846380669	0.89680058	0.99290956	0.995951252	0.990325271	0.97640987	0.896827324	0.854239479	0.988388575	0.440915424
OTU_1116-Bacteroides	0.888685159	0.92277769	0.995488921	0.874276203	0.623604416	0.99290956	0.995951252	0.936770348	0.97640987	0.896827324	0.898243399	0.954185885	0.314722353
OTU_1200-Bacteroides	0.800573539	0.92277769	0.881080754	0.874276203	0.620849738	0.99290956	0.781743592	0.990325271	0.97640987	0.896827324	0.898243399	0.993357507	0.314722353
OTU_1295-Bacteroides	0.800573539	0.92277769	0.881080754	0.874276203	0.89680058	0.99290956	0.995951252	0.990325271	0.97640987	0.919254613	0.898243399	0.993357507	0.566733622

supplementary Table S11 Pearson correlation-based analysis of clinical characteristics (all values) and gut microbiota (75 OTUs matched to Bacteroides & Lachnospiracea incertae sedis & Clostridium XIVa) ------- Pearson Correlation Coefficient

Clinical phenotype	BMI	ALT	AST	Total Protein	ALB	TB	DB	IB	triglyceride	total cholesterol	HDL	LDL	total serum bile acid
OTU_0033-Clostridium XlVa	-0.0682852	-0.06227762	-0.121625629	0.154248295	0.171333924	-0.0350646	-0.03750008	-0.0175723	0.016252639	-0.07596394	-0.06504564	-0.062358035	0.091677706
OTU_0042-Clostridium XlVa	0.001020441	0.022562545	0.026031649	-0.03050803	-0.10596675	-0.0497571	-0.04794597	-0.0590873	-0.148062438	0.010129655	-0.0180744	0.029802363	0.123877949
OTU_0064-Clostridium XlVa	0.049638332	0.016836903	-0.013164531	0.053861673	0.000394758	-0.0144811	-0.02351849	0.01480581	0.030721832	0.020009959	0.00968123	0.015947284	0.025284449
OTU_0085-Clostridium XlVa	-0.15346941	0.072655526	0.056401722	0.044291887	-0.11039348	-0.0882071	-0.07396019	-0.1085662	-0.155066213	0.022163409	0.06238129	-0.000595358	0.16165931
OTU_0123-Clostridium XlVa	0.111991393	0.005891448	0.000581604	-0.17011933	-0.1688456	-0.0608121	-0.04959212	-0.0783218	-0.083642181	0.032983852	-0.03802919	0.074034534	-0.036348865
OTU_0356-Clostridium XlVa	0.097223894	0.229153933	0.199960864	-0.00675132	0.018143161	-0.0260536	-0.03120397	-0.0005539	-0.072520822	0.082241753	-0.03512628	0.149081691	-0.046221134
OTU_0464-Clostridium XlVa	0.088509237	-0.09538158	-0.146187295	-0.19127534	-0.09108426	-0.0598741	-0.05313363	-0.0736476	-0.091373489	-0.05399988	0.06170082	0.019358618	-0.074272829
OTU_0779-Clostridium XlVa	-0.18951485	0.075702079	0.052740292	0.064662452	-0.11138255	0.01623234	0.014012087	0.01750782	-0.189108836	-0.06425311	0.00242329	-0.061884468	0.209513453
OTU_0794-Clostridium XlVa	-0.01758769	0.051643823	0.082572373	-0.07200257	-0.13222269	-0.0282495	-0.0206906	-0.0426484	-0.102674668	-0.08575285	-0.11465501	-0.112011895	0.151657882
OTU_0884-Clostridium XlVa	-0.05535721	0.16527937	0.167163045	0.005278591	-0.01648512	0.12737879	0.120656685	0.13708663	-0.040890473	0.07531621	0.13479708	0.018798754	0.146701604
OTU_0930-Clostridium XlVa	-0.13115792	0.078989249	0.117952591	0.042254178	-0.07628886	-0.0085337	-0.01134271	0.00254939	-0.110614961	-0.08539363	-0.04143106	-0.077289393	0.123722919
OTU_1150-Clostridium XlVa	0.256307143	-0.07455728	-0.146852962	-0.11219921	-0.05237385	-0.0454087	-0.04410264	-0.0599495	-0.001889687	-0.04457399	-0.07350802	-0.067459807	-0.032107518
OTU_1158-Clostridium XlVa	0.140166184	0.083948081	0.15432131	-0.07295597	-0.35266566	0.07519828	0.011019084	0.24491779	-0.060971227	0.010861416	0.17768616	-0.057516858	0.190297425

supplementary Table S11 Pearson correlation-based analysis of clinical characteristics (all values) and gut microbiota (75 OTUs matched to Bacteroides & Lachnospiracea incertae sedis & Clostridium XIVa) ------- q value

Clinical phenotype	BMI	ALT	AST	Total Protein	ALB	TB	DB	IB	triglyceride	total cholesterol	HDL	LDL	total serum bile acid
OTU_0033-Clostridium XlVa	0.682292626	0.739955985	0.396321014	0.445648716	0.319866818	0.92851952	0.90778283	0.995353915	0.93636316	0.915197418	0.9254174	0.885790884	0.543027226
OTU_0042-Clostridium XlVa	0.991441389	0.931133006	0.926927157	0.884451382	0.490222783	0.92851952	0.90778283	0.992013217	0.509460163	0.915197418	0.979677284	0.939098774	0.355998254
OTU_0064-Clostridium XlVa	0.71096797	0.931133006	0.964092431	0.853805294	0.996689046	0.92851952	0.90778283	0.995353915	0.882437683	0.915197418	0.979677284	0.939098774	0.79036903
OTU_0085-Clostridium XlVa	0.431962669	0.72217064	0.752749977	0.853805294	0.490222783	0.92851952	0.90778283	0.992013217	0.509460163	0.915197418	0.9254174	0.995006568	0.314642425
OTU_0123-Clostridium XlVa	0.514833862	0.95061766	0.995121926	0.445648716	0.319866818	0.92851952	0.90778283	0.992013217	0.702803965	0.915197418	0.9254174	0.885790884	0.79036903
OTU_0356-Clostridium XlVa	0.567591897	0.190176932	0.318083498	0.955749144	0.934281387	0.92851952	0.90778283	0.995353915	0.723547258	0.915197418	0.9254174	0.885790884	0.79036903
OTU_0464-Clostridium XlVa	0.570723546	0.72217064	0.318083498	0.445648716	0.548148555	0.92851952	0.90778283	0.992013217	0.702803965	0.915197418	0.9254174	0.939098774	0.627340444
OTU_0779-Clostridium XlVa	0.288507668	0.72217064	0.752749977	0.853805294	0.490222783	0.92851952	0.90778283	0.995353915	0.509460163	0.915197418	0.979677284	0.885790884	0.282744164
OTU_0794-Clostridium XlVa	0.924420453	0.763053804	0.624947416	0.853805294	0.490222783	0.92851952	0.90778283	0.995353915	0.702803965	0.915197418	0.9254174	0.885790884	0.314642425
OTU_0884-Clostridium XlVa	0.71096797	0.521372646	0.318083498	0.955749144	0.934281387	0.92851952	0.90778283	0.959704655	0.867336692	0.915197418	0.9254174	0.939098774	0.314642425
OTU_0930-Clostridium XlVa	0.431962669	0.72217064	0.396321014	0.853805294	0.609422032	0.92851952	0.90778283	0.995353915	0.702803965	0.915197418	0.9254174	0.885790884	0.355998254
OTU_1150-Clostridium XlVa	0.079817389	0.72217064	0.318083498	0.769407101	0.756186347	0.92851952	0.90778283	0.992013217	0.984151608	0.915197418	0.9254174	0.885790884	0.79036903
OTU_1158-Clostridium XlVa	0.431962669	0.72217064	0.318083498	0.853805294	0.001658754	0.92851952	0.90778283	0.116161994	0.752818953	0.915197418	0.776386218	0.885790884	0.282744164

supplementary Table S11 Pearson correlation-based analysis of clinical characteristics (all values) and gut microbiota (75 OTUs matched to Bacteroides & Lachnospiracea incertae sedis & Clostridium XIVa) ------- Pearson Correlation Coefficient

Clinical phenotype	BMI	ALT	AST	Total Protein	ALB	TB	DB	IB	triglyceride	total cholesterol	HDL	LDL	total serum bile acid
OTU_0013-Lachno	-0.05302149	0.000426343	-0.001172193	0.091061362	0.079609015	-0.0121559	-0.011395478	-0.026557554	-0.045123491	-0.16697263	-0.124218965	-0.14805595	-0.049830214
OTU_0020-Lachno	-0.02874101	0.133858222	0.137883483	-0.112071075	-0.107672105	-0.022292362	-0.034892098	0.020661932	-0.061411532	0.070304256	0.067531479	0.099655486	0.168065659
OTU_0023-Lachno	0.024202195	0.190115699	0.226029575	-0.031949987	-0.252079845	0.027932751	0.004632923	0.097874144	-0.113688363	-0.014506644	0.006451638	0.041413089	0.248513952
OTU_0024-Lachno	0.225972341	-0.10201169	-0.179837427	-0.026799628	0.038760826	-0.052198344	-0.055620573	-0.030245544	-0.019827047	-0.114358156	-0.156440041	-0.060575901	-0.158065048
OTU_0086-Lachno	-0.28266195	-0.2070375	-0.182656838	0.026504707	0.120947746	-0.055275788	-0.039172283	-0.085453507	-0.077081362	-0.134651369	-0.135504714	-0.111831947	-0.104590394
OTU_0088-Lachno	-0.08798313	-0.12680356	-0.130852613	-0.161197664	-0.180459904	-0.04452468	-0.040953531	-0.046292571	-0.053672173	-0.071123357	-0.019841944	-0.106318535	0.031973973
OTU_0127-Lachno	0.163620877	-0.04463889	0.058351236	-0.192996774	-0.180231122	-0.022703267	-0.026983974	-0.003121445	0.041292691	0.00055197	0.031899737	-0.104517254	0.106153338
OTU_0129-Lachno	0.11307472	0.26909661	0.131295671	0.023528928	-0.024400526	-0.027326564	-0.039029584	0.01253662	-0.043432979	-0.055573676	0.057540046	-0.106896611	-0.042553167
OTU_0134-Lachno	-0.12293637	0.086287521	-0.034262833	0.175076706	-0.022271106	-0.088485298	-0.081335195	-0.099108704	-0.124449738	0.042588084	-0.050106819	0.134112299	0.186501047
OTU_0171-Lachno	0.048675253	0.031588829	0.047643891	0.212667417	0.162681174	-0.076184993	-0.067325967	-0.086894408	-0.15867393	-0.013773095	-0.014541071	0.054815414	0.07155267
OTU_0200-Lachno	0.11157174	0.025870057	-0.034601823	0.155853256	0.054252117	-0.065855762	-0.060126602	-0.071286724	-0.018999717	0.175043865	-0.106474997	0.251510471	-0.06576951
OTU_0377-Lachno	0.093044404	0.01224423	-0.006481198	-0.061244127	-0.025127537	-0.046402801	-0.042470535	-0.067516083	-0.06730012	-0.061310556	0.002401544	-0.118576171	-0.029527089
OTU_0403-Lachno	-0.01359712	0.129846797	0.050928996	0.096125382	0.001778718	-0.071743973	-0.055434769	-0.101192217	-0.045983433	0.076773974	-0.12839172	0.107439999	0.089354166
OTU_0436-Lachno	0.078909002	-0.04993408	-0.073514657	0.097513985	-0.057589161	-0.045690472	-0.036258561	-0.068962681	0.055889824	-0.027364289	0.069021743	-0.090197198	0.030033746
OTU_0616-Lachno	-0.01870725	-0.16124358	-0.079118553	-0.148868057	0.019426147	-0.02291816	-0.035897336	0.016640157	-0.043204015	-0.086349676	-0.005834689	-0.030135587	-0.152530035
OTU_0742-Lachno	-0.08397129	0.045902671	0.014135213	0.010234884	-0.039122371	-0.012383801	-0.017735357	-0.054499015	-0.071745222	0.006534938	0.062250032	0.0288732	-0.000352797
OTU_0776-Lachno	0.072749629	0.120529556	0.243195403	-0.093933829	-0.084131321	0.012447931	0.002406039	0.001447783	-0.10527438	-0.103467052	-0.042270357	-0.169934361	0.084530297
OTU_0790-Lachno	0.104303228	0.066765416	0.115012569	-0.113112311	-0.069763617	0.018174219	-0.014681291	0.111985228	0.056318968	-0.018354321	0.037403788	0.019950239	-0.026055692
OTU_0825-Lachno	-0.05034814	-0.09393918	-0.097623032	0.11790581	0.104203123	-0.013225571	-0.024612771	0.029433417	-0.058173353	-0.143332724	-0.04165315	-0.167032992	-0.026446388
OTU_0826-Lachno	0.096814309	0.067029091	0.09790892	-0.053542767	0.053327339	-0.04676636	-0.044500695	-0.053715377	-0.102012791	-0.033115846	0.10239553	-0.071881502	-0.00049403
OTU_0849-Lachno	-0.09330525	-0.14409321	-0.158122905	-0.034106271	0.110788989	-0.048872327	-0.044846752	-0.049674192	-0.069065582	-0.121966467	-0.097200347	-0.086739895	-0.133108694
OTU_0864-Lachno	0.319411453	-0.01328924	0.020727915	-0.137420852	-0.16020216	0.020216619	-0.005388763	0.090887493	0.035217467	0.020546986	0.033233957	0.008380968	0.085720526
OTU_0947-Lachno	0.004237718	0.191244615	0.172391993	0.014046672	-0.023048973	-0.024151022	-0.024261318	-0.024735735	-0.068474815	0.075554403	0.021346852	0.115116546	0.053695253
OTU_0957-Lachno	-0.029875	0.025011394	0.150318375	0.005385241	0.053428226	0.018712046	-0.003358968	0.080971542	-0.099059587	0.053676093	0.169452537	0.032993945	-0.01609436
OTU_1016-Lachno	-0.00370533	-0.04105581	-0.074074624	0.11163362	0.118284622	-0.025787284	-0.038660026	0.016611669	0.042083005	0.01060745	0.033323583	-0.003505554	-0.117415359
OTU_1025-Lachno	0.084017725	0.109948809	-0.018343725	0.065225674	0.010265144	-0.042658308	-0.028703459	-0.097447005	-0.107769069	-0.013449588	0.041878408	0.00244265	0.056049398
OTU_1027-Lachno	-0.10778623	-0.09220516	-0.080142353	0.136655013	0.180395167	-0.017716849	-0.019690227	-0.012618788	-0.043944098	-0.123738331	-0.083571971	-0.108439259	0.019697085
OTU_1057-Lachno	-0.02461597	0.076156013	0.102124626	-0.151574388	-0.203968343	0.006065712	-0.013512351	-0.011859628	-0.086036973	-0.005729198	0.133950515	-0.01655023	0.101287694
OTU_1099-Lachno	-0.06752309	-0.15544111	-0.071468586	0.091949801	0.117792263	0.014876085	-0.006827978	0.079676954	0.115657231	0.020915844	0.03901359	0.080179468	-0.121609285
OTU_1119-Lachno	0.273197042	-0.10004931	-0.203768458	-0.047418162	0.009016084	-0.051451506	-0.047651625	-0.049671367	-0.052897903	-0.221393108	-0.06560096	-0.115570738	-0.134016442
OTU_1149-Lachno	-0.03332525	0.037092521	-0.052888276	-0.00522526	0.02480978	-0.061139539	-0.056763977	-0.108576466	-0.025812151	0.077190795	0.054483594	0.065608116	0.02235451

supplementary Table S11 Pearson correlation-based analysis of clinical characteristics (all values) and gut microbiota (75 OTUs matched to Bacteroides & Lachnospiracea incertae sedis & Clostridium XIVa) ------- q value

Clinical phenotype	BMI	ALT	AST	Total Protein	ALB	TB	DB	IB	triglyceride	total cholesterol	HDL	LDL	total serum bile acid
OTU_0013-Lachnospiracea_in	0.857668662	0.996424137	0.990168685	0.581153806	0.830657321	0.928261489	0.979821891	0.96290602	0.762491347	0.796918625	0.914267957	0.599167334	0.925343554
OTU_0020-Lachnospiracea_in	0.917540569	0.560412819	0.471020466	0.570226612	0.648716185	0.928261489	0.979821891	0.96290602	0.762491347	0.949281064	0.914267957	0.606836303	0.662030398
OTU_0023-Lachnospiracea_in	0.917540569	0.338703499	0.24913206	0.923825816	0.219192855	0.928261489	0.979821891	0.96290602	0.762491347	0.983708528	0.979859586	0.893858696	0.246482767
OTU_0024-Lachnospiracea_in	0.124779669	0.642058967	0.350659394	0.923825816	0.927981557	0.928261489	0.979821891	0.96290602	0.841682244	0.882660112	0.914267957	0.773365154	0.662030398
OTU_0086-Lachnospiracea_in	0.035281137	0.338703499	0.350659394	0.923825816	0.648716185	0.928261489	0.979821891	0.96290602	0.762491347	0.877404121	0.914267957	0.599167334	0.738201314
OTU_0088-Lachnospiracea_in	0.729571399	0.560412819	0.471020466	0.511775892	0.347822344	0.928261489	0.979821891	0.96290602	0.762491347	0.949281064	0.958430433	0.599167334	0.925343554
OTU_0127-Lachnospiracea_in	0.516665623	0.860142404	0.796041439	0.511775892	0.347822344	0.928261489	0.979821891	0.98785743	0.762491347	0.995370475	0.914267957	0.599167334	0.738201314
OTU_0129-Lachnospiracea_in	0.729571399	0.122463455	0.471020466	0.923825816	0.927981557	0.928261489	0.979821891	0.96290602	0.762491347	0.983708528	0.914267957	0.599167334	0.925343554
OTU_0134-Lachnospiracea_in	0.729571399	0.662823428	0.856841691	0.511775892	0.927981557	0.928261489	0.979821891	0.96290602	0.762491347	0.983708528	0.914267957	0.599167334	0.662030398
OTU_0171-Lachnospiracea_in	0.857668662	0.877501076	0.796041439	0.511775892	0.398902373	0.928261489	0.979821891	0.96290602	0.762491347	0.983708528	0.972631885	0.794974322	0.874539439
OTU_0200-Lachnospiracea_in	0.729571399	0.877501076	0.856841691	0.511775892	0.890963782	0.928261489	0.979821891	0.96290602	0.841682244	0.796918625	0.914267957	0.223362203	0.891458895
OTU_0377-Lachnospiracea_in	0.729571399	0.927502124	0.977204266	0.804939235	0.927981557	0.928261489	0.979821891	0.96290602	0.762491347	0.983708528	0.979859586	0.599167334	0.925343554
OTU_0403-Lachnospiracea_in	0.947465196	0.560412819	0.796041439	0.581153806	0.985082172	0.928261489	0.979821891	0.96290602	0.762491347	0.949281064	0.914267957	0.599167334	0.771637434
OTU_0436-Lachnospiracea_in	0.740527167	0.860142404	0.700458196	0.581153806	0.890963782	0.928261489	0.979821891	0.96290602	0.762491347	0.983708528	0.914267957	0.658229196	0.925343554
OTU_0616-Lachnospiracea_in	0.934526234	0.517578669	0.700458196	0.511775892	0.927981557	0.928261489	0.979821891	0.96290602	0.762491347	0.949281064	0.979859586	0.907882887	0.662030398
OTU_0742-Lachnospiracea_in	0.729571399	0.860142404	0.942691126	0.956195781	0.927981557	0.928261489	0.979821891	0.96290602	0.762491347	0.983708528	0.914267957	0.907882887	0.997040987
OTU_0776-Lachnospiracea_in	0.764359126	0.573500468	0.24913206	0.581153806	0.830657321	0.928261489	0.979821891	0.98785743	0.762491347	0.948812824	0.914267957	0.599167334	0.771637434
OTU_0790-Lachnospiracea_in	0.729571399	0.747554051	0.581549138	0.570226612	0.890963782	0.928261489	0.979821891	0.96290602	0.762491347	0.983708528	0.914267957	0.954219373	0.925343554
OTU_0825-Lachnospiracea_in	0.857668662	0.642058967	0.627513632	0.570226612	0.648716185	0.928261489	0.979821891	0.96290602	0.762491347	0.877404121	0.914267957	0.599167334	0.925343554
OTU_0826-Lachnospiracea_in	0.729571399	0.747554051	0.627513632	0.846254556	0.890963782	0.928261489	0.979821891	0.96290602	0.762491347	0.983708528	0.914267957	0.733056813	0.997040987
OTU_0849-Lachnospiracea_in	0.729571399	0.560412819	0.417978219	0.923825816	0.648716185	0.928261489	0.979821891	0.96290602	0.762491347	0.877404121	0.914267957	0.658229196	0.708014041
OTU_0864-Lachnospiracea_in	0.017479393	0.927502124	0.937839328	0.513010137	0.398902373	0.928261489	0.979821891	0.96290602	0.787338685	0.983708528	0.914267957	0.979514925	0.771637434
OTU_0947-Lachnospiracea_in	0.968929951	0.338703499	0.350659394	0.956195781	0.927981557	0.928261489	0.979821891	0.96290602	0.762491347	0.949281064	0.958430433	0.599167334	0.925343554
OTU_0957-Lachnospiracea_in	0.917540569	0.877501076	0.434073632	0.956195781	0.890963782	0.928261489	0.979821891	0.96290602	0.762491347	0.983708528	0.914267957	0.907882887	0.925343554
OTU_1016-Lachnospiracea_in	0.968929951	0.860142404	0.700458196	0.570226612	0.648716185	0.928261489	0.979821891	0.96290602	0.762491347	0.983708528	0.914267957	0.979514925	0.738201314
OTU_1025-Lachnospiracea_in	0.729571399	0.636367611	0.937839328	0.803508764	0.955307069	0.928261489	0.979821891	0.96290602	0.762491347	0.983708528	0.914267957	0.979514925	0.925343554
OTU_1027-Lachnospiracea_in	0.729571399	0.642058967	0.700458196	0.513010137	0.347822344	0.928261489	0.979821891	0.96290602	0.762491347	0.877404121	0.914267957	0.599167334	0.925343554
OTU_1057-Lachnospiracea_in	0.917540569	0.728015423	0.627513632	0.511775892	0.347822344	0.94915889	0.979821891	0.96290602	0.762491347	0.983708528	0.914267957	0.954219373	0.738201314
OTU_1099-Lachnospiracea_in	0.778793774	0.517578669	0.700458196	0.581153806	0.648716185	0.928261489	0.979821891	0.96290602	0.762491347	0.983708528	0.914267957	0.686401895	0.738201314
OTU_1119-Lachnospiracea_in	0.035281137	0.642058967	0.31418744	0.870767238	0.955307069	0.928261489	0.979821891	0.96290602	0.762491347	0.571740975	0.914267957	0.599167334	0.708014041
OTU_1149-Lachnospiracea_in	0.917540569	0.863660288	0.796041439	0.956195781	0.927981557	0.928261489	0.979821891	0.96290602	0.840311399	0.949281064	0.914267957	0.759397691	0.925343554

supplementary Table S12 Pearson correlation-based analysis of clinical characteristics (abnormal values) and gut microbiota (75 OTUs matched to Bacteroides & Lachnospiracea incertae sedis & Clostridium XIVa) ------- Pearson Correlation Coefficient

Clinical phenotype	BMI	ALT	AST	Total Protein	ALB	TB	DB	IB	triglyceride	total cholesterol	HDL	LDL	total serum bile acid
OTU_0002-Bacteroides	-0.1665054	-0.0871442	0.195788743	-0.45075056	-0.21557246	-0.13435526	-0.05709681	-0.201900597	NA	NA	NA	NA	0.460818325
OTU_0003-Bacteroides	0.018686387	0.068876079	0.16888464	0.34236057	0.272192583	0.701204527	0.506455474	0.619621139	NA	NA	NA	NA	-0.180343425
OTU_0010-Bacteroides	0.149395367	0.114213122	-0.055419755	0.050742067	0.188159863	-0.10441549	-0.03748767	-0.171563201	NA	NA	NA	NA	0.030371512
OTU_0011-Bacteroides	-0.10380731	-0.03853541	0.095569993	0.01793675	0.310822584	-0.11649423	-0.04438491	-0.167267416	NA	NA	NA	NA	0.358916086
OTU_0016-Bacteroides	-0.14343062	-0.1331884	-0.140906139	-0.04662117	0.340397881	-0.01713829	0.005268283	-0.004033314	NA	NA	NA	NA	0.126799158
OTU_0021-Bacteroides	-0.26543316	0.000372883	0.019065598	-0.07071993	0.201079293	-0.0613353	-0.04747475	-0.081036067	NA	NA	NA	NA	0.158303888
OTU_0030-Bacteroides	0.018549562	-0.16958747	-0.132997661	0.247943378	-0.08895151	-0.08981343	-0.0537073	-0.097020925	NA	NA	NA	NA	0.41076456
OTU_0032-Bacteroides	0.271151941	-0.10227247	0.106029062	0.219723026	-0.49105792	-0.01834959	-0.00394216	0.09819525	NA	NA	NA	NA	0.034098794
OTU_0073-Bacteroides	0.149117583	0.217519472	0.247641308	0.22788647	0.185449435	0.876846842	0.590645342	0.815348675	NA	NA	NA	NA	-0.070420484
OTU_0076-Bacteroides	0.053930555	-0.0907396	0.045144036	0.335630924	-0.12052206	-0.07048658	-0.02976118	-0.108191066	NA	NA	NA	NA	0.234560731
OTU_0179-Bacteroides	0.238179067	-0.09424204	-0.06754576	-0.06679601	0.246509748	-0.05283841	-0.03192174	-0.112231645	NA	NA	NA	NA	0.141723716
OTU_0248-Bacteroides	-0.16197737	0.063413069	0.284193495	0.153419839	0.278249849	-0.05653258	-0.01078816	-0.109036697	NA	NA	NA	NA	0.190841244
OTU_0256-Bacteroides	-0.19920767	0.250672457	-0.10662952	-0.45011412	0.063376183	-0.0630292	-0.0383348	-0.108682225	NA	NA	NA	NA	-0.011748423
OTU_0270-Bacteroides	0.126852018	-0.20119895	-0.057339489	0.298261359	0.095901217	0.01457061	-0.00832891	-0.040927123	NA	NA	NA	NA	0.364072634
OTU_0342-Bacteroides	-0.00227251	-0.16408242	-0.179589377	-0.16837993	0.127640489	0.196509384	-0.00687085	0.246875481	NA	NA	NA	NA	0.19371093
OTU_0409-Bacteroides	-0.09131907	-0.02181097	-0.001923775	0.077524013	0.282450986	-0.11417473	-0.05104329	-0.11246819	NA	NA	NA	NA	0.272979258
OTU_0414-Bacteroides	-0.00950172	0.061525568	0.138003757	0.245800383	-0.09574658	-0.07570348	-0.03437566	-0.056858591	NA	NA	NA	NA	0.159549158
OTU_0479-Bacteroides	-0.04667817	0.17524334	0.0338556	0.095550993	0.412007721	0.344571717	0.118159122	0.386523334	NA	NA	NA	NA	0.272614586
OTU_0511-Bacteroides	-0.01412112	-0.11948649	0.253056013	0.067733739	0.211992517	-0.06627848	-0.03073327	-0.132987227	NA	NA	NA	NA	-0.134031735
OTU_0552-Bacteroides	-0.08161289	0.083902002	-0.030055607	-0.10379953	0.150454044	-0.03260181	-0.02905503	0.033487805	NA	NA	NA	NA	0.31535811
OTU_0575-Bacteroides	-0.20201552	-0.15845526	-0.088465211	0.030204304	0.375443666	-0.0327871	-0.03792893	-0.01080339	NA	NA	NA	NA	-0.056177182
OTU_0585-Bacteroides	-0.04881776	-0.10662066	-0.038124763	0.291123086	0.24584719	0.08875594	0.07172453	0.005985543	NA	NA	NA	NA	-0.043445736
OTU_0611-Bacteroides	0.025605388	0.208185616	0.229873685	0.237242373	0.227717201	0.956055859	0.915799335	0.897527423	NA	NA	NA	NA	-0.071384652
OTU_0690-Bacteroides	-0.08199324	-0.1567707	-0.170047012	0.335045492	0.118354392	0.120518865	0.087851207	0.072791408	NA	NA	NA	NA	0.054494434
OTU_0796-Bacteroides	-0.14336624	0.002751499	-0.014799353	0.097615985	0.253500509	-0.03138985	-0.02020141	-0.058920599	NA	NA	NA	NA	0.264486871
OTU_0835-Bacteroides	-0.0798275	-0.03779371	-0.109569585	0.235040047	0.244563802	0.706384916	0.195550711	0.656385072	NA	NA	NA	NA	-0.108070723
OTU_1017-Bacteroides	-0.04406077	-0.20502661	-0.1624584	-0.01994209	#NUM!	-0.05526636	-0.03982226	-0.05610399	NA	NA	NA	NA	-0.014762268
OTU_1097-Bacteroides	0.13422972	-0.13300883	0.053134319	-0.29113511	0.153199969	-0.05737827	-0.03901797	-0.090324726	NA	NA	NA	NA	-0.033894155
OTU_1116-Bacteroides	0.070854034	0.005087482	-0.03972367	0.11513631	0.115042643	-0.04433043	-0.05666132	#NUM!	NA	NA	NA	NA	-0.326478524
OTU_1200-Bacteroides	-0.16311159	-0.1024803	-0.097273021	0.226042892	0.15036117	0.993019689	0.134547689	0.962778808	NA	NA	NA	NA	0.19896888
OTU_1295-Bacteroides	-0.09494764	-0.05282835	0.066588628	0.02333577	0.288355846	0.01567471	0.025544138	-0.036111449	NA	NA	NA	NA	0.082216135

supplementary Table S12 Pearson correlation-based analysis of clinical characteristics (abnormal values) and gut microbiota (75 OTUs matched to Bacteroides & Lachnospiracea incertae sedis & Clostridium XIVa) ------- q value

Clinical phenotype	BMI	ALT	AST	Total Protein	ALB	TB	DB	IB	triglyceride	total cholesterol	HDL	LDL	total serum bile acid
OTU_0002-Bacteroides	0.98869093	0.90692762	0.940533549	0.551069278	0.794475176	0.936914457	0.973407811	1	NA	NA	NA	NA	0.018175894
OTU_0003-Bacteroides	0.98869093	0.90084445	0.940533549	0.673419574	0.794475176	4.84356E-05	4.28554E-05	0.010011816	NA	NA	NA	NA	0.444546091
OTU_0010-Bacteroides	0.98869093	0.9753553	0.940533549	0.93685291	0.794475176	0.936914457	0.973407811	1	NA	NA	NA	NA	0.888045107
OTU_0011-Bacteroides	0.98869093	0.90084445	0.957703075	0.93685291	0.794475176	0.936914457	0.973407811	1	NA	NA	NA	NA	0.069565278
OTU_0016-Bacteroides	0.98869093	0.90084445	0.940533549	0.93685291	0.794475176	0.936914457	0.973407811	1	NA	NA	NA	NA	0.604307934
OTU_0021-Bacteroides	0.98869093	0.91771679	0.943338569	0.93685291	0.794475176	0.936914457	0.973407811	1	NA	NA	NA	NA	0.508280669
OTU_0030-Bacteroides	0.98869093	0.90084445	0.940533549	0.673419574	0.794475176	0.936914457	0.973407811	1	NA	NA	NA	NA	0.038564531
OTU_0032-Bacteroides	0.98869093	0.90692762	0.940533549	0.673419574	0.794475176	0.936914457	0.973407811	1	NA	NA	NA	NA	0.888045107
OTU_0073-Bacteroides	0.98869093	0.44212971	0.940533549	0.673419574	0.794475176	4.84394E-10	4.70597E-07	2.21438E-05	NA	NA	NA	NA	0.835432254
OTU_0076-Bacteroides	0.98869093	0.90084445	0.940533549	0.673419574	0.794475176	0.936914457	0.973407811	1	NA	NA	NA	NA	0.291947906
OTU_0179-Bacteroides	0.98869093	0.90084445	0.940533549	0.93685291	0.794475176	0.936914457	0.973407811	1	NA	NA	NA	NA	0.576676053
OTU_0248-Bacteroides	0.98869093	0.90084445	0.940533549	0.903485181	0.794475176	0.936914457	0.973407811	1	NA	NA	NA	NA	0.418139755
OTU_0256-Bacteroides	0.98869093	0.90084445	0.940533549	0.551069278	0.842813501	0.936914457	0.973407811	1	NA	NA	NA	NA	0.934119945
OTU_0270-Bacteroides	0.98869093	0.90084445	0.943338569	0.673419574	0.794475176	0.936914457	0.973407811	1	NA	NA	NA	NA	0.069565278
OTU_0342-Bacteroides	0.98966282	0.90084445	0.940533549	0.879296653	0.794475176	0.936914457	0.973407811	1	NA	NA	NA	NA	0.418139755
OTU_0409-Bacteroides	0.98869093	0.9753553	0.940533549	0.93685291	0.794475176	0.936914457	0.973407811	1	NA	NA	NA	NA	0.195884273
OTU_0414-Bacteroides	0.98869093	0.91303273	0.940533549	0.673419574	0.794475176	0.936914457	0.973407811	1	NA	NA	NA	NA	0.508280669
OTU_0479-Bacteroides	0.98869093	0.90084445	0.940533549	0.93685291	0.794475176	0.276168773	0.973407811	0.353776617	NA	NA	NA	NA	0.195884273
OTU_0511-Bacteroides	0.98869093	0.90084445	0.940533549	0.93685291	0.794475176	0.936914457	0.973407811	1	NA	NA	NA	NA	0.591554662
OTU_0552-Bacteroides	0.98869093	0.90084445	0.940533549	0.93685291	0.794475176	0.936914457	0.973407811	1	NA	NA	NA	NA	0.117651774
OTU_0575-Bacteroides	0.98869093	0.90084445	0.940533549	0.93685291	0.794475176	0.936914457	0.973407811	1	NA	NA	NA	NA	0.869491125
OTU_0585-Bacteroides	0.98869093	0.90084445	0.940533549	0.673419574	0.794475176	0.936914457	0.973407811	1	NA	NA	NA	NA	0.888045107
OTU_0611-Bacteroides	0.98869093	0.44212971	0.940533549	0.673419574	0.794475176	2.4131E-16	9.46596E-29	1.00195E-07	NA	NA	NA	NA	0.835432254
OTU_0690-Bacteroides	0.98869093	0.90084445	0.940533549	0.673419574	0.794475176	0.936914457	0.973407811	1	NA	NA	NA	NA	0.869491125
OTU_0796-Bacteroides	0.98869093	0.90692762	0.940533549	0.93685291	0.794475176	0.936914457	0.973407811	1	NA	NA	NA	NA	0.200199488
OTU_0835-Bacteroides	0.98869093	0.9753553	0.940533549	0.673419574	0.794475176	4.84288E-05	0.736062818	0.005193506	NA	NA	NA	NA	0.690835882
OTU_1017-Bacteroides	0.98869093	0.90084445	0.940533549	0.93685291	1	0.936914457	0.973407811	1	NA	NA	NA	NA	0.934119945
OTU_1097-Bacteroides	0.98869093	0.90084445	0.99040294	0.673419574	0.794475176	0.936914457	0.973407811	1	NA	NA	NA	NA	0.888045107
OTU_1116-Bacteroides	0.98869093	0.91771679	0.940533549	0.93685291	0.794475176	0.936914457	0.973407811	1	NA	NA	NA	NA	0.11264369
OTU_1200-Bacteroides	0.98869093	0.91771679	0.940533549	0.673419574	0.794475176	6.37924E-28	0.973407811	6.4635E-12	NA	NA	NA	NA	0.418139755
OTU_1295-Bacteroides	0.98869093	0.95569311	0.957703075	0.93685291	0.794475176	0.936914457	0.973407811	1	NA	NA	NA	NA	0.830051267

supplementary Table S12 Pearson correlation-based analysis of clinical characteristics (abnormal values) and gut microbiota (75 OTUs matched to Bacteroides & Lachnospiracea incertae sedis & Clostridium XIVa) ------- Pearson Correlation Coefficient

Clinical phenotype	BMI	ALT	AST	Total Protein	ALB	TB	DB	IB	triglyceride	total cholesterol	HDL	LDL	total serum bile acid
OTU_0033-Clostridium XlVa	-0.150322305	-0.165674	-0.11823784	-0.124089926	0.20586907	-0.1207954	-0.051366668	-0.18041794	NA	NA	NA	NA	0.39286411
OTU_0042-Clostridium XlVa	-0.275731514	-0.0551722	-0.03478031	-0.24031726	0.30170601	-0.18840836	-0.058516974	-0.27598528	NA	NA	NA	NA	0.098013091
OTU_0064-Clostridium XlVa	0.240320465	-0.1427319	-0.07993714	-0.056704543	-0.3519247	-0.02706863	-0.034961658	0.081817862	NA	NA	NA	NA	0.263454265
OTU_0085-Clostridium XlVa	-0.076894031	0.1768116	0.047498212	-0.372161833	0.36291138	-0.20921017	-0.108441185	-0.31838069	NA	NA	NA	NA	0.284144652
OTU_0123-Clostridium XlVa	0.105106922	0.32792008	0.270756042	-0.061277812	0.20860173	-0.04570265	-0.064475192	-0.04866774	NA	NA	NA	NA	0.036330646
OTU_0356-Clostridium XlVa	-0.09790128	0.1750992	0.197030653	-0.523198229	0.37850186	-0.10931276	-0.057866776	-0.15864684	NA	NA	NA	NA	0.291650839
OTU_0464-Clostridium XlVa	0.143424944	-0.0288722	0.003286354	-0.332297277	0.12531332	-0.11833888	-0.062976633	-0.19104917	NA	NA	NA	NA	0.180345808
OTU_0779-Clostridium XlVa	-0.043474924	0.07990797	-0.04760851	-0.171388542	0.34530334	-0.11103049	0.003273191	-0.20875932	NA	NA	NA	NA	0.398177084
OTU_0794-Clostridium XlVa	0.352206066	0.10699885	-0.01910063	-0.107040341	0.27314758	-0.13083019	-0.03998105	-0.13558202	NA	NA	NA	NA	0.111357163
OTU_0884-Clostridium XlVa	0.22183637	0.36861237	0.074214376	0.389990275	-0.055427	0.262274276	0.132754962	0.362422507	NA	NA	NA	NA	0.22073804
OTU_0930-Clostridium XlVa	0.05295275	0.10035493	-0.01182826	-0.348276169	0.32161261	-0.07837891	-0.032947737	-0.07568775	NA	NA	NA	NA	0.177080897
OTU_1150-Clostridium XlVa	0.514104621	-0.1265514	-0.08849737	0.071639112	0.0915609	-0.05860764	-0.051882156	-0.11299292	NA	NA	NA	NA	-0.24745805
OTU_1158-Clostridium XlVa	0.340903267	0.30938953	-0.03542972	0.118841887	-0.405202	0.006879249	-0.007531146	0.150468567	NA	NA	NA	NA	0.231747383

supplementary Table S12 Pearson correlation-based analysis of clinical characteristics (abnormal values) and gut microbiota (75 OTUs matched to Bacteroides & Lachnospiracea incertae sedis & Clostridium XIVa) ------- q value

Clinical phenotype	BMI	ALT	AST	Total Protein	ALB	TB	DB	IB	triglyceride	total cholesterol	HDL	LDL	total serum bile acid
OTU_0033-Clostridium XlVa	0.668037	0.708728499	0.983730502	0.802089279	0.577609095	0.896144787	0.922399988	0.776171053	NA	NA	NA	NA	0.025772162
OTU_0042-Clostridium XlVa	0.35384973	0.807830517	0.983730502	0.609604809	0.475623465	0.896144787	0.922399988	0.776171053	NA	NA	NA	NA	0.530179917
OTU_0064-Clostridium XlVa	0.42730323	0.720895168	0.983730502	0.802089279	0.475623465	0.956676896	0.922399988	0.792372554	NA	NA	NA	NA	0.153781107
OTU_0085-Clostridium XlVa	0.78074661	0.708728499	0.983730502	0.34008422	0.475623465	0.896144787	0.922399988	0.776171053	NA	NA	NA	NA	0.13391098
OTU_0123-Clostridium XlVa	0.7485631	0.270449214	0.983730502	0.802089279	0.577609095	0.94999605	0.922399988	0.825468922	NA	NA	NA	NA	0.798182635
OTU_0356-Clostridium XlVa	0.7485631	0.708728499	0.983730502	0.162070436	0.475623465	0.896144787	0.922399988	0.776171053	NA	NA	NA	NA	0.13391098
OTU_0464-Clostridium XlVa	0.668037	0.865300988	0.983730502	0.34008422	0.760828613	0.896144787	0.922399988	0.776171053	NA	NA	NA	NA	0.271916845
OTU_0779-Clostridium XlVa	0.8041519	0.754308646	0.983730502	0.802089279	0.475623465	0.896144787	0.977919164	0.776171053	NA	NA	NA	NA	0.025772162
OTU_0794-Clostridium XlVa	0.19528123	0.720895168	0.983730502	0.802089279	0.497292062	0.896144787	0.922399988	0.776171053	NA	NA	NA	NA	0.51043279
OTU_0884-Clostridium XlVa	0.43393032	0.270449214	0.983730502	0.34008422	0.838448092	0.896144787	0.922399988	0.776171053	NA	NA	NA	NA	0.188217293
OTU_0930-Clostridium XlVa	0.8041519	0.720895168	0.983730502	0.34008422	0.475623465	0.94999605	0.922399988	0.792372554	NA	NA	NA	NA	0.271916845
OTU_1150-Clostridium XlVa	0.02056605	0.720895168	0.983730502	0.802089279	0.797260596	0.94999605	0.922399988	0.790041716	NA	NA	NA	NA	0.16671582
OTU_1158-Clostridium XlVa	0.19528123	0.270449214	0.983730502	0.802089279	0.475623465	0.970192375	0.977919164	0.776171053	NA	NA	NA	NA	0.182568786

supplementary Table S12 Pearson correlation-based analysis of clinical characteristics (abnormal values) and gut microbiota (75 OTUs matched to Bacteroides & Lachnospiracea incertae sedis & Clostridium XIVa) ------- Pearson Correlation Coefficient

Clinical phenotype	BMI	ALT	AST	Total Protein	ALB	TB	DB	IB	triglyceride	total cholesterol	HDL	LDL	total serum bile acid
OTU_0013-Lachnospiracea_incerta	-0.075555923	-0.0167698	0.02014255	-0.331514334	0.036649685	-0.137448727	-0.034815463	-0.250768488	NA	NA	NA	NA	0.019094602
OTU_0020-Lachnospiracea_incerta	0.230601066	0.04712425	-0.07026581	-0.264031522	-0.5859692	-0.048676792	-0.000866317	0.000301116	NA	NA	NA	NA	0.088951229
OTU_0023-Lachnospiracea_incerta	0.103657728	0.207486691	0.30399055	-0.361057747	0.036938461	-0.068503367	-0.020064969	-0.142782356	NA	NA	NA	NA	0.243205021
OTU_0024-Lachnospiracea_incerta	0.425066971	0.14118305	0.145451286	-0.072213444	0.124904275	-0.071302099	-0.067093196	-0.110245889	NA	NA	NA	NA	-0.201019504
OTU_0086-Lachnospiracea_incerta	-0.202314159	-0.17412697	-0.3096858	-0.610251179	0.077972528	-0.065681928	-0.031646964	-0.093548361	NA	NA	NA	NA	-0.128822764
OTU_0088-Lachnospiracea_incerta	0.26924807	0.0200978	-0.01630546	-0.4655185	0.025456451	-0.081553744	-0.045758908	-0.124534364	NA	NA	NA	NA	-0.072931175
OTU_0127-Lachnospiracea_incerta	0.034848428	-0.17000644	0.122158003	-0.465672396	-0.41431483	-0.095707754	-0.047051452	-0.143327725	NA	NA	NA	NA	0.023518671
OTU_0129-Lachnospiracea_incerta	-0.02042341	0.215747639	-0.01789319	0.318973318	-0.22760179	-0.093033884	-0.049498098	-0.155024	NA	NA	NA	NA	0.052779314
OTU_0134-Lachnospiracea_incerta	0.137708812	0.215484814	-0.07312475	0.08196101	-0.45044956	-0.154136491	-0.085971264	-0.263451583	NA	NA	NA	NA	0.443604379
OTU_0171-Lachnospiracea_incerta	0.013089078	-0.07089705	0.091661028	0.05911347	-0.15830504	-0.145922271	-0.094162513	-0.228148374	NA	NA	NA	NA	0.307843993
OTU_0200-Lachnospiracea_incerta	-0.008156061	-0.14067261	-0.0887222	-0.238098374	0.146149297	-0.077305999	-0.072144247	-0.112846419	NA	NA	NA	NA	0.023066963
OTU_0377-Lachnospiracea_incerta	-0.003402299	-0.03150055	0.131872058	0.060336806	0.209057565	-0.083601249	-0.073380933	-0.132718235	NA	NA	NA	NA	0.070642844
OTU_0403-Lachnospiracea_incerta	-0.048011962	0.400788625	0.292291877	0.253906042	0.235513733	-0.122215369	-0.05927047	-0.271620192	NA	NA	NA	NA	0.259342909
OTU_0436-Lachnospiracea_incerta	0.290753478	-0.03156538	-0.26255245	0.234744207	-0.26973693	-0.087374405	-0.059236702	-0.153763623	NA	NA	NA	NA	0.083619477
OTU_0616-Lachnospiracea_incerta	0.180901404	-0.2064524	0.108627689	-0.026703275	0.209879723	-0.108257251	-0.067219297	-0.138544509	NA	NA	NA	NA	-0.041448489
OTU_0742-Lachnospiracea_incerta	0.104038206	-0.09354675	-0.12149974	-0.007797333	0.338761457	-0.073159418	-0.040849811	-0.105259506	NA	NA	NA	NA	-0.028316603
OTU_0776-Lachnospiracea_incerta	0.140007565	-0.11356391	0.202095985	-0.134215133	0.043655266	-0.105605124	-0.018969519	-0.185639925	NA	NA	NA	NA	-0.088052553
OTU_0790-Lachnospiracea_incerta	0.31772044	0.214776075	0.168645382	-0.054426807	0.236359594	-0.085746893	-0.040154903	-0.090367599	NA	NA	NA	NA	0.040578772
OTU_0825-Lachnospiracea_incerta	0.07809783	-0.10150545	0.07125067	-0.211294781	0.185301444	-0.083831057	-0.052736011	-0.086063187	NA	NA	NA	NA	0.181448622
OTU_0826-Lachnospiracea_incerta	0.167821168	-0.08146529	0.231506581	0.182177413	0.153973223	-0.171032232	-0.070723356	-0.257094812	NA	NA	NA	NA	-0.017696893
OTU_0849-Lachnospiracea_incerta	-0.083283696	-0.29640331	-0.1147259	-0.046313991	0.147712247	-0.101451737	-0.067788065	-0.131966971	NA	NA	NA	NA	-0.104427488
OTU_0864-Lachnospiracea_incerta	0.468790507	-0.11552693	0.110664417	-0.010055619	0.076439427	-0.0647041	-0.026499989	-0.097909957	NA	NA	NA	NA	-0.03228319
OTU_0947-Lachnospiracea_incerta	0.141256054	0.065427167	-0.09974153	-0.045292557	0.285974328	-0.044628453	-0.028013545	-0.057202964	NA	NA	NA	NA	0.086756925
OTU_0957-Lachnospiracea_incerta	-0.104853657	-0.04967647	0.098262102	0.211166586	0.072483515	-0.07389714	-0.025321594	-0.122822826	NA	NA	NA	NA	0.063681263
OTU_1016-Lachnospiracea_incerta	0.35409089	-0.17594952	-0.14022757	0.112288749	0.072483515	-0.078936619	-0.066597922	-0.134750942	NA	NA	NA	NA	-0.153829273
OTU_1025-Lachnospiracea_incerta	0.093391326	0.007845796	-0.12293278	-0.116037629	0.185686314	-0.178140069	-0.040161676	-0.293073192	NA	NA	NA	NA	0.109507001
OTU_1027-Lachnospiracea_incerta	0.09631797	-0.12599356	-0.09450484	-0.201138153	0.066308049	-0.106671719	-0.04046193	-0.163924447	NA	NA	NA	NA	0.120623224
OTU_1057-Lachnospiracea_incerta	0.208675341	0.097531854	0.129272782	-0.423249564	-0.27795086	-0.053594185	-0.023624494	0.069360741	NA	NA	NA	NA	-0.046366894
OTU_1099-Lachnospiracea_incerta	0.063311132	-0.01396384	0.263782999	0.084282419	0.170486372	-0.020274001	-0.026716667	0.0038697	NA	NA	NA	NA	0.165796197
OTU_1119-Lachnospiracea_incerta	0.348410687	-0.1191926	NA	-0.048446018	0.105766197	-0.075785593	-0.047510786	-0.116800034	NA	NA	NA	NA	-0.284822506
OTU_1149-Lachnospiracea_incerta	0.35485455	0.130984353	0.002387499	-0.390917903	0.088310771	-0.132157192	-0.062521276	-0.160813781	NA	NA	NA	NA	0.014459689

supplementary Table S12 Pearson correlation-based analysis of clinical characteristics (abnormal values) and gut microbiota (75 OTUs matched to Bacteroides & Lachnospiracea incertae sedis & Clostridium XIVa) ------- q value

Clinical phenotype	BMI	ALT	AST	Total Protein	ALB	TB	DB	IB	triglyceride	total cholesterol	HDL	LDL	total serum bile acid
OTU_0013-Lachnospiracea_incertae	0.86049927	0.963241108	0.982811847	0.573209466	0.922568945	0.832369665	0.901636667	0.799342546	NA	NA	NA	NA	0.918962377
OTU_0020-Lachnospiracea_incertae	0.62899999	0.963241108	0.789832178	0.756918512	0.529046409	0.835312672	0.994155176	0.998912034	NA	NA	NA	NA	0.918962377
OTU_0023-Lachnospiracea_incertae	0.86049927	0.901848469	0.602863416	0.51026242	0.922568945	0.832369665	0.901636667	0.799342546	NA	NA	NA	NA	0.510434391
OTU_0024-Lachnospiracea_incertae	0.16918159	0.901848469	0.783367954	0.931515844	0.922568945	0.832369665	0.901636667	0.799342546	NA	NA	NA	NA	0.790569628
OTU_0086-Lachnospiracea_incertae	0.68707462	0.901848469	0.602863416	0.079399106	0.922568945	0.832369665	0.901636667	0.799342546	NA	NA	NA	NA	0.918962377
OTU_0088-Lachnospiracea_incertae	0.45646513	0.963241108	0.982811847	0.299790905	0.925442637	0.832369665	0.901636667	0.799342546	NA	NA	NA	NA	0.918962377
OTU_0127-Lachnospiracea_incertae	0.96727695	0.901848469	0.783367954	0.299790905	0.922568945	0.832369665	0.901636667	0.799342546	NA	NA	NA	NA	0.918962377
OTU_0129-Lachnospiracea_incertae	0.98452417	0.901848469	0.982811847	0.573209466	0.922568945	0.832369665	0.901636667	0.799342546	NA	NA	NA	NA	0.918962377
OTU_0134-Lachnospiracea_incertae	0.83348019	0.901848469	0.789832178	0.931515844	0.922568945	0.832369665	0.901636667	0.799342546	NA	NA	NA	NA	0.030632985
OTU_0171-Lachnospiracea_incertae	0.98452417	0.944066888	0.783367954	0.931515844	0.922568945	0.832369665	0.901636667	0.799342546	NA	NA	NA	NA	0.409330069
OTU_0200-Lachnospiracea_incertae	0.98452417	0.901848469	0.783367954	0.756918512	0.922568945	0.832369665	0.901636667	0.799342546	NA	NA	NA	NA	0.918962377
OTU_0377-Lachnospiracea_incertae	0.98452417	0.963241108	0.783367954	0.931515844	0.922568945	0.832369665	0.901636667	0.799342546	NA	NA	NA	NA	0.918962377
OTU_0403-Lachnospiracea_incertae	0.93497565	0.432795557	0.602863416	0.756918512	0.922568945	0.832369665	0.901636667	0.799342546	NA	NA	NA	NA	0.491110809
OTU_0436-Lachnospiracea_incertae	0.39934349	0.963241108	0.602863416	0.756918512	0.922568945	0.832369665	0.901636667	0.799342546	NA	NA	NA	NA	0.918962377
OTU_0616-Lachnospiracea_incertae	0.77074663	0.901848469	0.783367954	0.968589858	0.922568945	0.832369665	0.901636667	0.799342546	NA	NA	NA	NA	0.918962377
OTU_0742-Lachnospiracea_incertae	0.86049927	0.901848469	0.783367954	0.97252767	0.922568945	0.832369665	0.901636667	0.799342546	NA	NA	NA	NA	0.918962377
OTU_0776-Lachnospiracea_incertae	0.83348019	0.901848469	0.783367954	0.931515844	0.922568945	0.832369665	0.901636667	0.799342546	NA	NA	NA	NA	0.918962377
OTU_0790-Lachnospiracea_incertae	0.32497017	0.901848469	0.783367954	0.931515844	0.922568945	0.832369665	0.901636667	0.799342546	NA	NA	NA	NA	0.918962377
OTU_0825-Lachnospiracea_incertae	0.86049927	0.901848469	0.789832178	0.763452229	0.922568945	0.832369665	0.901636667	0.799342546	NA	NA	NA	NA	0.876727748
OTU_0826-Lachnospiracea_incertae	0.79938191	0.932521451	0.75063553	0.808163619	0.922568945	0.832369665	0.901636667	0.799342546	NA	NA	NA	NA	0.918962377
OTU_0849-Lachnospiracea_incertae	0.86049927	0.901848469	0.783367954	0.931515844	0.922568945	0.832369665	0.901636667	0.799342546	NA	NA	NA	NA	0.918962377
OTU_0864-Lachnospiracea_incertae	0.1396128	0.901848469	0.783367954	0.97252767	0.922568945	0.832369665	0.901636667	0.799342546	NA	NA	NA	NA	0.918962377
OTU_0947-Lachnospiracea_incertae	0.83348019	0.944066888	0.783367954	0.931515844	0.922568945	0.835312672	0.901636667	0.850301241	NA	NA	NA	NA	0.918962377
OTU_0957-Lachnospiracea_incertae	0.86049927	0.963241108	0.783367954	0.763452229	0.922568945	0.832369665	0.901636667	0.799342546	NA	NA	NA	NA	0.918962377
OTU_1016-Lachnospiracea_incertae	0.24957348	0.901848469	0.783367954	0.931515844	0.922568945	0.832369665	0.901636667	0.799342546	NA	NA	NA	NA	0.918962377
OTU_1025-Lachnospiracea_incertae	0.86049927	0.963241108	0.783367954	0.931515844	0.922568945	0.832369665	0.901636667	0.799342546	NA	NA	NA	NA	0.918962377
OTU_1027-Lachnospiracea_incertae	0.86049927	0.901848469	0.783367954	0.763452229	0.922568945	0.832369665	0.901636667	0.799342546	NA	NA	NA	NA	0.918962377
OTU_1057-Lachnospiracea_incertae	0.68707462	0.901848469	0.783367954	0.385016609	0.922568945	0.835312672	0.901636667	0.833859375	NA	NA	NA	NA	0.918962377
OTU_1099-Lachnospiracea_incertae	0.8901525	0.963241108	0.602863416	0.931515844	0.922568945	0.912302099	0.901636667	0.998912034	NA	NA	NA	NA	0.918962377
OTU_1119-Lachnospiracea_incertae	0.24957348	0.901848469	1	0.931515844	0.922568945	0.832369665	0.901636667	0.799342546	NA	NA	NA	NA	0.420580391
OTU_1149-Lachnospiracea_incertae	0.24957348	0.901848469	1	0.446610798	0.922568945	0.832369665	0.901636667	0.799342546	NA	NA	NA	NA	0.918962377

supplementary Table S13 Metabolites of gut microbes according to Virtual Metabolic Human database (http://www.vmh.life)

NCBI Taxonomy name	abbreviation	fullName	chargedFormula	keggId	casRegistry
**Clostridium (cluster) XIVa**					
Coprococcus eutactus	12dhchol	12-Dehydrocholic acid; 12-Oxodeoxycholic acid; 12oxo-3alpha,7alpha-Dihydroxy-5beta-cholan-24-oic acid	C24H37O5		
	C02528	Chenodeoxycholic acid	C24H39O4	C02528	474-25-9
	cholate	Cholic acid	C24H39O5	C00695	81-25-4
	dgchol	Chenodeoxycholic acid glycine conjugate	C26H43NO5	C05466	640-79-9
	gchola	Glycocholic acid	C26H43NO6	C01921	475-31-0
	tchola	Taurocholic acid	C26H45NO7S	C05122	81-24-3
	tdchola	Taurochenodesoxycholic acid	C26H45NO6S	C05465	516-35-8
Clostridium asparagiforme	12dhchol	12-Dehydrocholic acid; 12-Oxodeoxycholic acid; 12oxo-3alpha,7alpha-Dihydroxy-5beta-cholan-24-oic acid	C24H37O5		
	cholate	Cholic acid	C24H39O5	C00695	81-25-4
Clostridium bolteae	C02528	Chenodeoxycholic acid	C24H39O4	C02528	474-25-9
	cholate	Cholic acid	C24H39O5	C00695	81-25-4
	dgchol	Chenodeoxycholic acid glycine conjugate	C26H43NO5	C05466	640-79-9
	gchola	Glycocholic acid	C26H43NO6	C01921	475-31-0
	tchola	Taurocholic acid	C26H45NO7S	C05122	81-24-3
	tdchola	Taurochenodesoxycholic acid	C26H45NO6S	C05465	516-35-8
Clostridium citroniae	12dhchol	12-Dehydrocholic acid; 12-Oxodeoxycholic acid; 12oxo-3alpha,7alpha-Dihydroxy-5beta-cholan-24-oic acid	C24H37O5		
	cholate	Cholic acid	C24H39O5	C00695	81-25-4
Clostridium clostridioforme	C02528	Chenodeoxycholic acid	C24H39O4	C02528	474-25-9
	cholate	Cholic acid	C24H39O5	C00695	81-25-4
	dgchol	Chenodeoxycholic acid glycine conjugate	C26H43NO5	C05466	640-79-9
	gchola	Glycocholic acid	C26H43NO6	C01921	475-31-0
	tchola	Taurocholic acid	C26H45NO7S	C05122	81-24-3
	tdchola	Taurochenodesoxycholic acid	C26H45NO6S	C05465	516-35-8
Clostridium symbiosum	3dhchol	3-Dehydrocholic acid; 3oxo-7alpha,12alpha-Dihydroxy-5beta-cholan-24-oic acid	C24H37O5		
	7dhcdchol	7-Dehydrochenodeoxycholic acid; 7oxo-3alpha-hydroxy-5beta-cholan-24-oic acid	C24H37O4		
	C02528	Chenodeoxycholic acid	C24H39O4	C02528	474-25-9
	cholate	Cholic acid	C24H39O5	C00695	81-25-4
Tyzzerella nexilis	C02528	Chenodeoxycholic acid	C24H39O4	C02528	474-25-9
	cholate	Cholic acid	C24H39O5	C00695	81-25-4
	dgchol	Chenodeoxycholic acid glycine conjugate	C26H43NO5	C05466	640-79-9
	gchola	Glycocholic acid	C26H43NO6	C01921	475-31-0
	tchola	Taurocholic acid	C26H45NO7S	C05122	81-24-3
	tdchola	Taurochenodesoxycholic acid	C26H45NO6S	C05465	516-35-8
**Lachnospiracea_incertae_sedis**					

Ruminococcus torques	uchol	Ursocholic acid; 3alpha,7beta,12alpha-Trihydroxy-5beta-cholan-24-oic acid; 3alpha,7beta,12alpha-Trihydroxy-5beta-cholanic acid; 7beta-Hydroxyisocholic acid; 7-Epicholic acid	C24H39O5		
	7dhcdchol	7-Dehydrochenodeoxycholic acid; 7oxo-3alpha-hydroxy-5beta-cholan-24-oic acid	C24H37O4		
	HC02194	Ursodeoxycholic acid	C24H39O4	C07880	128-13-2
Lachnospiraceae bacterium sp. 5_1_63FAA	12dhchol	12-Dehydrocholic acid; 12-Oxodeoxycholic acid; 12oxo-3alpha,7alpha-Dihydroxy-5beta-cholan-24-oic acid	C24H37O5		
	C02528	Chenodeoxycholic acid	C24H39O4	C02528	474-25-9
	cholate	Cholic acid	C24H39O5	C00695	81-25-4
	dgchol	Chenodeoxycholic acid glycine conjugate	C26H43NO5	C05466	640-79-9
	gchola	Glycocholic acid	C26H43NO6	C01921	475-31-0
	tchola	Taurocholic acid	C26H45NO7S	C05122	81-24-3
	tdchola	Taurochenodesoxycholic acid	C26H45NO6S	C05465	516-35-8
Eubacterium rectale	C02528	Chenodeoxycholic acid	C24H39O4	C02528	474-25-9
	cholate	Cholic acid	C24H39O5	C00695	81-25-4
	dgchol	Chenodeoxycholic acid glycine conjugate	C26H43NO5	C05466	640-79-9
	gchola	Glycocholic acid	C26H43NO6	C01921	475-31-0
	tchola	Taurocholic acid	C26H45NO7S	C05122	81-24-3
	tdchola	Taurochenodesoxycholic acid	C26H45NO6S	C05465	516-35-8
Eubacterium rectale	C02528	Chenodeoxycholic acid	C24H39O4	C02528	474-25-9
	cholate	Cholic acid	C24H39O5	C00695	81-25-4
	dgchol	Chenodeoxycholic acid glycine conjugate	C26H43NO5	C05466	640-79-9
	gchola	Glycocholic acid	C26H43NO6	C01921	475-31-0
	tchola	Taurocholic acid	C26H45NO7S	C05122	81-24-3
	tdchola	Taurochenodesoxycholic acid	C26H45NO6S	C05465	516-35-8
**Bacteroides**					
Bacteroides coprocola	C02528	Chenodeoxycholic acid	C24H39O4	C02528	474-25-9
	cholate	Cholic acid	C24H39O5	C00695	81-25-4
	dgchol	Chenodeoxycholic acid glycine conjugate	C26H43NO5	C05466	640-79-9
	gchola	Glycocholic acid	C26H43NO6	C01921	475-31-0
	tchola	Taurocholic acid	C26H45NO7S	C05122	81-24-3
	tdchola	Taurochenodesoxycholic acid	C26H45NO6S	C05465	516-35-8
Bacteroides coprophilus	C02528	Chenodeoxycholic acid	C24H39O4	C02528	474-25-9
	cholate	Cholic acid	C24H39O5	C00695	81-25-4
	dgchol	Chenodeoxycholic acid glycine conjugate	C26H43NO5	C05466	640-79-9
	gchola	Glycocholic acid	C26H43NO6	C01921	475-31-0
	tchola	Taurocholic acid	C26H45NO7S	C05122	81-24-3
	tdchola	Taurochenodesoxycholic acid	C26H45NO6S	C05465	516-35-8
Bacteroides dorei	3dhchol	3-Dehydrocholic acid; 3oxo-7alpha,12alpha-Dihydroxy-5beta-cholan-24-oic acid	C24H37O5		

	C02528	Chenodeoxycholic acid	$C_{24}H_{39}O_4$	C02528	474-25-9
	cholate	Cholic acid	$C_{24}H_{39}O_5$	C00695	81-25-4
	dgchol	Chenodeoxycholic acid glycine conjugate	$C_{26}H_{43}NO_5$	C05466	640-79-9
	gchola	Glycocholic acid	$C_{26}H_{43}NO_6$	C01921	475-31-0
	icdchol	Isochenodeoxycholic acid; 3beta,7alpha-Dihydroxy-5beta-cholanic acid	$C_{24}H_{39}O_4$		
	isochol	Isochenodeoxycholic acid; 3beta,7alpha,12alpha-Trihydroxy-5beta-cholanic acid	$C_{24}H_{39}O_5$	C17660	566-24-5
	tchola	Taurocholic acid	$C_{26}H_{45}NO_7S$	C05122	81-24-3
	tdchola	Taurochenodesoxycholic acid	$C_{26}H_{45}NO_6S$	C05465	516-35-8
Bacteroides eggerthii	C02528	Chenodeoxycholic acid	$C_{24}H_{39}O_4$	C02528	474-25-9
	cholate	Cholic acid	$C_{24}H_{39}O_5$	C00695	81-25-4
	dgchol	Chenodeoxycholic acid glycine conjugate	$C_{26}H_{43}NO_5$	C05466	640-79-9
	gchola	Glycocholic acid	$C_{26}H_{43}NO_6$	C01921	475-31-0
	tchola	Taurocholic acid	$C_{26}H_{45}NO_7S$	C05122	81-24-3
	tdchola	Taurochenodesoxycholic acid	$C_{26}H_{45}NO_6S$	C05465	516-35-8
Bacteroides finegoldii	7dhcdchol	7-Dehydrochenodeoxycholic acid; 7oxo-3alpha-hydroxy-5beta-cholan-24-oic acid	$C_{24}H_{37}O_4$		
	C02528	Chenodeoxycholic acid	$C_{24}H_{39}O_4$	C02528	474-25-9
	cholate	Cholic acid	$C_{24}H_{39}O_5$	C00695	81-25-4
	dgchol	Chenodeoxycholic acid glycine conjugate	$C_{26}H_{43}NO_5$	C05466	640-79-9
	gchola	Glycocholic acid	$C_{26}H_{43}NO_6$	C01921	475-31-0
	tchola	Taurocholic acid	$C_{26}H_{45}NO_7S$	C05122	81-24-3
	tdchola	Taurochenodesoxycholic acid	$C_{26}H_{45}NO_6S$	C05465	516-35-8
Bacteroides fragilis	7dhcdchol	7-Dehydrochenodeoxycholic acid; 7oxo-3alpha-hydroxy-5beta-cholan-24-oic acid	$C_{24}H_{37}O_4$		
	C02528	Chenodeoxycholic acid	$C_{24}H_{39}O_4$	C02528	474-25-9
	cholate	Cholic acid	$C_{24}H_{39}O_5$	C00695	81-25-4
	dgchol	Chenodeoxycholic acid glycine conjugate	$C_{26}H_{43}NO_5$	C05466	640-79-9
	gchola	Glycocholic acid	$C_{26}H_{43}NO_6$	C01921	475-31-0
	tchola	Taurocholic acid	$C_{26}H_{45}NO_7S$	C05122	81-24-3
	tdchola	Taurochenodesoxycholic acid	$C_{26}H_{45}NO_6S$	C05465	516-35-8
Bacteroides intestinalis	7dhcdchol	7-Dehydrochenodeoxycholic acid; 7oxo-3alpha-hydroxy-5beta-cholan-24-oic acid	$C_{24}H_{37}O_4$		
	C02528	Chenodeoxycholic acid	$C_{24}H_{39}O_4$	C02528	474-25-9
	cholate	Cholic acid	$C_{24}H_{39}O_5$	C00695	81-25-4
	dgchol	Chenodeoxycholic acid glycine conjugate	$C_{26}H_{43}NO_5$	C05466	640-79-9
	gchola	Glycocholic acid	$C_{26}H_{43}NO_6$	C01921	475-31-0
	tchola	Taurocholic acid	$C_{26}H_{45}NO_7S$	C05122	81-24-3
	tdchola	Taurochenodesoxycholic acid	$C_{26}H_{45}NO_6S$	C05465	516-35-8
Bacteroides massiliensis	C02528	Chenodeoxycholic acid	$C_{24}H_{39}O_4$	C02528	474-25-9
	cholate	Cholic acid	$C_{24}H_{39}O_5$	C00695	81-25-4
	dgchol	Chenodeoxycholic acid glycine conjugate	$C_{26}H_{43}NO_5$	C05466	640-79-9
	gchola	Glycocholic acid	$C_{26}H_{43}NO_6$	C01921	475-31-0

	tchola	Taurocholic acid	C26H45NO7S	C05122	81-24-3
	tdchola	Taurochenodesoxycholic acid	C26H45NO6S	C05465	516-35-8
Bacteroides nordii	C02528	Chenodeoxycholic acid	C24H39O4	C02528	474-25-9
	cholate	Cholic acid	C24H39O5	C00695	81-25-4
	dgchol	Chenodeoxycholic acid glycine conjugate	C26H43NO5	C05466	640-79-9
	gchola	Glycocholic acid	C26H43NO6	C01921	475-31-0
	tchola	Taurocholic acid	C26H45NO7S	C05122	81-24-3
	tdchola	Taurochenodesoxycholic acid	C26H45NO6S	C05465	516-35-8
Bacteroides ovatus	C02528	Chenodeoxycholic acid	C24H39O4	C02528	474-25-9
	cholate	Cholic acid	C24H39O5	C00695	81-25-4
	dgchol	Chenodeoxycholic acid glycine conjugate	C26H43NO5	C05466	640-79-9
	gchola	Glycocholic acid	C26H43NO6	C01921	475-31-0
	tchola	Taurocholic acid	C26H45NO7S	C05122	81-24-3
	tdchola	Taurochenodesoxycholic acid	C26H45NO6S	C05465	516-35-8
	7dhcdchol	7-Dehydrochenodeoxycholic acid; 7oxo-3alpha-hydroxy-5beta-cholan-24-oic acid	C24H37O4		
Bacteroides pectinophilus	12dhchol	12-Dehydrocholic acid; 12-Oxodeoxycholic acid; 12oxo-3alpha,7alpha-Dihydroxy-5beta-cholan-24-oic acid	C24H37O5		
	C02528	Chenodeoxycholic acid	C24H39O4	C02528	474-25-9
	cholate	Cholic acid	C24H39O5	C00695	81-25-4
	dgchol	Chenodeoxycholic acid glycine conjugate	C26H43NO5	C05466	640-79-9
	gchola	Glycocholic acid	C26H43NO6	C01921	475-31-0
	tchola	Taurocholic acid	C26H45NO7S	C05122	81-24-3
	tdchola	Taurochenodesoxycholic acid	C26H45NO6S	C05465	516-35-8
Bacteroides plebeius	C02528	Chenodeoxycholic acid	C24H39O4	C02528	474-25-9
	cholate	Cholic acid	C24H39O5	C00695	81-25-4
	dgchol	Chenodeoxycholic acid glycine conjugate	C26H43NO5	C05466	640-79-9
	gchola	Glycocholic acid	C26H43NO6	C01921	475-31-0
	tchola	Taurocholic acid	C26H45NO7S	C05122	81-24-3
	tdchola	Taurochenodesoxycholic acid	C26H45NO6S	C05465	516-35-8
Bacteroides salyersiae	C02528	Chenodeoxycholic acid	C24H39O4	C02528	474-25-9
	cholate	Cholic acid	C24H39O5	C00695	81-25-4
	dgchol	Chenodeoxycholic acid glycine conjugate	C26H43NO5	C05466	640-79-9
	gchola	Glycocholic acid	C26H43NO6	C01921	475-31-0
	tchola	Taurocholic acid	C26H45NO7S	C05122	81-24-3
	tdchola	Taurochenodesoxycholic acid	C26H45NO6S	C05465	516-35-8
Bacteroides sp. 1_1_14	7dhcdchol	7-Dehydrochenodeoxycholic acid; 7oxo-3alpha-hydroxy-5beta-cholan-24-oic acid	C24H37O4		
	C02528	Chenodeoxycholic acid	C24H39O4	C02528	474-25-9
	cholate	Cholic acid	C24H39O5	C00695	81-25-4
	dgchol	Chenodeoxycholic acid glycine conjugate	C26H43NO5	C05466	640-79-9

	gchola	Glycocholic acid	C26H43NO6	C01921	475-31-0
	tchola	Taurocholic acid	C26H45NO7S	C05122	81-24-3
	tdchola	Taurochenodesoxycholic acid	C26H45NO6S	C05465	516-35-8
Bacteroides sp. 1_1_30	7dhcdchol	7-Dehydrochenodeoxycholic acid; 7oxo-3alpha-hydroxy-5beta-cholan-24-oic acid	C24H37O4		
	C02528	Chenodeoxycholic acid	C24H39O4	C02528	474-25-9
	cholate	Cholic acid	C24H39O5	C00695	81-25-4
	dgchol	Chenodeoxycholic acid glycine conjugate	C26H43NO5	C05466	640-79-9
	gchola	Glycocholic acid	C26H43NO6	C01921	475-31-0
	tchola	Taurocholic acid	C26H45NO7S	C05122	81-24-3
	tdchola	Taurochenodesoxycholic acid	C26H45NO6S	C05465	516-35-8
Bacteroides thetaiotaomicron	7dhcdchol	7-Dehydrochenodeoxycholic acid; 7oxo-3alpha-hydroxy-5beta-cholan-24-oic acid	C24H37O4		
	C02528	Chenodeoxycholic acid	C24H39O4	C02528	474-25-9
	cholate	Cholic acid	C24H39O5	C00695	81-25-4
	dgchol	Chenodeoxycholic acid glycine conjugate	C26H43NO5	C05466	640-79-9
	gchola	Glycocholic acid	C26H43NO6	C01921	475-31-0
	tchola	Taurocholic acid	C26H45NO7S	C05122	81-24-3
	tdchola	Taurochenodesoxycholic acid	C26H45NO6S	C05465	516-35-8
Bacteroides sp. 2_1_22	7dhcdchol	7-Dehydrochenodeoxycholic acid; 7oxo-3alpha-hydroxy-5beta-cholan-24-oic acid	C24H37O4		
	C02528	Chenodeoxycholic acid	C24H39O4	C02528	474-25-9
	cholate	Cholic acid	C24H39O5	C00695	81-25-4
	dgchol	Chenodeoxycholic acid glycine conjugate	C26H43NO5	C05466	640-79-9
	gchola	Glycocholic acid	C26H43NO6	C01921	475-31-0
	tchola	Taurocholic acid	C26H45NO7S	C05122	81-24-3
	tdchola	Taurochenodesoxycholic acid	C26H45NO6S	C05465	516-35-8
Bacteroides sp. 2_1_33B	C02528	Chenodeoxycholic acid	C24H39O4	C02528	474-25-9
	cholate	Cholic acid	C24H39O5	C00695	81-25-4
	dgchol	Chenodeoxycholic acid glycine conjugate	C26H43NO5	C05466	640-79-9
	gchola	Glycocholic acid	C26H43NO6	C01921	475-31-0
	tchola	Taurocholic acid	C26H45NO7S	C05122	81-24-3
	tdchola	Taurochenodesoxycholic acid	C26H45NO6S	C05465	516-35-8
Bacteroides sp. 2_2_4	C02528	Chenodeoxycholic acid	C24H39O4	C02528	474-25-9
	cholate	Cholic acid	C24H39O5	C00695	81-25-4
	dgchol	Chenodeoxycholic acid glycine conjugate	C26H43NO5	C05466	640-79-9
	gchola	Glycocholic acid	C26H43NO6	C01921	475-31-0
	tchola	Taurocholic acid	C26H45NO7S	C05122	81-24-3
	tdchola	Taurochenodesoxycholic acid	C26H45NO6S	C05465	516-35-8
Bacteroides sp. 3_1_19	C02528	Chenodeoxycholic acid	C24H39O4	C02528	474-25-9
	cholate	Cholic acid	C24H39O5	C00695	81-25-4

	dgchol	Chenodeoxycholic acid glycine conjugate	C26H43NO5	C05466	640-79-9
	gchola	Glycocholic acid	C26H43NO6	C01921	475-31-0
	tchola	Taurocholic acid	C26H45NO7S	C05122	81-24-3
	tdchola	Taurochenodesoxycholic acid	C26H45NO6S	C05465	516-35-8
Bacteroides sp. 3_2_5	7dhcdchol	7-Dehydrochenodeoxycholic acid; 7oxo-3alpha-hydroxy-5beta-cholan-24-oic acid	C24H37O4		
	C02528	Chenodeoxycholic acid	C24H39O4	C02528	474-25-9
	cholate	Cholic acid	C24H39O5	C00695	81-25-4
Bacteroides sp. 4_1_36	C02528	Chenodeoxycholic acid	C24H39O4	C02528	474-25-9
	cholate	Cholic acid	C24H39O5	C00695	81-25-4
	dgchol	Chenodeoxycholic acid glycine conjugate	C26H43NO5	C05466	640-79-9
	gchola	Glycocholic acid	C26H43NO6	C01921	475-31-0
	tchola	Taurocholic acid	C26H45NO7S	C05122	81-24-3
	tdchola	Taurochenodesoxycholic acid	C26H45NO6S	C05465	516-35-8
Bacteroides sp. 9_1_42FAA	3dhchol	3-Dehydrocholic acid; 3oxo-7alpha,12alpha-Dihydroxy-5beta-cholan-24-oic acid	C24H37O5		
	C02528	Chenodeoxycholic acid	C24H39O4	C02528	474-25-9
	cholate	Cholic acid	C24H39O5	C00695	81-25-4
	dgchol	Chenodeoxycholic acid glycine conjugate	C26H43NO5	C05466	640-79-9
	gchola	Glycocholic acid	C26H43NO6	C01921	475-31-0
	icdchol	Isochenodeoxycholic acid; 3beta,7alpha-Dihydroxy-5beta-cholanic acid	C24H39O4		
	isochol	Isochenodeoxycholic acid; 3beta,7alpha,12alpha-Trihydroxy-5beta-cholanic acid	C24H39O5	C17660	566-24-5
	tchola	Taurocholic acid	C26H45NO7S	C05122	81-24-3
	tdchola	Taurochenodesoxycholic acid	C26H45NO6S	C05465	516-35-8
Bacteroides sp. D1	7dhcdchol	7-Dehydrochenodeoxycholic acid; 7oxo-3alpha-hydroxy-5beta-cholan-24-oic acid	C24H37O4		
	C02528	Chenodeoxycholic acid	C24H39O4	C02528	474-25-9
	cholate	Cholic acid	C24H39O5	C00695	81-25-4
	dgchol	Chenodeoxycholic acid glycine conjugate	C26H43NO5	C05466	640-79-9
	gchola	Glycocholic acid	C26H43NO6	C01921	475-31-0
	tchola	Taurocholic acid	C26H45NO7S	C05122	81-24-3
	tdchola	Taurochenodesoxycholic acid	C26H45NO6S	C05465	516-35-8
Bacteroides sp. D2	7dhcdchol	7-Dehydrochenodeoxycholic acid; 7oxo-3alpha-hydroxy-5beta-cholan-24-oic acid	C24H37O4		
	C02528	Chenodeoxycholic acid	C24H39O4	C02528	474-25-9
	cholate	Cholic acid	C24H39O5	C00695	81-25-4
Bacteroides sp. D20	C02528	Chenodeoxycholic acid	C24H39O4	C02528	474-25-9
	cholate	Cholic acid	C24H39O5	C00695	81-25-4
	dgchol	Chenodeoxycholic acid glycine conjugate	C26H43NO5	C05466	640-79-9
	gchola	Glycocholic acid	C26H43NO6	C01921	475-31-0
	tchola	Taurocholic acid	C26H45NO7S	C05122	81-24-3
	tdchola	Taurochenodesoxycholic acid	C26H45NO6S	C05465	516-35-8

Bacteroides sp. D22	7dhcdchol	7-Dehydrochenodeoxycholic acid; 7oxo-3alpha-hydroxy-5beta-cholan-24-oic acid	C24H37O4		
	C02528	Chenodeoxycholic acid	C24H39O4	C02528	474-25-9
	cholate	Cholic acid	C24H39O5	C00695	81-25-4
	dgchol	Chenodeoxycholic acid glycine conjugate	C26H43NO5	C05466	640-79-9
	gchola	Glycocholic acid	C26H43NO6	C01921	475-31-0
	tchola	Taurocholic acid	C26H45NO7S	C05122	81-24-3
	tdchola	Taurochenodesoxycholic acid	C26H45NO6S	C05465	516-35-8
Bacteroides uniformis	C02528	Chenodeoxycholic acid	C24H39O4	C02528	474-25-9
	cholate	Cholic acid	C24H39O5	C00695	81-25-4
	dgchol	Chenodeoxycholic acid glycine conjugate	C26H43NO5	C05466	640-79-9
	gchola	Glycocholic acid	C26H43NO6	C01921	475-31-0
	tchola	Taurocholic acid	C26H45NO7S	C05122	81-24-3
	tdchola	Taurochenodesoxycholic acid	C26H45NO6S	C05465	516-35-8
Bacteroides vulgatus	C02528	Chenodeoxycholic acid	C24H39O4	C02528	474-25-9
	cholate	Cholic acid	C24H39O5	C00695	81-25-4
	dgchol	Chenodeoxycholic acid glycine conjugate	C26H43NO5	C05466	640-79-9
	gchola	Glycocholic acid	C26H43NO6	C01921	475-31-0
	tchola	Taurocholic acid	C26H45NO7S	C05122	81-24-3
	tdchola	Taurochenodesoxycholic acid	C26H45NO6S	C05465	516-35-8
Bacteroides xylanisolvens	7dhcdchol	7-Dehydrochenodeoxycholic acid; 7oxo-3alpha-hydroxy-5beta-cholan-24-oic acid	C24H37O4		
	C02528	Chenodeoxycholic acid	C24H39O4	C02528	474-25-9
	cholate	Cholic acid	C24H39O5	C00695	81-25-4
	dgchol	Chenodeoxycholic acid glycine conjugate	C26H43NO5	C05466	640-79-9
	gchola	Glycocholic acid	C26H43NO6	C01921	475-31-0
	tchola	Taurocholic acid	C26H45NO7S	C05122	81-24-3
	tdchola	Taurochenodesoxycholic acid	C26H45NO6S	C05465	516-35-8

supplementary Table S14 The relative abundance of six OUT-markers in each sample

Sample	OTU_0134	OTU_0002	OTU_0409	OTU_0033	OTU_0794	OTU_0030	Overall Survival	Disease Free Survival
zfh001	0.07736	0.14660	0.00000	0.00000	0.00000	0.00042	72	72
zfh003	0.03902	0.13064	0.00811	0.05518	0.00030	0.01085	71	71
zfh004	0.02043	0.10226	0.01649	0.00647	0.00814	0.00136	30	12
zfh005	0.01023	0.01330	0.00576	0.00806	0.00010	0.00021	71	71
zfh006	0.00000	0.16109	0.00010	0.00000	0.00000	0.01870	33	8
zfh007	0.06683	0.00250	0.00054	0.00348	0.00772	0.00152	70	70
zfh009	0.00000	0.27938	0.00000	0.00441	0.00000	0.10132	69	55
zfh010	0.03490	0.01634	0.00122	0.00396	0.00193	0.00487	9	3
zfh011	0.04395	0.00528	0.00224	0.00030	0.00041	0.00122	16	11
zfh012	0.10246	0.25505	0.03672	0.01088	0.02437	0.01946	28	22
zfh013	0.05362	0.01160	0.00255	0.00128	0.02213	0.00106	68	35
zfh014	0.00000	0.02967	0.00051	0.00122	0.00051	0.00296	62	26
zfh015	0.01049	0.00517	0.00165	0.00198	0.00033	0.00099	4	3
zfh016	0.02039	0.00447	0.00104	0.00062	0.00281	0.00094	12	8
zfh017	0.01810	0.00164	0.00087	0.00033	0.00765	0.00109	63	63
zfh018	0.00000	0.81497	0.00160	0.00190	0.00080	0.00010	63	63
zfh019	0.02590	0.06876	0.01588	0.00414	0.00604	0.00771	44	18
zfh020	0.01010	0.02662	0.01816	0.00133	0.00337	0.00898	70	70
zfh021	0.09035	0.02772	0.06657	0.01371	0.00093	0.00330	17	12
zfh022	0.06287	0.19342	0.16984	0.00374	0.00395	0.00445	27	21
zfh023	0.03875	0.01286	0.00965	0.00086	0.00322	0.00375	72	23
zfh024	0.00000	0.02680	0.00520	0.00642	0.00061	0.00041	68	53
zfh025	0.04102	0.06716	0.06674	0.00105	0.00116	0.01650	71	50
zfh026	0.06017	0.01481	0.00023	0.00128	0.00362	0.00070	70	15
zfh027	0.02581	0.00044	0.00044	0.00022	0.00022	0.00033	70	70
zfh028	0.03640	0.00408	0.00000	0.00099	0.00353	0.00099	70	70
zfh029	0.00000	0.01810	0.00000	0.00763	0.00153	0.00244	34	17
zfh030	0.03246	0.01664	0.00021	0.00021	0.00295	0.00179	67	67
zfh031	0.07013	0.05575	0.01944	0.00501	0.00092	0.00307	55	20
zfh032	0.00000	0.03698	0.00436	0.00056	0.00670	0.03709	66	34
zfh033	0.01739	0.03206	0.00615	0.00081	0.00837	0.00595	64	64
zfh034	0.01002	0.00371	0.00060	0.00010	0.00000	0.00090	63	63
zfh035	0.01034	0.05098	0.01614	0.00609	0.00075	0.02790	30	20
zfh037	0.04470	0.00631	0.00631	0.00116	0.00662	0.00431	20	8
zfh038	0.00000	0.50346	0.00100	0.00000	0.00010	0.03857	61	26
zfh041	0.01785	0.00000	0.00000	0.00499	0.00053	0.03429	64	22
zfh042	0.01018	0.01058	0.00000	0.00114	0.00010	0.05807	63	1
zfh044	0.03973	0.00736	0.00000	0.00042	0.00978	0.00021	69	69
zfh045	0.04757	0.00966	0.00021	0.00103	0.00031	0.00154	69	69
zfh046	0.05070	0.02643	0.00631	0.00054	0.00749	0.00246	64	64
zfh101	0.00000	0.01267	0.00087	0.00628	0.00249	0.00032	71	71
zfh102	0.02291	0.06264	0.00084	0.00126	0.00934	0.01291	71	71
zfh103	0.01035	0.02035	0.00803	0.00975	0.00578	0.00675	15	6
zfh104	0.03253	0.02581	0.00264	0.00857	0.01418	0.00201	69	69
zfh105	0.00000	0.00000	0.00000	0.00000	0.00000	0.00000	64	1
zfh106	0.03518	0.01968	0.01248	0.00236	0.00135	0.01608	16	6

zfh107	0.06220	0.44688	0.04443	0.00000	0.01299	0.00000	55	38
zfh108	0.00000	0.04624	0.00000	0.00337	0.00232	0.00379	50	19
zfh109	0.01012	0.00307	0.00000	0.00041	0.00000	0.00000	71	24
zfh110	0.03394	0.28449	0.01298	0.00952	0.00052	0.01989	3	3
zfh111	0.05501	0.02220	0.00229	0.00261	0.00063	0.00063	65	50
zfh112	0.00000	0.12973	0.01559	0.00000	0.00000	0.06968	63	15
zfh113	0.04325	0.10062	0.01243	0.01364	0.01155	0.00275	14	8
zfh114	0.00000	0.08898	0.00721	0.01035	0.00073	0.00533	72	71
zfh115	0.00000	0.00265	0.00000	0.00159	0.00000	0.00000	72	35
zfh116	0.04437	0.02864	0.00437	0.00055	0.02219	0.00361	26	20
zfh117	0.00000	0.05402	0.00484	0.00421	0.01127	0.00053	12	4
zfh118	0.08733	0.08677	0.03881	0.00408	0.01378	0.06284	26	18
zfh119	0.02265	0.00851	0.00031	0.00041	0.00369	0.00441	72	25
zfh120	0.01749	0.00592	0.00204	0.00173	0.00071	0.00071	72	72
zfh121	0.00000	0.11380	0.00085	0.01586	0.00000	0.01830	19	11
zfh122	0.02341	0.06431	0.00121	0.00460	0.00800	0.01041	8	4
zfh123	0.00000	0.04625	0.01230	0.01023	0.00696	0.00076	55	52
zfh124	0.01006	0.09142	0.07534	0.00233	0.00000	0.01133	64	17
zfh125	0.01011	0.18305	0.05287	0.00000	0.00010	0.01268	12	6
zfh126	0.01009	0.23133	0.00000	0.01446	0.00000	0.01844	60	8
zfh127	0.08351	0.04590	0.00000	0.00260	0.00000	0.00197	30	14
zfh128	0.00000	0.05853	0.00260	0.00156	0.00010	0.00521	61	8
zfh129	0.01443	0.08549	0.00885	0.00875	0.00208	0.03363	55	49
zfh130	0.02025	0.03750	0.02418	0.01496	0.00020	0.00389	62	5
zfh131	0.06370	0.07172	0.00476	0.00355	0.00831	0.00425	20	10
zfh132	0.03817	0.03141	0.01030	0.00926	0.00894	0.00489	15	5
zfh133	0.04859	0.00174	0.00174	0.00390	0.01723	0.00154	40	14
zfh134	0.00000	0.26402	0.00000	0.00742	0.00000	0.08186	70	3
zfh135	0.04365	0.00349	0.00339	0.00095	0.01238	0.00169	13	6
zfh136	0.00000	0.45639	0.00000	0.00000	0.00000	0.00000	69	69
zfh137	0.03188	0.10101	0.00030	0.00589	0.00122	0.02347	53	50
zfh138	0.03474	0.05681	0.03197	0.00432	0.00342	0.00915	25	10
zfh139	0.01445	0.11895	0.06031	0.01137	0.09631	0.03235	40	11
zfh140	0.00000	0.02090	0.03772	0.00000	0.00000	0.00602	35	30
zfh141	0.00000	0.05041	0.04251	0.00820	0.00000	0.00040	60	30
zfh142	0.03513	0.01514	0.00235	0.00235	0.00404	0.00112	60	29
zfh143	0.03246	0.10048	0.00969	0.00653	0.00000	0.00600	44	20
zfh144	0.07618	0.07976	0.09409	0.01859	0.00569	0.00132	69	35
zfh145	0.04725	0.07809	0.00649	0.00771	0.00071	0.01684	59	58
zfh146	0.03349	0.09277	0.03578	0.06463	0.00143	0.01672	10	6
zfh147	0.00000	0.07790	0.02892	0.00173	0.00092	0.00784	65	14
zfh148	0.05028	0.04446	0.00000	0.00192	0.00303	0.00394	29	25
zfh149	0.00000	0.21759	0.02081	0.00760	0.00073	0.01394	18	14
zfh150	0.00000	0.13622	0.06242	0.04801	0.00041	0.02010	67	67
zfh151	0.00000	0.21201	0.02214	0.00000	0.00021	0.00000	63	58
zfh152	0.03381	0.02421	0.00177	0.00821	0.00270	0.00291	54	33
zfh153	0.00000	0.03669	0.00419	0.00276	0.00000	0.00491	66	44
zfh154	0.00000	0.19096	0.01761	0.00477	0.00021	0.01813	67	28

zfh155	0.01419	0.01338	0.00483	0.02305	0.00020	0.00040	66	66
zfh156	0.02489	0.00485	0.00258	0.00320	0.00031	0.00041	55	40
zfh157	0.07324	0.00859	0.00455	0.00283	0.00768	0.00101	61	20
zfh158	0.05081	0.00806	0.01720	0.00312	0.01129	0.00054	37	7
zfh159	0.03507	0.24324	0.08371	0.00707	0.00676	0.01455	36	11
zfh160	0.02292	0.02806	0.01093	0.00179	0.00105	0.00778	5	3
zfh161	0.00000	0.03574	0.03004	0.00280	0.01678	0.00093	41	27
zfh162	0.00000	0.11305	0.00442	0.00000	0.00000	0.04053	40	20
zfh163	0.04429	0.01506	0.00784	0.00444	0.00536	0.00743	40	19
zfh164	0.00000	0.08922	0.00913	0.00507	0.00010	0.12278	65	65
zfh165	0.01012	0.03452	0.01321	0.00359	0.00020	0.00830	66	13
zfh166	0.04190	0.06368	0.00836	0.00103	0.00021	0.00403	60	20
zfh167	0.00000	0.25397	0.00070	0.03903	0.00000	0.00593	38	32
zfh168	0.04745	0.14350	0.00133	0.00828	0.01821	0.03171	18	6
zfh169	0.06273	0.37539	0.00010	0.00574	0.00081	0.05402	35	10
zfh170	0.05915	0.28971	0.00000	0.00847	0.00402	0.00445	14	9
zfh171	0.00000	0.26129	0.00041	0.00174	0.00000	0.14145	63	30
zfh172	0.02005	0.06383	0.01940	0.00030	0.00030	0.00955	63	63
zfh173	0.02282	0.14952	0.09746	0.00000	0.00042	0.00718	64	18

supplementary Table S15 The corresponding bacterial genera of six OTU-markers

OTU_0134	Bacteria;Firmicutes;Clostridia;Clostridiales;Lachnospiraceae;Lachnospiracea_incertae_sedis
OTU_0002	Bacteria;Bacteroidetes;Bacteroidia;Bacteroidales;Bacteroidaceae;Bacteroides
OTU_0409	Bacteria;Bacteroidetes;Bacteroidia;Bacteroidales;Bacteroidaceae;Bacteroides
OTU_0033	Bacteria;Firmicutes;Clostridia;Clostridiales;Lachnospiraceae;Clostridium XlVa
OTU_0794	Bacteria;Firmicutes;Clostridia;Clostridiales;Lachnospiraceae;Clostridium XlVa
OTU_0030	Bacteria;Bacteroidetes;Bacteroidia;Bacteroidales;Bacteroidaceae;Bacteroides

supplementary Table S16 Stool form scale and stool moisture in all samples

Sample	Group	Stool character	Stool colour	Stool moisture
zfh001	HCC Group	Soft	yellow	0.6946446
zfh003	HCC Group	Soft	yellow	0.7475445
zfh004	HCC Group	Soft	yellow	0.7311178
zfh005	HCC Group	Soft	yellow	0.6799038
zfh006	HCC Group	Soft	yellow	0.7159762
zfh007	HCC Group	Soft	yellow	0.7375445
zfh009	HCC Group	Soft	yellowish-brown	0.7821791
zfh010	HCC Group	Soft	yellow	0.758587
zfh011	HCC Group	Soft	yellowish-brown	0.6752635
zfh012	HCC Group	Soft	yellow	0.7526799
zfh013	HCC Group	Soft	yellow	0.8289976
zfh014	HCC Group	Soft	yellowish-brown	0.7612526
zfh015	HCC Group	Soft	yellow	0.7801048
zfh016	HCC Group	Soft	yellow	0.7582896
zfh017	HCC Group	Soft	yellow	0.7633377
zfh018	HCC Group	Soft	yellow	0.6668415
zfh019	HCC Group	Soft	yellow	0.7637377
zfh020	HCC Group	Soft	yellow	0.7414158
zfh021	HCC Group	Soft	yellow	0.6813843
zfh022	HCC Group	Soft	yellow	0.7749642
zfh023	HCC Group	Soft	yellow	0.6725645
zfh024	HCC Group	Soft	yellow	0.7502719
zfh025	HCC Group	Soft	yellow	0.8312268
zfh026	HCC Group	Soft	yellow	0.7503774
zfh027	HCC Group	Soft	yellow	0.7294178
zfh028	HCC Group	Soft	yellow	0.7307494
zfh029	HCC Group	Soft	yellow	0.8157843
zfh030	HCC Group	Soft	yellow	0.7262258
zfh031	HCC Group	Soft	yellow	0.6812538
zfh032	HCC Group	Soft	yellow	0.6630705
zfh033	HCC Group	Soft	yellow	0.7227049
zfh034	HCC Group	Soft	yellow	0.6816598
zfh035	HCC Group	Soft	yellow	0.7201554
zfh037	HCC Group	Soft	yellowish-brown	0.7163075
zfh038	HCC Group	Soft	yellow	0.8028647
zfh041	HCC Group	Soft	yellow	0.7310277
zfh042	HCC Group	Soft	yellow	0.7581673
zfh044	HCC Group	Soft	yellow	0.7645471
zfh045	HCC Group	Soft	yellow	0.7325494
zfh046	HCC Group	Soft	yellow	0.6698846
zfh101	HCC Group	Soft	yellow	0.7246681
zfh102	HCC Group	Soft	yellowish-brown	0.7179414
zfh103	HCC Group	Soft	yellow	0.7547085
zfh104	HCC Group	Soft	yellow	0.7863853
zfh105	HCC Group	Soft	yellowish-brown	0.7768535
zfh106	HCC Group	Soft	yellow	0.724567
zfh107	HCC Group	Soft	yellow	0.7796545
zfh108	HCC Group	Soft	yellow	0.7281006
zfh109	HCC Group	Soft	yellow	0.7658688
zfh110	HCC Group	Soft	yellow	0.7495561

zfh111	HCC Group	Soft	yellow	0.6960038
zfh112	HCC Group	Soft	yellow	0.795372
zfh113	HCC Group	Soft	yellow	0.7538461
zfh114	HCC Group	Soft	yellow	0.7757987
zfh115	HCC Group	Soft	yellow	0.8215142
zfh116	HCC Group	Soft	yellow	0.7662446
zfh117	HCC Group	Soft	yellow	0.7723494
zfh118	HCC Group	Soft	yellow	0.743062
zfh119	HCC Group	Soft	yellow	0.7502719
zfh120	HCC Group	Soft	yellowish-brown	0.7581508
zfh121	HCC Group	Soft	yellow	0.7481549
zfh122	HCC Group	Soft	yellow	0.7456116
zfh123	HCC Group	Soft	yellow	0.7887372
zfh124	HCC Group	Soft	yellow	0.6779167
zfh125	HCC Group	Soft	yellow	0.7464464
zfh126	HCC Group	Soft	yellow	0.8297377
zfh127	HCC Group	Soft	yellow	0.8120414
zfh128	HCC Group	Soft	yellow	0.6667818
zfh129	HCC Group	Soft	yellow	0.7647502
zfh130	HCC Group	Soft	yellow	0.7412679
zfh131	HCC Group	Soft	yellow	0.7538431
zfh132	HCC Group	Soft	yellow	0.7123464
zfh133	HCC Group	Soft	yellowish-brown	0.7100551
zfh134	HCC Group	Soft	yellowish-brown	0.752122
zfh135	HCC Group	Soft	yellow	0.7759388
zfh136	HCC Group	Soft	yellow	0.7210055
zfh137	HCC Group	Soft	yellow	0.7921305
zfh138	HCC Group	Soft	yellow	0.7226227
zfh139	HCC Group	Soft	yellow	0.7336577
zfh140	HCC Group	Soft	yellowish-brown	0.7552062
zfh141	HCC Group	Soft	yellow	0.7352538
zfh142	HCC Group	Soft	yellow	0.732717
zfh143	HCC Group	Soft	yellow	0.7327705
zfh144	HCC Group	Soft	yellow	0.7228233
zfh145	HCC Group	Soft	yellow	0.7566197
zfh146	HCC Group	Soft	yellow	0.7687987
zfh147	HCC Group	Soft	yellow	0.8120259
zfh148	HCC Group	Soft	yellow	0.8399426
zfh149	HCC Group	Soft	yellow	0.7412179
zfh150	HCC Group	Soft	yellow	0.7240275
zfh151	HCC Group	Soft	yellow	0.7205357
zfh152	HCC Group	Soft	yellow	0.7576259
zfh153	HCC Group	Soft	yellow	0.7340009
zfh154	HCC Group	Soft	yellowish-brown	0.791209
zfh155	HCC Group	Soft	yellow	0.8140898
zfh156	HCC Group	Soft	yellow	0.682577
zfh157	HCC Group	Soft	yellow	0.6801775
zfh158	HCC Group	Soft	yellow	0.8184255
zfh159	HCC Group	Soft	yellow	0.7401598
zfh160	HCC Group	Soft	yellow	0.7231693
zfh161	HCC Group	Soft	yellow	0.6583363
zfh162	HCC Group	Soft	yellow	0.7611893

zfh163	HCC Group	Soft	yellow	0.741689
zfh164	HCC Group	Soft	yellow	0.7258904
zfh165	HCC Group	Soft	yellow	0.7227612
zfh166	HCC Group	Soft	yellow	0.8112543
zfh167	HCC Group	Soft	yellow	0.8331668
zfh168	HCC Group	Soft	yellow	0.7266681
zfh169	HCC Group	Soft	yellow	0.733129
zfh170	HCC Group	Soft	yellow	0.7612275
zfh171	HCC Group	Soft	yellow	0.7264126
zfh172	HCC Group	Soft	yellow	0.7311426
zfh173	HCC Group	Soft	yellow	0.7217793
zfh201	Healthy Control	Soft	yellow	0.7779305
zfh202	Healthy Control	Soft	yellow	0.7312178
zfh203	Healthy Control	Soft	yellow	0.7346211
zfh204	Healthy Control	Soft	yellow	0.7685916
zfh205	Healthy Control	Soft	yellow	0.7214158
zfh206	Healthy Control	Soft	yellow	0.7854464
zfh207	Healthy Control	Soft	yellow	0.7828631
zfh208	Healthy Control	Soft	yellowish-brown	0.7644441
zfh209	Healthy Control	Soft	yellow	0.7313084
zfh210	Healthy Control	Soft	yellow	0.7475426
zfh211	Healthy Control	Soft	yellow	0.7351378
zfh212	Healthy Control	Soft	yellow	0.6623774
zfh213	Healthy Control	Soft	yellow	0.6908176
zfh214	Healthy Control	Soft	yellow	0.7687644
zfh215	Healthy Control	Soft	yellow	0.8075428
zfh216	Healthy Control	Soft	yellow	0.7628927
zfh217	Healthy Control	Soft	yellow	0.6892294
zfh218	Healthy Control	Soft	yellow	0.7324062
zfh219	Healthy Control	Soft	yellow	0.6564363
zfh220	Healthy Control	Soft	yellow	0.7233589
zfh221	Healthy Control	Soft	yellow	0.815717
zfh222	Healthy Control	Soft	yellow	0.8256212
zfh223	Healthy Control	Soft	yellowish-brown	0.7682362
zfh224	Healthy Control	Soft	yellow	0.7577474
zfh225	Healthy Control	Soft	yellow	0.6787747
zfh226	Healthy Control	Soft	yellow	0.8211475
zfh227	Healthy Control	Soft	yellow	0.7234277
zfh228	Healthy Control	Soft	yellowish-brown	0.7675494
zfh229	Healthy Control	Soft	yellow	0.7211794
zfh230	Healthy Control	Soft	yellow	0.7738431
zfh231	Healthy Control	Soft	yellow	0.682512
zfh232	Healthy Control	Soft	yellow	0.7770536
zfh233	Healthy Control	Soft	yellow	0.795722
zfh234	Healthy Control	Soft	yellow	0.669587
zfh235	Healthy Control	Soft	yellow	0.7738626
zfh236	Healthy Control	Soft	yellow	0.6853843
zfh237	Healthy Control	Soft	yellow	0.6772633
zfh238	Healthy Control	Soft	yellow	0.7791799
zfh239	Healthy Control	Soft	yellow	0.8162843
zfh240	Healthy Control	Soft	yellow	0.8239762
zfh241	Healthy Control	Soft	yellow	0.7628964

zfh242	Healthy Control	Soft	yellow	0.7687244
zfh243	Healthy Control	Soft	yellow	0.7046929
zfh244	Healthy Control	Soft	yellow	0.7456701
zfh245	Healthy Control	Soft	yellowish-brown	0.7364455
zfh246	Healthy Control	Soft	yellow	0.7314377
zfh247	Healthy Control	Soft	yellow	0.7889799
zfh248	Healthy Control	Soft	yellow	0.7214151
zfh249	Healthy Control	Soft	yellow	0.8180589
zfh250	Healthy Control	Soft	yellow	0.6889038
zfh251	Healthy Control	Soft	yellowish-brown	0.7207494
zfh252	Healthy Control	Soft	yellow	0.822112
zfh253	Healthy Control	Soft	yellow	0.6701458
zfh254	Healthy Control	Soft	yellow	0.8314212
zfh255	Healthy Control	Soft	yellow	0.6812167
zfh256	Healthy Control	Soft	yellow	0.732281
zfh257	Healthy Control	Soft	yellow	0.7764464
zfh258	Healthy Control	Soft	yellowish-brown	0.7181088
zfh259	Healthy Control	Soft	yellowish-brown	0.7215129
zfh260	Healthy Control	Soft	yellow	0.7525916
zfh261	Healthy Control	Soft	yellow	0.7842999
zfh262	Healthy Control	Soft	yellowish-brown	0.7785547
zfh263	Healthy Control	Soft	yellow	0.7436464
zfh264	Healthy Control	Soft	yellow	0.7422028
zfh265	Healthy Control	Soft	yellow	0.7414158
zfh266	Healthy Control	Soft	yellow	0.7250055
zfh267	Healthy Control	Soft	yellow	0.786524
zfh268	Healthy Control	Soft	yellowish-brown	0.7458127
zfh269	Healthy Control	Soft	yellow	0.8181268
zfh270	Healthy Control	Soft	yellow	0.772362
zfh271	Healthy Control	Soft	yellow	0.69296
zfh272	Healthy Control	Soft	yellow	0.8245567
zfh273	Healthy Control	Soft	yellow	0.7336116
zfh274	Healthy Control	Soft	yellow	0.7603774
zfh275	Healthy Control	Soft	yellow	0.7432719
zfh276	Healthy Control	Soft	yellow	0.7094017
zfh277	Healthy Control	Soft	yellow	0.6723071
zfh278	Healthy Control	Soft	yellow	0.7245461
zfh279	Healthy Control	Soft	yellow	0.7442554
zfh280	Healthy Control	Soft	yellowish-brown	0.7443627
zfh281	Healthy Control	Soft	yellow	0.7369287
zfh282	Healthy Control	Soft	yellow	0.7695865
zfh283	Healthy Control	Soft	yellow	0.8018927
zfh284	Healthy Control	Soft	yellow	0.6853774
zfh285	Healthy Control	Soft	yellow	0.7456116
zfh286	Healthy Control	Soft	yellow	0.7404795
zfh287	Healthy Control	Soft	yellow	0.7454455
zfh288	Healthy Control	Soft	yellow	0.7326976
zfh289	Healthy Control	Soft	yellow	0.7476211
zfh290	Healthy Control	Soft	yellow	0.7231791
zfh291	Healthy Control	Soft	yellow	0.7712436
zfh292	Healthy Control	Soft	yellow	0.7724436
zfh293	Healthy Control	Soft	yellow	0.8211317

zfh294	Healthy Control	Soft	yellow	0.7644062
zfh295	Healthy Control	Soft	yellow	0.7241088
zfh296	Healthy Control	Soft	yellow	0.7370536
zfh297	Healthy Control	Soft	yellow	0.7762294
zfh298	Healthy Control	Soft	yellow	0.7217378
zfh299	Healthy Control	Soft	yellowish-brown	0.6796004
zfh300	Healthy Control	Soft	yellow	0.7842633
