# Supplementary material for: Integrated analysis of microbiome and host transcriptome reveals correlations between gut microbiota and clinical outcomes in HBV-related hepatocellular carcinoma
Source: Genome Med. 2020 Nov 23;12:102. doi: 10.1186/s13073-020-00796-5 (PMC7682083; doi:10.1186/s13073-020-00796-5)
Supplement: Supplementary file 3 — Additional file 3. Supplementary Methods (including detailed custom code and mathematical algorithm). [file 13073_2020_796_MOESM3_ESM.pdf]

Supplementary Methods

Integrated analysis of microbiome and host transcriptome reveals correlations between gut microbiota and clinical outcomes in HBV-related hepatocellular carcinoma

Hechen Huang[1,2,3], Zhigang Ren[4,1], Xingxing Gao[1,2,3], Xiaoyi Hu[1,2,3], Yuan Zhou[1,2,3], Jianwen Jiang[1], Haifeng Lu[5], Shengyong Yin[1,2,3], Junfang Ji[6], Lin Zhou[1,2,3], Shusen Zheng[1,2,3]

[1]Division of Hepatobiliary and Pancreatic Surgery, Department of Surgery, First Affiliated Hospital, School of Medicine, Zhejiang University, Hangzhou, China

[2]NHFPC Key Laboratory of Combined Multi-organ Transplantation, Hangzhou, China

[3]Key Laboratory of Organ Transplantation, Zhejiang Province, Hangzhou, China

[4]Department of Infectious Diseases, the First Affiliated Hospital of Zhengzhou University, Zhengzhou, China

[5]State Key Laboratory for Diagnosis and Treatment of Infectious Disease;

Collaborative Innovation Center for Diagnosis and Treatment of Infectious Diseases, Zhejiang University, Hangzhou, China.

[6]MOE key Laboratory of Biosystems Homeostasis & Protection, Life Sciences Institute, Zhejiang University, Hangzhou, China

Corresponding authors: Professor Shusen Zheng and Professor Lin Zhou.

Hechen Huang, Zhigang Ren and Xingxing Gao contributed equally.

Supplementary Methods

Inclusion and exclusion criteria for faecal sample collection

A total of 113 HCC patients were diagnosed according to the international guidelines by the comprehensive integration of imaging, clinical symptoms and physical signs, laboratory tests and medical history. The diagnosis was confirmed by histopathological examination of specimens from surgical resection or percutaneous ultrasound-guided liver needle core biopsy, being the "gold standard" for HCC diagnosis. HCC patients with or without cirrhosis were screened and confirmed. The exclusion criteria were as follows: (a) intrahepatic cholangiocarcinoma; (b) prior anticancer treatment; (c) presence of other diseases such as hypertension, diabetes and metabolic disease; and (d) participants missing clinical information or clinical outcome data. Tumor differentiation was graded following Edmondson [1] and liver function was assessed following the Child-Pugh classification.

The control group consisted of 100 healthy volunteers who visited our hospital for their annual physical examination. The inclusion criteria for healthy volunteers referred to our previous study [2]. In all healthy controls, physical examination, liver biochemistry,

routine examination of blood, urine and stool, serological tests (including the detection of hepatitis B surface antigen, hepatitis C virus antibody, Treponema pallidum antibody and human immunodeficiency virus antibody), liver function, renal function, electrolyte, liver ultrasound, electrocardiogram and chest X-ray results were in the normal range. The exclusion criteria for healthy volunteers included hypertension, diabetes, obesity, metabolic syndrome, irritable bowel syndrome (IBD), nonalcoholic fatty liver disease, coeliac disease and liver cirrhosis. Individuals who received antibiotics and/or probiotics within 8 weeks before enrollment were also excluded. Individuals whose health status could not be confirmed in September 2019 were also excluded.

The participants' demographics, clinicopathological data, CT/MRI/Ultrasound scans, histopathology data and clinical treatment information were collected from hospital electronic medical records and questionnaires. Clinical images including computed tomography (CT) scans and enhanced CT were collected for healthy controls and HCC patients.

Inclusion and exclusion criteria for liver tissues collection

Among the 113 HCC patients, paired tumor and adjacent non-tumor liver tissues from the 32 HCC patients were finally collected for NGS RNA-seq based on stringent criteria. The exclusion criteria were as follows: (a) more than seven days from collection of feces to liver cancer resection; (b) the use of drugs that affect liver metabolism and function before resection, such as adenosyl methionine, ursodeoxycholic acid, polyene phosphatidylcholine, glutathione, fructose diphosphate, vitamins and Chinese materia medica; (c) for multiple tumors of HCC, a variety of types of tissue differentiation in pathological diagnosis; and (d) unqualified RNA from tumor or adjacent non-tumor specimens (detected by agarose gel electrophoresis and Agilent 2100).

Faecal sample collection, DNA extraction and stool moisture measurement

Each individual provided a fresh stool sample at 6:30-8:30 am; this was delivered immediately from our hospital to the laboratory in an ice bag using insulating polystyrene foam containers. In the laboratory, the sample was divided into five aliquots of 200 mg and immediately stored at -80 °C. Any samples that stayed at room

temperature for more than 2 hours were discarded. A frozen aliquot (200 mg) of each fecal sample was processed by phenol trichloro methane DNA extraction using a bead beater to mechanically disrupt cells, followed by phenol-chloroform extraction, as we previously described [3,4]. DNA was further purified using the Quick gel extraction kit (Qiagen, Germany) according to the manufacturer's instructions. DNA concentration was measured by a NanoDrop (Thermo Scientific, USA), and its molecular size was estimated by agarose gel electrophoresis. Stool consistency was assessed using routine stool testing results. Stool moisture content was determined in duplicate on the frozen homogenized faecal material (−80°C) as the percentage of stool mass loss after lyophilization (Labconco FreeZone, LABCONCO, USA).

Gut microbiota richness, composition and microbial markers are strongly associated with stool consistency and moisture [5]. The stool characteristics of in all 213 samples were soft and most of the samples presented yellow or yellow-brown in stool color (Additional file 1: Fig. S11). There were no significant differences in terms of the stool moisture between groups (Additional file 1: Fig. S12, Additional file 2: Table S16).

DNA libraries construction and 16S Miseq rRNA sequencing

The extracted DNA samples were amplified with a set of primers targeting the hypervariable V3-V5 region (338F/806R) of 16S rRNA gene. The forward primer was 5'-ACTCCTACGGGAGGCAGCA-3' and the reverse primer was 3'-GGACTACHVGGGTWTCTAAT-5'. Barcode and adapter were incorporated between the adapter and the forward primers. The PCR was conducted in a PCR machine (ABI GeneAmp® 9700) under the following conditions: 95°C for 2 min; 30 cycles of 95°C for 30 s, 55°C for 30 s, 72°C for 30 s, and completed with a final extension at 72°C for 5 min. PCR products were detected on a 2 % (w/v) agarose gel, and the band was extracted and purified using the AxyPrepDNA Gel (Axygen, USA) and PCR Clean-up System. The purified PCR product for each sample was mixed. DNA libraries were constructed according to the manufacturer's instructions, and the sequencing was performed on the Illumina MiSeq platform by Shanghai Itechgene Technology Co. Ltd., China. The raw Illumina read data for all samples have been deposited in the European Bioinformatics Institute European Nucleotide Archive database under the accession number PRJEB8708. According to the specific barcodes, the filtered reads were

assigned into different samples, and then the barcodes and primers were trimmed off. The amplified reads were processed with following steps: (a) pair end sequenced reads of each library were overlapped by FLASH version 1.2.10 [6] with default parameters. (b) a custom per program was used to perform more specific quality control of overlapped reads generated by FLASH: 1) No ambiguous bases (N) were allowed in reads; 2) No more than 5 mismatches were allowed in overlap region; 3) No mismatches were allowed in barcode/primer region. (c) reads were de-multiplexed and assigned into different samples according to barcodes; (d) chimeric sequences were detected and removed with UCHIME version 4.2.40 [7] with 16S "golden standard" database provided by Broad Institute as reference (version microbiome util-r20110519, http://drive5.com/uchime/gold.fa) to match Operational Taxonomy Units (OTUs).

Operational Taxonomy Units (OTUs) taxonomy annotation and clustering

We randomly chose reads from all samples with equal number, and then OTUs were binned by UPARSE pipeline [8] with following steps: (a) abundant sequences and singletons were firstly removed; (b) unique sequences were binned into OTUs with

command “usearch-cluster_otus”; (c) randomly selected sequences were aligned against OTU sequences with command “usearch-usearch_global-id 0.97”, the identity threshold was set as 0.97, and then OTU composition table was created.

Bacterial diversity and taxonomic analysis

The Bray-Curtis distances between Non-Small HCC and Small HCC were calculated using Manhattan method in metaMDSdist() function from R “vegan” package and the NMDS analysis was assessed using metaMDS() with k = 2 [9]. Faecal microbial characterization was analyzed by linear discriminant analysis (LDA) effect size (LEfSe) method (http://huttenhower.sph.harvard.edu/galaxy) [10]. Based on the normalized relative abundance matrix, features with significantly different abundances between assigned taxa were determined by LEfSe with factorial Kruskal-Wallis test ($p<0.05$) and LDA was used to assess the effect size of each feature ($\log_{10}$(LDA score) = 3 as cut-off value).

RNA Extraction and RNA-seq

Liver tumor tissues and adjacent tissues (approximately 0.5*0.5*0.5 cm) were placed in a sterile, empty freezer vial and snap frozen in liquid nitrogen immediately after resection. After 3 hours, these tissues were moved to a -80-degree freezer for long-term storage. At the same time, liver tumor tissues and adjacent tissues (approximately 0.5*0.5*0.5 cm) from the same patient were subjected to formalin fixation immediately, and after one week, immunohistochemical analysis was performed.

In January 2019, total RNA was extracted from the frozen paired tumor and adjacent tissues of 32 patients in one batch. Since we collected these tissues from November 2013 to July 2014, they were stored in a -80-degree refrigerator for approximately 5 years (during which there was no repeated freezing, thawing or cutting). Total RNA was extracted and purified from frozen tissues using TRIzol reagent (Invitrogen, USA). RNA integrity was measured on an Agilent 2100 Bioanalyzer (Agilent Technologies, USA). Paired samples with high RNA integrity (RNA integrity number > 6), no contaminants and enough RNA were used to prepare the transcriptome libraries. RNA was isolated using Sera-Mag oligo (dT) beads (Thermo Scientific, USA) and fragmented with the NEB Fragmentation Reagents kit (New England Biolabs, USA).

The cDNA synthesis, end-repair, A-base addition, and ligation of the Illumina index adapters were performed according to Illumina's TruSeq RNA protocol (Illumina, USA). Library quality was measured on an Agilent 2100 Bioanalyzer for product size and concentration. Paired-end libraries were sequenced by an Illumina HiSeq 2500 (pair-end 150-nucleotide read length). For paired tumor and adjacent non-tumor liver tissues samples from 32 patients, RNA-seq resulted in an average of 47.92 M and 45.53 M high-quality reads, respectively (Additional file 1: Fig. S2A). Clean Data were deposited in NCBI Gene Expression Omnibus (accession number: GSE138485/PRJNA576155).

RNA-seq data analysis with edgeR and GFOLD

After removal of adaptor contamination, polyA and polyC, sequencing reads were aligned using HISAT2 (version 2.1.0) to human reference sequence (UCSC hg38.p12 assembly) [11]. featureCounts (release 1.6.3) was performed for each gene count from trimmed reads against the GENCODE (release 30) (https://www.gencodegenes.org) [12]. For data from paired liver tissues samples of 32 patients, gene expression levels were

quantitated by edgeR (version 3.26.0), and biological coefficient of variation (BCV) was 0.6114732 (Additional file 1: Fig. S2B) [13]. The input file contained 64 samples as columns and 58870 genes (22 +X +Y + mitochondrion) as rows. Differential gene was defined as its adjusted p value <0.05 and $|log_2FC| > 0.8$. As to single paired tumor and adjacent non-tumor liver tissues samples from each patient, gene expression values were calculated by GFOLD (V1.1.4) [14]. GFOLD is especially useful when no replicate is available. GFOLD generalizes the fold change by considering the posterior distribution of log fold change, such that each gene is assigned a reliable fold change. The input file contained 2 samples as columns and 58833 genes (22 +X +Y) as rows. Differential expressed gene was defined as absolute values of "gfold" >0.8 and $|log_2FC| > 0.8$. When calculating the correlation between differential gene expression and OTU abundance, the $|log_2FC|$ values of these genes that did not satisfy the above conditions were forcibly defined as zero.

Immunohistochemistry

Tumor and adjacent non-tumor liver tissues were made into 3 μm paraffin sections and

pretreated, followed by deparaffinization. After antigen retrieval, primary antibodies were applied (CD6, 1:100, abcam, ab109217; MAPK10/JNK3, 1:100, abcam, ab236096; ABCC4/MRP4, 1:100, abcam, ab233382) overnight at 4°C. Slides were incubated for 30 mins at 37°C with secondary antibody (ZB-2301, ZSGB-BIO). HRP activity was detected using DAB+ Substrate Chromogen System (ZLI-9018, ZSGB-BIO). The sections were photographed by microscopy (Zeiss, Germany).

Clinic outcomes and pair-wise gene expression correlation analysis on GEPIA

GEPIA (containing 369 hepatocellular carcinoma samples and 50 normal liver tissues, 36 cholangial carcinoma samples and 9 normal cholangial tissues, 179 pancreatic adenocarcinoma samples and 4 normal pancreatic tissues, 275 colon adenocarcinoma samples and 41 normal colon tissues, 92 rectum adenocarcinoma samples and 10 normal rectum tissues) based on TCGA was used as an independent diagnostic tool to compensate for the bias caused by small samples in this study [15]. Overall survival and disease free survival were identified by Log-rank test, a.k.a the Mantel-Cox test (Cutoff-High and Cutoff-Low is both 50%). Pair-wise gene expression correlation

analysis using Pearson method was performed to describe gene co-expression in 369 hepatocellular carcinoma samples, which was visualized by Heml (1.0.3.7) [16].

Pathway Enrichment Analysis

Pathway enrichment analysis (GO & KEGG) of OTU-related genes was performed using DAVID 6.8 and Metascape (http://metascape.org) [17,18]. Pathways with a p value threshold of 0.05 were regarded to be significantly regulated. Pathway enrichments analyzed by Metascape were imported into Cytoscape (v 3.7.2) to create functional genes enrichment network [19].

REFERENCES

[1] Wittekind C. [Pitfalls in the classification of liver tumors]. Pathologe 2006; 27: 289-93.

[2] Qin N, Yang F, Li A, Prifti E, Chen Y, Shao L, et al. Alterations of the human gut microbiome in liver cirrhosis. Nature 2014; 513: 59-64.

[3] Ren Z, Jiang J, Lu H, Chen X, He Y, Zhang H, et al. Intestinal microbial variation may

predict early acute rejection after liver transplantation in rats. Transplantation 2014; 98: 844-52.

[4] Chen Y, Yang F, Lu H, Wang B, Lei D, Wang Y, et al. Characterization of fecal microbial communities in patients with liver cirrhosis. Hepatology 2011; 54: 562-72.

[5] Vandeputte D, Falony G, Vieira-Silva S, et al. Stool consistency is strongly associated with gut microbiota richness and composition, enterotypes and bacterial growth rates. Gut 2016; 65:57-62.

[6] Magoc T, Salzberg SL. FLASH: fast length adjustment of short reads to improve genome assemblies. Bioinformatics 2011; 27: 2957-63.

[7] Edgar RC, Haas BJ, Clemente JC, Quince C, Knight R. UCHIME improves sensitivity and speed of chimera detection. Bioinformatics 2011; 27: 2194-200.

[8] Edgar RC. UPARSE: highly accurate OTU sequences from microbial amplicon reads. Nat Methods 2013; 10: 996-8.

[9] Oksanen J, Blanchet Fg, Kindt r, et al. Ordination methods, diversity analysis and other functions for community and vegetation ecologists. 05-26 edn. vegan: community ecology Package, 2015.

[10] Segata N, Izard J, Waldron L, et al. Metagenomic biomarker discovery and explanation. Genome Biol 2011; 12: R60.

[11] Kim D, Langmead B, Salzberg SL. HISAT: a fast spliced aligner with low memory requirements. Nat Methods 2015; 12: 357-60.

[12] Liao Y, Smyth GK and Shi W. featureCounts: an efficient general-purpose program for assigning sequence reads to genomic features. Bioinformatics 2014; 30: 923-30.

[13] Robinson MD, McCarthy DJ, Smyth GK. edgeR: a Bioconductor package for differential expression analysis of digital gene expression data. Bioinformatics 2010; 26: 139-40.

[14] Feng J, Meyer CA, Wang Q, et al. GFOLD: a generalized fold change for ranking differentially expressed genes from RNA-seq data. Bioinformatics 2012; 28: 2782-8.

[15] Tang Z, Li C, Kang B, et al. GEPIA: a web server for cancer and normal gene expression profiling and interactive analyses. Nucleic Acids Res 2017; 45: W98-W102.

[16] Deng W, Wang Y, Liu Z, et al. Heml: a toolkit for illustrating heatmaps. PLoS One 2014; 9: e111988.

[17] Huang da W, Sherman BT, Lempicki RA. Systematic and integrative analysis of large gene lists using DAVID bioinformatics resources. Nat Protoc 2009; 4: 44-57.

[18] Zhou Y, Zhou B, Pache L, et al. Metascape provides a biologist-oriented resource for the analysis of systems-level datasets. Nat Commun 2019; 10: 1523.

[19] Shannon P, Markiel A, Ozier O, et al. Cytoscape: a software environment for integrated models of biomolecular interaction networks. Genome Res. 2003; 13: 2498-504.

The detailed scripts of Pearson correlation calculations and predicted model constructions for clinical prognosis (python 3.7.0)

```
import numpy as np

import pandas as pd

from scipy.stats.stats import pearsonr

from statsmodels.stats.multitest import fdrcorrection


#calculate correlation of gene and OTU

gene_num=5874

otu_num=310

pvalue=[]

coef=np.zeros((gene_num,otu_num))

p_value=np.zeros((gene_num,otu_num))

for i in range(1, gene_num):

    for j in range(1,otu_num):
```

```
        row_gene =

        row_otu =

        list_gene = pd.Series(row_gene)

        list_otu = pd.Series(row_otu)

        r_value,p = pearsonr(list_gene,list_otu)

        pvalue.append(p)

        p_value[i,j]=p

        coef[i,j] = r_value    # value of correlation


# false discovery rate

t,q=fdrcorrection(pvalue,alpha=0.05,method='indep',is_sorted=False)
```

```
from scipy import interp

import matplotlib.pyplot as plt

from sklearn.metrics import roc_curve, auc
```

```
from sklearn.model_selection import StratifiedKFold

from sklearn import svm

from sklearn.preprocessing import MinMaxScaler



def autoNorm(dataset):          # normalization for all otu data

    x = dataset[: , :]

    minMax = MinMaxScaler()

    x_std = minMax.fit_transform(x)

    return x_std



random_state = np.random.RandomState(0) # random number generation

cv = StratifiedKFold(n_splits=5)             # fivefold cross-validation

i = 1

df_train = pd.read_csv('')      # Table S14

X = df_train.loc[:,:]      # otu data with otu label

y = df_train.loc[:,]       # overall survival and disease free survival data with label
```

```
all_data=df_train.values # all data

norm_data = autoNorm(all_data[:,1:6])

tprs = [] # mean of false positive,false positive,True positive

aucs = [] # area under curve

mean_fpr = np.linspace(0, 1, 11) # draw diagonal line




# train and test of random

estimators= 25

for train, test in cv.split(X, y):

    classifier = RandomForestClassifier(n_estimators=estimators, max_depth=None,

min_samples_split=2, random_state=0)

    probas_ = classifier.fit(ccc[train,5:11], y[train]).predict_proba(ccc[test,5:11])



# train and test of svm

for train, test in cv.split(X, y):
```

```
	model = svm.SVC(kernel=' sigmoid ', C=1, gamma=5,probability=True, random_state=0)
	probas_ = model.fit(norm_data[train,:], y[train]).predict_proba(norm_data[test,:])


# compute ROC curve and area the curve
	fpr, tpr, thresholds = roc_curve(y[test], probas_[:, 1]) #false positive, true positive
	tprs.append(interp(mean_fpr, fpr, tpr))
	tprs[-1][0] = 0
	roc_auc = auc(fpr, tpr)
	aucs.append(roc_auc)
	plt.plot(fpr, tpr, lw=1, alpha=0.3,label='ROC fold %d (AUC = %0.2f)' % (i, roc_auc))
	i += 1
plt.plot([0, 1], [0, 1], linestyle='--', lw=2, color='r',label='Chance', alpha=.8)
```

```
mean_tpr = np.mean(tprs, axis=0)

mean_tpr[-1] = 1

mean_auc = auc(mean_fpr, mean_tpr)

std_auc = np.std(aucs)

plt.plot(mean_fpr, mean_tpr, color='b',label=r'Mean ROC (AUC = %0.2f $\pm$ %0.2f)' % (mean_auc, std_auc),lw=2, alpha=.5)

std_tpr = np.std(tprs, axis=0)

tprs_upper = np.minimum(mean_tpr + std_tpr, 1)

tprs_lower = np.maximum(mean_tpr - std_tpr, 0)

plt.fill_between(mean_fpr, tprs_lower, tprs_upper, color='grey', alpha=.2,label=r'$\pm$ 1 std. dev.')

plt.xlim([-0.05, 1.05])

plt.ylim([-0.05, 1.05])

plt.xlabel('1-Specificity',fontdict={'family' : 'Times New Roman', 'size':30})

plt.ylabel("Sensitivity",fontdict={'family' : 'Times New Roman', 'size':30})

plt.yticks(fontproperties = 'Times New Roman', size = 23)
```

```
plt.xticks(fontproperties = 'Times New Roman', size = 23)

plt.legend(loc="lower right",fontsize=13)
```

The detailed scripts of RNA-seq data analysis by edgeR

```
library(limma)

library(edgeR)

data <- read.csv(" ",header =F) # read count read matrix

Patient<-
factor(c(1,1,3,3,4,4,5,5,6,6,7,7,8,8,10,10,11,11,12,12,13,13,14,14,15,15,16,16,17,17,1
8,18,19,19,20,20,21,21,22,22,23,23,24,24,25,25,26,26,27,27,28,28,29,29,30,30,31,31,
32,32,33,33,34,34))

Tissue<-
factor(c("A","B","A","B","A","B","A","B","A","B","A","B","A","B","A","B","A","B
","A","B","A","B","A","B","A","B","A","B","A","B","A","B","A","B","A","B","A","
B","A","B","A","B","A","B","A","B","A","B","A","B","A","B","A","B","A","B","A"
,"B","A","B","A","B","A","B"))

y <- DGEList(counts=data)

y <- calcNormFactors(y)
```

```
y$samples

design <- model.matrix(~Patient+Tissue)

ownames(design) <- colnames(y)

rownames(design) <- colnames(y)

design

y <- estimateDisp(y, design, robust=TRUE)

y$common.dispersion

plotBCV(y)

fit <- glmFit(y, design)

lrt <- glmLRT(fit)

topTags(lrt)

results = lrt$table

write.csv (lrt$table,file = "")
```
